# The molecular cartography of malignant and benign sebaceous tumours

**DOI:** 10.1038/s41467-025-66584-0

**Published:** 2025-12-19

**Authors:** I. Ferreira, O. M. Rueda, L. van der Weyden, S. Sahni, O. Cast, K. Wong, M. Del Castillo Velasco-Herrera, H. Caldwell, J. M. Boccacino, T. Alegbe, I. Mehta, A. Gunjur, P. Gupta, V. Harle, K. Koga, I. Matzusaki, M. Fujimoto, K. Wiedemeyer, A. Stratigos, A. Oniscu, K. Wang, E. Ruppin, P. Demetter, I. M. Frayling, M. J. Arends, T. Brenn, D. J. Adams

**Affiliations:** 1 Experimental Cancer Genetics, Wellcome Sanger Institute, Wellcome Genome Campus, Hinxton, United Kingdom; 2 Université Libre de Bruxelles, Brussels, Belgium; 3 MRC Biostatistics Unit, University of Cambridge, Cambridge, United Kingdom; 4 Cancer Data Science Laboratory, National Cancer Institute, National Institutes of Health, Bethesda, United States of America; 5 Cancer Research UK Cambridge Institute, University of Cambridge, Cambridge, United Kingdom; 6 Edinburgh Pathology, Cancer Research UK Scotland Centre, The University of Edinburgh, Institute of Genetics & Cancer, Edinburgh, United Kingdom; 7 Open Targets, Wellcome Sanger Institute, Wellcome Genome Campus, Hinxton, United Kingdom; 8 European Molecular Biology Laboratory, European Bioinformatics Institute (EMBL-EBI), Wellcome Genome Campus, Hinxton, United Kingdom; 9 Department of Pathology, Faculty of Medicine, Fukuoka University Hospital, Fukuoka, Japan; 10 Department of Diagnostic Pathology, Wakayama Medical University, Wakayama, Japan; 11 Department of Diagnostic Pathology, Kyoto University Hospital, Kyoto, Japan; 12 Department of Pathology & Laboratory Medicine, The Arnie Charbonneau Cancer Institute, Cumming School of Medicine, University of Calgary, Calgary, Canada; 13 Departments of Pathology and Dermatology, University of Michigan, Ann Arbor, Michigan USA; 14 1st Department of Dermatology-Venereology, Andreas Sygros Hospital, National and Kapodistrian University of Athens School of Medicine, Athens, Greece; 15 Department of Pathology and Cancer Diagnostics, Karolinska University Hospital Solna, Stockholm, Sweden; 16 Department of Comparative Biosciences, University of Illinois Urbana-Champaign, Urbana, IL USA; 17 Department of Bioengineering, University of Illinois Urbana-Champaign, Urbana, IL USA; 18 Cancer Center at Illinois, University of Illinois Urbana-Champaign, Urbana, IL USA; 19 Department of Pathology, Institut Jules Bordet, Université Libre de Bruxelles, Brussels, Belgium; 20 Laboratory for Experiemental Gastroenterology, Université Libre de Bruxelles, Brussels, Belgium; 21 Pathology department, CerbaPath, Division CMP, Brussels, Belgium; 22 Inherited Tumour Syndromes Research Group, Institute of Cancer & Genetics, School of Medicine, Cardiff University, Cardiff, UK

**Keywords:** Squamous cell carcinoma, Cancer genomics

## Abstract

Sebaceous tumours (STs) are rare skin appendage tumours and include benign sebaceous adenoma (SA) and sebaceoma (SM), malignant extra-ocular sebaceous carcinoma (SC-E) and peri-ocular sebaceous carcinoma (SC-O). Here, an extensive worldwide collection of 286 tumours is deeply characterised, revealing a propensity to develop in the context of a high tumour mutational burden (except in SC-O) which is most frequently associated with mismatch repair deficiency (dMMR), followed by UV-induced damage, *POLE/POLD1* mutations, and AID/APOBEC activation signatures. Biallelic *TP53* inactivation with concomitant *ZNF750* and/or *RB1* mutation is seen in SC-E/SC-O. Amplification of 8q (including *MYC*) is related to SC-O, while amplification of 1q21.3 (including *HRNR*) and chromosome 20 are shared by SC-O and SC-E, as is deletion of 13q14.3 (where *RB1* resides). The most frequently mutated gene is *NOTCH1*. Extensive fusion gene, expression and molecular cluster analyses provide a molecular portrait of this rare and enigmatic tumour type.

## Abstract (web summary)

Sebaceous tumours are a rare skin tumour which have highly variable outcomes. Here, the authors analyse tumours from 222 patients to identify genomic mutations to assess the molecular portrait of the spectrum of tumours.

## Introduction

Sebaceous tumours (STs) are rare cutaneous and peri-ocular neoplasms of sebaceous gland origin, including benign sebaceous adenoma (SA) and sebaceoma (SM), and malignant sebaceous carcinoma (SC). SC is anatomically divided into peri-ocular (SC-O) for tumours located on the eyelid (conjunctiva and/or skin aspect), and extra-ocular (SC-E) for tumours elsewhere on the skin. Clinically, SA and SM typically present as solitary and asymptomatic slow-growing yellow nodules (occasionally ulcerated) on adult seborrhoeic skin areas and are treated by complete excision^1,2^. SC commonly appears as a solitary nodule in adults of 60–79 years^3,4^, with SC-O more common in people of Asian descent (40–60% of eyelid tumours). SC-O and SC-E are associated with significant morbidity and mortality, showing recurrence in 11.8% and 14% of patients, metastasis in 12.5% and 1.8% of cases, and a disease-specific mortality of ∼ 25.6% and ∼ 18%, respectively^3^. The recommended treatment is complete excision (including orbital exenteration), with radiotherapy used for inoperable cases or as an adjunct to surgery^3^.

STs can develop sporadically, as mismatch repair (MMR) proficient (pMMR) or deficient (dMMR) tumours, and may occur in association with Lynch Syndrome (LS), which results from constitutional (germline) pathogenic/disruptive variants in one of four MMR genes; *MSH2*, *MSH6*, *MLH1*, or *PMS2,* with LS-STs deficient for MMR (dMMR) also exhibiting microsatellite instability (MSI)^5^. LS patients have an increased lifetime risk of developing colorectal (CRC) and endometrial cancer, as well as a wide range of other primary cancers, including STs and keratoacanthoma. Muir-Torre Syndrome (MTS) is a subtype of LS that is characterised by at least one visceral LS-associated cancer and one cutaneous sebaceous tumour (except SC-O)^6^.

We perform a large-scale analysis of the sebaceous spectrum integrating whole-exome and transcriptome sequencing data^7–13^ of an extensive globally ascertained cohort including SAs, SMs and SCs totalling 286 cases to provide a comprehensive genomic portrait of these tumours and the molecular events that drive their development.

## Results

### Sample ascertainment and clinico-histopathological features

**Fig. 1:**
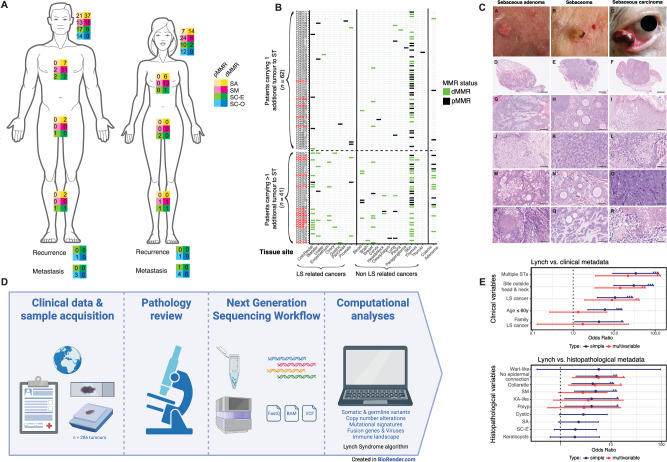
Patient and sample ascertainment and analysis of a multi-centre sebaceous tumour cohort. **A** Distribution of the primary sebaceous tumours (STs) over the body, including head and neck/peri-ocular area, trunk, limbs and genital area (as well as the number of recurrence/metastasis samples), separated by sex and MMR status. **B** Patients (*n* = 103) carrying STs associated with at least one neoplasm defined as a Lynch syndrome (LS) or non-LS related cancer as defined in clinical guidelines^98^. The PD number is the DNA sample identifier, with those in red font being from LS patients. Note: skin includes melanocytic and non-melanocytic skin malignancies, except sebaceous neoplasms. A further description of the histopathology is provided in the Supplementary Discussion in **Supplementary Information**. **C** Clinical and histopathological pictures of sebaceous tumours. Scale bar, 50 µm. **D** Diagram summarising the different process steps including (i) clinical data and samples collection around the world, (ii) pathology review of all the cases included into the study, (iii) next generation sequencing for which the workflow encompasses DNA & RNA extraction, library preparation, sequencing, and alignment, (iv) computational analysis with somatic & germline variants calling, fusion genes identification and immune landscape profiling (https://BioRender.com/n2cymxm). **E** Clinical and morphological variables tested to estimate their predicted potential of association with LS (*n* = 150 patients). Data are presented as the odd ratio (circle) +/− the standard error (error bars). Statistical test (uni- and multi- variable logistic regression with adjustment using the Benjamini-Hochberg method for multiple comparisons): *: two-tailed adjusted *p*-value < 0.05, **: two-tailed adjusted *p*-value < 0.01, ***: two-tailed adjusted *p*-value < 0.001 (exact *p*-value in Source Data Fig. 1E_*p*-value). Variables significant (two-tailed adjusted *p*-value < 0.05) by univariable logistic regression chosen for multivariable logistic regression testing. Abbreviations: *SA* sebaceous adenoma; *SM* sebaceoma; *SC-E* extra-ocular sebaceous carcinoma; *SC-O* peri-ocular sebaceous carcinoma.

Samples from 222 patients (286 tumours, of which 197 primary STs had matched/paired normal DNA) were ascertained from 11 institutions across six countries (Belgium, Canada, Germany, Greece, Japan, United Kingdom). To explore the genetic origin of the patients, their genotypes were projected against the 1000 genomes dataset (Supplementary Fig. 1 in **Supplementary Information**), revealing largely European and Asian descent as expected. Formalin-fixed paraffin-embedded (FFPE) samples were comprised of four ST subtypes, specifically SA (*n* = 102), SM (*n* = 92), SC-E (*n* = 49 primaries and *n* = 1 metastasis), and SC-O (*n* = 26 primaries, *n* = 2 recurrences and *n* = 7 metastases); the latter being underrepresented due to the rarity of this ST subtype, which might limit the power of certain analyses. Additional related lesions were included in the study for comparative purposes, specifically sebaceous hyperplasia (SH, *n* = 6) and a keratoacanthoma with sebaceous differentiation (KA, *n*= 1). For the four ST subtypes (SA, SM, SC-E and SC-O), the median age was 73, 69, 76 and 75 years at diagnosis, with a sex ratio M/F of 2.5/1, 1.04/1, 1.9/1, and 1.2/1, respectively. Most tumours were located on the head and neck (65.8%), followed by the trunk (16.4%), the peri-ocular area (12.6%, of which 9.7% were SC-O), the limbs (3%) and the genital area (1.9%), while one case was from an unspecified site (0.3%). A summary of the clinico-histopathological features of the four ST subtypes, as well as an overview of the project is shown in Fig. 1A–E (Supplementary Data 1 and Supplementary Discussion in **Supplementary Information**).

Immunohistochemistry (IHC) for MMR proteins (MSH2, MSH6, MLH1 and PMS2) was performed on 235 tumours (on at least one tumour per patient). The results of the MMR IHC were used together with indel (insertion/deletion) rate, dMMR mutational signatures, and somatic and germline variant calls to classify the MMR status of tumours (pMMR and dMMR; see “**Methods**”, Supplementary Data 1 and Supplementary Discussion in **Supplementary Information**).

Immunosuppression is associated with an increased incidence of STs, and acquired dMMR can be recognised in some of these tumours^14^. Three out of the six tumours from immunocompromised patients were dMMR in our cohort. Other neoplasms occurred in some patients and were found in individuals who had presented with any of the four ST subtypes (*n* = 103; Fig. 1B, Supplementary Data 1). ST development can be the first clinical sign of LS seen in from 9% and possibly up to 52% of the 23 LS patients in our cohort. Critically, this highlights that the diagnosis of an ST could be used to initiate appropriate cancer screening or potentially prophylactic treatment strategies^15^. To evaluate this possibility further, the clinical and histopathological attributes associated with LS were examined in our cohort using univariable and multivariable logistic regression. Of 6 clinical attributes, developing multiple STs, having a tumour outside the head and neck, and having another non-ST LS-related neoplasm were all significantly associated with LS after multivariable testing and following false-discovery correction (multivariable odds ratios of 22, 14, 8.8, respectively; Fig. 1E and Supplementary Discussion in **Supplementary Information**). Ten histopathological features were also tested. No epidermal connection and delimitation of the ST by a collarette appeared to be statistically significantly related to LS on multivariable testing and after false-discovery correction (multivariable odds ratio 5.6 and 4.8, respectively; Fig. 1E and Supplementary Discussion in **Supplementary Information**).

Follow-up was obtained for 179 patients (mean: 39 months, median: 26 months; range: 1–144 months), and from the clinical data it was observed that SC-O patients had a worse prognosis than SC-E patients; recurrence and metastasis occurred in 7.7% and 19.2% of the SC-O patients after an average time of 8.5 and 48 months compared with 2.2% and 8.7% of the SC-E patients after an average time of 21 and 16.2 months, respectively (Supplementary Data 1). Of interest, metastasis occurred to lymph nodes for SC-E, while lymph node, parotid gland, lung and brain metastasis was observed for SC-O. Tumour-related death occurred in 7.7% and 4.3% of the SC-O and SC-E patients, respectively. A summary of the individual sample details is provided in Supplementary Data 1.

### Somatic landscape of sebaceous tumours

**Fig. 2:**
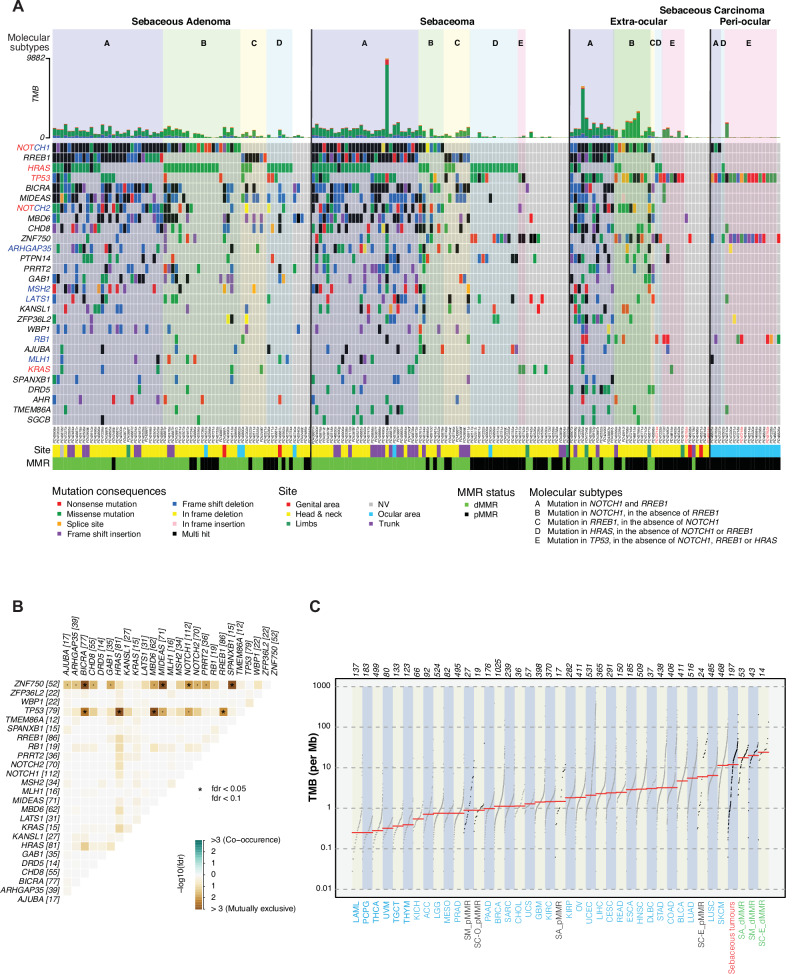
The somatic mutational landscape of sebaceous tumours across subtypes. **A** Oncoplot showing somatic mutations in the driver genes of sebaceous tumours (STs). This includes the 25 driver genes identified when taking all four ST subtypes together, and three additional driver genes when considering only specific ST subtypes and/or MMR status. Gene names in red denote oncogenes, genes in blue denote tumour suppressors, while gene names in both colours are those reported to have both functions depending on the context. Patterns of mutations in the top 4 driver genes (*NOTCH1, RREB1, TP53* and *HRAS*) allow division of the STs into five molecular subtypes (**A**–**E**) across the four ST subtypes. Site is the body location of the tumour. The PD number is the DNA sample identifier, with those in red font being from patients that developed metastases. **B** Mutual exclusive and co-occurring interactions based on DISCOVER. **C** Comparison of the tumour mutation burden (TMB) of the sebaceous tumours (STs; *n* = 197 samples, as indicated on the top *X*-axis) against 33 TCGA cohorts (TCGA tumour types are shown using standard abbreviation). The ST cohort are shown all together (blue font), or separated by subtype and mismatch repair (MMR) status (green and red font). Abbreviations: *MMR* mismatch repair; *dMMR* deficient in MMR; *pMMR* proficient in MMR; *NV* not available; *TMB* tumour mutational burden (in mutations/Mb, including SNVs, MNVs and indels).

Whole-exome sequencing (WES) was performed to profile somatic mutations, somatic copy number alterations (SCNAs) and mutational signatures. The tumour mutational burden (TMB; including single nucleotide variants (SNVs), multi-nucleotide variants (MNVs) and insertions/deletions (indels)) varied markedly across the cohort. Using mutation calls from the 197 primary STs where a germline matched normal sample had also been sequenced, we found a statistically significantly higher TMB for dMMR STs compared to all other tumours in The Cancer Genome Atlas (TCGA) dataset, and dMMR STs also had a much higher TMB compared to pMMR STs (linear model with pairwise comparisons using Tukey’s method; Fig. 2A–C and Supplementary Data 2). dMMR STs also showed a higher TMB than a range of skin tumours sequenced by MSK-impact (linear model with pairwise comparisons using Tukey’s method; Supplementary Data 2). Interestingly, indel rate (Area Under the Curve; AUC 0.999) was a better predictor of dMMR status compared with TMB (AUC 0.887), as there were pMMR tumours with a low indel rate and high TMB (Supplementary Fig. 2A in **Supplementary Information**). Importantly, for this specific analysis, we defined dMMR cases as those having lost one of the MMR proteins by immunohistochemistry and also carrying biallelic inactivating events in an MMR gene. We did not use the indel or SNV rate (see “**Methods**”). Using a threshold of 0.1 on the predicted probability of dMMR computed with our model, a rate of > 0.345 indels/Mb will give 97% sensitivity and 99% specificity to predict dMMR. However, this threshold is specific to our pipelines, as the sensitivity and accuracy of mutation calling is an inherent property of each variant calling pipeline. Amongst the different ST subtypes, SC-Es had the highest TMB regardless of MMR status, while SC-Os (which were all pMMR) had a TMB comparable to that of benign pMMR tumours (Supplementary Data 3). Interestingly, pauci-mutated samples (with < 5 mutations/Mb) were found across all four ST subtypes, usually related to pMMR status, however, in SC-O, pauci-mutation was the predominant phenotype (Supplementary Fig. 2B in **Supplementary Information** and Supplementary Data 3).

The complete list of somatic variants is provided in Supplementary Data 4 (with cross-validation using RNAseq data provided in Supplementary Fig. 4A in **Supplementary Information** and see “**Methods**”). Considering only the paired primary tumours (*n* = 70 SA, *n* = 70 SM, *n* = 38 SC-E and *n* = 19 SC-O; *n* = 197 in total), the top 20 recurrently mutated genes mainly consisted of known cancer-associated genes (Supplementary Fig. 3A, B in **Supplementary Information**). Driver gene analysis of the somatic variants across all four ST subtypes (using dNdScv; see “**Methods**”) identified 25 statistically significantly mutated genes (Fig. 2A, Supplementary Data 5 and Supplementary Fig. 3C in **Supplementary Information** includes the unpaired tumours). Additional driver genes were identified when considering only specific ST subtypes and/or MMR status, such as *PRRT2* in dMMR tumours, *DRD5* in dMMR SC-Es and *SPANXB1* in dMMR-MSH2/MSH6-negative SC-Es (Fig. 2A, Supplementary Fig. 3C in **Supplementary Information** and Supplementary Data 5A). To better understand the role of these driver genes in STs, the variant types they carry (Supplementary Fig. 4B in **Supplementary Information**), their presence in SCNAs (Supplementary Fig. 4C in **Supplementary Information**) and their predicted mechanism of contribution to tumourigenesis (oncogene versus tumour suppressor gene (TSG); Supplementary Fig. 4D in **Supplementary Information**), and their variant allele frequency (VAF; Supplementary Fig. 4E in **Supplementary Information**) were considered. *HRAS*, *TMEM86A* and *SGCB* were characterised by missense mutation only, suggesting they are acting as oncogenes; while most driver genes show a range of variant types (including indels and truncating mutations), more suggestive of a TSG profile^16^. Similarly, several established oncogenes were amplified (such as *KRAS*), while loss and copy neutral loss of heterozygosity (cnLOH) were identified at tumour suppressor gene loci, such as *MSH2* and *RB1*. In addition, these 28 driver genes were cross-referenced with pan-cancer analyses^17^, to better understand their impact and activities in other cancers (Supplementary Fig. 4F in **Supplementary Information**); 15 out of the 28 driver genes (53.6%) identified by dNdScv were shared with genes in this pan-cancer drive gene resource. Additionally, reduced hypothesis testing was also performed with 312 previously defined cancer driver genes from Bailey et al.^17^, allowing us to identify further potential driver genes (Supplementary Fig. 5 in **Supplementary Information** and Supplementary Data 5B). Lastly, using another tool (OncoDriveFML), further candidate driver genes were called (see **GitHub repository**), while 22 out of the 25 drivers genes (88%) found by dNdScv were also found with this method. 

The transmembrane receptor *NOTCH1* was the most recurrently mutated driver gene (*n* = 112/197), with many tumours having multiple hits (*n* = 67/112). The mutations predominantly occurred in the extracellular epidermal growth factor (EGF) domain (*n* = 87/112; 70/87 of these tumours were dMMR), with a hotspot at p.A465T identified (*n* = 10; COSM1217602; Supplementary Fig. 6A in **Supplementary Information**). *NOTCH1* can function as both a tumour suppressor gene and oncogene, depending on the cellular context^18^, however, the mutations observed in STs were primarily inactivating and this suggests that *NOTCH1* predominantly functions as a TSG. The next most recurrently mutated driver gene was the transcription factor *RAS responsive element binding protein-1* (*RREB1*; *n* = 86/197; Supplementary Fig. 6B in **Supplementary Information**), with many tumours having multiple mutations (*n* = 47/86). Tumours with *RREB1* mutations predominantly (but not exclusively) also had *NOTCH1* mutations (*n* = 71/86). The *HRAS* oncogene was the third most recurrently mutated driver gene (*n* = 81/197), with predominantly missense mutations in the well-established hotspots of exon 2, p.G12C/D/R/S (*n* = 19/81) and p.G13D/R/S/V (*n* = 17/81), and exon 3, p.Q61H/K/R (*n* = 10/81), as well as other less well known variants that are reported in COSMIC, such as p.K117N (*n* = 12/81), p.A18V (*n* = 11/81) and p.A146T (*n* = 5/81; Supplementary Fig. 6C in **Supplementary Information** and Supplementary Data 4). Interestingly, when considering the ST subtypes individually, *HRAS* was only identified as a statistically significant driver gene in the benign tumours. Notably, hotspot *HRAS* mutations were observed in malignant subtypes, but these mutations occurred at a much lower mutation frequency in these smaller cohorts (Supplementary Data 5A). In addition, specific variants of *HRAS* were found to associate with MMR status, as pMMR tumours had p.G13R/V or p.Q61K/R variants, whilst dMMR tumours specifically harboured p.G12S, p.A18V, p.R68Q/W and p.A146P/T/V variants (with the p.K117N/R variants being associated with either MMR status). Another gene found to associate with MMR status was the BAP1 complex regulator *methyl-CpG-binding domain protein 6* (*MBD6*), in which mutations (predominantly frameshift insertions or deletions, with hotspot mutations at p.A783Sfs*10 (*n* = 24), p.G782Efs*13 (*n* = 17), p.Q694Sfs*5 (*n* = 10) and p.Q734Nfs*18 (*n* = 6)) were only found in dMMR tumours (*n* = 62/197, except for one pMMR SC-E). The fourth most recurrently mutated driver gene was *TP53* (*n* = 79/197), with missense mutations predominating (*n* = 36/79).

The presence of mutations in the top four most mutated driver genes allowed the identification of five molecular subtypes, specifically those with *NOTCH1* mutations in the presence of *RREB1* mutations (molecular subtype A), *NOTCH1* mutations in the absence of *RREB1* mutations (molecular subtype B), *RREB1* mutations in the absence of *NOTCH1* mutations (molecular subtype C), *HRAS* mutations in the absence of *NOTCH1* or *RREB1* mutations (molecular subtype D), and *TP53* mutations in the absence of *NOTCH1* and *RREB1* mutations (molecular subtype E); highlighting a shared mutational profile amongst the four ST subtypes despite their histological differences (Fig. 2A). Whilst molecular subtypes A, D and E could be identified in SC-O, they also carried mutation of *TP53* (*n* = 18/19) and the transcription factor *ZNF750* (*n* = 13/19; *TP53* and *ZNF750*, *n* = 12/19; Fig. 2A), which was also seen in the metastatic SC-Os (Supplementary Fig. 3B in **Supplementary Information**). This is consistent with a recent exome study of five eyelid SCs^19^. A proportion of SC-Es also had mutations in both *TP53* and *ZNF750* (*n* = 10/38). Conversely, the *dopamine receptor 5* (*DRD5*) gene was mainly identified as a driver gene in dMMR SC-Es associated with both *NOTCH1-RREB1* mutation (mutated in 9/38; Supplementary Data 5A). Finally, both SC-E and SC-O showed recurrent mutations in the TSG, *RB1* (*n* = 8/38 and *n* = 6/19, respectively), in contrast to a previous report in which *RB1* mutations were only found in SC-O (not SC-E)^10^. Of note, both *ZNF750* and *RB1* mutations were largely associated with *TP53* mutation in SC (in 85% and 86% of cases, respectively, of SC-E and SC-O cases combined (Fig. 2A, B). In addition, concomitant *TP53/RB1* mutations were identified in metastatic SCs. When stratifying by ST subtypes, and using DISCOVER^20^, three mutually exclusive interactions were identified (*ZNF750* remained mutually exclusive from *RREB1*, *MIDEAS* and *BICRA* (*q*-value = 0.038 for each)), which were also seen amongst the 17 mutually exclusive interactions observed in the unstratified analysis. Unstratified DISCOVER analysis (see “**Methods**”) also found that *NOTCH1* (*q*-value = 0.015), *RREB1* (*q*-value = 0.008) and *HRAS* (*q*-value = 0.001) mutations were mutually exclusively from *TP53* mutations, and *NOTCH1* (*q*-value = 0.010) mutations were mutually exclusively from *ZNF750* mutations, confirming the separation of the STs into five molecular subtypes, especially as molecular subtype E has additional driver gene mutual exclusivity (Fig. 2B and Supplementary Data 6).

**Fig. 3:**
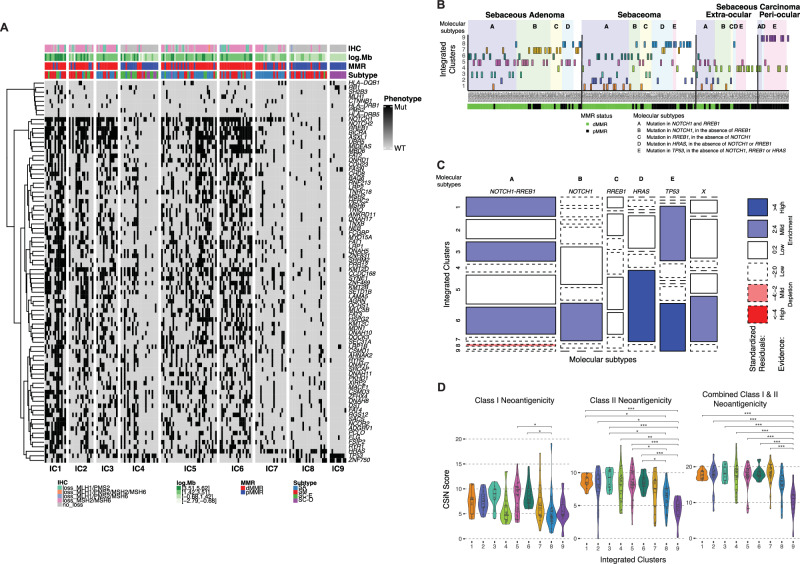
Integrative clustering in sebaceous tumours defines molecular sub-groups. Based on somatic variants (**A**) and expression data (Supplementary Fig. 7A) in **Supplementary Information**, MOFA^78^ was used to fit a regularised latent variable model-based clustering. Nine integrative clusters (ICs) were selected as shown in (**A**), associated with clinical details (immunohistochemistry (IHC) status, tumour mutational burden (log_Mb), MMR status, and ST tumour subtypes (*n* = 170)). These 9 ICs were then correlated with the 5 molecular subtypes (**B**), and their enrichment into each of these molecular subtypes were analysed (standardised residuals) (**C**). **D** Violin boxplots showing correlation between predicted neoantigenicity and integrative clusters for class-I – class-II and combined classes (class-I & class-II). Boxplot annotations: centre line, median; box limits, upper (75^th^) and lower (25^th^) quartiles; whiskers, 1.5 x interquartile range; dots, values. Abbreviations: *IHC* immunohistochemistry; *MMR* mismatch repair; *dMMR* deficient in MMR; *pMMR* proficient in MMR; Mut, mutant;* SA* sebaceous adenoma; *SM* sebaceoma; *SC-E* extra-ocular sebaceous carcinoma; *SC-O* peri-ocular sebaceous carcinoma;* log.Mb* tumour mutational burden; *WT* wild-type; *X* unassigned tumours. Statistical test (ANOVA test followed by Tukey Honest Significant Differences): *: *p*-value < 0.05, **: *p*-value < 0.01, ***: *p*-value < 0.001 (exact *p*-values in Source Data Fig. 3D_*p*-value).

Integrative cluster analysis combining the somatic mutation and transcriptional profiles (see **GitHub repository**) of the 170 STs for which both exome/DNA and transcriptome/RNA analyses had been performed (see “**Methods**”), identified 9 integrative clusters (IC; Fig. 3, Supplementary Fig. 7 in **Supplementary Information** and Supplementary Data 7), each characterised by distinct clinicopathological features, indel/SNV rates, somatically mutated genes, gene expression/pathway enrichment, and tumour microenvironment (TME) features. Interestingly, these individual ICs shared many features with the individual molecular subtypes. For example, IC1 – IC3 and IC6 overlapped with molecular subtype A (standardised residuals 2:4 each), which is characterised by a high proportion of mutations in driver genes such as *NOTCH1* and *RREB1* (with or without *BICRA* and *MIDEAS* mutations) and was found mostly in benign dMMR tumours. They also contained a high proportion of *ASXL1* mutations in addition to *NOTCH2* in IC3 and IC6, and *MBD6* in IC6. Interestingly, these three ICs, driven either by mutational burden (IC1 and IC6) or indel burden (IC3), showed different pathway enrichments and a different tumour microenvironment, with IC1 having up-regulation of G2M checkpoint and E2F targets, and containing a high proportion of CD8 + T cells and fibroblasts, IC3 being down-regulated of most hallmark pathways and showing a high proportion of CD4 + T cells, and IC6 being up-regulated in the *MYC* pathway; suggesting possible differences in the tumour behaviour or cellular makeup in each molecular subtype. IC7 showed similarities with *NOTCH1* mutated molecular subtype B (standardised residuals 2:4). Displaying a lower mutational burden profile, this IC (IC7), mostly encountered in pMMR and dMMR SAs, is mutated for *NOTCH1* and *HRAS* in 50% of cases, showed a high proportion of endothelial cells and fibroblasts, and is down-regulated for interferon pathways. Interestingly, IC8 differed from molecular subtype A (standardised residuals −4:−2) and overlapped with molecular subtype D (standardised residuals > 4). Mostly composed of pMMR benign STs, IC8 is characterised by a low mutational burden with mutations in *HRAS*, *ZNF750* and *TP53* occurring in 40 to 50% of cases, the contribution of UV related mutational signatures, and down-regulation of many pathways. Finally, molecular subtype E was linked to IC4 and IC9 (standardised residuals 2:4 and >4, respectively), both showing a high proportion of mutation of *TP53* and larger copy number alterations (at a chromosomal arm level) compared to the other ICs. IC4 exhibited additional *ZNF750* and/or *NOTCH1* mutations in 40% of cases and was mostly composed of pMMR SCs (SC-Es predominantly), included a high population of B and T cells, showed UV and APOBEC related mutational signatures, and up-regulation of the interferon response. In contrast, IC9 cases had an even higher proportion of mutations in *ZNF750* with some cases also mutated for *RB1*. These cases were exclusively pauci-mutated (pMMR) SC-Os and showed up-regulated of E2F and G2M checkpoint pathways, and a lower immune component.

### Mutational signatures of sebaceous tumours

**Fig. 4:**
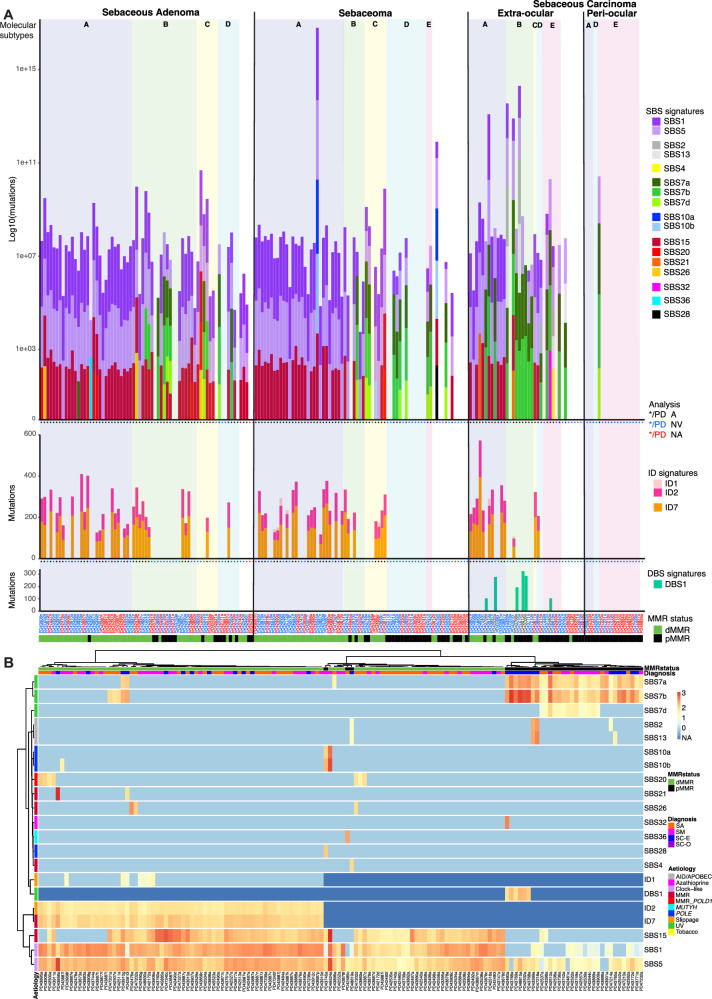
Mutational signatures of sebaceous tumours. **A** Barplot showing the mutational signature profiles, including SBS (single-base substitutions), ID (insertions and deletions) and DBS (doublet-base substitutions) signatures, across each of the four sebaceous tumour subtypes. PD numbers refer to the DNA sample identifiers, while the asterisk denotes samples with/without SBS or ID signatures. A black asterisk or a PD number in black (*/PD A) indicates that a signature was detected in that sample, while a blue (*/PD NV) a or red asterisk or PD number (*/PD NA) indicates samples where no mutational signature was detected, or samples were not analysed, respectively. COSMIC signature groups: SBS: clock-like signatures (SBS1 and SBS5), AID/APOBEC activation signatures (SBS2 and SBS13), the signature associated with tobacco smoking (SBS4). UV light signatures (SBS7a, SBS7b, SBS7d), *POLE* exonuclease domain mutation signatures (SBS10a and SBS10b), dMMR signatures (SBS15, SBS20, SBS21 and SBS26; SBS20 is linked to dMMR and *POLD1* mutation), the signature associated with prior azathioprine treatment (SBS32), *MUTYH* signature (SBS36), signature with unknown aetiology (SBS28). ID: DNA replication slippage signatures (ID1 and ID2), dMMR signature (ID7). DBS: UV light signature (DBS1). **B** Clustered heatmap of each signature log10 counts (as rows) in each sample (columns) grouping based on the 3 main mutational profiles within this cohort: MMR (SBS15 + ID7), UV (DBS1 + SBS7a + SBS7b) and others (AID/APOBEC activation, *POLE* mutation). Abbreviations: *SA* sebaceous adenoma; *SM* sebaceoma; *SC-E* extra-ocular sebaceous carcinoma; *SC-O* peri-ocular sebaceous carcinoma.

Mutational signatures are characteristic patterns of somatic single-base substitutions (SBS), doublet-base substitutions (DBS) and small indels (ID) that reflect underlying mutational processes^21^. As expected, in the dMMR cases, the high TMB was attributed to dMMR-associated mutational signatures, specifically SBS15, SBS20, SBS21, SBS26 and ID7 (Fig. 4A, B, Supplementary Fig. 8 in **Supplementary Information** and Supplementary Data 8), consistent with previous reports^10,22^. Interestingly, most tumours with ID7 signatures also had ID2 signatures; ID2 is associated with slippage during DNA replication of the template DNA strand and tends to be highly elevated in dMMR cancer samples^21^. Signatures associated with ultraviolet (UV) light exposure, specifically SBS7a, SBS7b, SBS7d and DBS1^23,24^, were also associated with a high TMB and were identified in a proportion of samples from all four ST subtypes (DBS1 being identified in SC-E), validating previous observations^10,22^. However, SBS7 was most notably found in the SC-Es (*n* = 16/38, of which 6 also showed a DBS1 mutational signature; all were from sun-exposed sites, and *n* = 14/16 were pMMR). Importantly, one case (PD42510a) of the 19 SC-Os harboured UV signatures, and while all other SC-Os were from the eyelid or eye, this sample was from the canthus (Supplementary Fig. 9 in **Supplementary Information**). Of note, signatures SBS2 and SBS13, which are attributed to the activity of the AID/APOBEC family of cytidine deaminases, were found in some pMMR SC-Es (*n* = 5/38; all of which were from sun-exposed sites and pMMR; Fig. 4A, B).

Interestingly, one sample with dMMR-associated signatures and a high SNV/indel rate (PD45524a; Fig. 4A) was also found to have signatures SBS10a and 10b, which are associated with *POLE* exonuclease proofreading defects. In CRC, *POLE* mutation results in a large number of somatic mutations (>100 mutations/Mb) and are described as ultramutated^25^ as compared to the dMMR-associated hypermutated samples^21^. Importantly, this sample had somatic mutations in the exonuclease domain (ED) of *POLE* (*n* = 2) and *POLD1* (*n* = 1), as well as in *MSH2* (*n* = 1) and *MSH6* (*n* = 3) and 273 mutations/Mb. Four other samples had one somatic mutation each in the ED of *POLE* (PD43395a, PD43745a, PD43910d, PD47239a), however, SBS10a/b mutational signatures were only identified in one of them (PD43745a). Similarly, signature SBS20, which is associated with dMMR in the context of concurrent *POLD1* mutation^26^, was identified in seven tumours, however, in four tumours a *POLD1* mutation could not be found (PD43911a, PD47202d, PD42500a, PD43869a). STs associated with somatic *POLD1* and *POLE* mutations and/or their related mutational signatures have never been reported before. One sample was found to have signature SBS36, which is correlated with defective DNA base excision repair due to disruption of *MUTYH* (germline or somatic)^27^, and a homozygous *MUTYH* pathogenic allele was identified in this case (germline; PD42485c). Interestingly, signature SBS32, which is linked to prior treatment with the immunosuppressive drug azathioprine^28^, was attributed to a SC-E from a renal transplant patient (PD42480a).

### Copy number landscape of sebaceous tumours

**Fig. 5:**
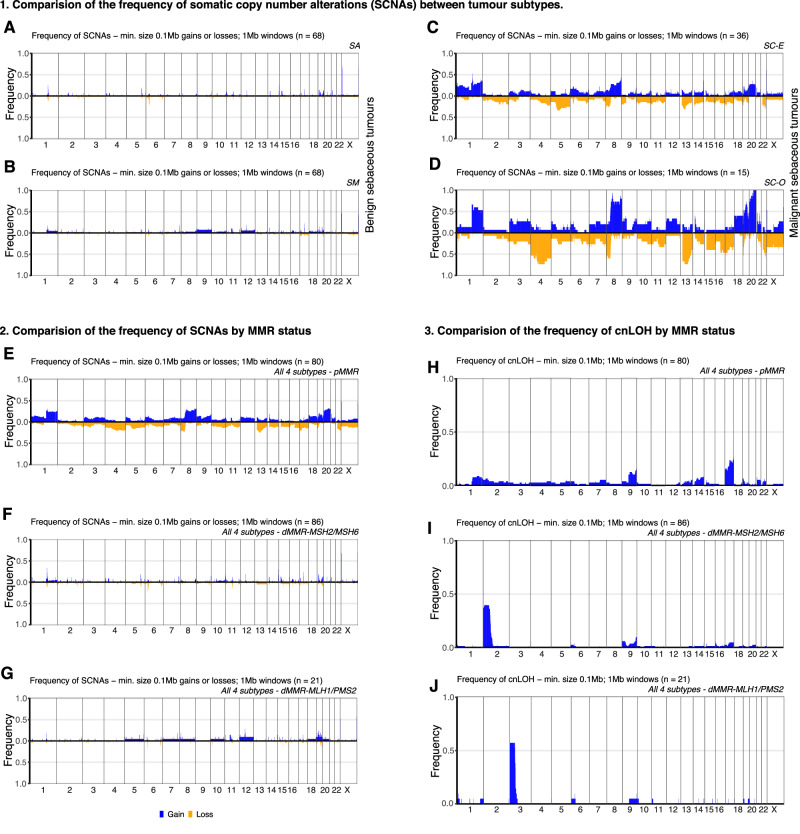
Somatic copy number alterations in sebaceous tumours. Penetrance plots of somatic copy number alterations in (**A**) sebaceous adenoma (SA), (**B**) sebaceoma (SM), (**C**) extra-ocular sebaceous carcinoma (SC-E), (**D**) peri-ocular sebaceous carcinoma (SC-O), (**E**) mismatch repair proficient (pMMR) sebaceous tumours (STs), (**F**), mismatch repair deficient (dMMR) STs showing loss of MSH2/MSH6 by immunohistochemistry, and (**G**) dMMR STs showing loss of MLH1/PMS2 by immunohistochemistry. Frequency plots of copy neutral loss of heterozygosity (cnLOH) in (**H**) pMMR STs, (**I**) dMMR STs showing loss of MSH2/MSH6 by immunohistochemistry, and (**J**) dMMR STs showing loss of MLH1/PMS2 by immunohistochemistry. Abbreviations: dMMR-*MSH2/MSH6* deficient in MMR with MSH2/MSH6 loss of expression by immunohistochemistry; dMMR-*MLH1/PMS2* deficient in MMR with MLH1/PMS2 loss of expression by immunohistochemistry.

Analysis of somatic copy number alterations (SCNAs) revealed distinct profiles between the four ST subtypes (Fig. 5). The benign tumours had relatively few chromosomal aberrations (Fig. 5A, B), whereas the malignant tumours were replete with large chromosomal gains and losses (Fig. 5C, D). There was overlap in the SCNA profiles of the malignant tumour subtypes, with both showing recurrent copy number (CN) gains of chromosomes 1, 8q and 20 and CN loss of chromosomes 4, 8p and 13q, however, the frequency of these CN aberrations was higher in SC-O than SC-E (Fig. 5C, D).

When taking all four ST subtypes together and separating them based on MMR status, SCNAs predominantly occurred in the pMMR tumours (Fig. 5E), as the dMMR tumours with loss of MSH2/MSH6 or MLH1/PMS2 expression by immunohistochemistry showed relatively few SCNAs (Fig. 5F, G). This suggests two different types of genomic instability occur with little overlap, specifically chromosomal instability in pMMR tumours and microsatellite instability (MSI) in dMMR tumours. Of note, regions of recurrent cnLOH differed based on the tumours MMR status. For example, recurrent chromosome 17 cnLOH was observed in the pMMR cases (Fig. 5H), with *TP53* cnLOH seen in SC-Os (*n* = 5/15), SC-Es (*n* = 6/36) and SAs (*n* = 3/68), and *ZNF750* cnLOH observed in SC-Os (*n* = 8/15), SC-Es (*n* = 6/36), SMs (*n* = 11/68) and SAs (*n* = 2/68) (Supplementary Data 9). *TP53* and *ZNF750* cnLOH resulted in biallelic inactivation in 100% and 81.5% of cases, respectively, as these tumours also had a somatic mutation in these genes. Importantly, *TP53* inactivation by cnLOH has been reported in several cancer types^29–34^; it has never been reported in STs. Recurrent *MSH2* and *MSH6* cnLOH was identified in 38/187 tumours (Fig. 5I), of which 89.5% were dMMR with loss of expression of MSH2/MSH6 by immunohistochemistry. Of these tumours, 66% were from LS patients with a constitutional (germline) disruptive/pathogenic *MSH2* variant (Supplementary Data 9). Similarly, *MLH1* cnLOH was observed in 11 tumours (Fig. 5J), out of the 21 dMMR tumours with a loss of MLH1 expression, with one tumour coming from an LS patient. Of note, two pMMR SCs (one SC-E and one SC-O) with no loss of MMR expression by immunohistochemistry exhibited *MLH1* cnLOH. *PMS2* cnLOH was identified in one pMMR SC-O. MMR inactivation through *MLH1* and *MSH2* cnLOH have been reported in MSI CRC cell lines and/or tumours^35–37^. Lastly, chromosome 9q cnLOH, including *CDKN2A* (*n* = 8/187) and/or *NOTCH1* (*n* = 20/187; mostly mutated for *NOTCH1*), was identified predominantly in pMMR tumours, and to a lesser extent in dMMR tumours (Fig. 5H and Supplementary Data 9). Interestingly, 9q cnLOH has been previously reported in normal skin^38^.

**Fig. 6:**
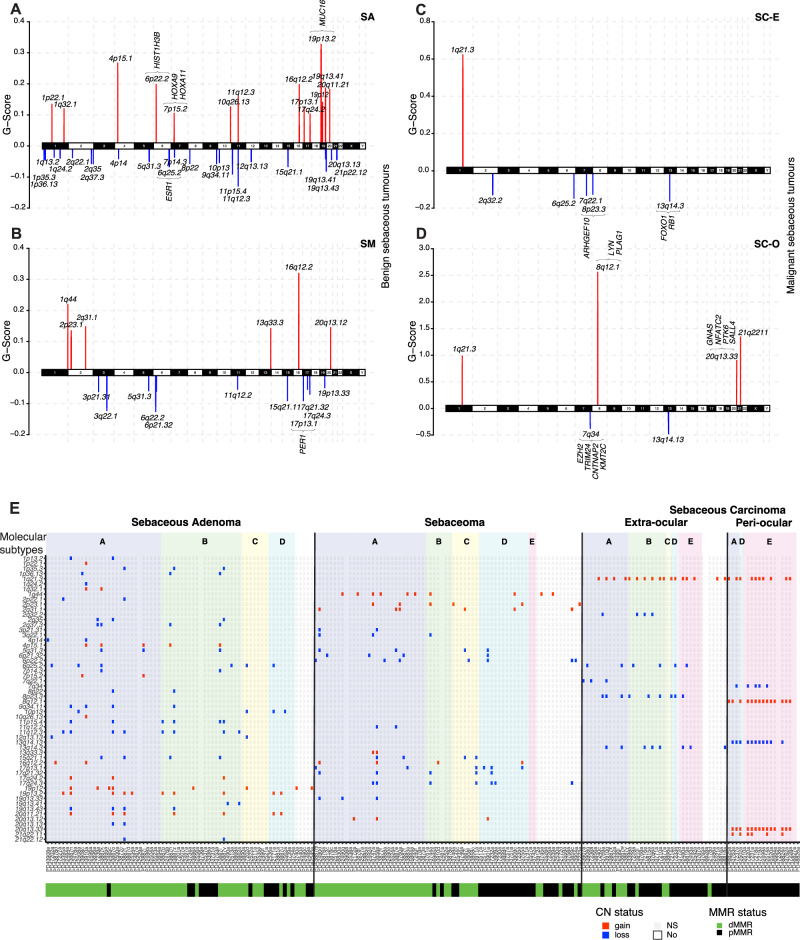
Significant focal somatic copy number alterations in sebaceous tumours. Representation of the GISTIC2.0-identified by ST subtype significant focal peak regions (≤ half a chromosome arm) in (**A**) sebaceous adenoma (SA), (**B**) sebaceoma (SM), (**C**) extra-ocular sebaceous carcinoma (SC-E) and (**D**) peri-ocular sebaceous carcinoma (SC-O). Chromosomal location of the peaks is indicated by the cytoband, with amplifications (gains) shown in red and deletions (losses) shown in blue. The presence of known tumour suppressor genes or oncogenes within these peak regions are labelled. For the G-score, positive values refer to gains, while negative values are related to deletions. **E** Oncoplot showing the individual tumour samples with the amplification and deletion regions shown in (**A**–**D**). Samples with no significant gains or losses are indicated by NS. Five samples were not included in the GISTIC2.0 analysis (see “**Methods**”), so no data is shown (indicated by No). Abbreviations: *MMR* mismatch repair; *dMMR* deficient in MMR; No, not analysed; *NS* not significant; *pMMR* proficient in MMR; *SA* sebaceous adenoma; *SM* sebaceoma; *SC-E* extra-ocular sebaceous carcinoma; *SC-O* peri-ocular sebaceous carcinoma. Note: *focal peaks were considered as significant when reaching the 4 criteria: 1. residual *q*-value < 0.1, 2. peak size ≥ 100,000 bp, 3. concordance with ASCAT CN calls ≥ 0.75, 4. portion of peak overlap with Genome In A Bottle difficult regions ≤ 0.4 (Supplementary Data 10); **approximate cytobands are shown with related genomic coordinates available in Supplementary Data 10.

To identify candidate driver genes in regions of CN gain or loss, both focal regions (≤half a chromosome arm; Supplementary Data 10) and broad regions (>half a chromosome arm; Supplementary Data 11) were considered. Focal peaks were defined as significant when reaching the 4 criteria: (i) GISTIC2 residual *q*-value < 0.1, (ii) peak size ≥ 100,000 bp, (iii) concordance with ASCAT CN calls ≥ 0.75, (iv) portion of peak not overlapping with Genome In A Bottle difficult regions by > 40% (Supplementary Data 10); while broad peaks were determined as significant when reaching the 3 criteria: (i) GISTIC2 residual *q*-value < 0.1, (ii) concordance with ASCAT CN calls ≥ 0.75, (iii) gain with log ratio > 0.25 or loss with log ratio < − 0.25 (Supplementary Data 11 and see “**Methods**”). There were no significant focal or broad SCNAs shared between the benign and malignant tumours. SC-Os showed the greatest number of focal SCNAs (Fig. 6). Oncogenes in these regions included *GNAS*, *NFATC2*, *PTK6* and *SALL4* (*n* = 14/15 SC-Os), and *LYN* and *PLAG1* (*n* = 14/15 SC-Os). Deletions were also observed in the TSGs *EZH2*, *TRIM24*, *CNTNAP2* and *KMT2C* (*n* = 5/15 SC-Os). Of note, the focal SCNAs in the SC-Es only included known TSGs, specifically *FOXO1* and *RB1* (*n* = 8/36), and *ARHGEF10* (*n* = 9/36). Interestingly, a significant focal amplification was seen at 1q21.3 in SCs (*n* = 17/36 SC-Es and *n* = 10/15 SC-Os), which contains *HRNR* (Hornerin, a S100 family protein; Fig. 6 and Supplementary Data 10). Similarly, a significant focal deletion at 13q14.13 was observed in 11/15 SC-Os, including no known TSGs (Fig. 6 and Supplementary Data 10).

At a chromosome arm level, SC-Os showed the greatest number of significant recurrent SCNAs, including chromosomes 1q, 8q, 19p and 20 amplifications, as well as chromosome 4 and 13q deletions (Supplementary Fig. 10A–D in **Supplementary Information**), with the 13q deletions encompassing *RB1* and *BRCA2* (*n* = 8/15; one of them being somatically mutated for *RB1*; Supplementary Fig. 10E in **Supplementary Information**). In contrast, SC-Es showed significant chromosomes 1 and 20 amplification, and significant 17p deletions, which encompassed *TP53* (*n* = 7/36 tumours, of which 6 also had a somatic *TP53* mutations; Supplementary Data 11).

### Germline alleles in sebaceous tumour patients

**Fig. 7:**
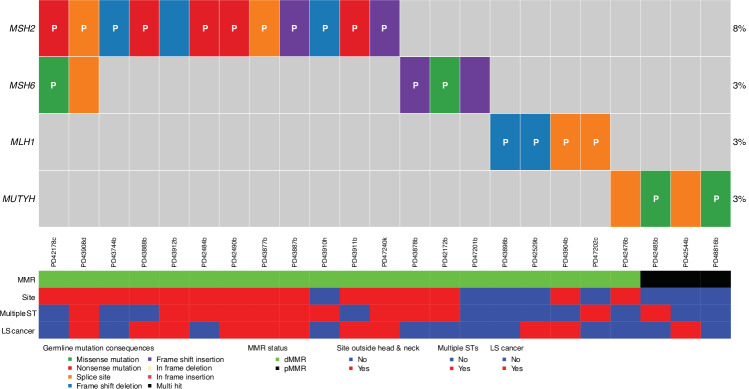
Germline variant landscape of sebaceous tumour patients. Oncoplot showing the variants present in the normal samples. Constitutional (germline) variants or those with disruptive consequences (i.e., consequences classified as frameshift, nonsense or splice site by VEP, or variants defined as pathogenic/likely-pathogenic by ClinVar) are shown. Only genes included in the NHS Cancer National Genomic Test Directory and mutated in at least two patients are shown. Abbreviations: *P* variant defined as pathogenic/likely-pathogenic in the ClinVar database.

Putative pathogenic constitutional (germline) variants (SNVs and indels) were inspected in each of the four ST subtypes (*n* = 154 patients/197 samples; Supplementary Data 12). To focus on clinically relevant genes, only genes known to be associated with cancer-predisposition were examined (see “**Methods**”) and the variants were filtered to consider only disruptive variants, specifically, frameshift, nonsense and splice site variants (as annotated by Variant Effect Predictor (VEP)^39^) and/or alleles reported as pathogenic/likely pathogenic (as defined by ClinVar^40^). Using these criteria, the most recurrently mutated gene was *MSH2* (*n* = 12/154; 8% patients) with ClinVar clinical significance defined pathogenic/likely-pathogenic variants in 11 patients (Fig. 7). The remaining patient carrier of an *MSH2* germline variant, which has been reported as non-pathogenic/likely pathogenic in ClinVar, developed multiple STs as well as Lynch-associated internal cancers (PD43912), suggesting the variant (p.Ser153ProfsTer21) in this individual may be disruptive and thus disease causing. Interestingly, two patients carried ≥2 variants in MMR genes; patients with variants in more than one MMR gene are termed digenic/double heterozygous LS, and whilst it is not yet clear if this results in a more severe form of LS, there are clear implications for genetic counselling^41,42^. Lynch-like syndrome (LLS) patients develop LS-associated neoplasms (in particular CRC) but do not carry constitutional variants in MMR genes, or somatic *BRAF* mutations or *MLH1* hypermethylation in CRCs^43^ they develop. A proportion of these cases are caused by biallelic *MUTYH* inactivation^44,45^. In our ST cohort, four patients carried a disruptive/pathogenic constitutional variant in *MUTYH* (Fig. 7). Three of these tumours were pMMR; two of the patients developed colonic neoplasia (adenoma or CRC), consistent with the LLS phenotype, one of them (PD42485b) having a homozygous genotype.

Lastly, other ClinVar disruptive/(likely)pathogenic variants were identified in several cancer predisposition genes, including *BRCA2* (*n* = 6 patients), *MSH6* (*n* = 5 patients), *MLH1* (*n* = 4 patients) and *TP53* (*n* = 2 patient). Of note, no *PMS2* constitutional variants were found.

### Identification of fusion genes in sebaceous tumours

In addition to WES, exome capture RNA-seq was performed on tumours for the purpose of the IC analysis (see above) and for identifying fusion genes in the transcriptome and viruses and other pathogens/organisms from unmapped reads (Supplementary Discussion in **Supplementary Information**, Supplementary Data 13 and see “**Methods**”). 105 fusions were identified across all samples, including 9 that were recurrent between tumours from different patients or were found in distinct (unrelated) tumours from the same patient. Of these, 4 were inframe fusion events (Supplementary Data 14). Notably, *PAK2* fusions (*PAK2::DLG1, PAK2::LRIG1* and *DLG1::PAK2*) were identified (Supplementary Fig. 11A in **Supplementary Information** and Supplementary Data 14) with the reciprocal event *DLG1::PAK2* observed in the case with a *PAK2::DLG1* fusion. We did not identify a reciprocal event in the tumour with the *PAK2::LRIG1* fusion, which might suggest it is expressed below the level of detection or it is not generated. Importantly, our analysis suggests that *PAK2* fusions are associated with benign tumours and with sebaceous differentiation, as was recently reported in apocrine poroma^46^, with fusions found in two SMs and one SA, without specific morphological features, in our cohort (Supplementary Data 14). Previously unreported fusions (Supplementary Discussion in **Supplementary Information**) included *RCOR1::GRHL2*, an interchromosomal fusion identified in two sebaceomas from the same patient, both showing the same breakpoint (Supplementary Fig. 11B in **Supplementary Information**), suggesting a shared, possibly post-zygotic, origin with this specific patient, noted as having multiple recurrent sebaceous cysts on his back.

### Predicting the immune landscape of sebaceous tumours

Factors that restrain ST tumour growth and malignant progression likely include contributions from the immune system, as seen in melanoma and other cancers. dMMR cancers carry numerous tumour neoantigens, which are critical targets of the host anti-tumour response and correlate with the efficacy of immunotherapy. Thus, tumour neoantigens were predicting across the cohort (see “**Methods**”). Hypothetical predicted neoantigens were analysed at the gene and variant level. Across the cohort, the top three genes harbouring mutations predicted to produce neoantigens (combined class-I and class-II) were the driver genes *HRAS*, *RREB1* and *NOTCH1*, with neoantigens predicted in 28, 20 and 19 samples, respectively (Supplementary Fig. 12A in **Supplementary Information** and Supplementary Data 15). At the variant level (Supplementary Fig. 12B in **Supplementary Information** and Supplementary Data 15), *HRAS* showed recurrent predicted neoantigens (combined class-I and class-II) at p.G12S (*n* = 7), p.K117N (*n* = 4), p.A18V *(n* = 3), p.Q61K *(n* = 3), p.Q61R (*n* = 2) and p.G13R (*n* = 2; Supplementary Fig. 12B in **Supplementary Information** and Supplementary Data 15). *ACVR2A* p.K437Rfs*5 was the most recurrent variant (*n* = 11), while *NOTCH1* showed 19 distinct predicted neoantigen variants. The predicted neoantigen *RREB1* p.P1064Rfs*19 was found in three samples. Other driver genes with putative neoantigens included *ZNF750*, *MBD6*, *NOTCH2*, *PTPN14* and *TP53* (Supplementary Data 15).

Taking all four ST subtypes together or separately, predicted class-I and class-II neoantigen load, as well as hypothetical predicted immunogenicity of the neoantigens, were significantly higher in dMMR tumours compared with pMMR tumours (Supplementary Fig. 13A–H in **Supplementary Information**). However, no differences between dMMR and pMMR tumours was observed for the SC-E cohort, apart from a minor increase in class I neoantigenicity. Interestingly, the SC-E cohort (regardless of MMR status) showed similar neoantigen counts and neoantigenicity as dMMR SA and SM. Looking at the sub-groups identified by the Integrative Clustering analysis, IC3 and IC5 had the highest neoantigen tumour immunogenicity class-I, while IC4, IC8 and IC9 were among the lowest (Fig. 3D). In comparison, IC1 to IC7 were in the highest groups of neoantigen tumour immunogenicity class-II and combined, while IC9 was among the lowest part.

## Discussion

Several findings in this study may prove clinically relevant in the diagnosis of ST and LS. Use of somatic mutational profiling may help in the differential diagnosis of benign versus malignant STs, which can be challenging. For example, malignant STs were driven by chromosomal instability and typically showed biallelic *TP53* inactivation, as well as *TP53* mutations co-occurring with *ZNF750* and/or *RB1* mutations. In addition, dMMR SC-E was characterised by mutations in *NOTCH1, RREB1, and DRD5*, whereas SC-O was characterised by significant focal amplifications encompassing *GNAS/SALL4* and *LYN/PLAG1*, significant broad 13q deletions (encompassing *BRCA2* and *RB1*), and 8q amplifications (encompassing *MYC*), with amplification of the *MYC* proto-oncogene previously reported by targeted sequencing and FISH^11^. In pMMR SC-E from the genital area, high-risk HPV virotypes can be causative with a wild-type phenotype for *TP53* and *RB1* (PD45521a and PD48821a), like previously described in SC-O^12^. Lastly, both SC-E and SC-O showed significant amplification of chromosomes 1q21.3 and 20. The former amplification encompasses *HNRN* (Hornerin), which is part of the epidermal differentiation complex on 1q21. Hornerin is expressed by the ocular surface (corneal and conjunctival aspects, including meibomian and Zeis glands), the lacrimal apparatus and the skin, and has antimicrobial and protective (keratinisation) functions^47,48^, reinforcing its potential role in sebaceous carcinoma. STs, with the exception of SC-O, can be the first sentinel sign of LS, and 3 clinical criteria and 4 histopathological variables seem to be associated. These are: (i) the presence of multiple STs, (ii) STs located outside the head and neck region, and (iii) the presence of an LS-associated cancer in the clinical model; and (i) the absence of epidermal connection, (ii) delimitation of the ST by a collarette, (iii) a KA-like architecture, and (iv) the diagnosis of SM in the histopathological model, together with loss of MMR expression by immunohistochemistry. These features were found to be significantly associated with an LS diagnosis, with the clinical model being the better predictor, and should therefore raise the index of suspicion and prompt consideration of referral for germline genetic testing. Of note, while the concept of generating a classifier is valid, a comparable dataset of STs to the one described in our study does not currently exist, which makes validation of such a tool problematic and therefore premature at this stage. Further, this study highlights the importance of recording the precise location of SC-O tumours, and specifically restricting the use of the term SC-O for only those arising on the eyelid. Indeed, the sample coming from the canthus showed a different phenotype from the other SC-O, being characterised by UV mutational signatures rather than being pauci-mutated.

A subset of SC can have an aggressive behaviour leading to metastasis and/or death, and no well-established treatments are available. Typically, this subset was of pMMR status, showed grade 3 histopathological criteria (architectural growth – cytological differentiation and cytological atypia), and had co-occuring *TP53-RB1* mutations. Lastly, in aggressive SCs, screening for *KIT* amplification may be worth performing, with tyrosine kinase inhibitors being well-established for treatment of *KIT* altered tumours^49^, as *KIT* amplification was detected in a multi-metastatic SC-O in a young female (PD47219).

In conclusion, this study has demonstrated that although STs are histopathologically distinct, there are similarities in their molecular landscapes, suggesting underlying epigenetic phenomena or different cells of origin likely define different subtypes. Molecular alterations driving sebaceous tumorigenesis can divide STs into highly-mutated and pauci-mutated tumours. Highly-mutated tumours can develop in a context of (i) an inherited predisposition such as LS or *MUTYH* syndrome; (ii) an acquired alteration such as mismatch repair deficiency, UV-damage, *POLE/POLD1* syndrome, or APOBEC activation; and are enriched for mutations of the molecular subtypes A (*NOTCH1* & *RREB1*), B (*NOTCH1*), C (*RREB1*) and D (*HRAS*). In contrast, pauci-mutated tumours, including predominantly SC-O, are mostly characterised by molecular subtype E (*TP53*), with no associated endogenous or exogenous factors. In addition, cnLOH was shown to play an important role in biallelic inactivation of driver genes such as *MSH2*, *MLH1*, *NOTCH1*, *TP53* and *ZNF750*. Although rarely encountered, HPV can be associated with sebaceous carcinoma and we report cases of SC-E with this virus. Multiple tumours from the same patient were clearly shown to be independent as they differ in their mutational landscape. Lastly, *PAK2* fusions may be related to sebaceous differentiation as found in sebaceous tumours and apocrine poromas^46^. This study also highlighted molecular alterations that could be used as diagnostic tools for STs and provides potential avenues to explore for the development of novel therapeutic strategies for the rare cases of ST that are inoperable or metastatic.

## Methods

### Case selection and material extraction

Ethical approval for the use of these samples and associated data was obtained by a local committee at the institution of origin and via Research Governance at the Wellcome Sanger Institute. This study is part of a larger study that has been approved by the National Health Service Health Research Authority (Research Ethics Committee reference 21/PR/1024, IRAS project ID 304621).

The samples (SA (*n* = 102 primary tumours), SM (*n* = 92 primary tumours), SC-E (*n* = 49 primary tumours and *n* = 1 metastasis), and SC-O (*n* = 26 primary tumours, *n* = 2 recurrences and *n* = 7 metastases), SH (*n* = 6) and KA with sebaceous differentiation (*n* = 1)) were identified from eleven clinical centres across six countries. Representative haematoxylin & eosin (HE)-stained sections were independently reviewed by two specialist dermatopathologists to confirm diagnoses and identify areas for sampling (tumour +/− normal tissue). Immunohistochemistry stains were also reviewed when possible or were performed to aid diagnosis. Clinical data and follow-up were obtained from patient records in keeping with ethical approvals.

All tumour and normal tissue samples were obtained from formalin-fixed paraffin-embedded (FFPE) tissue blocks by either using a tissue microarray (TMA) coring needle or macrodissecting unstained 10-micron thick tissue sections attached to glass slides to collect specifically identified tumour and normal material following a detailed assessment of the H&E slide of each case. Genomic DNA and RNA was extracted from the tumour samples (with genomic DNA only extracted from normal samples) using the AllPrep DNA/RNA FFPE Kit (Qiagen), according to the manufacturer’s instructions.

### Sequencing and data processing

#### Whole exome

Sequencing libraries were prepared from the FFPE-extracted DNA using a NEBNext Ultra II DNA Library Prep Kit (New England Biolabs), according to the manufacturer’s instructions. Unique dual index tags were applied, and the samples were amplified by PCR using the KAPA HiFi Kit (KAPA Biosystems) for a minimum of eight cycles. The libraries were quantified using Accuclear dsDNA Quantitation Kit (Biotium), pooled (8-plex) in an equimolar fashion and hybridised overnight with the SureSelect Human All Exon V5 baits (Agilent). The multiplexed samples were paired-end sequenced using the HiSeq and NovaSeq platforms (Illumina) to generate 101 bp reads.

Sequencing reads were aligned to the GRCh38 reference genome, using BWA-MEM^50^, and PCR duplicates were marked using Biobambam2 bammarkduplicates2 (v2.0.146). Tumour-normal sample concordance as well as cross-individual contamination was assessed using Conpair v0.2^51^. Samples were excluded on the basis of quality issues, such as < 80% of the targeted regions covered with 20X coverage or more, excessively small library insert size, or cross-individual contamination > 5% in either the tumour or normal.

#### Transcriptome

The FFPE-extracted RNA samples were transcribed, and sequencing libraries prepared using the NEBNext Ultra II Directional RNA Library Prep kit (New England Biolabs) according to the manufacturer’s instructions. Unique dual index tags were applied, and the samples were amplified by PCR using the KAPA HiFi HotStart ReadyMix PCR Kit (Roche) for a minimum of 16 cycles. The libraries were quantified using Accuclear dsDNA Quantitation Kit (Biotium), pooled (8-plex) in an equimolar fashion and hybridised overnight with the SureSelect Human All Exon V5 baits (Agilent). The multiplexed samples were paired-end sequenced using the NovaSeq platform (Illumina) to generate 101 bp reads. Reads were aligned using STAR v2.5.0c15^52^ against the GRCh38 human reference genome alongside Ensembl v103 gene annotation. Expression was assessed by counting reads using HTseq v0.7.2^53^ with the appropriate stranded parameter and subsequently transformed into transcripts per million (TPM). Data quality was assessed by running RNA-SeqQC 2^54^ and looking at the total number of counts obtained per sample. We discarded any samples that reported: low expression profiling efficiency < 40%, 3prime bias < 0.3 or 3prime bias > 0.5, proportion of reads intersecting rRNAs > 2.5, read pairs counted < 20 × 10^7^, had higher number of low quality, ambiguous, alignment not unique than reads counted; or number of genes with five counts or more < 14 × 10^3^^55^.

### Analyses

#### Whole exome

##### Somatic variant calling

Somatic point mutations were identified using cgpCaVEMan (v1.15.1)^56^. SmartPhase (v1.2.1)^57^ was used to identify MNVs from SNVs called with cgpCaVEMan, while adjacent SNVs were first extracted from VCFs generated by CaVEMan. In addition, indels on the autosomes and chromosomes X and Y were identified using cgpPindel (v.3.10.0)^58^ (https://github.com/cancerit/cgpPindel). For both cgpCaVEMan and cgpPindel, tumours without a matched normal tissue sample were analysed using a BAM file containing ∼ 50x exome coverage of simulated 100-bp paired-end sequencing reads with an average 300 bp insert size generated from the GRCh38 reference genome sequence, without mismatches.

Gene models and the Variant Effect Predictor (VEP)^39^ from Ensembl v103 were used to predict the consequences of base changes and indels on proteins. The canonical transcript, as defined in Ensembl, was used to determine the variant consequence. VEP was also used to add custom annotations from the COSMIC (v97)^59^, ClinVar (update 20230121)^40^, gnomAD (v3.1.2)^60^ and dbSNP (v155)^61^ databases.

Common SNVs, defined as variants present in 1% or more of the total population in the gnomAD database or the 1000 Genomes Phase 3 dataset (as indicated by dbSNP v155), were excluded as germline variants. Pindel calls were further refined by retaining only variants with VAF ≥ 0.1 and multinucleotide variants of length 3 bp or less. Variants were retained where both the reference allele and alternative allele are ≤ 25 bp in length and both alleles are not > 10 bp, otherwise the variants were retained only if the tumour VAF > 0.25 and sequencing coverage at the variant site was ≥ 20 in both the tumour and matched normal. Finally, variants within 100 bp of exome targeted regions and not flagged by cgpCaVEMan or cgpPindel filters were taken forward for further analysis. For the generation of the oncoplots, the FLAG genes were not included as they are frequently found to bear rare, likely functional variants within the general population^62^.

##### Somatic copy number alteration

Somatic copy number alterations (SCNAs) were identified in tumours with matched normal tissue using ASCAT (v3.1.2)^63^ in all autosomes and the chromosome X. The required hg38 reference files for processing WES data (loci, allele, GC correction and replication timing correction files) were downloaded from https://github.com/VanLoo-lab/ascat/tree/master/ReferenceFiles/WES (git commit ID 29f2fad). The loci and allele files were used as input to the ascat.perpareHTS function, along with tumour and matched normal BAM files. Other parameters used were as follows: genomeVersion = hg38, minCounts = 10, min_base_qual = 20, min_map_qual = 35 and seed = 485028101 for reproducibility. A BED file containing the genomic coordinates of the exome pull-down regions sequenced was also provided, as recommended. The sex parameter was determined by the clinical data provided for each patient, and alleleCount (v.4.3.0; https://github.com/cancerit/alleleCount) was also used. The outputs of ascat.prepareHTS were used to run the ascat.correctLogR function with the reference GC and replication timing files, and the ascat.aspcf function was then run with penalty = 70 and seed = 483024451 for reproducibility. Finally, the ascat.runAscat function was run using gamma = 1 to estimate purity, ploidy and allele-specific copy number at each loci. Cases with a solution with a goodness-of-fit score < 0.9 were considered noisy and excluded from further analysis.

To find significant recurrent amplifications and deletions, the outputs from ASCAT were used to generate input files for GISTIC2 (v2.0.23)^64^, which requires segment coordinates, the number of markers and a log2-scaled copy number. For each segment obtained from segmentation from ASCAT analysis, the normalised depth log ratio was extracted, and the number of assessed loci within each segment was used as the number of markers. GISTIC2 was run with the following parameters: -refgene hg38.UCSC.add_miR.160920.refgene.mat -genegistic 1 -smallmem 1 -broad 1 -brlen 0.75 -conf 0.95 -armpeel 1 -savegene 1 -gcm extreme -v 20 -ta 0.25 -td 0.25. The hg38.UCSC.add_miR.160920.refgene.mat file is included with GISTIC2. The threshold for the residual q-value for both broad and focal amplifications and deletions was set at 0.10. To remove artefactual recurrent SCNAs, we further removed GISTIC2 wide peaks with an overlap of more than 40% with regions known to be problematic for sequencing and/or read mapping, as defined by the Genome in a Bottle Consortium benchmark union set of all difficult regions (v3.3; https://ftp-trace.ncbi.nlm.nih.gov/ReferenceSamples/giab/release/genome-stratifications/v3.3/GRCh38@all/Union/GRCh38_alldifficultregions.bed.gz). Finally, we also excluded a wide peak if the list of samples predicted to have a particular SCNA in GISTIC2 did not concur with the original ASCAT results.

##### Driver genes

To identify driver genes, we used dNdscv v0.1.0 (git commit ID 64f8443)^65^, which detects genes under positive selection in cancer. The reference gene annotations and covariates files used to run dNdScv were provided in the dNdScv package (RefCDS_human_GRCh38_GencodeV18_recommended.rda and covariates_hg19_hg38_epigenome_pcawg.rda, respectively). The outputs include p-values that were adjusted using the Benjamini & Hochberg method, and the q-value threshold was set at 0.10. A gene was selected if any of the q-values for substitutions and indels combined, substitutions only, missense mutations, nonsense mutations or indels only was below the q-value threshold. For patients with more than one tumour, variants were first merged to create a composite sample by patient prior to running dNdScv.

Hypothesising that the identification of several drivers might have been missed when testing all genes, restricted hypothesis testing (RHT) was performed considering 312 previously reported cancer driver genes^17^.

OnodriveFML (v 2.4.0)^66^ was also used to identify candidate driver genes. OncodriveFML determines whether the average functional impact score of somatic mutations on an element (in this case, a coding gene) is significantly higher than expected, OncodriveFML was run with the following parameters: genome build, hg38; sequencing, wes; signature method, complement; classifier = CANCER_TYPE; statistic method, amean; sampling, 100,000; sampling max, 1,000,000; sampling chunk, 100; sampling min obs, 10; include indels, true; indel method, max; indel max consecutive, 7. Functional impact scores for the genome were downloaded from Combined Annotation Dependent Depletion (CADD; v1.7) website (https://cadd.gs.washington.edu; https://krishna.gs.washington.edu/download/CADD/v1.7/GRCh38/whole_genome_SNVs.tsv.gz). A regions file consisting of 100 bp padded bait set regions was used. A gene was considered significantly mutated if it was mutated in at least 2 samples and had *q* < 0.1.

##### Mutually exclusive and co-occurring interactions in driver genes (DISCOVER)

DISCOVER^20^ was used to identify mutually exclusive and frequently co-occurring mutations. A binarised alteration matrix was generated for 17657 genes across the 197 primary STs with associated matched normal, where 0 and 1 indicated the absence or presence of a nonsynonymous mutation, respectively. Using the DISCOVER R package v.0.9.4, pairwise tests were performed for all genes which were altered in at least 10 STs. The tests were performed (i) with no stratification by ST subtype, (ii) with stratification by ST subtype, (iii) with stratification by MMR status and (iv) with stratification by ST subtype and MMR status. A false discovery rate (FDR) of 5% was applied to determine statistically significant mutual exclusivities and co-occurrences, focusing on the 28 driver genes called by dNdScv (Supplementary Data 6).

##### Mutational signatures

To identify somatic mutational signatures for single base substitutions, doublet base substitutions, and indels, somatic mutations from tumours with matched normal tissue were analysed using SigProfilerExtractor (v1.1.21)^67^. SigProfilerExtractor performs de novo extraction of mutational signatures and assigns known signatures to samples by refitting known COSMIC signatures to the extracted signatures using SigProfilerAssignment^68^. SigProfilerExtracter was run in exome mode using GRCh38 as the reference and opportunity genome, 500 replicates for non-negative matrix factorisation (NMF) with 1 to 10 signatures. The solution implemented for downstream analysis was the optimal solution provided by SigProfillerExtractor (referred to as the suggested solution). Results from signature decomposition and assignment were taken forward if the cosine similarity between the de novo extracted signatures and signatures reconstructed from assigned COSMIC signatures was ≥ 0.9. Similarly, on a per-sample basis, signature analysis was considered reliable if the cosine similarity between the original mutational spectrum and reconstructed spectrum was ≥ 0.9 and there were at least 100 mutations per sample, for each signature type (SBS, DBS, ID).

##### Germline variant calling

Reads aligned using BWA-MEM^50^ against the GRCh38 reference, and PCR duplicates were marked using Picard’s MarkDuplicates v2.25.4 (https://broadinstitute.github.io/picard/). Germline variants were identified from whole- exome data from normal samples using GATK v4.2.6.1^69^ following GATK Best Practices^70^ for cohort calling of SNP and short INDELs. Variants were first called using GATK’s HaplotypeCaller in -ERC GVCF mode with parameters -G StandardAnnotation, -G StandardHCAnnotation, -G AS_StandardAnnotation. Followed by the creation of a genomicsdb database using GenomicsDBImport. Finally, joint genotyping was performed using GenotypeGVCFs. We separated SNP and INDELs using Picard’s SelectVariants v2.27.1. Subsequently, we used Picard’s VariantFiltration v2.27.1 to hard filter variants. For SNPs we filtered out variants: outside 100 bp upstream or downstream from the baitset regions, QUAL < 30.0, SOR > 3.0, FS > 60, MQ < 40, MQRankSum < − 12.5 and ReadPosRankSum < − 8.0. For indels, we filtered out variants: outside 100 bp up and downstream from the baitset coordinates used, QD < 2.0, QUAL < 30.0, FS > 200.0 and ReadPosRankSum < − 20.0. High-quality variants functional consequences were annotated using Ensembl Variant Effect Predictor (VEP) v103 using the same parameters, and additional annotations from ClinVar (Updated 2023.11), COSMIC, and gnomAD were added, as the somatic variants. Only indels ≤ 10 bp were kept. To identify variants present within known germline cancer predisposition genes, we looked for variants present within the set of genes reported to be used by England’s National Health Service (NHS) Cancer National Genomic Test Directory v7.2 June 2023 [https://www.england.nhs.uk/wp-content/uploads/2018/08/Cancer-national-genomic-test-directory-version-7.2-June-2023.xlsx] to diagnose cancer predisposition in patients. Only variants with predicted moderate or High Impact over the encoded protein were reported. Variants found in *POT1* were removed, as after manual inspection, they appeared to be artefactual.

##### Ancestry analysis

We projected the germline single-nucleotide polymorphisms from the whole-exome sequencing normal samples into the principal component space of the 1000 Genomes (1000 G) project^71^ using the package AKT v0.3.3^72^. Reported country of birth was aligned with projected superpopulation by visual inspection of 1000 G individuals alongside study individuals in the first four principal components, this confirmed that most individuals were either of European or East Asian ancestry.

##### MMR status

MMR status was defined based on 3 criteria, such as MMR expression (MLH1, MSH2, MSH6 and PMS2) by immunohistochemistry, indels rate and dMMR mutational signatures. Tumours were called dMMR when presenting loss of expression of at least one MMR, high indels rate (>10/Mb) and/or at least one dMMR signature (SBS and/or ID). In contrast, tumours were named pMMR when showing retained expression of MMR, low indels rate (<10/Mb) and/or no dMMR signatures (SBS and/or ID). Of note, tumours with no matched normal presented a higher mutational burden/indels rate due to the lack of accurate SNP filtering.

However, when statistically testing the best predictor for dMMR detection (i.e., indel rate versus tumour mutational burden; TMB (including SNVs, MNVs and indels), only confirmed dMMR and pMMR tumours were selected, in which IHC and somatic/germline variant calling where available. dMMR STs were retained when showing a MMR loss of expression, in addition to a biallelic mutation of an MMR genes.

#### Transcriptome

##### Fusion genes

Gene fusion identification was performed using STAR-Fusion v1.10.1^73^, with STAR v2.78a and the Trinity Cancer Transcriptome Analysis Toolkit (CTAT) genome library StarFv1.10 for GRCh38 using GENCODEv37(Ensembl v103) gene annotation. To assess the coding effect of the fusions and to annotate their presence in previous databases and validate them in silico, we used FusionInspector v2.6.0 by running STAR-Fusion with the parameters —FusionInspector validate —examine_coding_effect —denovo_reconstructFusion. Subsequently, we filtered out gene fusions: fusions with the total number of Junction read counts plus Spanning frag counts < 5, FFPM < 0.1, not a predicted coding effect, annotated as previously reported in normal tissues of the Genotype-Tissue Expression (GTEx) project or neighbours. Collation of results, filtering, summaries per cohort and plotting was performed using custom scripts. Fusion gene structures were plotted using the Chimeraviz v1.24.0^74^ package in R. In cases where data for multiple samples for the same patient was available, we used only a single sample to report the observed frequency of fused gene pairs within the cohorts.

##### Cell-fraction estimation of the tumour microenvironment (TME)

The Immune Estimation module of TIMER2.0 (Tumour Immune Estimation Resource version 2)^75^ for immune infiltration estimation was run on the tumour samples. TPM-normalised, gene by sample, expression matrices were provided as input to the TIMER2.0 shiny web server (http://timer.cistrome.org/), selecting AUTO as the cancer type.

Using EPIC output from TIMER2, cell-fraction estimation of nine cell types relevant to the TME of skin malignancies were obtained: B cells, cancer associated fibroblasts (CAFs), CD4 + T cells, CD8 + T cells, endothelial cells, tumour cells, and macrophages. These data were used alongside other data for the integrative clustering (described below).

#### Whole exome and transcriptome

##### Integrative clustering

*Pre-processing of mutation data*: SNVs and indels were collated per gene into a binary matrix, with 1 s if the gene was mutated in a sample and 0 if the gene was wild-type. The selection of features was based on three criteria: first, genes which were at least mutated in 50 samples were selected. Second, those genes with mutations in at least 15 samples were tested (using a *t* test) for association between the presence of mutation and total mutational burden in the tumour. Those genes with a p-value larger than 0.01 after FDR (false discovery rate) correction were selected, as those corresponded to genes where the presence of a mutation is not associated with high mutational burden in the tumour. Finally, based on biological knowledge, cancer genes such as *TP53*, *MSH2*, *MSH6*, *MLH1*, *PMS2* were also included (if not selected by previous rules), and others like *TTN*, *MUC6* and *OBSCN* were excluded (these genes are FLAGS^76^, i.e., these are genes often non-pathogenic and passengers, but are frequently mutated in most of the public exome studies). In total, 84 genes were selected.

*Pre-processing of RNA-SEQ data*: First, transcripts were mapped to gene symbols. In cases where more than one transcript corresponded to the same gene, the transcript with the higher interquartile range (IQR) was selected. Then, the RNA-SEQ read count matrix was converted to transcripts per million kilobases (TPMs) and log transformed (log(1 + TPM)). In addition, the voom transformation^77^ was applied to attain a more suitable distribution for linear modelling. Next, the IQR of each gene was computed, and the 1000 genes that showed the most variability in expression were selected for clustering. We also added to this list the genes selected from the mutation list described above to account for cis-effects, ending up with 1082 genes. This number provided a good balance between high variance in the expression and a reasonable number of features for the computational performance of the Integrative Clustering algorithm. Finally, each gene was standardised to a z-score.

*Sample selection*: Only one sample replicate per tumour was chosen. Tumours with a diagnosis of SA, SM, SC-E and SC-O were selected, for a total of 170 tumours.

#### Integrative cluster algorithm

MOFA^78^ was used to fit a regularised latent variable model-based clustering. This model assumes a series of latent variables to represent *k* different molecular drivers that predict the observed combination of genomic and transcriptomic values in the tumours. These variables represent continuous activation levels of the different driver processes. The mutation data, represented with a binomial distribution and the expression data, represented with a Gaussian distribution, are connected to the latent driver processes via a parametric joint model. Regularisation is incorporated in the model to reduce model complexity and perform automatic feature selection.

We used the default number of factors (15), studied the robustness of the models fitting 10 runs and comparing the outputs, and selected the best model using the Evidence Lower Bound (ELBO) criteria. We observed that only the first 10 factors explained at least 1% of the variability, and we decided to cluster the tumours based on those first 10 latent variables.

We performed hierarchical clustering using the Ward method on the Euclidian distance matrix and selected the best partition using the silhouette index criteria, composed of 9 clusters. We compared alternative solutions and found that this solution provided also the most meaningful structure.

To interpret the most common features of each group, we compared the frequency of mutations in each cluster and conducted differential expression between the tumours in that cluster and the rest using limma^79^. Finally, we conducted gene ontology enrichment analysis of those differentially expressed genes using the package *clusterprofiler*^80^, correcting the p-values with FDR and selecting those pathways enriched with an FDR < 0.05, either over or underrepresented.

##### Neoantigen prediction

Class-I and -II haplotyping and neoantigen prediction were performed using the nextNEOpi nextflow pipeline^81^. Both DNA and RNA were used for predictions where available. Class-I and -II haplotypes for each sample were ascertained using Optitype^82^ and HLA-HD^83^, respectively. Variant calling was performed using Mutect2^84^, Varscan2^85^, Manta^86^ and Strelka2^87^. Variants called by Mutect2 and at least one other caller were selected as candidate neoepitopes. Clonality of neoantigens were determined by ASCAT^87^ and Sequenza^88^. Further assessment of peptide-HLA binding was performed by pVACseq from the pVACtools suite^89,90^. Finally, neoantigenicity was evaluated by a Cauchy-Schwarz index of Neoantigens (CSiN)^91^.

##### Pathogen sequence analysis

Screening for pathogens used Kraken2^92^ v2.1.2. Unfiltered paired-end whole exome and transcriptome sequencing FASTQ files were supplied as input, which was run with a 16GB-capped reference database containing RefSeq sequences from archaea, bacteria, viruses, plasmids, humans, UniVec_Core, protozoa, fungi, and plants [https://benlangmead.github.io/aws-indexes/k2] (version of March 2023). The following Kraken2 settings were used to generate i) report-style outputs: *—paired —gzip-compressed —use-names —confidence 0.1 —db path/to/DB —report path/to/report —report-minimiser-data —output /dev/null path/to/fastq1 path/to/fastq2*; ii) MPA-style outputs: *kraken2 —paired —gzip-compressed —use-names —confidence 0.1 —db path/to/DB —report path/to/report —use-mpa-style —output /dev/null path/to/fastq1 path/to/fastq2*. A confidence score threshold (*—*confidence) of 0.1 was applied to compensate the risk of false positives that is inherent to the method [https://github.com/DerrickWood/kraken2/blob/master/docs/MANUAL.markdown]. Using Kraken2 results, the proportion of minimisers was calculated from Kraken2’s reference database found in each sample for each taxon according to:

1 $${Proportion}=\frac{{Number\; of\; distinct\; minimisers\; in\; input\; read\; data}}{{Total\; clade\; level\; minimisers}\,}$$

To assess the significance of the results, a normal distribution was used to calculate the right-tailed probability of finding at least *m* minimisers, out of the total *M* minimisers available for a taxon, in *n* reads of a sample, considering the total *N* reads evaluated by Kraken2 in that sample. The Benjamini-Hochberg method^93^ was performed to correct for multiple comparisons. Results whose adjusted *p-*value was smaller than 0.05 were considered statistically significant.

##### Impact of alterations in pathway activations

Hippo and Yap1 pathway activations were computed based on the REACTOME definitions downloaded from the Molecular Signatures Database^94^ using Gene Set Enrichment Analysis with the R package gsva^95^. Next, we identified potential molecular aberrations that might have an effect on the inactivation of these pathways^96^. Each mutation was classified as follows: for oncogenes, missense variants, splice acceptor variants and splice donor variants were classified as activating mutations. For tumour suppressor genes, frameshift variants, start-lost, stop-gained and stop-lost variants were classified as truncating mutations. We also computed loss of heterozygosity using the copy number data. In order to test the association of the presence of these mutations with changes in the activation levels of the pathway, we performed a linear model and conducted a *t* test on the parameters of the model.

##### *MLH1* promoter hypermethylation testing

Analysis performed using tumour FFPE tissue derived DNA, which was bisulphite converted using the EpiTect Fast Bisulfit Kit. The methylation status of the tumour tissue was assessed by methylation sensitive high resolution melting analysis (MS-HRM), using MethylDetect MLH1 primer kits and EpiTech HRM PCR master mix on a Rotor-Gene Q 5Plex HRM platform. The MethylDetect MLH1 kit contains primers that amplify a region covering 5 CpG sites in the 5’ UTR region of the *MLH1* gene (NM_000249.3), along with high-, low- and non-methylated control materials. MS-HRM data analysis was conducted using the Rotor-Gene Q Software v2.3.4 (build 3).

### Ethics approval

Ethical approval was obtained from the different Institutes contributing to this project. Genetic analysis was further approved by the Sanger Human Materials and Data Management Committee.

### Reporting summary

Further information on research design is available in the Nature Portfolio Reporting Summary linked to this article.

## Supplementary information

Supplementary Information

Transparent Peer Review file

Reporting Summary

Description of Additional Supplementary Files

Supplementary Data 1

Supplementary Data 2

Supplementary Data 3

Supplementary Data 4

Supplementary Data 5

Supplementary Data 6

Supplementary Data 7

Supplementary Data 8

Supplementary Data 9

Supplementary Data 10

Supplementary Data 11

Supplementary Data 12

Supplementary Data 13

Supplementary Data 14

Supplementary Data 15

## Source data

Source Data

## Data Availability

Sequencing data are available from the European Genome-Phenome Archive (EGA) under the following dataset accessions: EGAS00001003553 (DNA) and EGAS00001007803 (RNA). Access requests should follow the Sanger Institute data sharing policy (https://www.sanger.ac.uk/about/research-policies/open-access-science/). Please contact datasharing@sanger.ac.uk and/or https://www.sanger.ac.uk/about/edam2-guide/. Immediate access is available upon completion of the necessary data access and legal documentation. All datasets are available indefinitely. Data are freely accessible to researchers who agree to the EGA guidelines on patient anonymity and data security, in line with the patient consent provided. The processed data generated in this study (e.g., variant calls) are available in the Supplementary Information and Source Data files. Source data are provided in this paper.

## Code availability

Scripts are available at https://github.com/team113sanger/NatCommun_Ferreira2025.
