# Supplementary material for: The molecular cartography of malignant and benign sebaceous tumours
Source: Nat Commun. 2025 Dec 19;17:14. doi: 10.1038/s41467-025-66584-0 (PMC12764876; doi:10.1038/s41467-025-66584-0)
Supplement: Supplementary file 1 — Supplementary Information [file 41467_2025_66584_MOESM1_ESM.pdf]

SUPPLEMENTARY FIGURES

Supplementary Figure 1

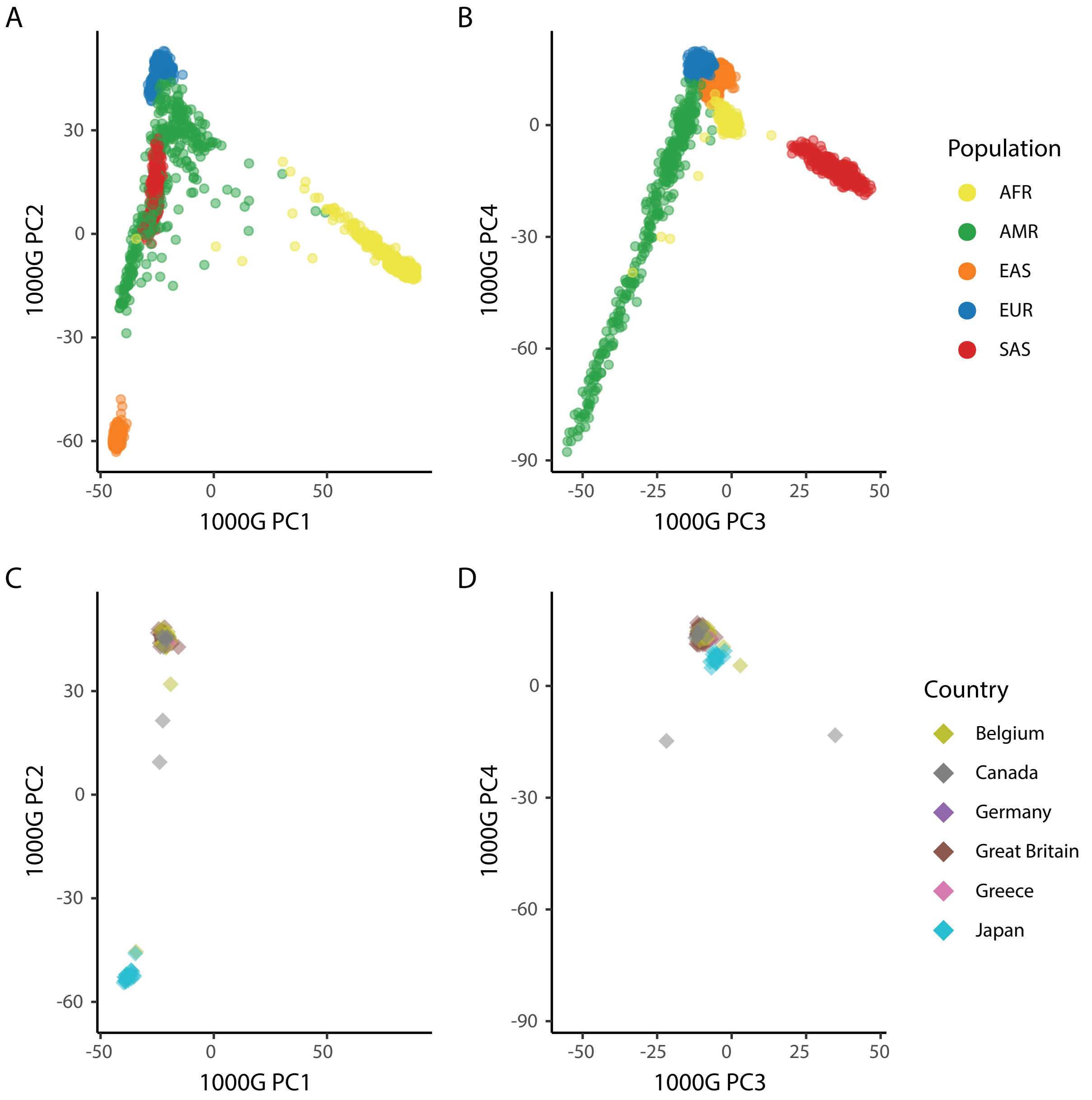

Supplementary Figure 1 – Ancestry analysis. (A-B) The first four eigenvectors from principal component analysis on the 2504 individuals in the final phase of the 1000 Genomes Project. Individuals (*n* = 2504) are coloured by the population the sample was derived from: Africa (AFR), East Asia (EAS), Europe (EUR), South Asia (SAS), and the Americas (AMR). (C-D) Individuals from our study (*n* = 166) projected into the first four eigenvectors of 1000G principal component space, coloured according to their country of birth.

Supplementary Figure 2

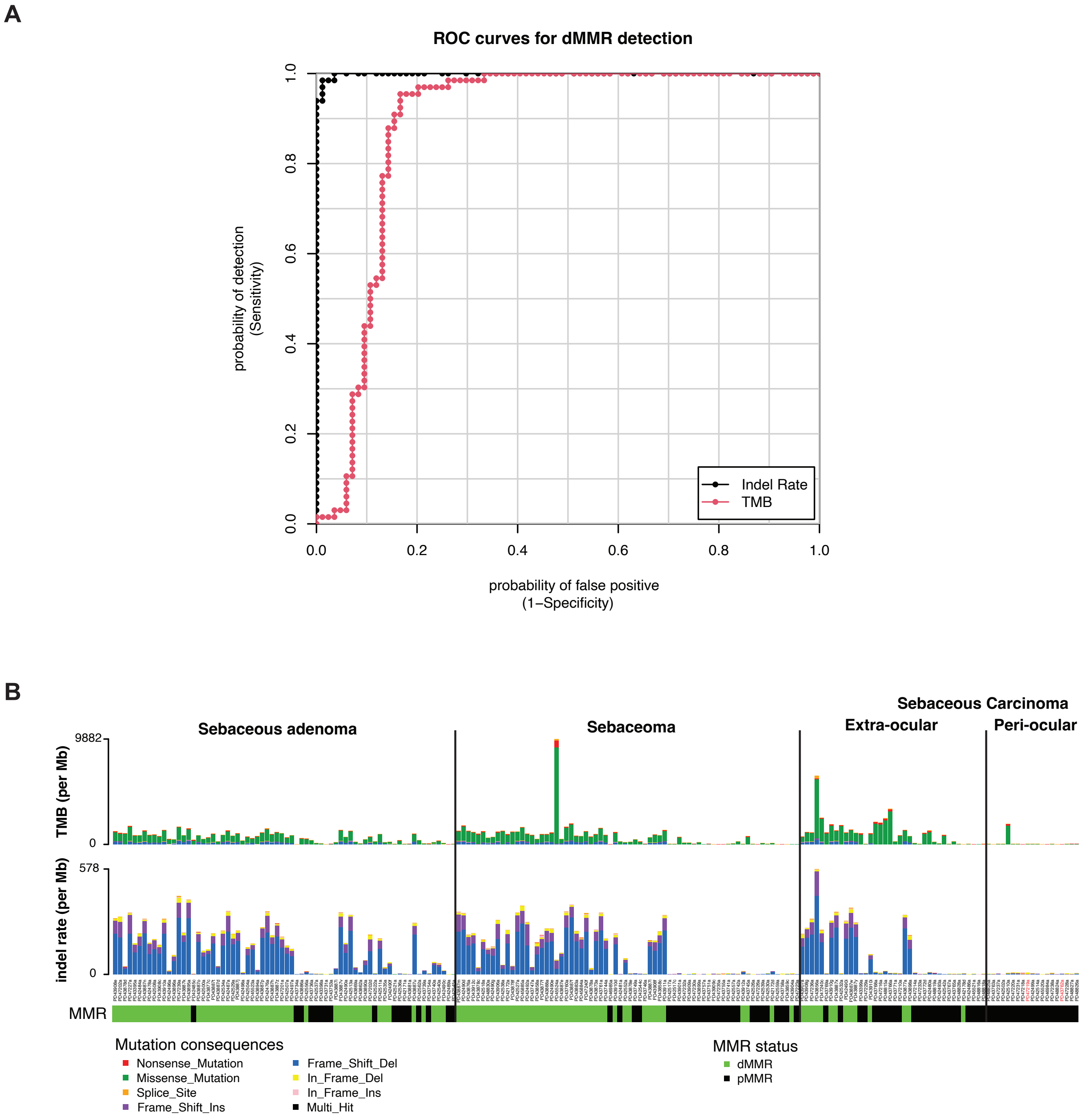

Supplementary Figure 2. (A) **Receiver Operating Characteristic (ROC) curve for dMMR detection** - Using logistic regression for both indel rate and TMB, and computing the ROC/area under the curve (AUC) using leave-one-out crossvalidation to define the best predictor for MMR status (pMMR versus dMMR), the AUC showed that indel rate (AUC = 0.999) is a better predictor than TMB (AUC = 0.887). (B) **Tumour mutation burden** - Comparison of the mutational burden when measured by the TMB rate (including SNVs, MNVs and indels) versus the indel rates, across the 4 tumour subtypes (*n* = 197 samples).

Supplementary Figure 3

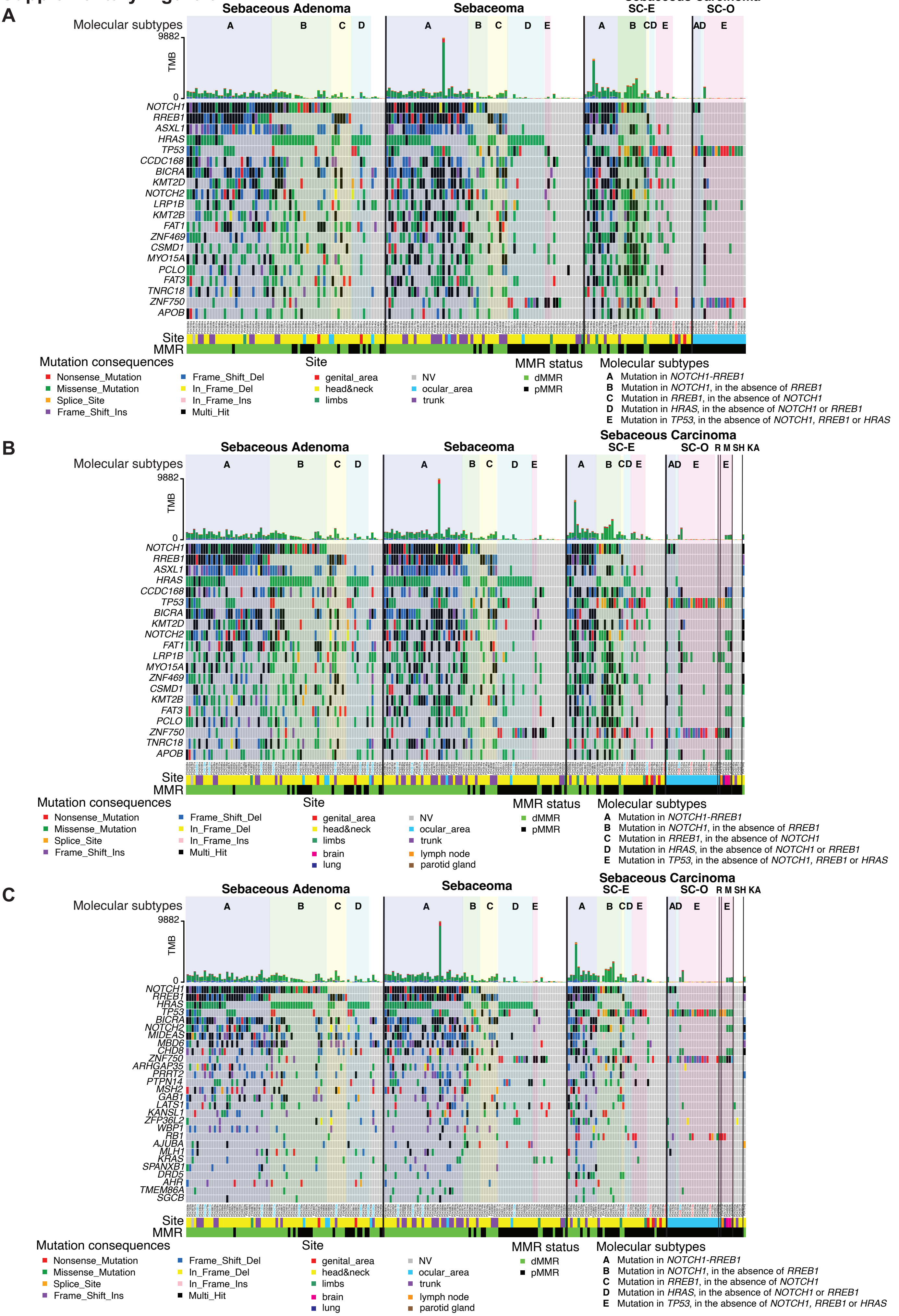

Supplementary Figure 3 – Somatic mutational landscape of sebaceous tumours, sebaceous hyperplasia and keratoacanthoma-like squamous cell carcinoma. (A) Oncoplot showing the somatic mutations in the top 20 most recurrently mutated genes of the sebaceous tumour (ST) cohort (n = 197 samples; tumour samples with matched normal samples only). (B) Oncoplot showing the somatic mutations in the driver genes of the ST cohort (n = 226 samples; all tumour samples regardless of whether matched normal was available), SC-O recurrence (R) sample (n = 1), SC-O metastasis (M) samples (n = 5), sebaceous hyperplasia SH samples (n = 4), and keratoacanthoma (KA) sample (n = 1). This includes the 25 driver genes identified when taking all four tumour subtypes together, and three additional driver genes when considering only specific tumour subtypes. (C) Oncoplot showing the somatic mutations in the top 20 most recurrently mutated genes of the ST cohort (n = 226 samples; all tumour samples regardless of whether matched normal was available), SC-O recurrence sample (n=1), SC-O metastasis samples (n = 5), SH samples (n = 4), and KA sample (n = 1). Site is the body location of the tumour. The PD number is the DNA sample identifier, with those in red font being from patients that developed metastases and those in blue font are the tumour samples that did not have a matched normal sample available. Abbreviations: MMR, mismatch repair; dMMR, deficient in MMR; pMMR, proficient in MMR; NV, not available; TMB, tumour mutational burden (in mutations/MB, including SNVs, MNVs and indels).

Supplementary Figure 4

A

Variant type	Mutations with >10x coverage in RNAseq	Total mutations observed in RNAseq	Mutations found in RNAseq	Variants with ≤ 10x coverage in RNAseq
SNV	676	667	98.7%	202
DNV	15	15	100%	5
DEL	414	407	98.3%	104
INS	128	133	96.2%	55

B

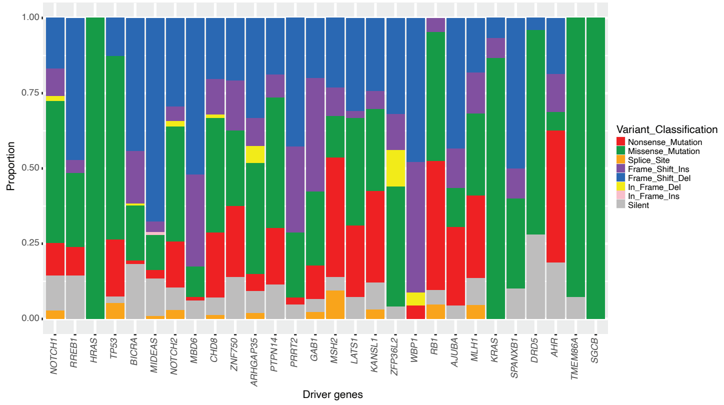

C

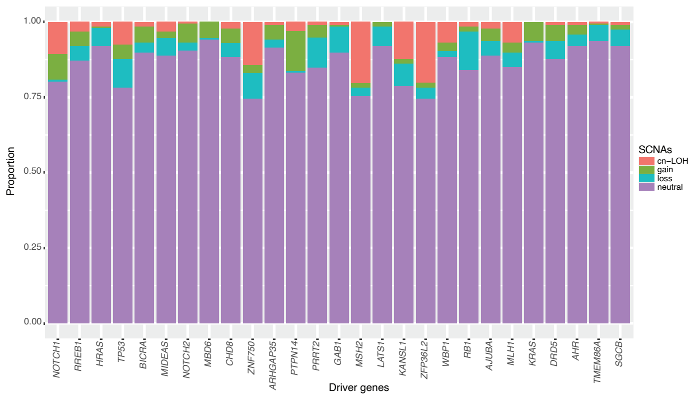

D

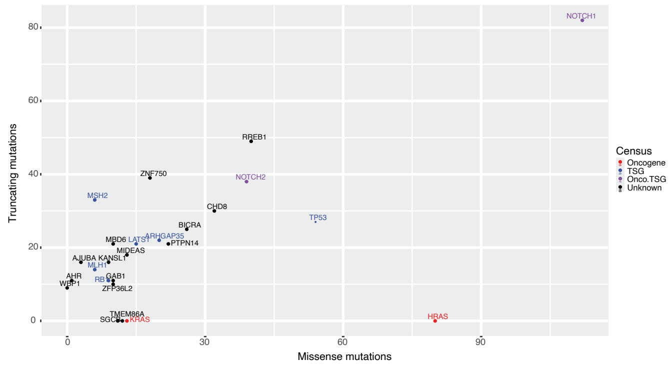

E

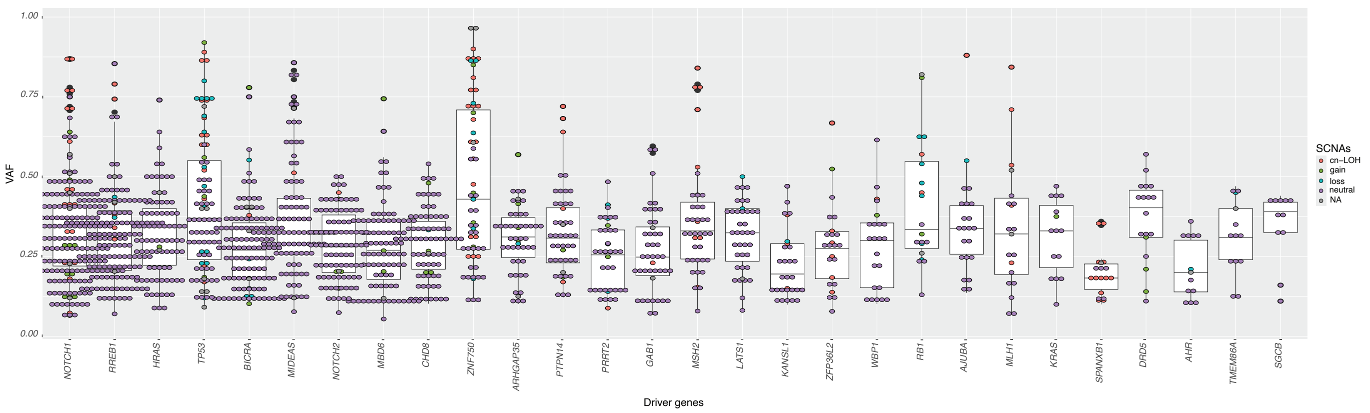

F

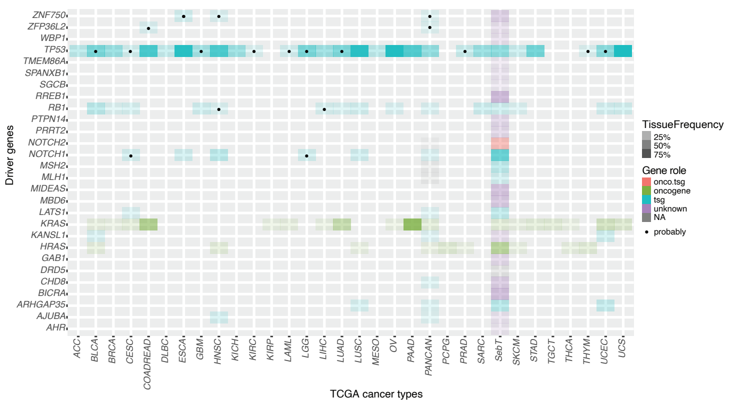

Supplementary Figure 4 – Driver genes and associated changes. (A) Table showing the proportion of somatic mutations also present in RNAseq. (B) Barplot showing the proportion of the different variant types across driver genes. (C) Barplot showing the proportion of the somatic copy number alterations (SCNAs) across autosomal driver genes. (D) Scatter plot showing missense versus truncating mutation profiles across the driver genes. (E) Barplot showing the distribution of the variant allele frequency (VAF) across the driver genes. Each dot is a tumour. Of note, *SPANXB1* is located on the X chromosome which is likely reflective of the low VAF we observe. (F) Tileplot showing the alteration of the driver genes across sebaceous tumours and pan-cancer analyses. Genes are from Bailey MH et al.[1]. The gene role is taken as defined by Bailey MH et al., in [1] and COSMIC Cancer Gene Census database for TCGA cancers and sebaceous tumours, respectively. Abbreviations: NA, not defined; onco, oncogene; onco.tsg, oncogene and tumour suppressor genes; tsg, tumour suppressor gene.

Supplementary Figure 5

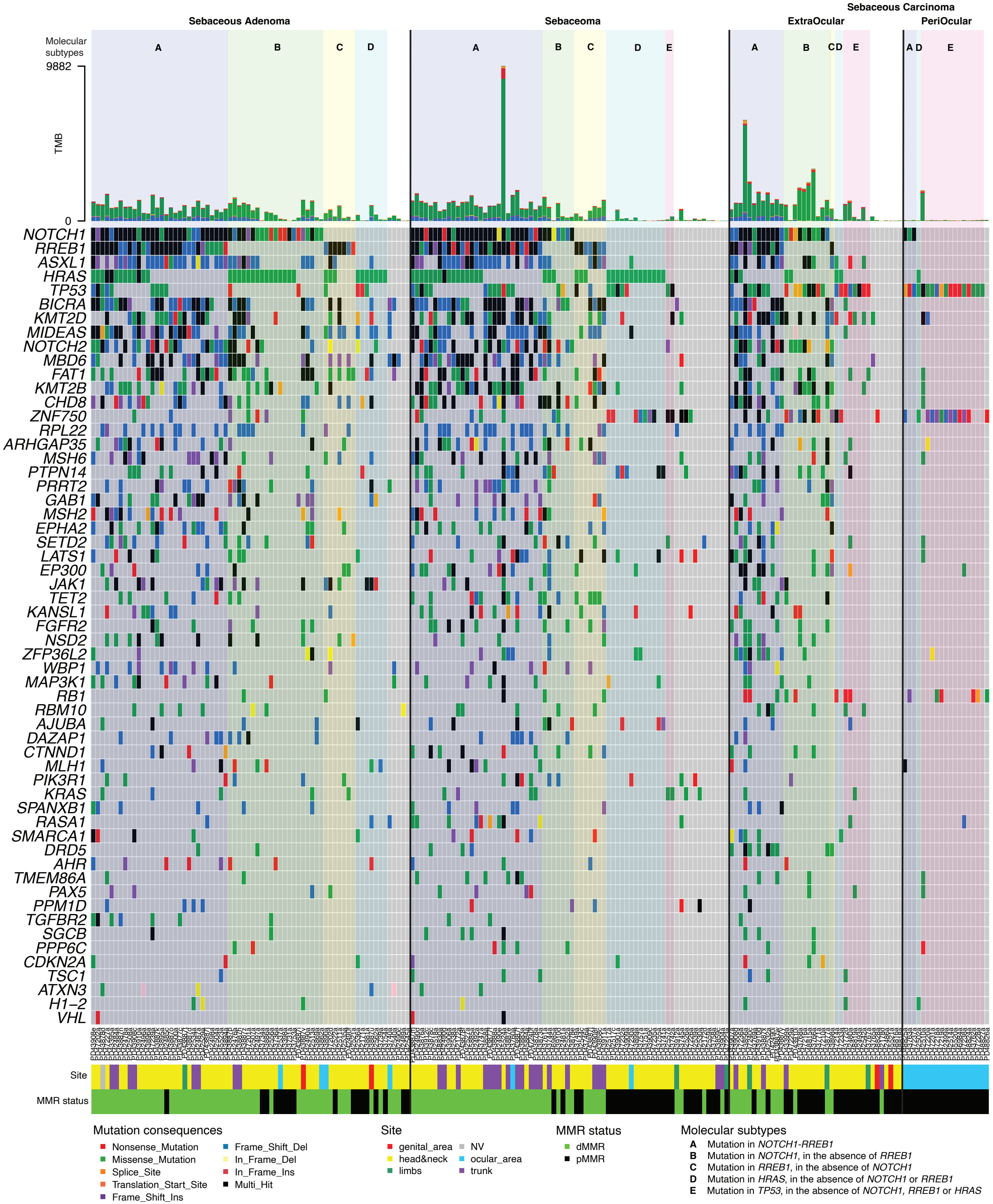

Supplementary Figure 5 – The somatic mutational landscape (driver genes post restricted hypothesis testing (RHT)) of sebaceous tumours across tumour subtypes. Oncoplot showing somatic mutations in the driver genes after RHT (ranking based on the percentage of samples altered (decreasing order)) of sebaceous tumours (STs; n = 197 samples; tumour samples with matched normal samples only). This includes the 44 driver genes identified by dNdScv when taking all four ST subtypes together, and performing RHT. Patterns of mutation of the top 4 driver genes (*NOTCH1*, *RREB1*, *TP53* and *HRAS*) allows division of the STs into five molecular subtypes (A-E) across the four ST subtypes. Site is the body location of the tumour. The PD number is the DNA sample identifier, with those in red font being from patients that developed metastases. TMB is in mutations/Mb, including SNVs, MNVs and indels). Abbreviations: NV, not available.

Supplementary Figure 6

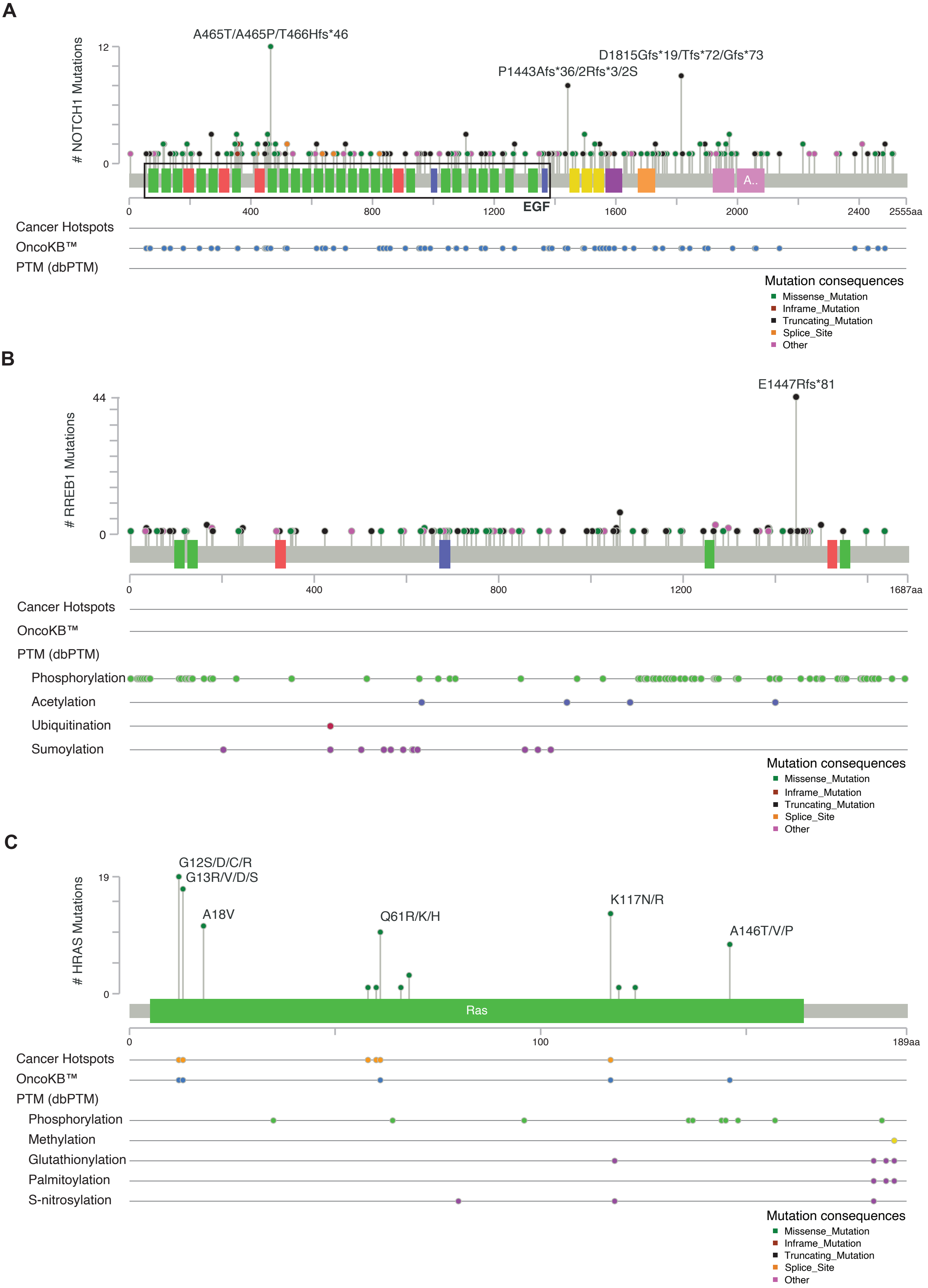

Supplementary Figure 6 – Lollipop plots of key driver genes. (A) *NOTCH1*, (B) *RREB1* and (C) *HRAS*. Abbreviations: EGF: extracellular epidermal growth factor domain.

Supplementary Figure 7

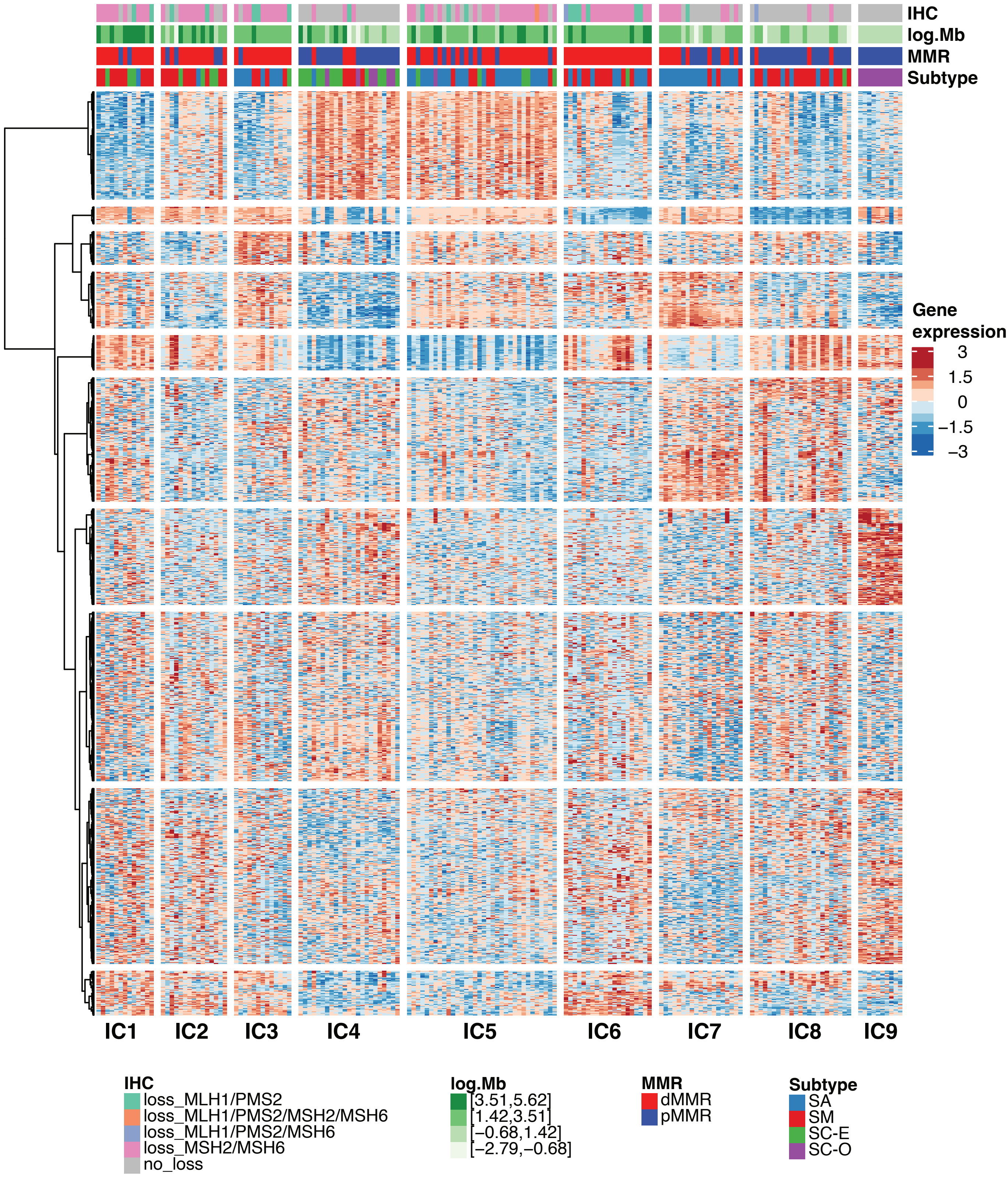

Supplementary Figure 7 – Integrative clustering. Based on somatic variants (**Figure 3A**) and expression data (A), MOFA[2] was used to fit a regularized latent variable model-based clustering to the data. Nine integrative clusters were selected as shown, associated with clinical details (immunohistochemistry (IHC) status, tumour mutational burden (log_Mb), MMR status, and ST tumour subtypes (*n* = 170)). Genes are shown on the Y-axis. Abbreviations: IHC, immunohistochemistry; MMR, mismatch repair; dMMR, deficient in MMR; pMMR, proficient in MMR; SA, sebaceous adenoma; SM, sebaceoma; SC-E, extra-ocular sebaceous carcinoma; SC-O, peri-ocular sebaceous carcinoma; log.Mb, tumour mutational burden. Gene expression is in log10(TPM) with the colour gradient going from high (in red) to low (dark blue) expression.

Supplementary Figure 8

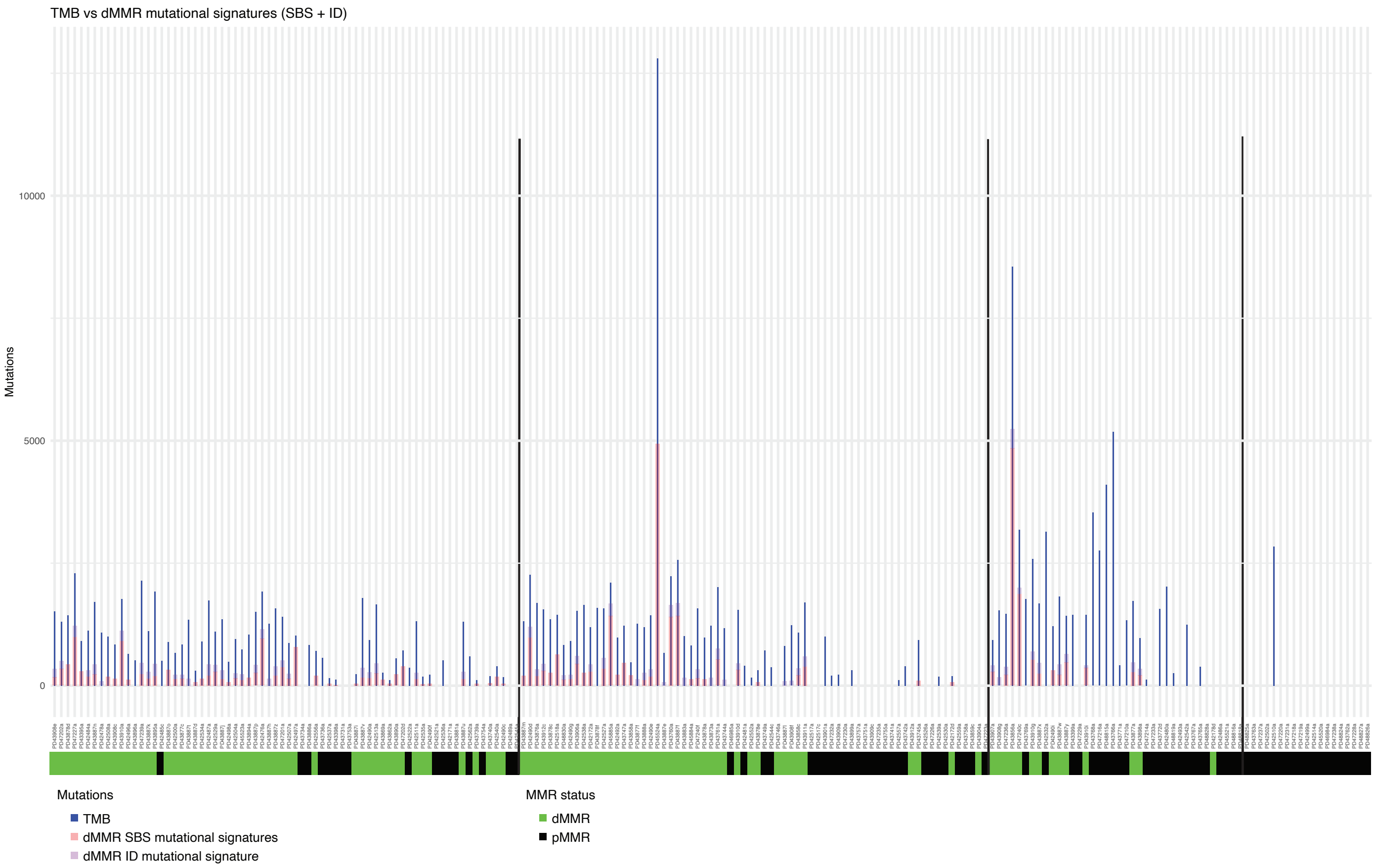

Supplementary Figure 8 – Tumour mutational burden versus mutational signature load (SBS and ID).

Supplementary Figure 9

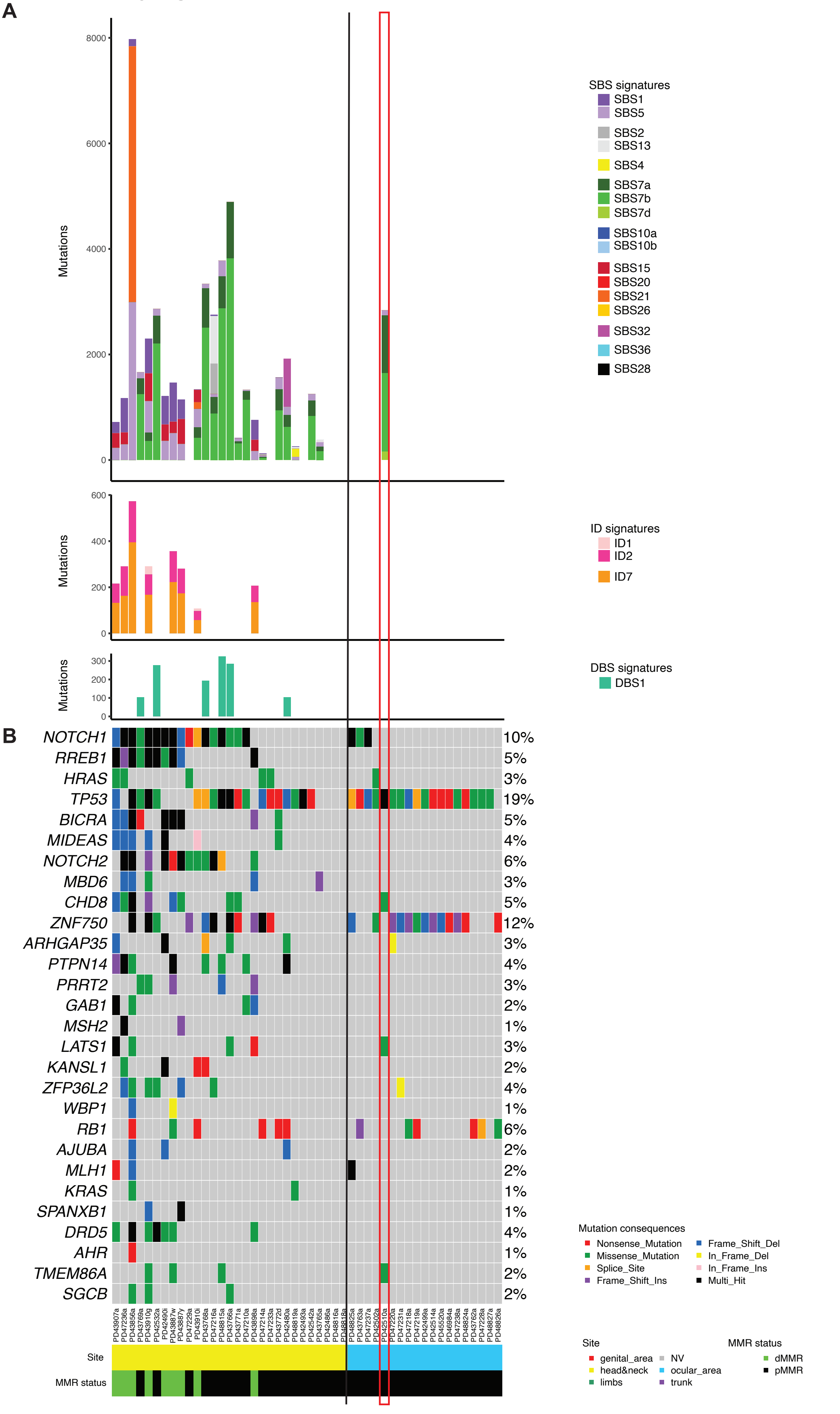

Supplementary Figure 9 – SC-E versus SC-O. Comparison of SC-E (*n* = 29) versus SC-O (*n* = 19) across mutational signatures (A) and somatic mutations of driver genes (B).

Supplementary Figure 10

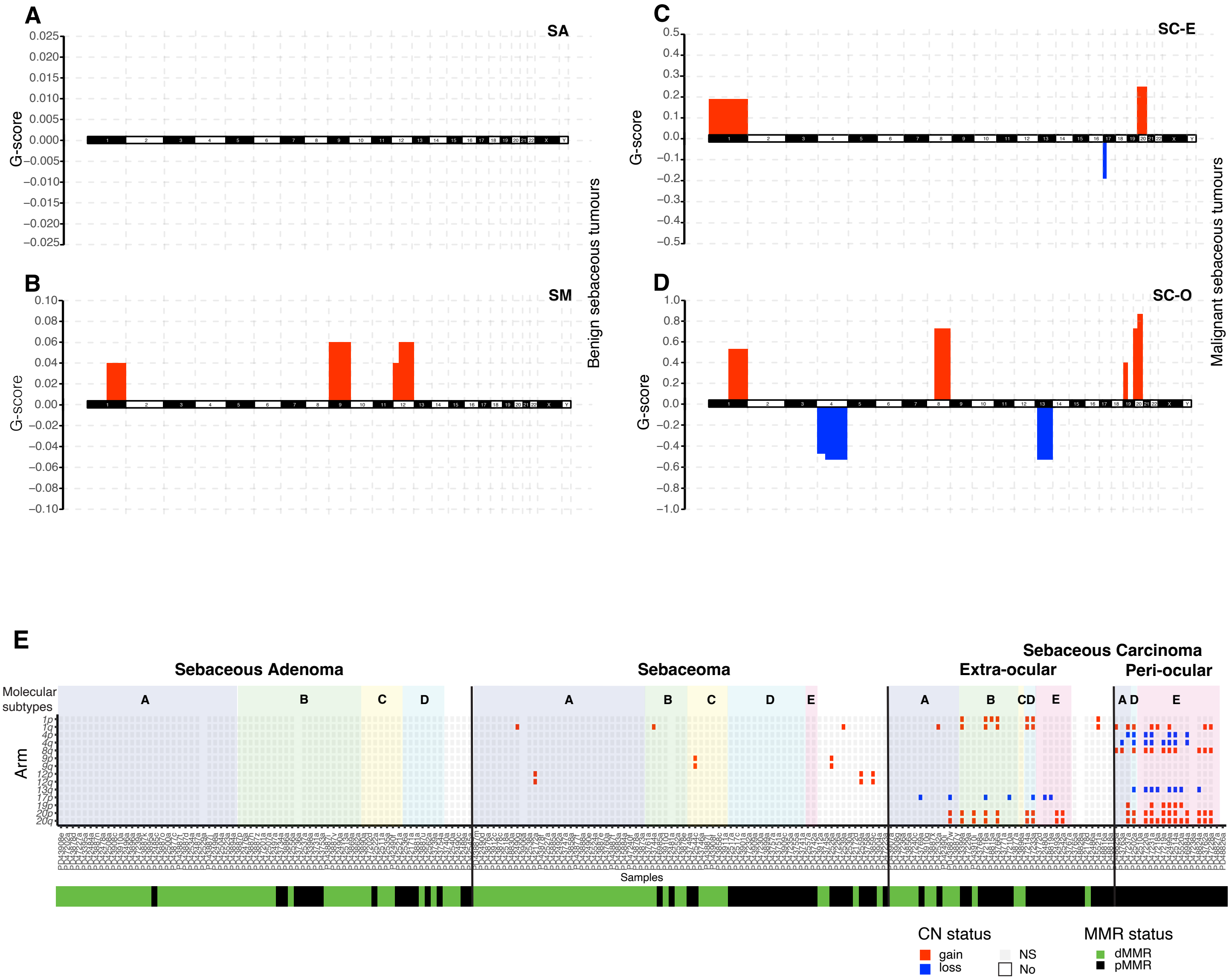

Supplementary Figure 10 – Significant broad somatic copy number alterations in sebaceous tumours. Representation of the GISTIC2.0-identified by ST subtype significant broad peak regions (≥ half a chromosome arm) in the (A) sebaceous adenomas, (B) sebaceomas, (C) extra-ocular sebaceous carcinomas and (D) peri-ocular sebaceous carcinomas. Chromosomal location of the broad peak indicated by the numbers on the black and white horizontal bar, with amplifications (gains) shown in red (positive frequency values) and deletions (losses) shown in blue (negative frequency values). (E) Oncoplot showing the individual tumour samples within the amplification and deletion broad regions shown in A-D. Samples with no significant gains or losses are indicated by NS. Five samples were not included in the GISTIC2.0 analysis (see **Methods**), so no data is shown. Abbreviations: MMR, mismatch repair; dMMR, deficient in MMR; No, not analysed; NS, not significant; pMMR, proficient in MMR; SA, sebaceous adenoma; SM, sebaceoma; SC-E, extra-ocular sebaceous carcinoma; SC-O, peri-ocular sebaceous carcinoma.

Note: *broad peaks were considered as significant when reaching the 3 criteria: 1. residual q-value < 0.1, 2. concordance with ASCAT CN calls ≥ 0.75, 3. gain with log ratio > 0.25 or loss with log ratio < -0.25 (**Supplementary Table 11**); **there were no significant results for sebaceous adenomas.

Supplementary Figure 11

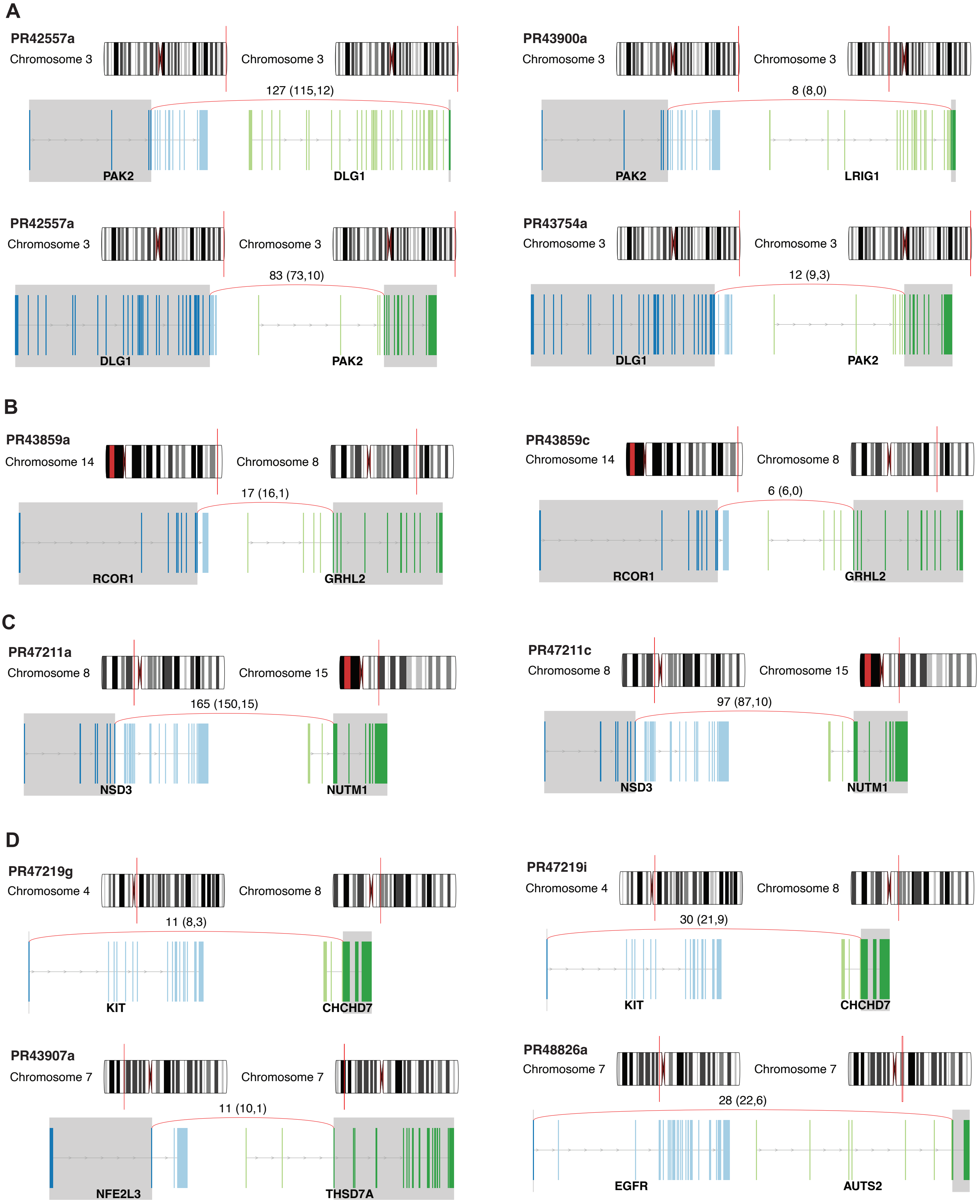

Supplementary Figure 11 – Fusion genes identified in sebaceous tumours.

Supplementary Figure 12

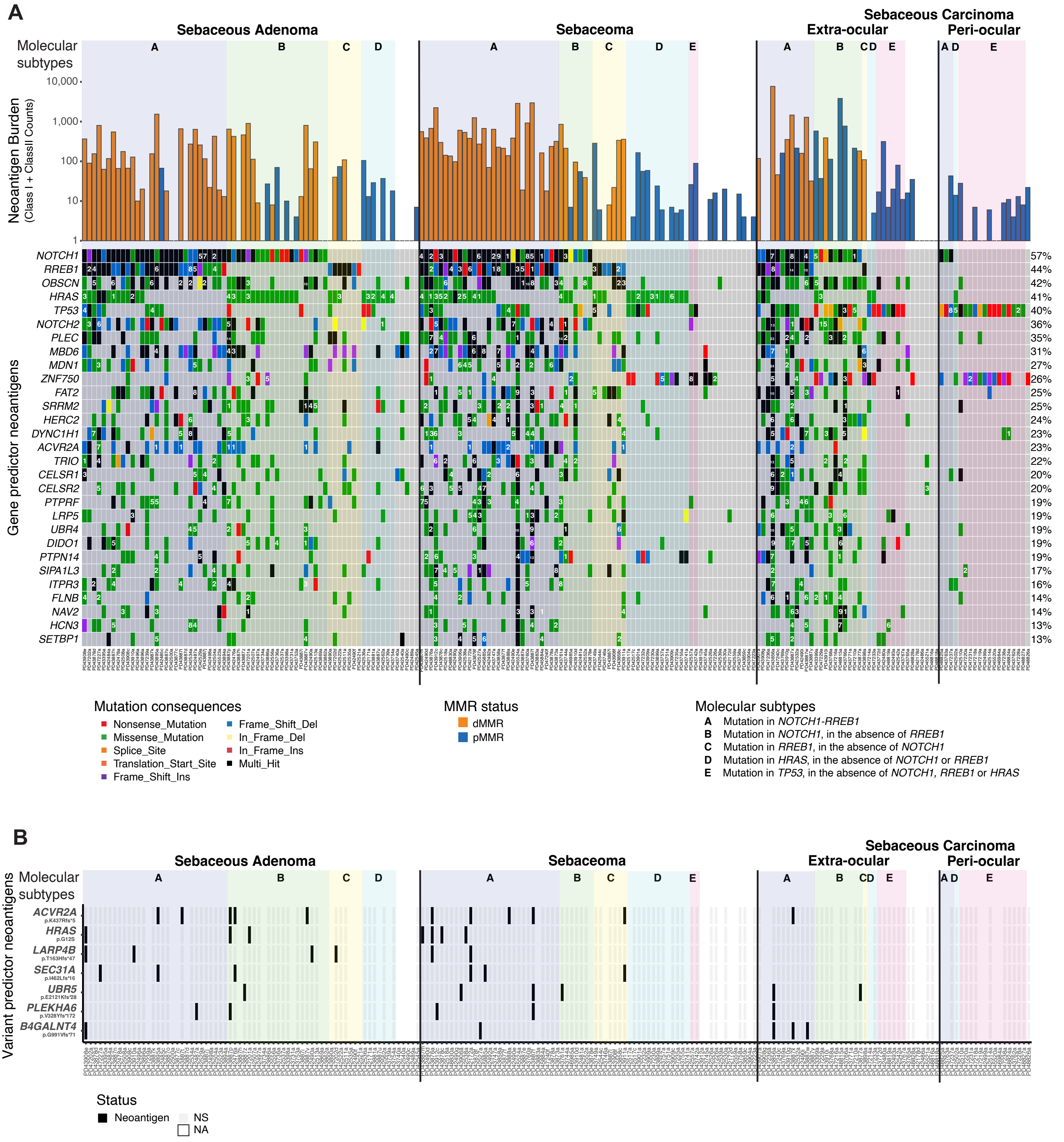

Supplementary Figure 12 – Landscape of gene mutations predicting neoantigen production in sebaceous tumours. (A) Oncoplot showing the propensity of somatic mutations to produce neoantigens in certain genes (genes shown where predicting neoantigens were found in at least 10 samples) of sebaceous tumours (STs; *n* = 197). The number in each box indicates the number of mutations predicted to produce neoantigens for the related gene. On the top, the barplot represents the neoantigen burden of each tumour (dMMR in orange and pMMR in blue). (B) Tileplot harbouring shared variants predicted to produce neoantigens across the cohort in at least 5 samples. Abbreviations: MMR, mismatch repair; dMMR, deficient in MMR; pMMR, proficient in MMR; NA, not analysed; NS, not significant.

Supplementary Figure 13

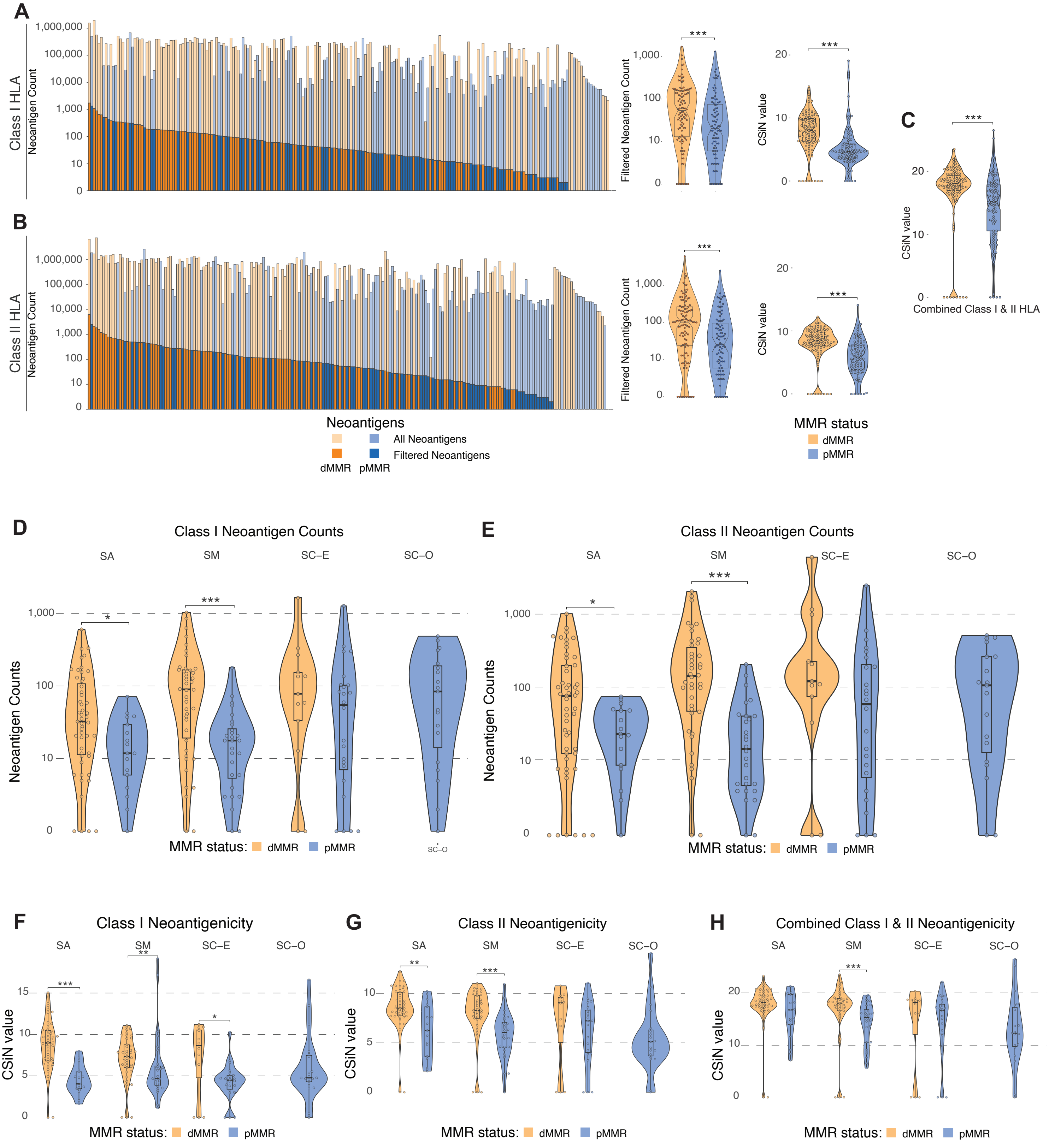

Supplementary Figure 13 – Neoantigen profile of sebaceous tumours. (A-B) Barplots showing neoantigen count (class-I (A) and class-II (B)) across the cohort (*n* = 194). Note the distinction between mismatch repair deficient (dMMR in orange; *n* = 106) and proficient (pMMR in blue; *n* = 88) STs, as well as non-filtered (light colour) and filtered (dark colour) neoantigens. (A-C) Violin boxplots displaying filtered neoantigen class-I (A) and class-II (B) count, and Cauchy-Schwartz index of neoantigens (CSiN) score evaluating responsiveness for class-I (A), class-II (B) and combined classes (class-I & class-II; C) as a genetics-based prediction method. (D-H) Violin boxplots representing neoantigen count for class-I (D) and class-II (E), and predicted neoantigenicity (CSiN score) for class-I (F) – class-II (G) and combined classes (class-I & class-II; H), across the four ST subtypes split between dMMR (in orange) and pMMR (in blue) (SA (*n* = 68; *n* = 51 dMMR and *n* = 17 pMMR), SM (*n* = 70; *n* = 42 dMMR and *n* = 28 pMMR), SC-E (*n* = 37; *n* = 13 dMMR and *n* = 24 pMMR and SC-O (*n* = 19)). Boxplot annotations: centre line, median; box limits, upper (75th) and lower (25th) quartiles; whiskers, 1.5x interquartile range; dots, values. Statistical test (ANOVA two-sided test followed by Tukey Honest Significant Differences (two-sided test)): *: p-value <0.05, **: p-value <0.01, ***: p-value <0.001 (exact p-value in Data Source SuppFigure 13_p-value).

SUPPLEMENTARY DISCUSSION

Histopathological Analysis

Histologically (**Figures 1A-R; below**), sebaceous adenomas (**Figure 1A**) and sebaceomas (**Figure 1B**) were usually well-circumscribed dome-shaped tumours (**Figure 1E**). Sometimes, specific architecture was encountered, predominantly observed in sebaceous adenoma (SA), such as delimitation by a collarette (SA 26.5%, SM 14.5%) or a polyp, warty, cystic, or keratoacanthoma-like (KA-like) growth pattern (SA 36.8%, SM 43.5%) (**Figure 1D**). Mostly occurring within the upper and mid dermis with epidermal connection/component (SA 91.2%, SM 62.3%), benign sebaceous tumours can also be deeply located into the dermis, even the subcutis, with no epidermal connection (SA 8.8%, SM 37.7%) (**Figure 1E**). While SA tended to retain a sebaceous gland architecture (**Figure 1G**) with tumour lobules composed of mature sebocytes surrounded by plump germinative basal cells, this layout disappeared in sebaceomas (SM) where germinative cells predominate over mature sebocytes (**Figure 1H**). SM also shows different growth patterns (**Figures 1H, K, N** and **Q**) with only sparse or small islands of mature sebocytes. Mature sebocytes are characterised by scalloped/indented nuclei with bubbly cytoplasm, while germinative basal cells are composed of scant cytoplasm and small nucleoli. Holocrine secretion was often observed, mostly in SA (**Figure 1J**). Cyst formation, keratocyst (**Figure 1H**), duct (**Figures 1M-N**) or squamous differentiation (**Figure 1P**) were occasional additional features. Mitotic activity may be brisk, especially in the germinative component, with no atypical mitoses. Tumours may ulcerate, but no necrosis, cytological atypia, perineural infiltration or lymphovascular invasion were encountered. Owing to their predominantly head and neck localisation, adjacent solar elastosis was frequently observed with severe elastosis more commonly observed in association with sebaceous adenoma (elastosis grading (**Supplementary Note** [see below]): 0, (SA 32.7%, SM 37%), 1 (SA 2%, SM 8.7%), 2 (SA 12.9%, SM 17.4%), 3 (SA 36.6%, SM 17.4%), *NA* (SA 15.8%, SM 19.6%)).

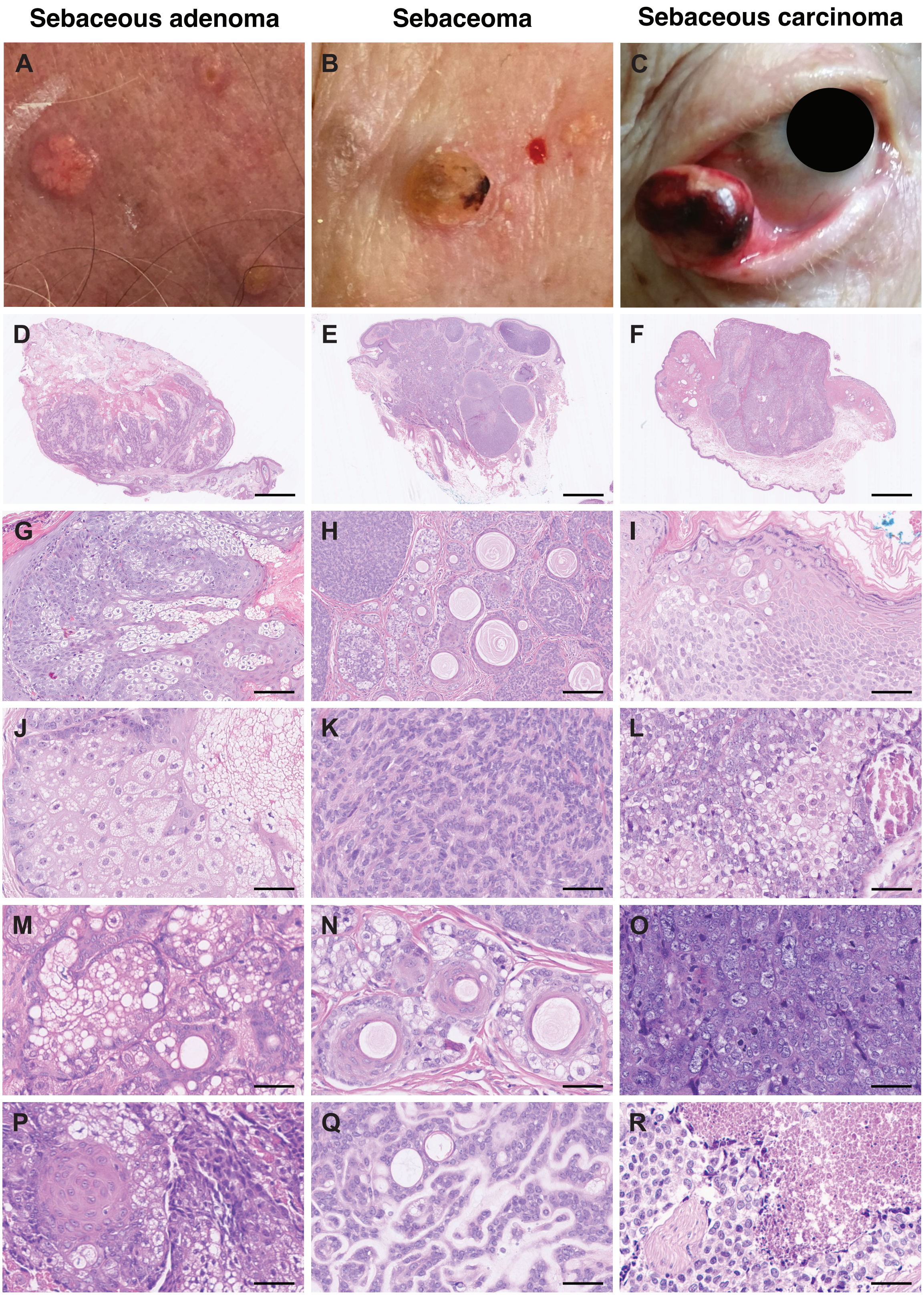

Figure 1 - Clinical and histopathological pictures of sebaceous tumours. Scale bar, 50 μm.

In contrast, sebaceous carcinomas (SC; **Figure 1C, above**) were usually ulcerated (SC-E 64.9%, SC-O 73.1%) infiltrating dermal tumours (**Figure 1F**). They were composed of variably sized nodules often connected to the epidermis (SC-E 73.7%, SC-O 78.9%) and composed of hypercellular sheets of cells, and less frequently of trabecules. Infiltrative margins were more commonly found in peri-ocular SC (SC-O) (architectural growth pattern: 1 (11.5%), 2 (46.2%), 3 (42.3%)) compared to extra-ocular (SC-E) (architectural growth pattern: 1 (49%), 2 (30.6%), 3 (16.3%), *NA* (4.1%)), sometimes invading the subcutis and/or underlying skeletal muscle (SC-E 26.5% of which 2/13 metastasized, SC-O 30.7% of which 4/8 metastasized). Tumour cells were usually basophilic with an increased nucleocytoplasmic ratio (**Figure 1O**), small to intermediate nucleoli, and a variable percentage of lipid-containing cells reminiscent of mature sebaceous cells. Sebaceous differentiation was usually more obvious in SC-E (cell differentiation grade (**Supplementary Note** [see below]): 1 (47%), 2 (42.9%), 3 (10.2%)) than SC-O (cell differentiation grade: 1 (3.8%), 2 (34.6%), 3 (61.5%)). Sometimes, tumour cells were characterised by a large clear cytoplasm or occasionally a voluminous pleomorphic nucleus. Cytological atypia (**Supplementary Note** [see below]) was also more often severe in SC-O (grade: 1 (3.8%), 2 (34.6%), 3 (61.5%)) than SC-E (grade: 1 (38.7%), 2 (51%), 3 (10.2%)). Intraepidermal spread or *in situ* component (**Figure 1I**) was present in 42.9% and 46% of SC-E and SC-O cases (SC-E 24/49; SC-O 12/26), respectively, with an additional squamous cell carcinoma in situ (SCCis) component in four SC-Es (2 from the scalp and 2 from the genital area). Mitoses were commonly seen (SC-E mean: 14/mm^2, median 10/mm^2 and range: 0-74/mm^2; SC-O mean: 19/mm^2, median 16.5/mm^2 and range: 2-66/mm^2). Lymphocytic infiltrates varied in intensity and were mostly seen surrounding tumour nodules rather than infiltrating them. Necrosis was often observed (SC-E 12/49; SC-O 13/26) usually as comedonecrosis (**Figure 1R**), as well as solar elastosis (SC-E: 0 (24.5%), 1 (6.1%), 2 (30.6%), 3 (34.7%), *NA* (4.1%); SC-O: 0 (23.1%), 1 (19.2%), 2 (46.2%), 3 (3.8%), *NA* (7.7%)) and calcifications (**Figure 1L**). Perineural infiltration (PNI) (**Figure 1R**) was found in 8% of sebaceous carcinomas (SC-E 4/49; SC-O 2/26), whereas lymphovascular invasion (LVI) was not seen. Overall, SC-O tended to have a less differentiated morphological appearance (architecture, cytological differentiation and atypia, mitosis rate, necrosis) compared to SC-E. Metastatic SC-Os (n=5) were ulcerated and deeply invading tumours (subcutis or skeletal muscle) characterised by architecture, cytological differentiation and atypia grade 3 (**Figure 1O**), elastosis grade 2, dense peritumoral lymphocytic infiltrate and retained expression of the MMR proteins. Metastatic SC-Es (n=3) were similar to metastatic SC-Os with architectural and cytological grading as well as elastosis varying from grade 2 to 3, with fibrosis being an additional finding.

For comparison, sebaceous hyperplasias were composed of increased normal sized sebaceous lobules discharging into a single centrally located duct. They contained predominantly mature sebocytes and a single layer of small germinative basaloid cells at the periphery. The keratoacanthoma with sebaceous differentiation was a well-circumscribed tumour characterised by a crateriform aspect with a centrally located keratin plug and surrounded by a collarette. Tumour cells were typically made of abundant

eosinophilic pale glassy cytoplasm. Focally, isolated or small islands of mature sebocytes were identified.

By immunohistochemistry, all pMMR primary tumours (n=130: 29 SAs, 41 SMs, 34 SC-Es and 26 SC-Os) showed retained expression of the MMR proteins, while all dMMR primary tumours (n=139: 73 SAs, 51 SMs and 15 SC-E, of which 27, 27 and 11 came from a LS patient) had lost the expression of at least one MMR protein (except for one case harbouring a *MSH6* missense mutation). Twenty-one dMMR tumours showed loss of MLH1/PMS2 expression and twelve of these were tested for *MLH1* promoter hypermethylation, which was found in two cases (as has previously been reported only in SM[3]) (**Supplementary Data 1**).

As expected, loss of one MMR protein by IHC was strongly associated with LS in the 154 patients with germline testing data. Specifically, 23/23 LS patients and 45/131 non-LS patients showed loss of expression of at least one MMR protein (Chi-square $p=1.91*10^{-08}$) and 23/68 (33%) patients with MMR expression loss had LS suggesting negative MMR IHC staining should raise the index of suspicious for LS.

In LS, STs can precede other cancers, and so, can be an inestimable information in the screening of LS carrier. The literature reports several algorithms/criteria, each including different variables such as diagnosis of SA, age at diagnosis under 60 years, presence of multiple STs, ST with KA-like or cystic architecture, and a diagnosis of a LS-associated cancer.[4, 5, 6, 7, 8] Giving the value of defining LS carriers and our large unprecedented collection of clinical and histopathological data, statistical testing were performed to find potential variables for the screening of patient with STs as LS patients/carriers.

To evaluate the clinical and histopathological attributes associated with LS, univariable and multivariable logistic regression was used. Of the six clinical attributes evaluated (encompassing having: multiple STs, ST located outside the head and neck area, an age at the ST diagnostic under 60 years, a LS-associated cancer, a family history of LS-associated cancer), three were significantly associated with LS on multivariable testing and after false-discovery correction, specifically developing multiple STs, having a tumour outside the head and neck, and having another non-ST LS-related neoplasm (multivariable odds ratio 22, 14, 8.8, respectively) (**Figure 2**).

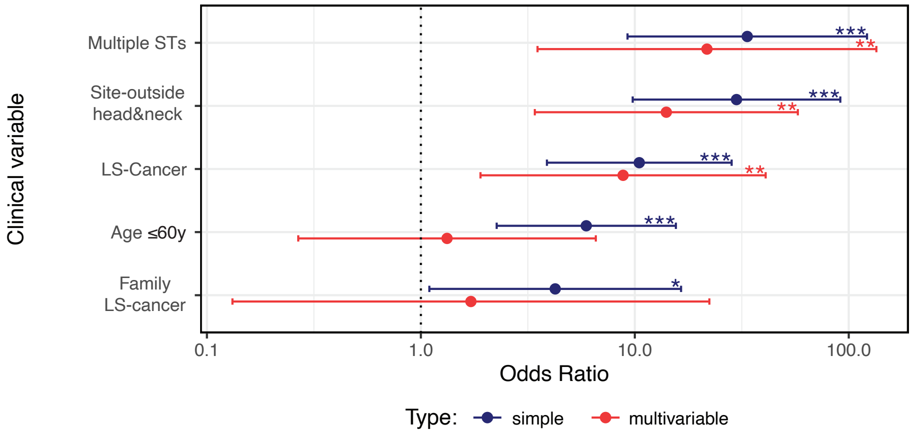

Figure 2 - Clinical variables tested to estimate their predicted potential of association with LS (n=150 patients). Data are presented as the odd ratio (circle) +/- the standard error (error bars). Statistical test (uni- and multi- variable logistic regression with adjustment using the Benjamini-Hochberg method for multiple comparisons): *: two-tailed adjusted p-value <0.05, **: two-tailed adjusted p-value <0.01, ***: two-tailed adjusted p-value <0.001 (exact p-value in Data Source Figure 1E_p-value). Variables significant (two-tailed adjusted p-value <0.05) by univariable logistic regression chosen for multivariable logistic regression testing. Reproduced in Figure 1 (see **Manuscript**).

Specific histopathological features like growth pattern such as polyp, cystic, warty-like, or KA-like – keratocysts – delimitation by a collarette – no connection with the overlying epidermis - as well as the histopathological diagnosis (SA, SM, SC-E) were tested. No epidermal connection and collarette appeared to be statistically significantly related to LS on multivariable testing and after false-discovery correction (multivariable odds ratio 5.6 and 4.8, respectively) (**Figure 3**).

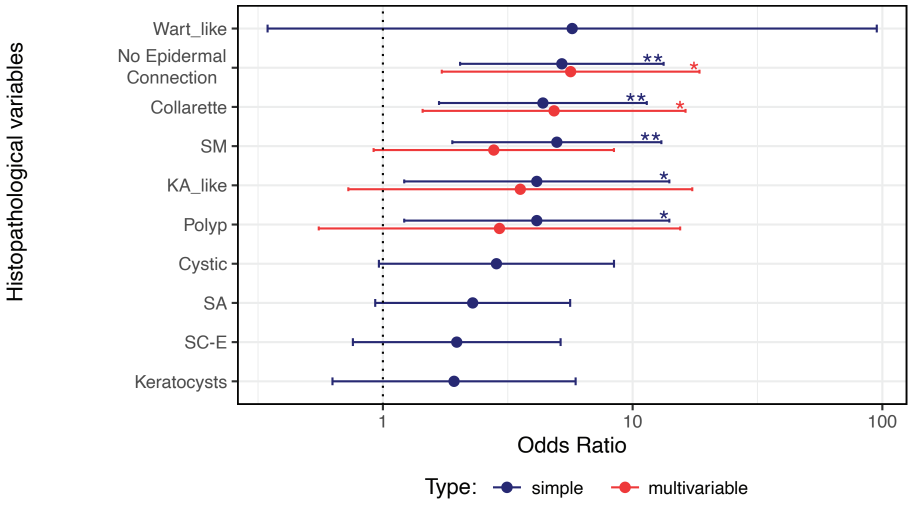

Figure 3 - Histopathological variables tested to estimate their predicted potential of association with LS (n=150 patients). Data are presented as the odd ratio (circle) +/- the standard error (error bars). Statistical test (uni- and multi-variable logistic regression with adjustment using the Benjamini-Hochberg method for multiple comparisons): *: two-tailed adjusted p-value <0.05, **: two-tailed adjusted p-value <0.01, ***: two-tailed adjusted p-value <0.001 (exact p-value in Data Source Figure 1E_p-value). Variables significant (two-tailed adjusted p-value <0.05) by univariable logistic regression chosen for multivariable logistic regression testing. Reproduced in Figure 1 (see **Manuscript**).

Further validation using bootstrapping (number of bootstrap samples, B=100) and crossvalidation, confirm the significance of these variables and added SM and KA-like variables to the histopathological model with marginal predictive value. Finally, leave-one-out crossvalidation shows an AUC of 0.87 and 0.71, for the clinical and histopathological models, respectively (**Figure 4**), suggesting that the clinical model is a better predictor than the histopathological model. Interestingly, when combining these two models (clinic-histopathological model), the variables that came up as significant were the same as the ones obtained by the clinical model. This suggests that all the information related to Lynch syndrome and collected in the histopathological variables are already represented by the clinical variables.

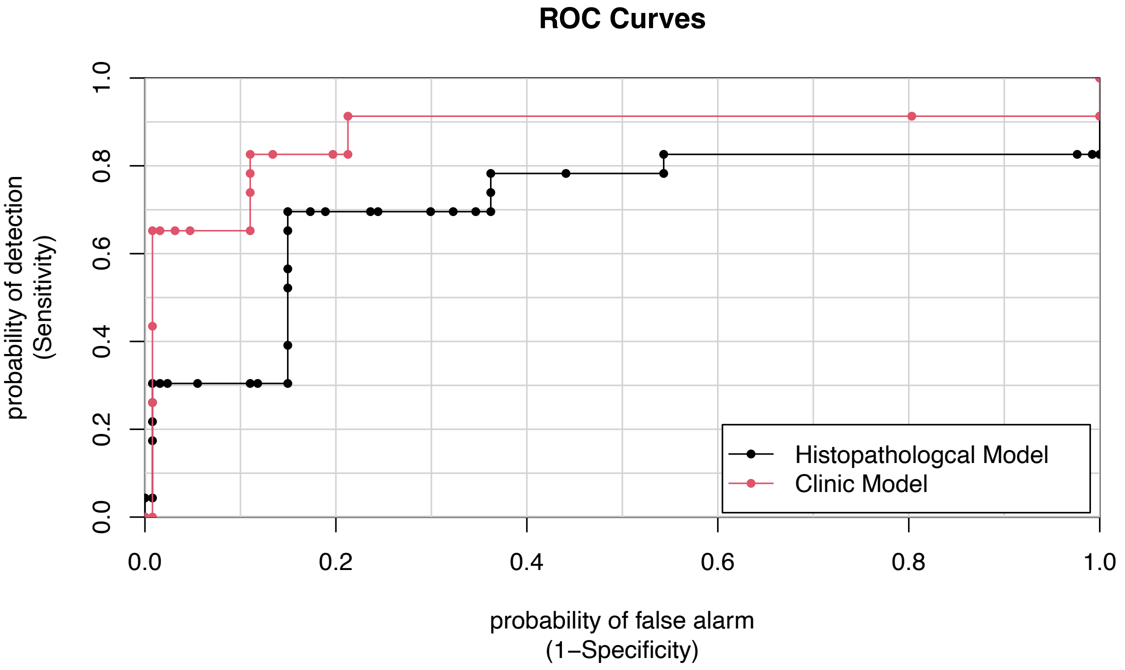

Figure 4 - Receiver Operating Characteristic (ROC) curve for dMMR detection. Using logistic regression for both clinical and histopathological models, and computing the ROC/area under the curve (AUC) using leave-one-out cross validation to define the best predictor for LS, the AUC shown that the clinical model (AUC = 0.87) is a better predictor than the histopathological model (AUC = 0.71). Reproduced from Supplementary Figure 2 in **Supplementary Data 1**.

Using a threshold of >0.1 on the predicted probability of LS computed with our clinical model to increase the detection of LS patient, multiple STs – an ST located outside the head and neck area and a LS-associated cancer appear to be valuable clinical variables to screen LS patient with a sensitivity of 91.3% and a specificity of 78.7%. The following table (**Table 1**) shows the probability of being LS carrier based on the three significant clinical variables:

	**MultipleST**	**Location outside the head&neck area**	**LS-associated cancer**	**Probability to be Lynch**
**1**	No	No	No	2%
**2**	No	No	Yes	15.9%
**3**	No	Yes	No	23.9%
**4**	Yes	No	No	31.8%
**5**	No	Yes	Yes	74.5%
**6**	Yes	No	Yes	81.2%
**7**	Yes	Yes	No	87.8%
**8**	Yes	Yes	Yes	98.5%

Table 1 - Probability to be Lynch syndrome carrier based on the clinical model.

In comparison, using a threshold of >0.1 on the predicted probability of LS computed with our histopathological model to increase the detection of LS patient, no epidermal connection – collarette - KA-like and SM seem to be valuable histopathological variables in the screening of LS carrier with a sensitivity of 73.9% and a specificity of 63.8%; although less powerful than the clinical model. The following table (**Table 2**) shows the probability of being LS carrier based on the four significant histopathological variables:

	NoEpidermalConnection	Collarette	KA_like	SM	Probability to be Lynch
**1**	No	No	No	No	3.0%
**2**	No	No	No	Yes	8.6%
**3**	No	No	Yes	No	11.9%
**4**	No	Yes	No	No	12.8%
**5**	Yes	No	No	No	15.3%
**6**	No	No	Yes	Yes	29.0%
**7**	No	Yes	No	Yes	30.9%
**8**	Yes	No	No	Yes	35.3%
**9**	No	Yes	Yes	No	39.1%
**10**	Yes	No	Yes	No	44.0%
**11**	Yes	Yes	No	No	46.1%
**12**	No	Yes	Yes	Yes	66.0%
**13**	Yes	No	Yes	Yes	70.4%
**14**	Yes	Yes	No	Yes	72.2%
**15**	Yes	Yes	Yes	No	78.9%
**16**	Yes	Yes	Yes	Yes	91.9%

Table 2 - Probability to be Lynch syndrome carrier based on the histopathological model.

In conclusion, based on this cohort, the clinical and histopathological variables appear applicable variables in the screening of LS carriers in patient with STs and could be become important tools in clinical practice. The clinical model was demonstrated to be more powerful than the histopathological model, and thus should be the preferred option where possible. Of note, the results obtained could not be validated as, to our knowledge, no other study has collected as much clinical and histopathological data as ours. Nevertheless, when possible, further validation should be performed.

Analysis of Driver genes

The NOTCH signalling pathway plays a key role in sebaceous gland differentiation and expansion[9]. Consistent with this, somatic inactivation of the driver gene *NOTCH1* was the most recurrent alteration in this ST cohort. *NOTCH1* inactivation occurred via mutation, consistent with previous reports. Multiple *NOTCH1* mutations per sample were often observed and were preferentially located within the extracellular epidermal growth factor (EGF) domain and with a recurrent variant p.A465T observed (**Supplementary**

Figure 6A in **Supplementary Information**). *NOTCH1* mutations, with or without *RREB1* mutations have previously been reported to occur in dMMR and UV-mutated pMMR SC-Es[10], however, this study demonstrated that such a profile also can be found in benign STs (**Figure 4** (see **Manuscript**)). In addition, biallelic inactivation of *NOTCH1* in STs also occurred via copy neutral loss of heterozygosity (cnLOH), as shown for the first time in this study (**Figure 5** (see **Manuscript**)). Another driver gene was *HRAS*, with recurrent variants at p.G13R, p.A18V, p.R68Q/W, p.A146P/T/V, and p.K117N/R. Interestingly, p.G13R was only found in pMMR SC-E cases, and the other variants occurred only in dMMR STs (consistent with previous reports[10, 11]), with only rare reports of all these variants in COSMIC. Thus, *HRAS* mutations were found in both benign (SA and SM) and malignant (SC-E) dMMR tumours and pauci-mutated pMMR tumours (**Figure 2** (see **Manuscript**)), whereas previously they had only been identified in SC-E[10, 11]. Importantly, HNSCC (head and neck squamous cell carcinoma) with mutations in exon 2 (p.G12C, p.G12S, p.G13R), exon 3 (p.Q61L) or exon 4 (p.K117N, p.A146T) of *HRAS* appear to be sensitive to farnesyltransferase inhibition via Tipifarnib[12], which has implications for pMMR SC-E with *HRAS* p.G13R mutations that are typically not suitable for immune checkpoint inhibitors. *MBD6*, identified as a ST driver gene in this study, is a methyl-CpG-binding domain protein that binds the subunits ASXL1-3, and stabilizes the *BAP1* complex on chromatin (or Polycomb repressive complex PR-DUB)[13]. Interestingly, *ASXL1* was recurrently mutated in STs, with indels frequently being found in both *MBD6* and *ASXL1*. The *ASXL1* nonsense and frameshift mutations are predicted to result in the formation of truncated proteins with increased stability, which in turn would increase the *BAP1* complex association with chromatin and thus increment transcription of target genes[14]. Mutations in *MBD6* have been reported in multiple cancer types[13, 15, 16] and analysis of the TCGA data revealed that the *MBD6* mutations were more frequently found in LS-related cancer types (i.e. bladder, kidney, stomach, colorectal, pancreas and endometrial cancers, and glioblastoma[15]). As the *MBD6* frameshift mutations in theory would result in destabilisation of the BAP1 complex, resulting in decreased homologous recombination-mediated double-strand DNA repair[17], it is not surprising that such mutations were primarily found in dMMR STs. Interestingly, a small molecule inhibitor-mediated inhibition of *BAP1* catalytic activity has recently been described as a possible new targeted therapy for *ASXL1*-mutated leukemia[14].

Mutation of the *TP53* TSG is commonly found in sun-exposed skin cancer [reviewed in [18]]. However, *TP53* mutation is not always related to a UV-mutational signature, especially in SC-O tumours[19] (**Figure 4** (see **Manuscript**)), and it is not only linked to malignant tumours, with *TP53* mutations also seen in the benign SA and SM tumours[19, 20] (**Figure 2** (see **Manuscript**)); however, biallelic inactivation was identified only in SCs. Additionally, *TP53* mutation can co-occur with *ZNF750* and/or *RB1* in the malignant tumours (particularly SC-O), suggesting *TP53* might accelerate *ZNF750/RB1*-driven SC as occurs for *BRAF* p.V600E in melanoma[21]. In support of this hypothesis, concomitant *TP53/RB1* mutations were identified in some metastatic SC cases. Previous reports have found the *RB1* TSG to be somatically mutated preferentially in malignant STs compared to benign STs[10, 22]. This study reinforces the important role of *RB1* in malignant STs, with inactivation occurring via somatic mutation, significant focal deletion (in SC-E)

and significant 13q deletion (in SC-O) (**Figures 2 and 6** (see **Manuscript**), **and Supplementary Figure 10** in **Supplementary Information**). This contrasts with the benign STs, where very few SA and SM showed *RB1* mutations, and were not associated with *TP53* mutations (**Figure 1** (see **Manuscript**)). Similarly, mutations in the *ZNF750* TSG have been reported to be linked to pauci-mutational SC-O, although also found in a subset SC-E[10, 23, 24]. This study showed inactivation of *ZNF750* in all STs, although it was inactivated more frequently in the malignant subtypes with these events often co-occurring with *TP53* mutation (**Figures 2 and 5** (see **Manuscript**)).

MMR deficiency is the most well-characterised pathway of tumorigenesis for STs (except for SC-O). The 'first hit' can be a constitutional (germline) or sporadic mutation of an MMR gene(s), with the 'second hit' being a somatic mutation (**Figure 2** (see **Manuscript**)) or deletion (CN loss). This study revealed a novel third mechanism for the 'second hit', specifically MMR gene cnLOH (**Figure 5** (see **Manuscript**)), that has not previously been reported in STs. Interestingly, the mechanism for disruption of the second allele tended to differ across tumours from the same LS patient, defining tumours from the same patient as being independent. This project also showed for the first time that STs can arise in patients with constitutional variants in at least two different MMR genes (such as in digenic/double heterozygous LS)[25]. As highlighted in this study, ST can be the first sign of LS (from 9% and possibly up to 52%) and some additional clinical variables, such as the development of multiple sebaceous tumours – ST location outside the head and neck area or development of a LS-associated cancer, can suggest a referral for LS constitutional testing is warranted. Histological features, like specific growth pattern (KA-like) – location strictly within the dermis or subcutis – delimitation by a collarette or a diagnosis of SM, can also suggest a patient has LS. As an MMR constitutional mutation is not detectable in 35% of patients with a ST arising in a syndromic context, tumours are classified as dMMR positive/MTS-type1 or dMMR negative/MTS-type2[26, 27]. MTS-type2 tumours are suspected to have other constitutional molecular or epigenetic mechanisms such as *MUTYH* mutations or *MLH1* promoter hypermethylation[26, 28, 29], with the former event seen in patients within this study.

Intriguingly, many the genes altered in this study interact (directly or indirectly) with the Hippo pathway and its downstream effectors *YAP/TAZ* as shown in **Figures 5** and **6**). This signalling cascade regulates cell proliferation, tissue homeostasis, differentiation, apoptosis, regeneration, and organ size [review in [30, 31]]. Although the Hippo pathway functions as a tumour suppressor, genomic alterations activating *YAP* and *TAZ* can be regarded as both oncogenic and tumour suppressive events, depending on the transcription factors they interact with [reviewed in [30, 32, 33]]. Importantly, Hippo pathway members, as well as YAP/TAZ proteins, can be targeted for therapy [review in [31, 33]].

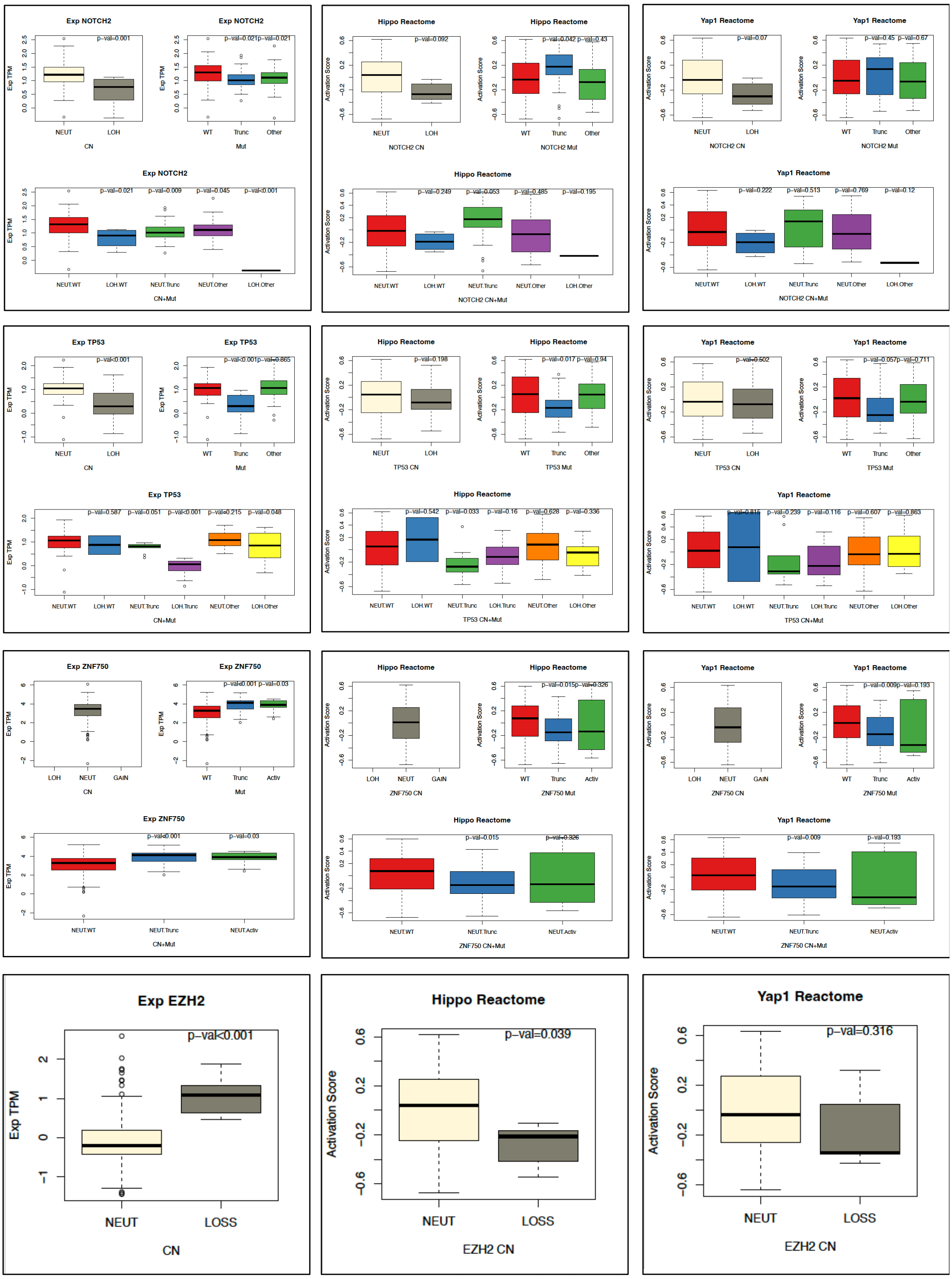

Figure 5 – Impact of *NOTCH2* – *TP53* – *ZNF750* – *EZH2* alterations on Hippo and YAP1 pathways activation/inactivation. Boxplot evaluating genes expressions (TPM), as well as Hippo and YAP1 pathways (REACTOME). 1) *NOTCH2*: truncating mutation decreases its expression (p-value = 0.021, linear model t-test) and activates Hippo pathway (p-value = 0.042, linear model t-test). 2) *TP53*: truncating mutation decreases its expression (p-value < 0.001, linear model t-test) and inactivates Hippo pathway (p-value = 0.017, linear model t-test). 3) *ZNF750*: truncating mutation increases its expression (p-value < 0.001, linear model t-test) and inactivates Hippo and YAP1 pathways (p-value = 0.015 and 0.009, respectively, linear model t-test). 4) *EZH2*: deletion of the gene increases its expression (p-value < 0.001) and inactivates Hippo pathway (p-value = 0.039, linear model t-test).

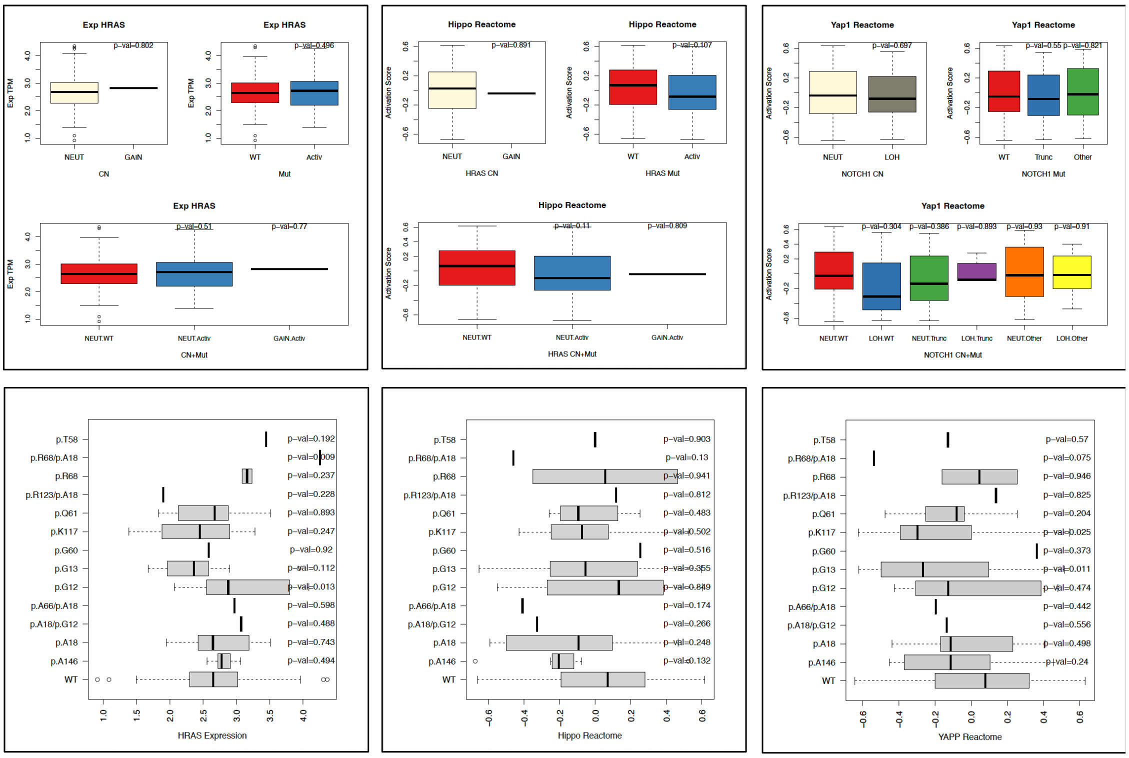

Figure 6 – Impact of *HRAS* alterations *and its variants* on Hippo and YAP1 pathways activation/inactivation. Boxplot evaluating genes expressions (TPM), as well as Hippo and YAP1 pathways (REACTOME). Overall, *HRAS* does not seem to alter the Hippo or YAP1 pathway. However, considering specific variants such as p.G13 and p.K117, their mutations appeared to inactivate the YAP1 pathway (p-value = 0.011 and 0.025, respectively, linear model t-test).

The somatic TMB rate (which includes SNVs, MNVs and indels) and profile has been reported as an effective metric to differentiate dMMR-related cancers from non-dMMR related cancers, as dMMR tumours are characterised by hypermutability and microsatellite instability[25, 34]. However, high TMB is not necessarily always linked to MSI [reviewed in [35]]. The results from this study show that the indel rate is a more reliable metric for correlating dMMR status in STs, due to defective MMR and MSI resulting in increased number of indels, consistent with previous observations[10, 11, 36]. Using the indel rate is particularly important for skin tumours where UV light exposure induces a high TMB independent of dMMR status, in addition to other specific factors such as *APOBEC* activation and mutations affecting other DNA repair mediators such as polymerase *ε* (*POLE*) [review in [35]]. The indel rate cannot be used, firstly, to differentiate sporadic dMMR from germline dMMR tumours and, secondly, as a metric to detect dMMR-*MSH6* samples, which are characterised by SNV mutations only[37]. Similarly, unlike CRCs [reviewed in [25]], somatic *BRAF* p.V600E and *MLH1* promoter hypermethylation testing is not a major contributor to STs and cannot be employed to distinguishing sporadic from germline dMMR tumours owing to the absence of *BRAF* p.V600E mutations in these tumours and our observation that hypermethylation of the *MLH1* promoter is a rare event in sporadic dMMR STs.

Lastly, a differential diagnosis for SA is SH[38], thus the molecular similarity between these entities was assessed. The mutational profile of SH (n=4) was very different from SA with only two of the ST driver genes mutated, specifically *LATS1* and *ZFP36L2* (n=1 sample each; **Supplementary Figure 3C** in **Supplementary Information**), and no mutations in the ST top 20 most recurrently mutated genes (**Supplementary Figure 3B** in **Supplementary Information**). The KA had mutations in nine of the ST driver genes (**Supplementary Figure 3C** in **Supplementary Information**) and 11 mutations in the ST top 20 most recurrently mutated genes (**Supplementary Figure 3B** in **Supplementary Information**), with mutations in genes like those seen in other dMMR tumours from the same LS patient, indicating possible molecular overlap between STs and this single KA.

Analysis of fusion genes and viral sequences in sebaceous tumours

In addition to *PAK2* fusions, there was an interchromosomal *NSD3::NUTM1* fusion (**Supplementary Figure 11C** in **Supplementary Information**), previously reported in midline carcinoma[39], which was identified in one SC-E and its regional lymph node metastasis (**Supplementary Data 15**). After further consideration and case review with additional immunohistochemistry stains, the case was best regarded as a NUT carcinoma, a newly described entity[40, 41, 42, 43], where *NSD3* was unreported as a fusion partner in skin. In addition, *NUT* fusions have been reported in adnexal skin tumours, such as *YAP1::NUTM1*, *WWTR1::NUTM1* [44, 45] and *EMC7::NUTM1*[45] in poroid neoplasms[44, 45], as well as *BRD3::NUTM2B* in a skin adnexal variant with follicular infundibular differentiation[42]. In the fusions that did not code for inframe proteins, events involving several established cancer genes were observed including *KIT* (*KIT::CHCHD7*), *NFE2L3* (*NFE2L3::THSD7A*) and *EGFR* (*EGFR::AUTS2*) (**Supplementary Figure 11D** in **Supplementary Information**).

Some subtypes of STs have been associated with viral infections, such as human immunodeficiency virus (HIV) in SCs[46], and human papillomavirus (HPV) in both benign and malignant histology subtypes[47, 48]. In particular, HPVs are DNA viruses that span many species and serotypes, for example Alphapapillomavirus 7 (αPV7; including sertoypes HPV18, 39, 45, 59, 68, 70, 85, 97) and αPV9 (including serotypes HPV16, 31 33, 35, 52, 58, 67) (https://ictv.global/). To look for possible viral aetiologies in our ST cohort, whole-exome and transcriptome reads were analysed for the presence of viral sequences using Kraken2[49] (see **Methods**). DNA and RNA sequences of αPV7 were identified in a pMMR SC-E from the pubis of a male (PD48821a and PR48821a, respectively) (**Supplementary Data 14**). Additionally, sequences of αPV9 were identified in a pMMR SC-E from the genital area (vulva) of a patient who concurrently developed a cervical cancer (PD45521a; only DNA data was available for this patient). These SCs cases showing high-risk HPV virotypes were both pMMR, consistent with previous case reports[50, 51]. Of note, our approach did not detect HPV in any SC-O cases[47]. Interestingly, the two HPV-positive genital SC-Es were wild-type for *TP53* and *RB1*. Convincing evidence for the presence of other viral sequences was not found but this may reflect the

use of whole-exome/pulldown transcriptome sequencing rather than whole-genome/whole-transcriptome sequencing.

SUPPLEMENTARY NOTE

HISTOPATHOLOGICAL CRITERIA	DESCRIPTION
**UV DAMAGE**[52]	**Grade 1**: single elastic fibres<br>**Grade 2**: bunches of fibres<br>**Grade 3**: basophilic material with no fibrillary texture.
**ARCHITECTURAL GROWTH PATTERNS**[52, 53, 54]	**Grade 1**: Tumour with well-demarcated, roughly equally sized cellular lobules<br>**Grade 2**: Tumour with an admixture of well-defined nests with infiltrative profiles or confluent cell groups<br>**Grade 3**: Tumour with highly invasive growth or a medullary sheet-like pattern
**CYTOLOGICAL DIFFERENTIATION**[47]	Histopathologic grade was defined according to the nuclear morphology and cytoplasmic evidence of sebaceous differentiation and determined according to the predominance (>50%) of the tumour cell population.<br>**Grade 1**: Well-differentiated tumours showed only slightly enlarged nuclei and frank evidence of sebaceous differentiation.<br>**Grade 2**: Intermediate grade tumours exhibited similar enlarged nuclei but with lower nuclear:cytoplasmic ratio and more obvious evidence of sebaceous differentiation.<br>**Grade 3**: High-grade tumours exhibited enlarged irregular nuclei with high nuclear:cytoplasmic ratio and only focal evidence sebaceous differentiation (intracytoplasmic vacuoles indenting the nucleus).
**CYTOLOGICAL ATYPIA**	**Grade 1**: Mild atypia<br>**Grade 2**: Moderate atypia<br>**Grade 3**: Severe atypia

REFERENCES

1. Bailey MH*, et al.* Comprehensive Characterization of Cancer Driver Genes and Mutations. *Cell* **173**, 371-385 e318 (2018).

2. Argelaguet R*, et al.* Multi-Omics Factor Analysis-a framework for unsupervised integration of multi-omics data sets. *Mol Syst Biol* **14**, e8124 (2018).

3. Kuwabara K*, et al.* Prevalence and molecular characteristics of DNA mismatch repair protein-deficient sebaceous neoplasms and keratoacanthomas in a Japanese hospital-based population. *Jpn J Clin Oncol* **48**, 514-521 (2018).

4. Shalin SC, Lyle S, Calonje E, Lazar AJ. Sebaceous neoplasia and the Muir-Torre syndrome: important connections with clinical implications. *Histopathology* **56**, 133-147 (2010).

5. Lazar AJ, Lyle S, Calonje E. Sebaceous neoplasia and Torre-Muir syndrome. *Curr Diagn Pathol* **13**, 301-319 (2007).

6. Singh RS*, et al.* Site and tumor type predicts DNA mismatch repair status in cutaneous sebaceous neoplasia. *Am J Surg Pathol* **32**, 936-942 (2008).

7. Dores GM, Curtis RE, Toro JR, Devesa SS, Fraumeni JF, Jr. Incidence of cutaneous sebaceous carcinoma and risk of associated neoplasms: insight into Muir-Torre syndrome. *Cancer* **113**, 3372-3381 (2008).

8. Rutten A*, et al.* Cystic sebaceous tumors as marker lesions for the Muir-Torre syndrome: a histopathologic and molecular genetic study. *Am J Dermatopathol* **21**, 405-413 (1999).

9. Veniaminova NA*, et al.* Niche-Specific Factors Dynamically Regulate Sebaceous Gland Stem Cells in the Skin. *Dev Cell* **51**, 326-340 e324 (2019).

10. North JP*, et al.* Cell of origin and mutation pattern define three clinically distinct classes of sebaceous carcinoma. *Nat Commun* **9**, 1894 (2018).

11. Georgeson P*, et al.* Tumor mutational signatures in sebaceous skin lesions from individuals with Lynch syndrome. *Mol Genet Genomic Med* **7**, e00781 (2019).

12. Gilardi M*, et al.* Tipifarnib as a Precision Therapy for HRAS-Mutant Head and Neck Squamous Cell Carcinomas. *Mol Cancer Ther* **19**, 1784-1796 (2020).

13. Tsuboyama N, Szczepanski AP, Zhao Z, Wang L. MBD5 and MBD6 stabilize the BAP1 complex and promote BAP1-dependent cancer. *Genome Biol* **23**, 206 (2022).

14. Wang L*, et al.* Epigenetic targeted therapy of stabilized BAP1 in ASXL1 gain-of-function mutated leukemia. *Nat Cancer* **2**, 515-526 (2021).

15. Du Q, Luu PL, Stirzaker C, Clark SJ. Methyl-CpG-binding domain proteins: readers of the epigenome. *Epigenomics* **7**, 1051-1073 (2015).

16. Choi YJ, Yoo NJ, Lee SH. Mutation and expression of a methyl-binding protein 6 (MBD6) in gastric and colorectal cancers. *Pathol Oncol Res* **21**, 857-858 (2015).

17. Yu H*, et al.* Tumor suppressor and deubiquitinase BAP1 promotes DNA double-strand break repair. *Proc Natl Acad Sci U S A* **111**, 285-290 (2014).

18. Pfeifer GP. Mechanisms of UV-induced mutations and skin cancer. *Genome Instab Dis* **1**, 99-113 (2020).

19. Tetzlaff MT*, et al.* Next-generation sequencing identifies high frequency of mutations in potentially clinically actionable genes in sebaceous carcinoma. *J Pathol* **240**, 84-95 (2016).

20. Harvey NT, Tabone T, Erber W, Wood BA. Circumscribed sebaceous neoplasms: a morphological, immunohistochemical and molecular analysis. *Pathology* **48**, 454-462 (2016).

21. Viros A*, et al.* Ultraviolet radiation accelerates BRAF-driven melanomagenesis by targeting TP53. *Nature* **511**, 478-482 (2014).

22. North JP, Solomon DA, Golovato J, Bloomer M, Benz SC, Cho RJ. Loss of ZNF750 in ocular and cutaneous sebaceous carcinoma. *J Cutan Pathol* **46**, 736-741 (2019).

23. Bao Y*, et al.* Mutations in TP53, ZNF750, and RB1 typify ocular sebaceous carcinoma. *J Genet Genomics* **46**, 315-318 (2019).

24. Xu S*, et al.* Whole-exome sequencing for ocular adnexal sebaceous carcinoma suggests PCDH15 as a novel mutation associated with metastasis. *Mod Pathol* **33**, 1256-1263 (2020).

25. Cerretelli G, Ager A, Arends MJ, Frayling IM. Molecular pathology of Lynch syndrome. *J Pathol* **250**, 518-531 (2020).

26. Ponti G, Ponz de Leon M. Muir-Torre syndrome. *Lancet Oncol* **6**, 980-987 (2005).

27. John AM, Schwartz RA. Muir-Torre syndrome (MTS): An update and approach to diagnosis and management. *J Am Acad Dermatol* **74**, 558-566 (2016).

28. Kuismanen SA, Holmberg MT, Salovaara R, de la Chapelle A, Peltomaki P. Genetic and epigenetic modification of MLH1 accounts for a major share of microsatellite-unstable colorectal cancers. *Am J Pathol* **156**, 1773-1779 (2000).

29. Cunningham JM*, et al.* Hypermethylation of the hMLH1 promoter in colon cancer with microsatellite instability. *Cancer Res* **58**, 3455-3460 (1998).

30. Faraji F, Ramirez SI, Anguiano Quiroz PY, Mendez-Molina AN, Gutkind JS. Genomic Hippo Pathway Alterations and Persistent YAP/TAZ Activation: New Hallmarks in Head and Neck Cancer. *Cells* **11**, (2022).

31. Samji P, Rajendran MK, Warrier VP, Ganesh A, Devarajan K. Regulation of Hippo signaling pathway in cancer: A MicroRNA perspective. *Cell Signal* **78**, 109858 (2021).

32. Howard A, Bojko J, Flynn B, Bowen S, Jungwirth U, Walko G. Targeting the Hippo/YAP/TAZ signalling pathway: Novel opportunities for therapeutic interventions into skin cancers. *Exp Dermatol* **31**, 1477-1499 (2022).

33. Zhang K*, et al.* YAP and TAZ Take Center Stage in Cancer. *Biochemistry* **54**, 6555-6566 (2015).

34. Muller MF, Ibrahim AE, Arends MJ. Molecular pathological classification of colorectal cancer. *Virchows Arch* **469**, 125-134 (2016).

35. Luchini C*, et al.* ESMO recommendations on microsatellite instability testing for immunotherapy in cancer, and its relationship with PD-1/PD-L1 expression and tumour mutational burden: a systematic review-based approach. *Ann Oncol* **30**, 1232-1243 (2019).

36. Fujimoto A*, et al.* Comprehensive analysis of indels in whole-genome microsatellite regions and microsatellite instability across 21 cancer types. *Genome Res* **30**, 334-346 (2020).

37. Helderman NC*, et al.* Molecular Profile of MSH6-Associated Colorectal Carcinomas Shows Distinct Features From Other Lynch Syndrome-Associated Colorectal Carcinomas. *Gastroenterology* **165**, 271-274 e272 (2023).

38. Ferreira I, Wiedemeyer K, Demetter P, Adams DJ, Arends MJ, Brenn T. Update on the pathology, genetics and somatic landscape of sebaceous tumours. *Histopathology* **76**, 640-649 (2020).

39. Chau NG*, et al.* An Anatomical Site and Genetic-Based Prognostic Model for Patients With Nuclear Protein in Testis (NUT) Midline Carcinoma: Analysis of 124 Patients. *JNCI Cancer Spectr* **4**, pkz094 (2020).

40. Kervarrec T*, et al.* Reply to: Expanding the Spectrum of Primary Cutaneous Carcinoma With BRD3-NUTM1 Fusion. *Am J Surg Pathol* **45**, 1584-1586 (2021).

41. Nishimura Y, Ryo E, Yamazaki N, Yatabe Y, Mori T. Cutaneous Primary NUT Carcinoma With BRD3-NUTM1 Fusion. *Am J Surg Pathol* **45**, 1582-1584 (2021).

42. Rubio Gonzalez B, Ortiz MV, Ross DS, Busam KJ. Skin adnexal carcinoma with BRD3-NUTM2B fusion. *J Cutan Pathol* **48**, 1508-1513 (2021).

43. Shah A, Box A, Brenn T, Flaman A. Primary cutaneous NUT carcinoma with BRD4::NUTM1 fusion. *J Cutan Pathol*, (2024).

44. Sekine S*, et al.* Recurrent YAP1-MAML2 and YAP1-NUTM1 fusions in poroma and porocarcinoma. *J Clin Invest* **129**, 3827-3832 (2019).

45. Macagno N*, et al.* NUT Is a Specific Immunohistochemical Marker for the Diagnosis of YAP1-NUTM1-rearranged Cutaneous Poroid Neoplasms. *Am J Surg Pathol* **45**, 1221-1227 (2021).

46. Lanoy E, Dores GM, Madeleine MM, Toro JR, Fraumeni JF, Jr., Engels EA. Epidemiology of nonkeratinocytic skin cancers among persons with AIDS in the United States. *AIDS* **23**, 385-393 (2009).

47. Tetzlaff MT*, et al.* Distinct Biological Types of Ocular Adnexal Sebaceous Carcinoma: HPV-Driven and Virus-Negative Tumors Arise through Nonoverlapping Molecular-Genetic Alterations. *Clin Cancer Res* **25**, 1280-1290 (2019).

48. Saliba M*, et al.* Sebaceous neoplasms: prevalence of HPV infection and relation to immunohistochemical surrogate markers. *Eur J Dermatol* **31**, 170-175 (2021).

49. Wood DE, Lu J, Langmead B. Improved metagenomic analysis with Kraken 2. *Genome Biol* **20**, 257 (2019).

50. Omori Y*, et al.* Bowen's carcinoma of the penis with sebaceous differentiation associated with human papillomavirus type 16. *Int J Dermatol* **53**, e531-532 (2014).

51. Hamza MA, Quick CM, Williams HR, Patil NM, Shalin SC. HPV-associated Vulvar Intraepithelial Carcinoma With Sebaceous Differentiation: Report of 2 Cases. *Int J Gynecol Pathol* **42**, 338-346 (2023).

52. Rutten A WM, Sangueza OP, Wallace C. In: *World Health Organization classification of tumours, pathology & genetics, skin tumours.* (ed LeBoit PE BG, Weedon D, Sarasin A.) (2006).

53. Rao NA, Hidayat AA, McLean IW, Zimmerman LE. Sebaceous carcinomas of the ocular adnexa: A clinicopathologic study of 104 cases, with five-year follow-up data. *Hum Pathol* **13**, 113-122 (1982).

54. Patterson JW WM. In: *Nonmelanocytic tumors of the skin*). AFIP (2006).
