# Supplementary material for: The molecular cartography of malignant and benign sebaceous tumours
Source: Nat Commun. 2025 Dec 19;17:14. doi: 10.1038/s41467-025-66584-0 (PMC12764876; doi:10.1038/s41467-025-66584-0)
Supplement: Supplementary file 3 — Reporting Summary [file 41467_2025_66584_MOESM3_ESM.pdf]

nature portfolio

Corresponding author(s): NCOMMS-24-26940B
Last updated by author(s): 2025/08/20

Reporting Summary

Nature Portfolio wishes to improve the reproducibility of the work that we publish. This form provides structure for consistency and transparency in reporting. For further information on Nature Portfolio policies, see our Editorial Policies and the Editorial Policy Checklist.

Statistics

For all statistical analyses, confirm that the following items are present in the figure legend, table legend, main text, or Methods section.

n/a	Confirmed	
☐	☒	The exact sample size (*n*) for each experimental group/condition, given as a discrete number and unit of measurement
☐	☒	A statement on whether measurements were taken from distinct samples or whether the same sample was measured repeatedly
☐	☒	The statistical test(s) used AND whether they are one- or two-sided<br>*Only common tests should be described solely by name; describe more complex techniques in the Methods section.*
☐	☒	A description of all covariates tested
☐	☒	A description of any assumptions or corrections, such as tests of normality and adjustment for multiple comparisons
☐	☒	A full description of the statistical parameters including central tendency (e.g. means) or other basic estimates (e.g. regression coefficient) AND variation (e.g. standard deviation) or associated estimates of uncertainty (e.g. confidence intervals)
☐	☒	For null hypothesis testing, the test statistic (e.g. *F*, *t*, *r*) with confidence intervals, effect sizes, degrees of freedom and *P* value noted<br>*Give P values as exact values whenever suitable.*
☒	☐	For Bayesian analysis, information on the choice of priors and Markov chain Monte Carlo settings
☒	☐	For hierarchical and complex designs, identification of the appropriate level for tests and full reporting of outcomes
☒	☐	Estimates of effect sizes (e.g. Cohen's *d*, Pearson's *r*), indicating how they were calculated

Our web collection on statistics for biologists contains articles on many of the points above.

Software and code

Policy information about availability of computer code

Data collection	Sequencing reads were aligned to the GRCh38 reference genome, using BWA-MEM75 and PCR duplicates from the Binary Alignment Map (BAM) file were marked using the samtools (v1.14) markdup function with parameters –mode s –S –include-fails. Matched tumour-normal sample concordance, as well as cross-individual contamination, was assessed using Conpair (v0.2). Reads were aligned using STAR (v2.5.0c15) against the GRCh38 human reference genome using Ensembl release v103 gene annotations. Expression levels were assessed by counting reads using HTseq (v0.7.2) with the appropriate stranded parameter and subsequently transformed into transcripts per million (TPM) values. Data quality was assessed by running RNA-SeqQC and assessing the total number of counts obtained per sample.
Data analysis	Somatic point mutations were identified using cgpCaVEMan (v1.15.2). The parameters used and input files are described in the Supplementary Methods. Variant flagging and annotation were not performed initially. Instead, adjacent, in cis SNVs called with cgpCaVEMan were evaluated using SmartPhase (v1.2.1) and casmsmartphase (v0.1.8; https://github.com/cancerit/CASM-Smart-Phase) to identify MNVs. Variants were then flagged using the cgpcavemanpostprocessing (v1.10) cgpFlagCaVEMan.pl utility using the 'WXS' mode for exomes. The parameters and flagging rules used are described in the Supplementary Methods.<br><br>Indels on the autosomes and chromosomes X and Y were identified using cgpPindel (v.3.10.0). A simple repeats file for GRCh38, generated using the UCSC Table Browser, and a list of regions to exclude due excessive high depth of coverage, were used as inputs. Soft flag FF017 was used, but variants were not hard filtered based on this flag. Additional details, including a description of parameters, flags and input files, are available in the Supplementary Methods.<br><br>SCNAs were identified using ASCAT (v3.1.2).<br><br>To find significant recurrent amplifications and deletions, the outputs from ASCAT were used to generate input files for GISTIC2 (v2.0.23).

dNdscv (v0.1.0; git commit ID 64f8443) and OncodriveFML (v 2.4.0).

To identify somatic mutational signatures for single base substitutions, doublet base substitutions and indels, somatic mutations were analysed using SigProfilerExtractor (v1.1.21) and SigProfilerAssignment.

DISCOVER (r_v0.9.4).

Genome Analysis Toolkit (GATK) Best Practices workflow.

We projected the germline single nucleotide polymorphisms from the whole exome sequencing normal samples into the principal component space of the 1000 Genomes (1000G) project69 using the package AKT v0.3.3.

STAR-Fusion (v1.10.1), with STAR (v2.78a) aligner and the Trinity Cancer Transcriptome Analysis Toolkit (CTAT) genome library StarFv1.10 for GRCh38 using GENCODE v37 (Ensembl v103) gene.

The Immune Estimation module of TIMER2.0 (Tumour Immune Estimation Resource version 2)73 for immune infiltration estimation was run on the tumour samples.

MOFA was used to fit a regularized latent variable model-based clustering.

Class-I and -II haplotyping and neoantigen prediction were performed using the nextNEOpi nextflow pipeline.

Screening for pathogens, Kraken2 v2.1.2 was tested on tumour samples.

Hippo and Yap1 pathway activations were computed based on the REACTOME definitions downloaded from the Molecular Signatures Database using Gene Set Enrichment Analysis with the R package gsva.

For manuscripts utilizing custom algorithms or software that are central to the research but not yet described in published literature, software must be made available to editors and reviewers. We strongly encourage code deposition in a community repository (e.g. GitHub). See the Nature Portfolio guidelines for submitting code & software for further information.

Data

Policy information about availability of data

All manuscripts must include a data availability statement. This statement should provide the following information, where applicable:

- Accession codes, unique identifiers, or web links for publicly available datasets
- A description of any restrictions on data availability
- For clinical datasets or third party data, please ensure that the statement adheres to our policy

Sequencing data are available from the European Genome-Phenome Archive (EGA) under dataset accessions EGAD00001015367 (DNA) (https://ega-archive.org/datasets/EGAD00001015367) and EGAD00001015368 (RNA) (https://ega-archive.org/studies/EGAS00001003553).

Research involving human participants, their data, or biological material

Policy information about studies with human participants or human data. See also policy information about sex, gender (identity/presentation), and sexual orientation and race, ethnicity and racism.

Reporting on sex and gender	We indicate if samples were obtained from individuals who were biologically male or female at birth.
Reporting on race, ethnicity, or other socially relevant groupings	We don't report on race.<br>However, we indicate where the patients came from and made an ancestry analysis.
Population characteristics	Our samples were collected worldwide/mainly in Europe/Japon/Canada.
Recruitment	We collected formalin-fixed paraffin-embedded (FFPE) samples from 222 patients, ascertained from eleven institutions across six countries. These countries are provided in the supplementary Data. We did not exclude any centres or samples unless they were analysed and found to not be sebaceous tumours (except SH and KA).
Ethics oversight	We collected formalin-fixed, paraffin-embedded (FFPE) samples from 222 patients, ascertained from eleven institutions across six countries. Ethical approval for the use of all patient samples was obtained by a local committee at the institution of origin and also via Research Governance at the Wellcome Sanger Institute.

Note that full information on the approval of the study protocol must also be provided in the manuscript.

Field-specific reporting

Please select the one below that is the best fit for your research. If you are not sure, read the appropriate sections before making your selection.

- [x] Life sciences
- [ ] Behavioural & social sciences
- [ ] Ecological, evolutionary & environmental sciences

For a reference copy of the document with all sections, see nature.com/documents/nr-reporting-summary-flat.pdf

Life sciences study design

All studies must disclose on these points even when the disclosure is negative.

Sample size	We analysed all samples we could obtain and successfully sequence of these exceeding rare conditions. After central pathology review some samples were found to be other entities, which we excluded.
Data exclusions	No samples were excluded after having passed sample QC and sequencing.
Replication	Replication was not required as part of the study design.
Randomization	Randomization was not required as part of the study design.
Blinding	Blinding was not required as part of the study design.

Reporting for specific materials, systems and methods

We require information from authors about some types of materials, experimental systems and methods used in many studies. Here, indicate whether each material, system or method listed is relevant to your study. If you are not sure if a list item applies to your research, read the appropriate section before selecting a response.

Materials & experimental systems

n/a	Involved in the study	
x		Antibodies
x		Eukaryotic cell lines
x		Palaeontology and archaeology
x		Animals and other organisms
x		Clinical data
x		Dual use research of concern
x		Plants

Methods

n/a	Involved in the study	
x		ChIP-seq
x		Flow cytometry
x		MRI-based neuroimaging

Plants

Seed stocks	N/A
Novel plant genotypes	N/A
Authentication	N/A
