# Supplementary material for: The molecular cartography of malignant and benign sebaceous tumours
Source: Nat Commun. 2025 Dec 19;17:14. doi: 10.1038/s41467-025-66584-0 (PMC12764876; doi:10.1038/s41467-025-66584-0)
Supplement: Supplementary file 4 — Description of Additional Supplementary Files [file 41467_2025_66584_MOESM4_ESM.pdf]

Description of Additional Supplementary Files

File name: Supplementary Data 1

Description: Clinical Data.

File name: Supplementary Data 2

Description: Sebaceous tumours TMB versus other datasets.

File name: Supplementary Data 3

Description: Tumour mutational burden (TMB) and indel rates for each of the sebaceous tumour subtypes.

File name: Supplementary Data 4

Description: Somatic short indels, single and multi-nucleotide variants (SNVs and MNVs) mutations in the four sebaceous tumour subtypes and associated lesions.

File name: Supplementary Data 5

Description: Driver gene analysis of the four sebaceous tumour subtypes.

File name: Supplementary Data 6

Description: DISCOVER analysis: mutual exclusive and co-occuring interactions in sebaceous tumours.

File name: Supplementary Data 7

Description: Results of the integrative clustering analysis of the sebaceous tumours.

File name: Supplementary Data 8

Description: Mutational signatures in sebaceous tumours.

File name: Supplementary Data 9

Description: Copy neutral loss of heterozygosity (cnLOH) in sebaceous tumours.

File name: Supplementary Data 10

Description: GISTIC2 – Focal deleted/amplified regions by subtype in sebaceous tumours.

File name: Supplementary Data 11

Description: GISTIC2 – Broad deleted/amplified regions by subtype in sebaceous tumours.

File name: Supplementary Data 12

Description:

File name: Supplementary Data 13

Description: Viral sequence data in sebaceous tumours.

File name: Supplementary Data 14

Description: Fusion genes in sebaceous tumours.

File name: Supplementary Data 15

Description: Neoantigens in sebaceous tumours.
