# Supplementary material for: The molecular cartography of malignant and benign sebaceous tumours
Source: Nat Commun. 2025 Dec 19;17:14. doi: 10.1038/s41467-025-66584-0 (PMC12764876; doi:10.1038/s41467-025-66584-0)
Supplement: Supplementary file 2 — Transparent Peer Review file [file 41467_2025_66584_MOESM2_ESM.pdf]

nature portfolio

Peer Review File

The molecular cartography of malignant and benign sebaceous tumours

Corresponding Author: Dr David Adams

This file contains all reviewer reports in order by version, followed by all author rebuttals in order by version.

Version 0:

Reviewer comments:

Reviewer #1

(Remarks to the Author)
This is a large and comprehensive study of genomic alterations in sebaceous neoplasms, reaching from benign to malignant tumors. The data generated represents a considerable resource, which is important for the scientific community. While the scope of the study and the number of samples is impressive, there are several significant weaknesses. Overall, it is not fully clear what novel aspects of these tumors has been revealed. The 5 mutational patterns are not fully convincing and the analysis of copy number changes and structural rearrangements not compelling as presented.
The manuscript is difficult to read with many nested sentences full of abbreviations and not always clear meaning and partially problematic grammar. More importantly, no coherent picture is distilled by integrating the multi-omic data to extend the knowledge on the biology of these tumors, i.e. what signaling pathways are consistently altered, what constitutes the barrier between benign and malignant, and how any of these features might relate to clinical course. Outcome data is mentioned to exist but does not seem to feature in the analysis.

Specific points:

1) Figure 1B y-axis labels should be clarified. What additional tumors qualify? Other STs. Could be phrased more clearly and succinctly.
2) Figure 1C is not referenced in the text and the labels are not clear. The pictures are clinical and histological and not clinicopathological
3) It would be good to show a lollipop diagram for RREB1 as well. Is there any information that can be gleaned about the functional relationship between RREB1 and HRAS mutations? For example, are particular types of RREB1 mutations associated with HRAS mutations? What are the anticipated functional consequences of RREB1 mutations?
4) The last sentence on mutually exclusivity is difficult to parse and the pattern of exclusivity not apparent (to me) from figure 2. It looks like many tumors with TP53 mutations also have mutations in RREB1, HRAS, or NOTCH1
5) The data on copy number changes implicating individual genes is not compelling. GISTIC scores in fig 5 looks noisy rather than showing bona fide copy number changes. This is particularly true for SA and SM, which seem to have overall ‘flat’ genomes. The sentence on page 8 line 342 onwards on amplifications of specific genes cannot be understood.
Also, what constitutes a ‘significantly focal amplification’? Compelling examples of the raw copy number data for example for HRNR and KIT amplifications should be shown for this section to be more compelling, ideally with additional supporting information nominating these genes (FISH, IHC). A better integration with mutation data is desirable when nominating specific genes based on copy number change. E.g. how would an amplification of GNAS be plausible in this tumor type, in which no activating mutations of GNAS are seemingly present?
The wording and grammar in these paragraphs is awkward e.g. ‘In contrast, SC-Es showed significantly chromosomes 1 and 20 amplifications, and significantly 17p deletions, which includes TP53 (7/36 tumours, of which 6 also had a somatic TP53 mutations’
6) Additional consideration and discussion should be given to the PAK2 fusions. The fusions with PAK2 at the 5’ end appear to be novel/unprecedented. Is there a reciprocal fusion product that was perhaps missed?
7) The finding of an identical fusion of RCOR1::GRHL2 needs additional discussion as it implies a clonal relationship between the two SAs of this patient.
8) The finding on CIN primarily in pMMR tumors is interesting and mirrors the findings in CRC. This indicates, as you state, that genomic instability is necessary for the malignant transformation. Any insights on why and how?

Reviewer #2

(Remarks to the Author)
General comments:

In this multi-center international study, Ferreira et al. present the largest exome and transcriptome analysis of sebaceous tumors (ST) to date. This allowed investigators to identify mutated genes under positive selection (drivers) for the first time in this tumor type, which provides an incredibly valuable resource to the cancer research community. In my opinion, this is an extremely important study for the ST and cancer genomics fields.

The initial data processing, including variant calling, mutation signature extraction, and copy number profiling, was conducted using well-established software and pipelines. However, several downstream analyses are problematic, and the presentation of the results can be significantly improved to enhance clarity and better guide the reader. Furthermore, in my opinion, parts of the neoantigen and ligand-receptor analyses are too speculative, and they lack additional data or experimentation for validation. These sections should be modified for clarity and rigor, and their results presented more cautiously or the authors may want to consider omitting these analyses from the study.

My recommendation is to improve the whole exome and transcriptome analysis as outlined in detailed below and re-consider inclusion of the ligand-receptor analysis and parts of the neoantigen analysis.

Concerns/suggested revisions:

Somatic landscape of sebaceous tumours
Driver Identification Analysis:

[1] The paper reports significantly mutated genes based on a single software tool (dNdScv), without the necessary validation steps (e.g. filtering for genes that are not expressed or have low VAF), raising concerns about false positives that are ubiquitous in the context of high TMB tumors. (For example, SPANXB1 is reported as a driver in Figure 2, but most mutations in this gene appear to have a suspiciously low VAF). To ensure robustness, the authors should filter their results to exclude likely false positives and, importantly, provide additional supporting evidence for the novel candidate drivers:
● What is the variant allele frequency (VAF) distribution for each gene?
● Do these variants mostly originate from a specific signature or positional site?
● What proportion of variants are seen in the RNA-Seq data for each gene?
● Are there significant co-occurrences of loss-of-function mutations with loss of heterozygosity (LOH) for the putative tumor suppressor genes (TSGs)?

[1.1] The authors of the dNdScv software have excluded hypermutator samples (>500 mutations) from their main analysis (iMartincorena et al, 2017, PMID:29056346). Such precautions are not mentioned in the method section of the current manuscript. Did they authors try running dNdScv exclusively on tumors with fewer than, say, 1000 or 2000 mutations to see how it affects the results?

[1.2] There are 24 samples with no matched normal. It is important to exclude these samples from the dNdScv analysis as they could contain germline variants misclassified as somatic (I’ve noticed VAF modes near 0.5 for many of these samples). Could the authors specify which samples have been included in their analyses, along with any exclusion criteria?

[1.3] Possible additional figures for the driver analysis (in main or supplementary): A barplot showing the relative proportion of each variant class (missense, frameshift stop gain etc) in each gene or selected genes. A scatter plot showing dN/dS:missense vs dN/dS:Lof similar to Figure 2B in Martincorena et al.(2017, PMID: 29056346). These figures would help support the analysis results and importantly inform about the action mode of the candidate drivers (i.e. oncogene vs.TSG).

[1.4] It would be helpful if the authors cross-reference their gene list with the results of some pan-cancer analyses, such as Bailey et al. (2018, PMID: 29625053) or Martincorena et al. (2017, PMID: 29056346). This information could be easily summarized in a supplementary figure having genes as rows and cancer types as columns, with each tile colored according to their report status (e.g. oncogene, potential oncogene, tsg and potential tsg, which is available in Supplementary Table 1 of Bailey et al.). It would be useful for the readers for genes that haven’t been reported as significantly mutated, what does the literature say about them that could support their role as an oncogene or tumor suppressor?

[1.5] I speculate the authors may have missed the identification of several drivers by testing all genes as they did impose a burden on significance levels after multiple testing corrections. In addition to their global analysis, I think it may be worth performing a restricted hypothesis testing (RHT), such as in Lawrence et al. (2014, PMID: 24390350) by considering only previously reported genes, for example those from Bailey et al. (2018), and those reported as drivers in sebaceous tumors. This additional analysis would be straightforward, requiring only the subsetting of the global results to these genes before applying multiple testing corrections. This may be valuable to the readers.

[1.6] The main oncoplot could be enhanced in several ways.
• Adding copy number track for the most frequently affected genes (deleted TSG or amplified oncogenes).

• Some drivers with less supporting evidence or of lower interest could be removed from the main figure for simplicity and clarity (but shown in a full supplementary oncoplot).
• Annotation tracks with tumor purity, RNA subtypes and mutational signature groups could be added.
• Mode of action of each driver (TSG vs oncogene) could be highlighted.

[1.7] The authors only report the gene names with their corrected p-values in the supplementary material. It is important to include the complete dNdScv output in the supplementary material to provide a comprehensive view of the analysis and facilitate further validation and reproducibility of results.

[1.8] On lines 199-200, the authors write, "Interestingly, when considering the ST subtypes individually, HRAS was only identified as a driver gene in the benign tumors." What is the uncorrected p-value for HRAS in the SC-E and SC-O subgroup analyses? Would it pass RHT? Even if not significant, the oncoplot shows several HRAS mutations in SC-E and SC-O, most of which are known activating mutations at G12/13 and Q61. Moreover, HRAS harbors no silent mutations in these tumors, strongly suggesting it is an actual driver, albeit less frequently mutated. Accordingly, it would be more appropriate to drop this statement or at least revise it to "HRAS was not reported as significant in (...) probably due to its lower mutation frequency and the smaller size of these groups (...)," and mention the presence of these hotspot mutations. In general, the absence of a significant result for a particular gene in a subgroup analysis cannot be used on its own to suggest an absence of “driver” activity.

[1.9] In the discussion, lines 491-492, the authors state: “Use of somatic mutational profiling may help in the differential diagnosis of benign versus malignant STs, which can be challenging.” It would be helpful if more clinical context could be provided here, particularly around the standard histopathological diagnostic workflow. What are the gold-standard IHC stains, histopathological correlates, or clinical decision algorithms currently used to differentiate between the four types of sebaceous tumors? A deeper discussion on how mutational testing could provide clinical input would be ideal, given the clinical correlation angle in the discussion. Figure 2 does not clearly identify a mutational profile with high sensitivity or specificity for diagnosis. Could the authors consider using classifiers (e.g., Random Forests) and measure their performance with cross-validation?

Mutually exclusive and co-occurring mutations:

[2] The “mutual exclusions and co-occurring interactions” analysis is problematic as it combines the results of a Fisher’s test with the output of DISCOVER, a tool specifically developed to replace the inappropriate usage of the Fisher’s test in such analyses. The Fisher’s test does not control for TMB, leading to a high number of false positive co-occurring interactions (as shown in Figure 2B). In contrast, DISCOVER accounts for TMB in each sample and gene, thus limiting these spurious results. Combining the results from both approaches does not make sense, as exemplified by the contradictions between the interactions reported in Figure 2B and the DISCOVER analysis output provided in Supplementary Table 6 (e.g., significant co-occurrence between ZNF750 and BICRA while Supplementary Table 6 reports significant mutual exclusion). I would suggest that the authors should rely solely on DISCOVER (or similar approaches controlling for TMB), and completely exclude the Fisher’s test from this analysis.

Mutational signatures of sebaceous tumours:

[3] The mutational signature analysis was not comprehensively explored and presented, and a global view of the cohort is lacking, specifically related to the relative contribution of the main signatures in each sample. The presentation of the results in Figure S5 show too many signatures simultaneously, and additional figures and analyses would be necessary to support the claims and observations made in the main text and discussion. In my opinion, an improved global view of the mutational signatures would warrant inclusion as a main figure.

[3.1] Figure S5 needs clarification. The graphical legend mentions */PD A, */PD NA, and */PD NV in three different colors, but none of these are explained in the textual legend. There is a reference to the blue and red labeling of the sample names, but this is confusing since "blue" samples (no significant signature) actually show some signatures. Additionally, the legend states that "one in red text was not included," but half of the samples are in red.

[3.2] The method section states that SigProfilerExtractor was used for de novo extraction, considering solutions with 1 to 10 signatures by NMF. However, Supplementary Figure 5 shows 21 signatures (17 SBS, 3 ID, and 1 DBS), and Supplementary Table 8 reports mutation counts for 27 signatures. This discrepancy needs clarification. It is essential to explain the criteria for determining the final set of signatures and how they relate to the initial NMF analysis to ensure consistency and transparency.

[3.3] I strongly suggest the authors include a main figure summarizing the mutational signature analysis. For example, a clustered heatmap of each signature log10 counts (as rows) in each sample (columns) clearly shows the 3 main mutational profiles within this cohort: MMR (SBS15 + ID2 + ID7 + high SBS1 + high SBS5), UV (DBS1 + SBS7a + SBS7b + low SBS1 + low SBS5) and others (low SBS1 + low SBS5). Annotation tracks for samples could include the MMR status, log10TMB, diagnosis and presence/absence of LoF mutations in MSH2 & MSH6. An annotation track for the signatures could inform the reader about their aetiology (UV, MMR etc). Such heatmap can be easily produced with R’s pheatmap package and the ward.D2 clustering method. To make it more concise and suitable for a main figure, some highly correlated or same aetiology signatures could be merged (e.g. SBS7a & SBS7b) and low contributing signatures could be dropped and deferred to supplementary figures (if necessary). Alternatively, the authors could use the W matrix from NMF or apply PCA on the 96-SBS mutation proportion matrix to create a scatter plot of all samples colored by cancer types, MMR status and

other variables of interest.

[3.4] Lines 268-269, the authors write, “As expected, in the dMMR cases, the high TMB was attributed to dMMR-associated mutational signatures.” What proportion of TMB is attributed to dMMR signatures? Could the authors please include a figure supporting this? It seems that other signatures (SBS1 & SBS5) contribute more to TMB than dMMR-associated signatures, even in dMMR samples.

[3.5] Lines 163-166, the authors write, “Interestingly, indel rate rather than TMB, more accurately predicted MMR status (indel rate p-value < 2.2e-16 versus TMB rate p-value = 2.203e-08, Welch two samples t-test)”. I have a few concerns regarding this analysis. First, the considered data doesn’t meet this test's assumptions about normality. Second, p-values are not an appropriate measure of prediction accuracy. Third, according to the methods section, MMR status was in part determined by the indel rate (lines 772-774), which means using the indel rate to predict MMR status is circular reasoning. These issues should be addressed, for example by comparing ROC-AUCs and and an MMR classification that doesn’t rely on indel rate or TMB.

[3.6] On lines 501-513, the authors write “this study highlights the importance of recording the precise location of SC-O tumours, and specifically restricting use of the term SC-O for only those arising on the eyelid. Indeed, the sample coming from the canthus showed a different phenotype from the other SC-O, being characterised by UV mutational signatures rather than being pauci-mutated”. Could the authors please include a figure and additional analyses or references supporting this claim, for example by showing differences in mutated drivers or gene expression that suggest it is more similar to SC-E than SC-O?

[3.7] Lines 1377-1379: “SBS” should be “DBS”. Also, a typo in “mutationa”.

[3.8] In some other cancers, MMR status is linked to survival (e.g. Endometrial). Did the authors investigate whether in this cohort, MMR status is linked to survival in SC regardless of subtype? Authors note that sample size in SC was too small to measure but it may be helpful for readers to see this information anyways even if not statistically significant.

Integrative cluster analysis:

[4] The results of this analysis are rather cryptic, and the methodological details raise concerns about their validity. First, the expression data was combined with the mutation profiles of approximately 80 genes selected on the basis that they are frequently mutated in this cohort. Given that a typical tumor contains fewer than 10-15 driver mutations (referring here to Martincorena et al, 2017) and that these sebaceous tumors have huge TMBs (nearly half of them have a thousand mutations or more), we can be sure that most of the mutations in these 80 genes are passenger mutations. While a few relevant genes such as TP53 and MSH2 have been manually added and others like TTN have been manually removed, Supplementary Figure 4 suggests that the limited number of potentially meaningful mutations is still overwhelmed by the number of passengers.
Second, the method contains many steps with somehow arbitrary choices (e.g., a first selection step out of 16 fitted models followed by a second selection step for the number of k-means clusters), making it very difficult to assess the robustness of the findings. What happens if we use another fitted model (many had a similar balance of variance explained and a low number of clusters)? What happens if a different k is chosen or if we change the gene sets?
I should note, even if the authors are able to address the concerns described above, and although I appreciate these exploratory types of integrative analysis, I would argue they cannot replace the more well-established analyses that many readers would expect, if only for clarity and easier interpretation of the results. In other words:
[4.1] How many (purely) transcriptional groups seem to be there? If the exact number is hard to determine, the smallest number that make sense (visually) should be the starting point.
[4.2] Once these groups have been identified, do they correspond to the ST subtypes (SA vs. SC-E vs. SC-O)?
[4.3] Are they enriched for specific mutations or CNAs?
[4.4] Which immune and other non -tumor cell types are present in each of these bulk tumors? How does this correlate with tumor purity?
[4.5] Which pathways or genes seems differentially expressed between these groups? Could this be confounded by tumor purity or non-tumor cells or is there evidence that they could represent genuine differences between the cancerous or neoplastic cells?

Clinical data:

[5] Supplementary Table 1 is a somewhat ambiguous and includes a mix of patient-related and sample-related data with several issues. I recommend that the authors provide two separate tables: one for the patient-related data and one for the sample-related data, each with appropriate IDs (patient and sample).

[5.1] Some fields lack sufficient description in the legend, such as "Age," which does not specify whether it refers to the age at diagnosis or at sample procurement. Similarly, “Recurrence”, “TimeAfterInitialDiagnosis_Rec(months)”, “Metastasis” and “TimeAfterInitialDiagnosis_Met(months)” also needs some explanation.

[5.2] Column AE has no name. It contains “Father, Brother” for sample “PD42477a”, but “FamilyCancerLynch” says “no” for this sample.

[5.3] Some field names differ between the table and the legend. For example, "MMRstatus" is mentioned in the legend, but

"MMR.d.MMR.p_simplified" appears in the table. Same for some IHC fields.

[5.4] The MMR status is not written correctly for PD42171a (MMR-p instead of pMMR).

[5.5] For "MLH1Hypermathylation," a mix of blank and "not tested" is used. Please use a unique value (either blank or "not tested") for all cases to maintain consistency.

[5.6] Typos: “Hypermathylation”, “Supplemntary”

[5.7] There are a number of issues in this table. It would be ideal to ensure that all fields used in the analyses are clearly documented and are error-free. I would recommend fields that cannot be used in any reasonable analyses, because of incomplete or ambiguous data, should be dropped.

Germline alleles in sebaceous tumour patients:

[6.1] This section could be further developed. Associated Figure 6 contains 27 genes, but only a handful of these genes are mentioned in the text. I would recommend improving figure (e.g., removing genes not discussed and adding other plots showing how mutations correlate with phenotypes) or moving it to a Supplementary Figure.

[6.2] VAF and other important information is missing from Supp. Table 12. While the method section states that germline variants were identified with GATK HaplotypeCaller and GenotypeGVCFs, Supp. Table 12 includes fields such as “Tumor_Seq_Allele2” which are typically associated with somatic variant calling. Could the authors clarify this?

Copy number landscape of sebaceous tumours:

[7.1] This section could be better developed. It would be ideal if both Figures 4 and 5 could be simplified to include only the key panels that are clearly highlighted to emphasize points made in the main text.

Neoantigen and ICI response prediction:

[8] The last section of the paper (lines 413-482) reports results based on a number of software tools (CODEFACS, TIMER2.0, EPIC, LIRICS, CIBERSORT, pVACseq, and others) and several external datasets that I evaluate as highly speculative. There are insufficient details provided to properly understand the methodology and the results, which are supported by a single, not particularly informative, main figure. At this stage, I would recommend removing this section to ensure clarity and rigor of the whole exome and transcriptome analysis of the paper, or significantly revise this section with more clear details on how the analysis was performed. However, I should note I anticipate the results will likely still be speculative and omission of the ligand-receptor analysis should be considered if validation by IHC/IF staining studies are not being included.

Other:

[9.1] There is some inconsistent naming and abbreviation usage for the tumor subtypes across the main text, figures, and their legends. For instance, "SM" is used for sebaceoma in Figure 1 and Table S1, while only "S" is used in Figure 3 and S5. Similarly, "PeriOcular," "Peri-ocular," and "ocular" are all used to refer to ocular sebaceous carcinoma. Please standardize the terminology and abbreviations for each disease subtype.

[9.2] According to ST1, sample PD43886c and PD48829c have been used in DNA analysis (DNA_Analysis == Yes). However, I couldn’t find any mutations associated with these samples in ST4.

[9.3] SF1A has no legend describing the meaning of each color and does not seem appropriate to clearly support the claims.

[9.4] The cohort size is relatively large, considering the rarity of these tumors. However the SC sub-group is quite small, particularly the SC-O subgroup which may limit the power of the analyses. More discussion of this (and other limitations) would be appreciated in a short, dedicated section.

[9.5] Suggestion: Is it necessary to note that no recurrence or metastasis arose in SA or SM (Line.149) as these are benign tumors that do not usually (or at all?) metastasize. If they do, this should be noted in the text.

[9.6] Suggestion: It would be ideal to list the 6 countries that these tumors come from (line 81) and mention in discussion any potential biases from tumor selection based on ethnicity or geographical location – for example, authors mention that SC-O is more common in people of Asian descent (40-60% of eyelid tumors). Have the authors investigated the ethnic makeup of their cohort? Is it representative of the global or regional prevalence/distribution of these tumor subtypes? If not, how might this affect the results of their study?

Reviewer #3

(Remarks to the Author)
Authors investigated the genetics and mutational signatures of sebaceous tumors and reached into the conclusion that there are multiple subtypes present. The study also has an interesting observation that some of these tumors also have lynch

signatures. The lynch signatures should be investigated further using current non tumor and historical patient samples to reach a definitive conclusion that these mutations were not acquired later in development. Utilizing some published material to support Sebaceous tumor prevalence in Lynch syndrome would also be informative. Authors utilize available pipelines to perform a lot of their computational analysis, it would be helpful if same pipelines are used on some examples of published historical skin tumors such as Melanoma, squamous carcinoma, UV mediated Merkel cell carcinoma for comparison in these figures such that the reader could make comparisons of mutational burden etc. these results may be skewed by the pipelines used. Authors make some suggestions about the immunogenicity of RCC tumors, this is still currently a puzzle since we don't know what makes RCC tumors highly immunogenic. First the Mutational burden for sure does not explain RCC immunogenicity and secondly the CD8 T cell infiltration in RCC tumors is actually deleterious. So the discussion and comparison to these tumors would need to be carefully worded and considered in the text. Authors make conclusions about the immunogenicity of the tumors. This part requires additional work. First the authors again utilize some pipeline to do their analysis, this would be valid if there are some controls presented for comparison. So the Neoantigen score for Melanomas, squamous carcinomas and UV induced merkel cell carcinoma utilizing published data should be presented as a control comparison. Additionally the authors present histology data in figure 1. As a supplement an immune score calculation utilizing CD4/CD8 infiltration should be presented for each tumor since the authors have access to FFPE blocks. This would make the interpretation of the all neoantigen loads the authors are presenting and would have to validate some of the observations

Version 1:

Reviewer comments:

Reviewer #1

(Remarks to the Author)
The authors did a thorough job in addressing the reviewers' concerns and the manuscript has been significantly improved. My comments have been adequately addressed.

Reviewer #2

(Remarks to the Author)
The authors’ have made considerable efforts to revise their manuscript and respond to suggested revisions and comments. However, two key issues have not been appropriately addressed and require further attention.

1. Driver Gene Analysis & False Positives

In the initial review, concerns were raised about the likely high proportion of false positives due to the authors' reliance on a single tool (dNdScv) without proper filtering or orthogonal validation. Specifically, SPANXB1 was highlighted as an example of a likely false positive due to the low VAF of its mutations. In response, the authors argue that, based on the interquartile range method, SPANXB1’s VAF is not an outlier. Unfortunately, the authors response misses the point of the major concern raised—that further examination of the significantly mutated gene list is needed to identify and remove/flag likely false positives before publication. For example, in re-analyzing the data provided by the authors, 85% of SPANXB1 mutations have a VAF < 0.25, which is directly at odds with their own Figure R8, where they claim LOH is observed in nearly 70% of cases. Additionally, it is important to note that SPANXB1 is located on the X chromosome, and most of its reported mutations occur in males—where there is no heterozygosity to lose. This raises concerns regarding the LOH claim in Figure R8 and the robustness of the significant mutated gene list.

Importantly, the suggested additional analyses —VAF distribution, mutation signatures, gene-level RNA-Seq validation (e.g. RNA-VAF vs DNA-VAF), and co-occurrence of LOH with loss-of-function mutations —were essential recommendations for validation/filtering steps to refine the driver mutation gene list before publication (i.e. identify and remove/flag the likely false positives). These analyses should be included in the paper to demonstrate that the final significantly mutated gene list is robust and biologically meaningful.

One last additional point, when showing the distribution of mutation consequences by gene (Figure R9), synonymous mutations must be included, as they are essential for interpreting selection patterns (e.g. a high proportion of synonymous mutations suggests weak or no selection).

2. Integrative Clustering Analysis Based on Unreliable Data

Another major concern was that the integrative clustering analysis relies on mutation data dominated by passenger mutations (not just a few as claimed by the authors). The authors did perform a more rigorous expression-based analysis for in their revised manuscript. However, instead of highlighting this new and improved analysis, they relegated it to the supplementary material, while keeping the original problematic figure in the main text. I suggest they do the opposite: put their new analysis in the main text and relegate the one based on passenger mutations in the supp mat (or consider discarding it).

Conclusion

We encourage the authors to directly address our original concerns and incorporate the necessary analyses to ensure their findings are well-supported.

Reviewer #4

(Remarks to the Author)
I believe the authors have appropriately addressed all of the valid concerns raised by Reviewer #3. Regarding the previous question about 'these results may be skewed by the pipelines used' and whether 'it would be helpful if the same pipelines were applied to some examples of published historical skin tumors,' while this is a valid concern regarding potential bias arising from differences in bioinformatics pipelines, however, re-analyzing the data using the same pipeline would not eliminate systematic biases between studies. These biases also arise from variations in sequencing methods and sample preparation protocols used by different research institutes. Additionally, publicly available datasets, such as MSK-IMPACT, utilize custom panels with different targeted regions, re-analyzing these datasets using the same bioinformatics pipeline would not resolve the inherent systematic differences.

Version 2:

Reviewer comments:

Reviewer #2

(Remarks to the Author)
Despite previous requests for more robust validation of the nominated drivers, the authors persist with their original list of 28 drivers, relying solely on the outputs from dNdScv. While dNdScv is effective, it is prone to reporting false positives especially in high TMB contexts. This is concerning as many of the nominated drivers are not established cancer-associated genes or have not been identified in large pan-cancer driver mutation analysis (for example nearly half (13/28) not recognized in a characterization of cancer driver genes in the TCGA PanCancer analysis by Bailey et al). The authors do mention using oncodriveFML as a complementary tool in their methods section, yet these results are never discussed in the manuscript, nor are they integrated with those from dNdScv. The raw dNdScv output is still not included in this revision as originally requested

Despite clear evidence that indicates SPANXB1 is a false positive, the authors continue to nominate it as a driver gene. They incorrectly attribute the unusually low variant allele frequency (VAF) of its mutations to its location on the X chromosome in their revised manuscript. Indeed, VAF of mutations on the X chromosome should be twice that of heterozygous mutations on autosomes in males.

In Supplementary Table 5B, where the results of a driver analysis were supposedly restricted to drivers identified by Bailey et al., genes such as TTN, OBSCN, and NEB with extremely low p-values were included. These genes are known for accumulating passenger mutations due to their length and are not listed among the drivers in Bailey et al. study. This discrepancy indicates that either the analysis was not conducted properly or the incorrect results were presented.

Fig S4A contains obvious errors. How could 676 SNV mutations be observed with >10X coverage if the total number of SNV mutations is 667? The authors also missed the point since the idea here was to show that most mutations in *each* of the nominated drivers are observed in the RNA-seq, in contrast to passenger mutations in other genes that are often not expressed. As is, the table does not inform about the individual plausibility of the nominated drivers. Figure S4C and S4E also miss the point and do not inform the plausibility of the nominated drivers. The idea was to show if mutations often co-occur with cnLOH or cnLoss in nominated TSGs (and hence have higher VAF), in contrast to passenger mutations in *other* genes, as would be expected. Additionally, in Figures S4B and S4C, the y-axis is inaccurately labeled as 'count'.

The purpose of these additional analyses we requested was to identify which candidate drivers are substantiated by orthogonal evidence and to pinpoint likely false positives. Unfortunately, the authors chose to present inconsequential data and assert that their initial list of drivers remains valid without requiring any modifications.

I do still think this is an important resource paper to the cancer genomics community.

Reviewer #5

(Remarks to the Author)
Dear Dr McGinnis.

I have gone through the questions from Reviewer #2 and the authors' responses. Although some of the points are addressed in detail, there are still some points that require clarification.

a) In the author's response, it was mentioned that 15 out of 28 predicted drivers are present in the TCGA PanCancer analysis by Bailey et al., and the remaining ones are linked to tumorigenesis based on literature evidence. I think this is informative, and the authors may consider providing this information in the supplementary table and also mentioning this overlap number in the results section.

b) The results of restricted hypothesis testing using Bailey et al., (299 cancer driver genes) reported in Supplementary Table

5B still show genes such as TTN, OBSCN, and NEB, which are likely false positives. The authors stated that they were removed, but they're not reflected in the table yet.

c) The results of oncodriveFML were not directly included in the supplementary table, so it is difficult to compare the results. The GitHub link to the oncodriveFML output is also not working. It would be helpful for the readers to know at least what fraction of the 28 potential drivers predicted by the dNdScv overlap with the oncodriveFML significant hits. In particular, whether the four drivers (NOTCH1, RREB1, HRAS, TP53) used for molecular subtyping are supported by oncodriveFML or not.

Open Access This Peer Review File is licensed under a Creative Commons Attribution 4.0 International License, which permits use, sharing, adaptation, distribution and reproduction in any medium or format, as long as you give appropriate credit to the original author(s) and the source, provide a link to the Creative Commons license, and indicate if changes were made.
In cases where reviewers are anonymous, credit should be given to 'Anonymous Referee' and the source.
The images or other third party material in this Peer Review File are included in the article's Creative Commons license, unless indicated otherwise in a credit line to the material. If material is not included in the article's Creative Commons license and your intended use is not permitted by statutory regulation or exceeds the permitted use, you will need to obtain permission directly from the copyright holder.
To view a copy of this license, visit https://creativecommons.org/licenses/by/4.0/

RESPONSE TO REVIEWERS' COMMENTS

Reviewer #1 (Remarks to the Author): clinical expertise in sebaceous tumour biology and clinical

This is a large and comprehensive study of genomic alterations in sebaceous neoplasms, reaching from benign to malignant tumors. The data generated represents a considerable resource, which is important for the scientific community. While the scope of the study and the number of samples is impressive, there are several significant weaknesses. Overall, it is not fully clear what novel aspects of these tumors has been revealed. The 5 mutational patterns are not fully convincing and the analysis of copy number changes and structural rearrangements not compelling as presented.
The manuscript is difficult to read with many nested sentences full of abbreviations and not always clear meaning and partially problematic grammar. More importantly, no coherent picture is distilled by integrating the multi-omic data to extend the knowledge on the biology of these tumors, i.e. what signaling pathways are consistently altered, what constitutes the barrier between benign and malignant, and how any of these features might relate to clinical course. Outcome data is mentioned to exist but does not seem to feature in the analysis.

We thank Reviewer1 for their constructive comments and insights. As mentioned, we have collected the largest ever dataset of sebaceous tumours, comparing benign (sebaceous adenoma and sebaceoma) and malignant (extra-ocular and peri-ocular sebaceous carcinoma) cohorts which we have examined through extensive computational analyses.

We were able to identify several novel aspects in this study:

1. We discovered (and validate statistically) new driver genes (for example *HRAS, MSH2/MLH1, RB1*) not previously reported in sebaceous tumours and profiled the full spectrum of mechanisms by which previously reported genes are altered to become drivers (i.e. somatic mutation and/or somatic copy number alterations (deletion, amplification or cnLOH)). For example, we detected copy neutral LOH of mismatch repair (MMR) genes and amplification of other drivers such as *GNAS*, *PLAG1* and Hornerin. We also describe new fusions and importantly show that *PAK* fusions are not restricted to apocrine poromas – this is of clinical/diagnostic significance. Remarkably, we also identified pathogenic (exonuclease proofreading defects) *POLE/POLD1* somatic mutations, not previously reported to be linked to sebaceous tumours.
2. We also show, for the first time, that tumours may take different paths to tumourigenesis. For example, Lynch patients can carry STs with a germline and somatic mutations, as well as a germline mutation and a cnLOH event. While other tumours which are pMMR, specifically SCs, exhibited biallelic inactivation of *TP53* co-occuring with *ZNF750* and/or *RB1* disruption (particularly SC-O), suggesting *TP53* might accelerate *ZNF750/RB1*-driven SC as occurs for *BRAF* p.V600E in melanoma. In support of this hypothesis, concomitant *TP53/RB1* mutations were identified in some metastatic SC cases. This study reinforces the important role of *RB1* in malignant STs, with inactivation occurring via somatic mutation, focal deletion (in SC-E) and 13q deletion (in SC-O). Moreover, the spectrum of the somatic mutations in the tumours across the same individual can vary (see patient PD43887).

3. We clearly found that sebaceous tumours have a propensity to develop in the context of a high mutational burden including mismatch repair (MMR) deficiency and UV-damage as previously reported, but also *POLE/POLD1* somatic mutation and APOBEC activation. In addition, *MLH1* promoter hypermethylation was revealed as a rare event in sebaceous tumours, associated with somatic dMMR.
4. We also show that clinical variables such as developing a sebaceous tumour outside the head and neck area, having multiple sebaceous tumours, and presenting with another non-sebaceous Lynch syndrome related neoplasm are good Lynch syndrome predictors. In addition, histopathological variables such as no epidermal connection, tumour delimitated by a collarette, KA-like architecture and diagnosis of SM are also good LS predictors, with clinical variables being better predictors.
5. We demonstrated that indel rate is more reliable than TMB in defining dMMR. In skin, UV-damage increases the TMB and the level of these mutations differs depending on the site and age of the patient, while a high indel burden is largely acquired as a result of dMMR.
6. The method applied to detect Lynch syndrome via the analysis of colon tumours cannot be directly applied to sebaceous tumours. Indeed, unlike colorectal cancers, somatic *BRAF* p.V600E and *MLH1* promoter hypermethylation testing is not relevant to STs and cannot be employed to distinguish sporadic from germline dMMR tumours owing to the absence of *BRAF* p.V600E mutations in these tumours and our observation that hypermethylation of the *MLH1* promoter is a rare event in sporadic dMMR STs.
7. The use of somatic mutational profiling may help in the differential diagnosis of benign versus malignant STs, which can be challenging. For example, malignant STs were driven by chromosomal instability and typically showed biallelic *TP53* inactivation, as well as *TP53* mutations co-occurring with *ZNF750* and/or *RB1* mutations. In addition, dMMR SC-E was characterised by mutations in *NOTCH1, RREB1, and DRD5*, whereas SC-O was characterised by significant focal amplifications encompassing *GNAS/SALL4* and *LYN/PLAG1*, significant broad 13q deletions (encompassing *BRCA2* and *RB1*), and 8q amplifications (encompassing *MYC*), with amplification of the *MYC* proto-oncogene previously reported by targeted sequencing and FISH[11]. Lastly, both SC-E and SC-O showed significant amplification of chromosomes 1q21.3 and 20. The former amplification encompasses *HNRN* (Hornerin), which is part of the epidermal differentiation complex on 1q21.

Despite the enormous size of this cohort and the deep characterization we are still not powered enough to make conclusions about patient outcome because at the time of writing only 4 patients had died of their disease.

Addressing specifically the point about multi-omic/data integration – in Figure 3 we integrate our SNV and expression catalogue to define 9 statistically significant groups.

Regarding the grammar, we have reviewed this carefully and had a professional editor review the text prior to resubmission.

Specific points:

1) Figure 1B y-axis labels should be clarified. What additional tumors qualify? Other STs. Could be phrased more clearly and succinctly.

We thank Reviewer1 for making this interesting comment. The figure shows all additional tumours in each patient that were not sebaceous tumours. The y axis was amended for clarity. The designation of Lynch syndrome related cancers comes from the "Revised guidelines for the clinical management of Lynch syndrome (HNPCC): recommendations by a group of European experts" guidelines/paper published in Gut. 2013 Jun;62(6):812-23 (https://gut.bmj.com/content/62/6/812). We have added the reference into the Figure1 legend (please see revised Manuscript). As discussed in the guidelines there remains some ambiguity as to which tumours are/aren't Lynch related but we followed these consensus guidelines which are used clinically and thus represent the state of the art at this time.

2) Figure 1C is not referenced in the text and the labels are not clear. The pictures are clinical and histological and not clinicopathological

We thank Reviewer1 for pointing out this mistake and we have amended the text and Figure 1 accordingly (please see revised Manuscript and **Figure 1**). We also added Figure 1C to the Supplementary Discussion with more detailed labels and an explanation of the histology.

3) It would be good to show a lollipop diagram for RREB1 as well. Is there any information that can be gleaned about the functional relationship between RREB1 and HRAS mutations? For example, are particular types of RREB1 mutations associated with HRAS mutations? What are the anticipated functional consequences of RREB1 mutations?

We thank Reviewer1 for this suggestion and we added a *RREB1* lollipop diagram in **Supplementary Figure 6**.

Focusing first on the highly recurrent *RREB1* frameshift (p.E1502Rfs*81) mutation, we found that it was not significantly co-occurring with *HRAS* mutation (Chisq-test: p-value=0.624, or Fisher test: p-value=0.602).

		RREB1	
		WT	frameshift_p.E1502Rfs*81
**HRAS**	WT	92	24
	Mut	61	20

In the same way we do not find an obvious association with other events, for example when we examine significance using all samples with frameshift events.

Regarding the function of the *RREB1* mutations – *RREB1* is involved in regulation of cell proliferation, transcriptional regulation and DNA damage repair. We are also aware of

unpublished data suggesting a role for mutation of this gene in immune evasion. From the mutations we called, other than loss of *RREB1* being tumour promoting, we are unable to glean any obvious mechanism. It is possible/likely that loss of *RREB1* contributes to multiple mechanisms of tumorigenesis, and this is something to be explored in future studies.

4) The last sentence on mutually exclusivity is difficult to parse and the pattern of exclusivity not apparent (to me) from figure 2. It looks like many tumors with TP53 mutations also have mutations in RREB1, HRAS, or NOTCH1

Tile plots do not consider gene length or sequence context or variant consequence, which is considered in the statistical model deployed within DISCOVER meaning that it can be difficult to decern genetic interactions by looking at these profiles alone. We note that our analysis with DISCOVER (and also with Fishers; removed from the revised manuscript as requested by the reviewers) was performed without stratification for tumour type, thus including the SC-O cases where the mutual exclusivity between the abovementioned genes is more evident.

We have reviewed these associations and they remain statistically robust.

TP53 and *HRAS* were statistically mutually exclusive, which was demonstrated by DISCOVER (adjusted pvalue = 0.00091) and Fisher (one-tailed pvalue = 0.0381) tests. Although we have samples with both mutations, it was defined as statistically significant, likely driven by SC-O.

We also ran DIALECT (Shuaibi A *et al*. 2024; https://www.ncbi.nlm.nih.gov/pmc/articles/PMC11071465/), an alternative algorithm for identifying co-occurring and mutually exclusive interactions. However, even after corresponding with the authors and rebuilding their code we were unable to generate meaningful output. We therefore conclude that DISCOVER remains the state-of-the art for such analyses and thus we report these results in the paper.

5) The data on copy number changes implicating individual genes is not compelling. GISTIC scores in fig 5 looks noisy rather than showing bona fide copy number changes. This is particularly true for SA and SM, which seem to have overall 'flat' genomes.

We have re-run the analysis as shown below.

Figure 6

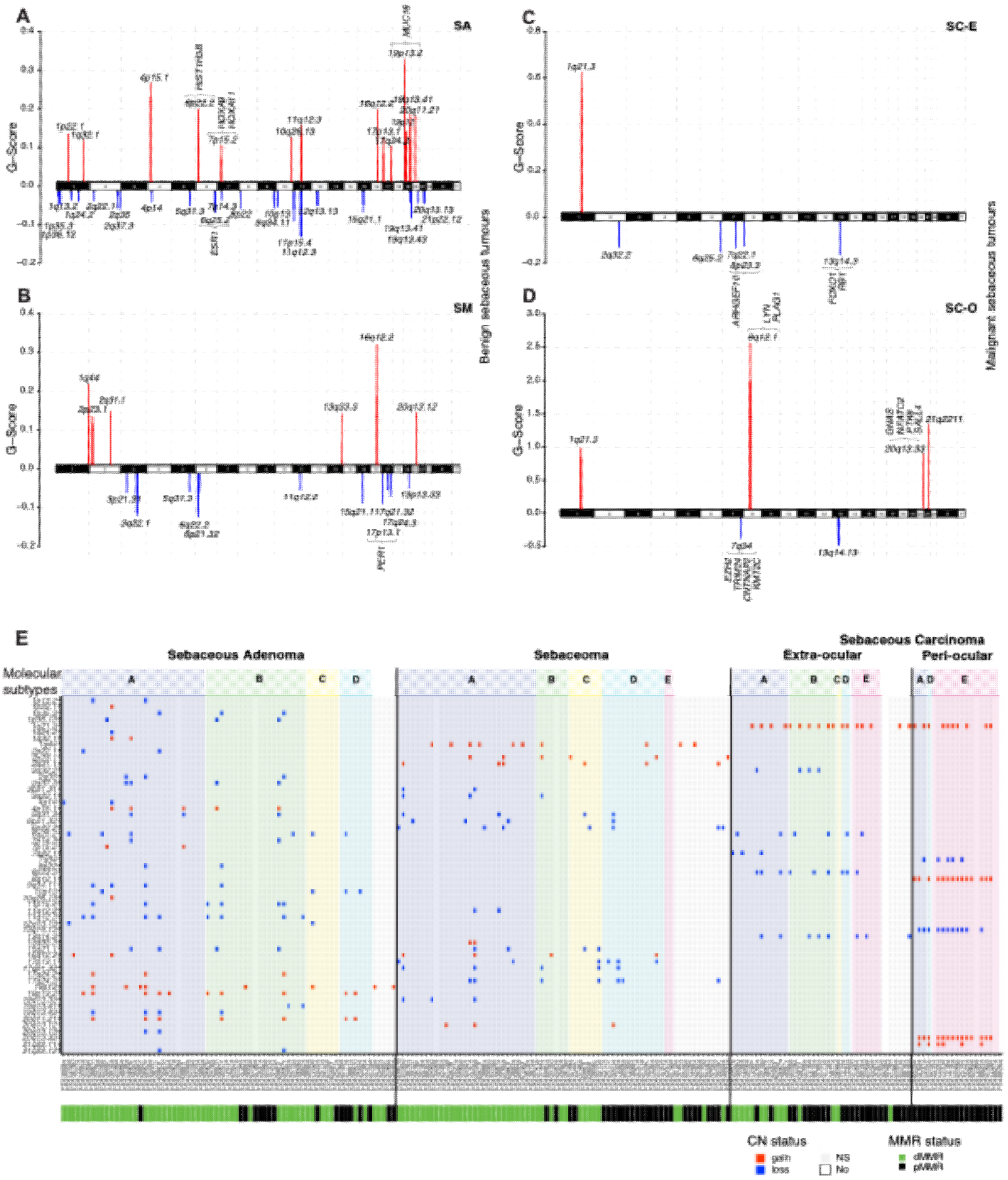

Figure R1/Figure 6 – Significant focal somatic copy number alterations in sebaceous tumours.
Representation of the GISTIC2.0-identified by ST subtype significant focal peak regions (≤ half a chromosome arm) in (A) sebaceous adenomas (SA), (B) sebaceomas (SM), (C) extra-ocular sebaceous carcinomas (SC-E) and (D) peri-ocular sebaceous carcinomas (SC-O). Chromosomal location of the peaks is indicated by the cytoband, with amplifications (gains) shown in red and deletions (losses) shown in blue. The presence of known tumour suppressor genes or oncogenes within these peak regions are labelled. For the G-score, positive values refer to gains, while negative values are related to deletions. (E) Oncoplot showing the individual tumour samples with the amplification and deletion regions shown in A-D. Samples with no significant gains or losses are indicated by ‘NS’. Five samples were not included in the GISTIC2.0 analysis (see **Methods**), so no data is shown (indicated by ‘No’). Abbreviations: MMR, mismatch repair; dMMR, deficient in MMR; No, not analysed; NS, not significant; pMMR,

proficient in MMR; SA, sebaceous adenoma; SM, sebaceoma; SC-E, extra-ocular sebaceous carcinoma; SC-O, peri-ocular sebaceous carcinoma.
Note: *focal peaks were considered as significant when reaching the 4 criteria: 1. residual q-value < 0.1, 2. peak size >= 100,000bp, 3. concordance with ASCAT CN calls ≥ 0.75, 4. portion of peak overlap with Genome In A Bottle 'difficult regions' <= 0.4 (**Supplementary Table 10**); **approximate cytobands are shown with related genomic coordinates available in **Supplementary Table 10.**

The sentence on page 8 line 342 onwards on amplifications of specific genes cannot be understood.

SC-Os showed the greatest number of focal SCNAs (**Figure R1/Figure 6**). Oncogenes in these regions included *GNAS*, *NFATC2*, *PTK6* and *SALL4* (14/15), and *LYN* and *PLAG1* (14/15). We also observed deletions of the TSGs *EZH2*, *TRIM24*, *CNTNAP2* and *KMT2C* (5/15). Of note, the focal SCNAs in SC-Es only included known TSGs, specifically *FOXO1* and *RB1* (8/36), and *ARHGEF10* (9/36).

Also, what constitutes a 'significantly focal amplification'?

We have changed significantly focal to significant focal amplification.

To find significant recurrent amplifications and deletions, the output of ASCAT was used to generate input files for GISTIC2 (v2.0.23)[65], which requires segment coordinates, the number of markers and a log2-scaled copy number for each segment. GISTIC2 was run with the following parameters: -refgene hg38.UCSC.add_miR.160920.refgene.mat -genegistic 1 -smallmem 1 -broad 1 -brlen 0.75 -conf 0.95 -armpeel 1 -savegene 1 -gcm extreme -v 20 -ta 0.25 -td 0.25. The hg38.UCSC.add_miR.160920.refgene.mat file is included with GISTIC2. The threshold for the residual q-value for both broad and focal amplifications and deletions was set at 0.10. To identify driver events, we focused on genes within significant amplifications and deletions <= 10Mb in size. To remove artefactual recurrent SCNAs, we further removed GISTIC2 wide peaks with an overlap of more than 40% with regions known to be problematic for sequencing and/or read mapping, as defined by the Genome in a Bottle Consortium benchmark union (v3.3; https://ftp-trace.ncbi.nlm.nih.gov/ReferenceSamples/giab/release/genome-stratifications/v3.3/GRCh38@all/Union/GRCh38_alldifficultregions.bed.gz). Finally, we also excluded a wide peak if the list of samples predicted to have a particular SCNA in GISTIC2 did not concur with the original ASCAT results.

i.e. to be defined as a significant focal copy number event a region needs to have a GISTIC2 residual q-value < 0.1, a peak size **>** 100,000bp, show concordance with the ASCAT CN call of >= 0.75, and for the region to not overlap with a Genome In A Bottle "difficult regions" by >40%.

The manuscript was amended to clarify the above (please see revised Manuscript).

Compelling examples of the raw copy number data for example for HRNR and KIT amplifications should be shown for this section to be more compelling, ideally with additional supporting information nominating these genes (FISH, IHC).

Looking at absolute CN calls for Hornerin (HRNR) and KIT, we can clearly see these 2 genes are amplified as shown below:

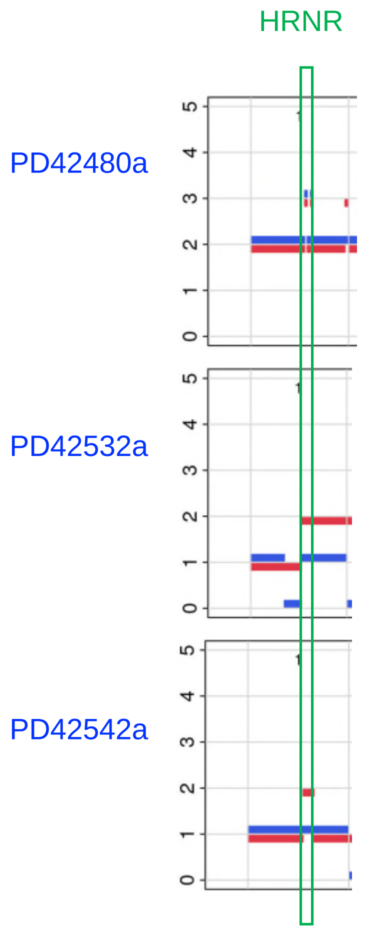

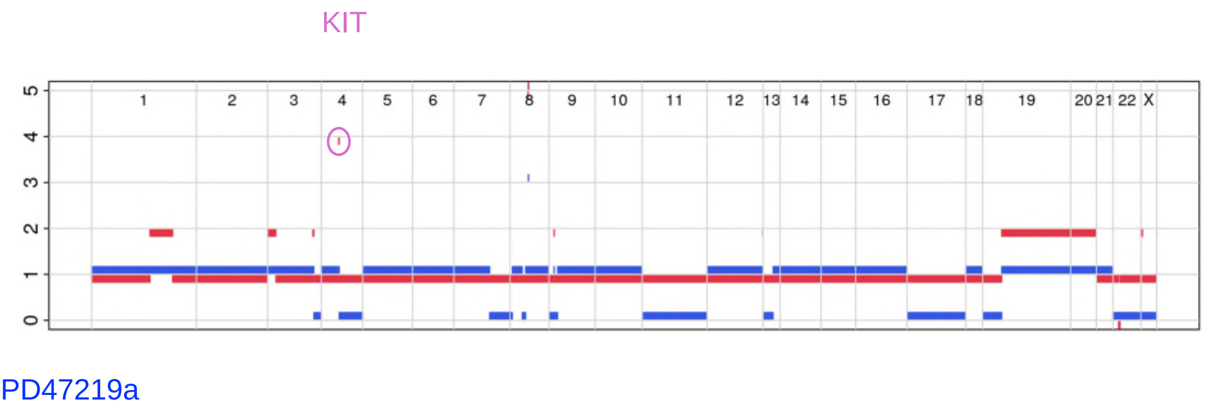

Figure R4 - Absolute CN calls of *KIT* and *HNRN* in sebaceous tumours.

We do not have the material to do FISH and based on the RNA-Seq data we know that these genes are expressed so we don’t feel that IHC will be informative and at best such an analysis would be semi-quantative.

A better integration with mutation data is desirable when nominating specific genes based on copy number change. E.g. how would an amplification of GNAS be plausible in this tumor type, in which no activating mutations of GNAS are seemingly present?

We looked at the *GNAS* amplification (as well as the other candidate oncogenes on the same cytoband). None of the amplified *GNAS* sample had a co-occurrent somatic mutation of *GNAS* (same for *SALL4, NFATC2* and *PTK6*). Please see "NatureComs_GNAS" in "SupplementaryMaterial" folder.

Several cancer-related genes are frequently amplified but not commonly mutated in tumours. Amplification generally refers to the increase in the number of copies of a particular gene, which can lead to overexpression/mis-expression of the encoded protein and in this way contribute to tumorigenesis. This does not have to be with concomitant mutation of the gene.

Here are some key examples:

1. *ERBB2* (HER2/neu)
- Function: Encodes a receptor tyrosine kinase involved in cell growth and differentiation.
- Amplification: Commonly amplified in breast cancer, gastric cancer, and other solid tumours.
- Clinical Significance: Overexpression of *HER2* is associated with aggressive tumour behaviour and poor prognosis. HER2-targeted therapies (e.g., trastuzumab) are effective in treating cancers with *HER2* amplification.

2. *MYC* (*c-MYC*)
- Function: Encodes a transcription factor that regulates genes involved in cell proliferation, growth, and metabolism.
- Amplification: Often amplified in various cancers, including breast cancer, lung cancer, and neuroblastoma.
- Clinical Significance: *MYC* amplification is associated with high tumour grade, aggressive behaviour, and poor prognosis.

3. MDM2
- Function: Encodes a protein that inhibits the tumour suppressor *p53*.
- Amplification: Frequently amplified in sarcomas, glioblastomas, and certain carcinomas.
- Clinical Significance: *MDM2* amplification leads to *p53* inactivation, contributing to tumour progression. It is a potential target for cancer therapies.

4. *CCND1* (Cyclin D1)
- Function: Encodes a protein that regulates cell cycle progression.
- Amplification: Commonly amplified in breast cancer, head and neck cancers, and lymphomas.
- Clinical Significance: *Cyclin D1* overexpression due to gene amplification drives cell proliferation, making it a target for therapeutic intervention.

5. *EGFR* (Epidermal Growth Factor Receptor)

- Function: Encodes a receptor tyrosine kinase involved in cell growth and survival.
- Amplification: Frequently amplified in glioblastomas, non-small cell lung cancer, and other tumours.
- Clinical Significance: *EGFR* amplification is associated with increased cell proliferation and survival, and EGFR-targeted therapies are used in treating certain cancers.

6. *MET*

- Function: Encodes a receptor tyrosine kinase involved in cellular growth, motility, and survival.
- Amplification: Often amplified in gastric cancer, lung cancer, and papillary renal cell carcinoma.
- Clinical Significance: *MET* amplification can lead to constitutive activation of the receptor, driving oncogenesis. Targeted therapies against *MET* are being developed.

7. *CDK4*

- Function: Encodes a cyclin-dependent kinase that plays a key role in cell cycle progression.
- Amplification: Commonly amplified in melanoma, sarcoma, and glioblastoma.
- Clinical Significance: *CDK4* amplification promotes cell cycle progression and is a target for CDK4/6 inhibitors in cancer therapy.

8. *FGFR1* (Fibroblast Growth Factor Receptor 1)

- Function: Encodes a receptor tyrosine kinase involved in cell proliferation, differentiation, and angiogenesis.
- Amplification: Amplified in lung squamous cell carcinoma, breast cancer, and other tumours.
- Clinical Significance: *FGFR1* amplification drives tumorigenesis, and FGFR inhibitors are being explored in clinical trials.

These genes are frequently amplified in various cancers, only occasionally with mutation, and their amplification often plays a critical role in driving tumour progression, making them key targets for cancer therapies.

The wording and grammar in these paragraphs is awkward e.g. 'In contrast, SC-Es showed significantly chromosomes 1 and 20 amplifications, and significantly 17p deletions, which includes TP53 (7/36 tumours, of which 6 also had a somatic TP53 mutations'

The entire edited paragraph is below: "At a chromosome arm level, SC-Os showed the greatest number of significant recurrent SCNAs, including chromosomes 1q, 8q, 19p and 20 amplifications, as well as chromosome 4 and 13q deletions (**Supplementary Figure 10A-D**), with the 13q deletion encompassing *RB1* and *BRCA2* (8/15; one of them being somatically mutated for *RB1*) (**Supplementary Figure 10E**). In contrast, SC-Es showed significant chromosomes 1 and 20 amplifications, and significant 17p deletions, which encompass *TP53* (7/36 tumours, of which 6 also had a somatic *TP53* mutations; **Supplementary Table 11**)." (please see revised Manuscript).

6) Additional consideration and discussion should be given to the PAK2 fusions. The fusions with PAK2 at the 5' end appear to be novel/unprecedented. Is there a reciprocal fusion product that was perhaps missed?

Notably, *PAK2* fusions (*PAK2::DLG1, PAK2::LRIG1* and *DLG1::PAK2*) were identified **Supplementary Figure 11A** and **Supplementary Table 14**) with the reciprocal event *DLG1::PAK2* observed in the case with a *PAK2::DLG1* fusion. We did not identify a reciprocal event in the tumour with the *PAK2::LRIG1* fusion which might suggest it is expressed below the level of detection or it is not generated. Importantly, our analysis suggests that *PAK2* fusions are associated with benign tumours and with sebaceous differentiation as was recently reported in apocrine poroma as well as in two SMs and one SA, without specific features, in our cohort (**Supplementary Table 14**).

7) The finding of an identical fusion of RCOR1::GRHL2 needs additional discussion as it implies a clonal relationship between the two SAs of this patient.

Out of the two sebaceomas samples from this patient sample PD43859a did not passed DNA QC. Thus, to explore the question of a shared clonal origin further we compared the mutations found in the DNA/exome sequence of PD43859c to variants called from the transcriptome sequence of PD43859a.

This analysis revealed:

- Of the 7 sites called as somatic variants from PD43859c with sufficient coverage in **PR43859a** 5 / 7 variant calls were concordant. i.e. they appear as somatic variants in both PR43859a and PD43859c.
- If we consider all sites, at least 8 / 15 ((5+3) / 15) variants in 'c' are found in 'a'; however, 5 sites had coverage < 10X and therefore may be false negatives.

Collectively, these observations do suggest a shared origin for a and c. However, these two samples were both benign and collected with a time gap of 3 years between each excision. This time gap means that it is unlikely to be a recurrence. We therefore wonder if these tumours have a shared, possibly post-zygotic, origin with this specific patient noted as having multiple (many) sebaceous cysts in their clinical notes. This is an intriguing observation indeed.

8) The finding on CIN primarily in pMMR tumors is interesting and mirrors the findings in CRC. This indicates, as you state, that genomic instability is necessary for the malignant transformation. Any insights on why and how?

Comparisons of CIN (measured in terms of numbers of gains and losses of chromosome arms or long DNA segments therein) in pMMR and dMMR colorectal cancers (CRC) has shown a clear difference with pMMR CRCs showing significantly more CIN than dMMR CRCs. These data are consistent with the hypothesis that at least one form of genomic instability, either CIN as seen in the majority of CRC that are pMMR, or dMMR is required to allow transformation to malignancy. Here, we hypothesise similar mechanisms occurring in

sebaceous tumours with CIN in pMMR or MSI in dMMR cases. Underlying CIN there can be increased replication stress and defects in mitotic spindle assembly, errors in chromosome cohesion and segregation, centrosome amplification, and/or dysfunctional checkpoints. Telomere dysfunction and epigenetic mechanisms may also contribute.

References:

DNA Mismatch Repair Deficiency in Sporadic Colorectal Cancer and Lynch Syndrome.
Poulogiannis G, Frayling IM, **Arends MJ**. *Histopathology* 2010; **56**: 167 - 179.

Prognostic relevance of DNA copy number changes in colorectal cancer.
Poulogiannis G, Ichimura K, Hamoudi R, Luo F, Leung SY, Yuen ST, Harrison DJ, Wyllie AH, **Arends MJ.** *Journal of Pathology* 2010; **220**: 338 - 347.

Lynch Syndrome – Cancer Pathways, Heterogeneity and Immune Escape
Seth S, Ager A, **Arends MJ**, Frayling IM.
J Pathology 2018 246(2):129-133. doi: 10.1002/path.5139. PMID: 30027543

Molecular Pathology of Lynch Syndrome.
Cerretelli G, Ager A, **Arends MJ**, Frayling IM.
J Pathol. 2020 Apr;250(5):518-531. doi: 10.1002/path.5422. PMID: 32141610

Lynch Syndrome. Frankel W, **Arends MJ**, Frayling IM, Nagtegaal ID. In: "World Health Organization Classification of Tumours of the Digestive System". 5th Edition. IARC Press: Lyon, France, 2019. Chapter 14, pp515-521. Genetic Tumour Syndromes of the Digestive System. ISBN 978-92-832-4499-8.

Genetic tumour syndromes associated with skin malignancies: Muir-Torre syndrome.
Tetzlaff M, **Arends MJ**, Wood BA. 2024, World Health Organization Classification of Skin Tumours. 5th ed. IARC Press, Lyon, France. Chapter 9, Section 9.7 (in press)."

We thank Reviewer 1 for their comments.

Reviewer #2 (Remarks to the Author):

General comments: expertise in multi-omics integration

In this multi-center international study, Ferreira et al. present the largest exome and transcriptome analysis of sebaceous tumors (ST) to date. This allowed investigators to

identify mutated genes under positive selection (drivers) for the first time in this tumor type, which provides an incredibly valuable resource to the cancer research community. In my opinion, this is an extremely important study for the ST and cancer genomics fields.

We thank Reviewer 2 for supporting our project and highlighting the fact this is an incredibly valuable resource and extremely important study.

The initial data processing, including variant calling, mutation signature extraction, and copy number profiling, was conducted using well-established software and pipelines. However, several downstream analyses are problematic, and the presentation of the results can be significantly improved to enhance clarity and better guide the reader. Furthermore, in my opinion, parts of the neoantigen and ligand-receptor analyses are too speculative, and they lack additional data or experimentation for validation. These sections should be modified for clarity and rigor, and their results presented more cautiously or the authors may want to consider omitting these analyses from the study.

We thank Reviewer2 for sharing their concern about the neoantigen and ligand-receptor analyses. We agree these analyses are purely speculative and that follow-up functional analysis are required to confirm them. We have significantly reduced or removed these analyses or moved them to the supplementary information (Figure 7 has become **Supplementary Figure 15, for example**) and we have rephrased the text to make clear follow-up studies will be required for validation.

My recommendation is to improve the whole exome and transcriptome analysis as outlined in detailed below and re-consider inclusion of the ligand-receptor analysis and parts of the neoantigen analysis.

We thank the reviewer for their comments which we have addressed in full below.

Concerns/suggested revisions:

Somatic landscape of sebaceous tumours
Driver Identification Analysis:

[1] The paper reports significantly mutated genes based on a single software tool (dNdScv), without the necessary validation steps (e.g. filtering for genes that are not expressed or have low VAF), raising concerns about false positives that are ubiquitous in the context of high TMB tumors. (For example, SPANXB1 is reported as a driver in Figure 2, but most mutations in this gene appear to have a suspiciously low VAF). To ensure robustness, the authors should filter their results to exclude likely false positives and, importantly, provide additional supporting evidence for the novel candidate drivers:

- What is the variant allele frequency (VAF) distribution for each gene?

As requested, we have made a plot showing the VAF distribution for each driver gene (please see Figure R5). We can observe that *SPANXB1* is not outside the distribution, while

ZNF750 (VAF (mean): 0.479661) is the only statistically significant outlier based on the interquartile range (IQR) method (please see **Figure_R5** in "SupplementaryMaterial" folder). i.e. only *ZNF750* is outside the distribution with the VAFs for this gene being higher than for other drivers.

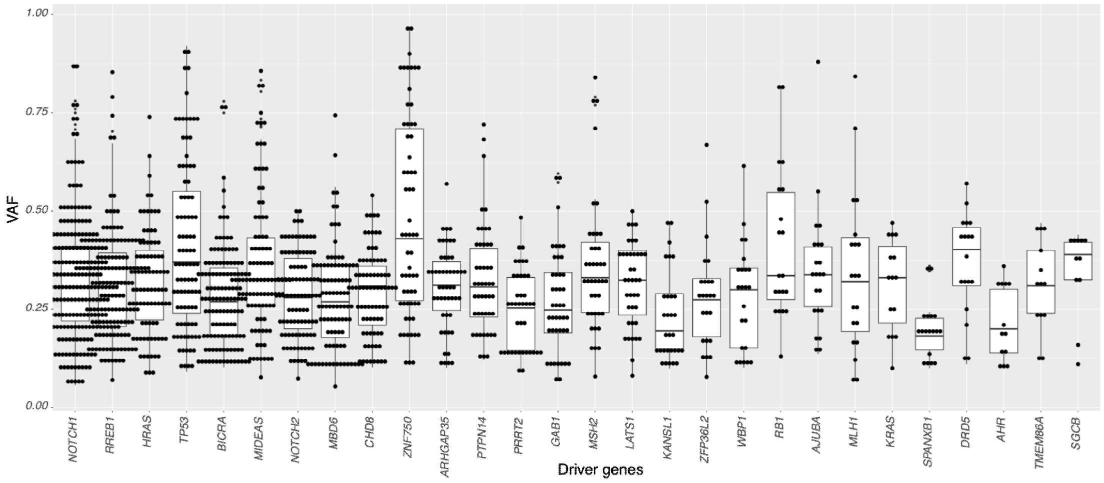

Figure R5 - Driver genes and variant allele frequencies (VAF) in sebaceous tumours. (A) Barplot showing the distribution of the VAF across the driver genes. Each dot is a tumour.

● Do these variants mostly originate from a specific signature or positional site?

As requested, we have made a plot showing the VEP variant classifications across the 28 driver genes identified in this study (**Figure R6/Supplementary Figure 4A**). Further, we also provide a figure illustrating the profile of nucleotide changes found in these genes (**Figure R7** see below). This analysis revealed that most driver genes are characterised by a range of variant types, except *HRAS*, *TMEM86A* and *SGCB*, which contain only missense mutations suggesting they are oncogenes.

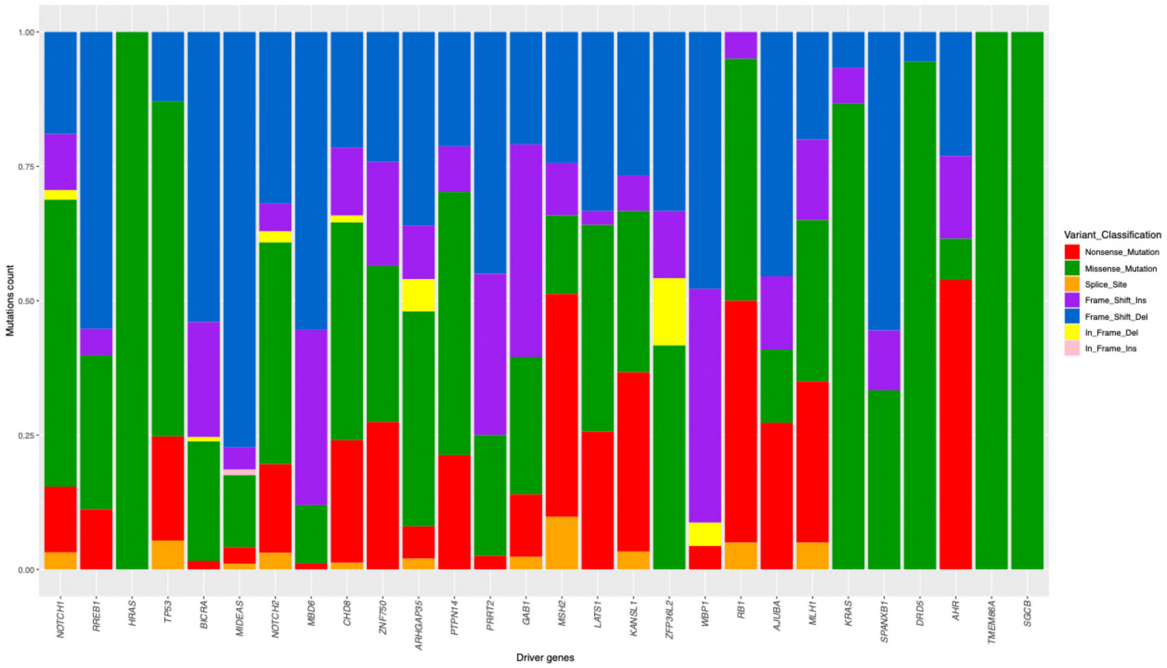

Figure R6/Supplementary Figure 4A - Driver genes and associated variant types in sebaceous tumours. Barplot showing the proportion of the different variant types across the driver genes.

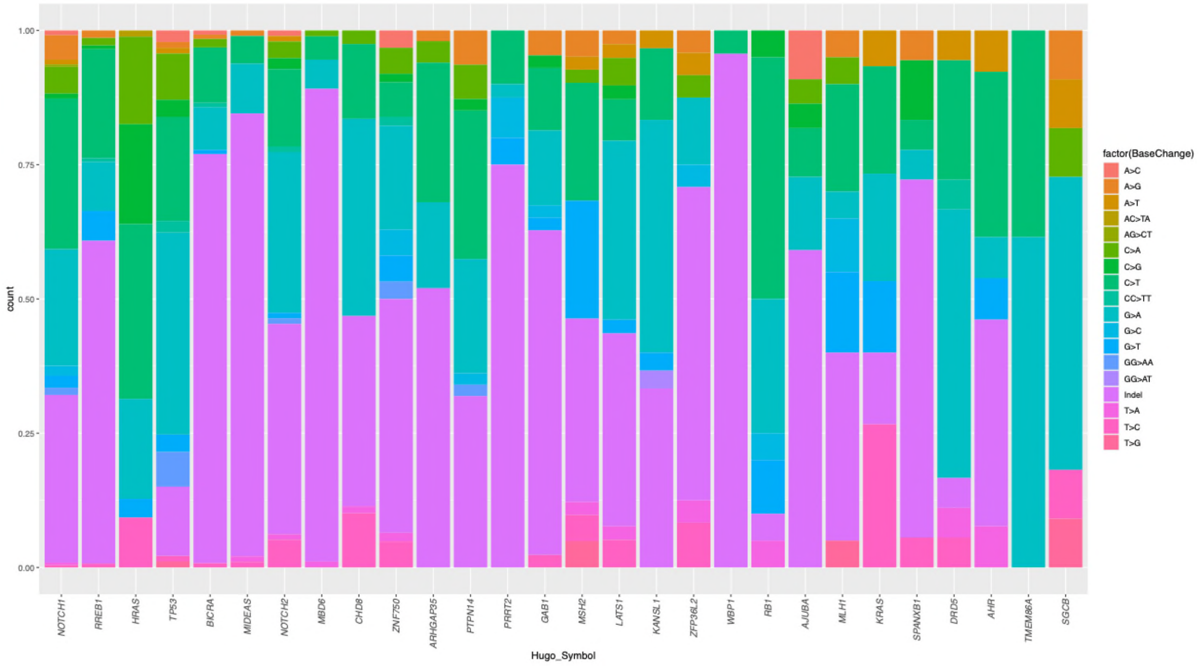

Figure R7/Driver genes and associated nucleotide changes in sebaceous tumours. Barplot showing the proportion of the different nucleotide changes across the driver genes.

- What proportion of variants are seen in the RNA-Seq data for each gene?

As suggested, we checked for the presence of the somatic variants call from our exome data in the RNA-seq data generated on each case. We can observe that most of the somatic mutation found in the DNA are present/expressed in the RNA-seq.

Variant type	Mutations with coverage > 10x in RNAseq	Total mutations observed in RNAseq	Mutations found in RNAseq	Variants with <= 10x coverage in RNAseq
SNV	676	667	98.7%	202
DNV	15	15	100%	5
DEL	414	407	98.3%	104
INS	128	133	96.2%	55

- Are there significant co-occurrences of loss-of-function mutations with loss of heterozygosity (LOH) for the putative tumor suppressor genes (TSGs)?

For each driver genes, we have calculated the percentage of LOH and made a new plot for(below):

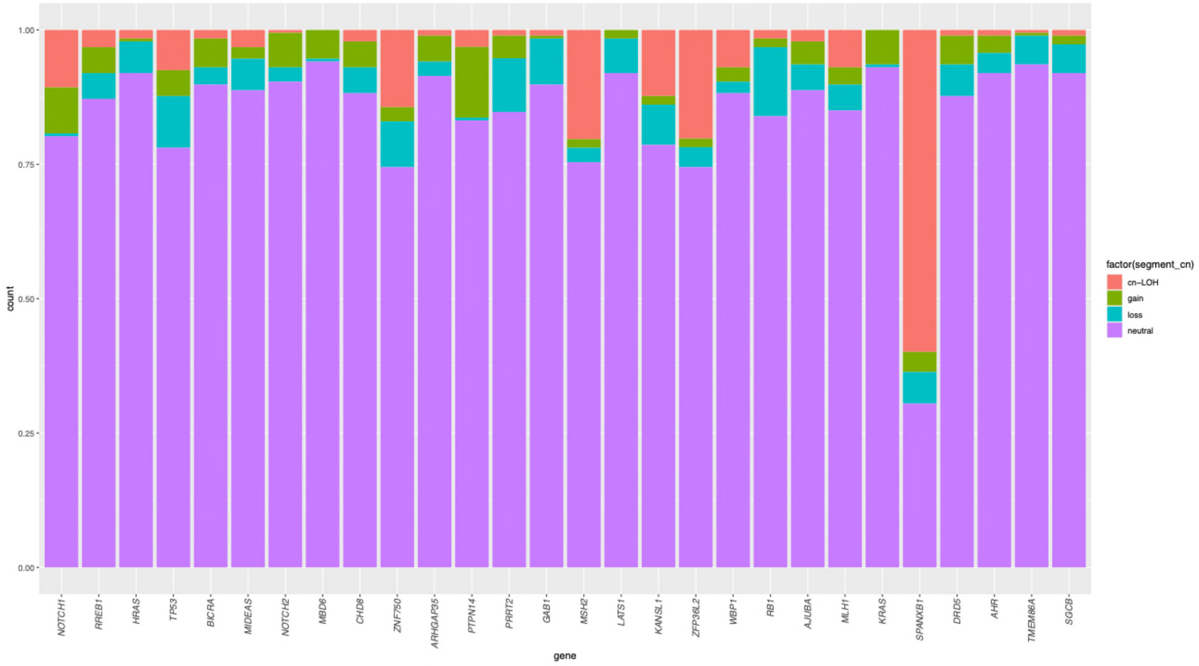

Figure R8 - Supplementary Figure 4C - Driver genes and associated copy number alterations in sebaceous tumours. Barplot showing the proportion of the different copy number alterations across the driver genes.

We can see that some of the established oncogenes we detected were amplified (such as *KRAS*), while loss and cnLOH of TSGs (such as *MSH2* and *RB1*) was identified. Surprisingly, *SPANXB1* is frequently affected by cnLOH.

[1.1] The authors of the dNdScv software have excluded hypermutator samples (>500 mutations) from their main analysis (iMartincorena et al, 2017, PMID:29056346). Such precautions are not mentioned in the method section of the current manuscript. Did they authors try running dNdScv exclusively on tumors with fewer than, say, 1000 or 2000 mutations to see how it affects the results?

We thank Reviewer2 for this comment. We consulted the lead-author of the dNdScv software, Inigo Martincorena, who is a member of our research division. He explained to us that “500 mutations” was an arbitrary number randomly chosen without any statistical testing/justification. As noted, we performed the dNdScv analysis on all mutations but in response to the reviewers comment we also provide the analysis performed with other cut-offs. More specifically, we ran dNdScv removing samples with >2000, >1000 and >500 mutations. Critically, regardless of the cut-off applied, 88%, 60% and 40%, of driver genes were retained when running the analysis across the entire cohort with driver gene detection appearing to mainly be affected by the number of samples used for the analysis at each TMB level. By providing this analysis in the revised manuscript readers can explore the data across these threshold levels. See “Supplementary_Table_5”).

[1.2] There are 24 samples with no matched normal. It is important to exclude these samples from the dNdScv analysis as they could contain germline variants misclassified as somatic (I've noticed VAF modes near 0.5 for many of these samples). Could the authors specify which samples have been included in their analyses, along with any exclusion criteria?

We thank Reviewer2 for this comment. The additional 24 samples were only included in the plots in **Supplementary Figure 3**. No other analyses included them.

[1.3] Possible additional figures for the driver analysis (in main or supplementary): A barplot showing the relative proportion of each variant class (missense, frameshift stop gain etc) in each gene or selected genes. A scatter plot showing dN/dS:missense vs dN/dS:Lof similar to Figure 2B in Martincorena et al.(2017, PMID: 29056346). These figures would help support the analysis results and importantly inform about the action mode of the candidate drivers (i.e. oncogene vs.TSG).

As requested, the two new plots were generated and included in **Supplementary Figures 4A-B** in the revised manuscript.

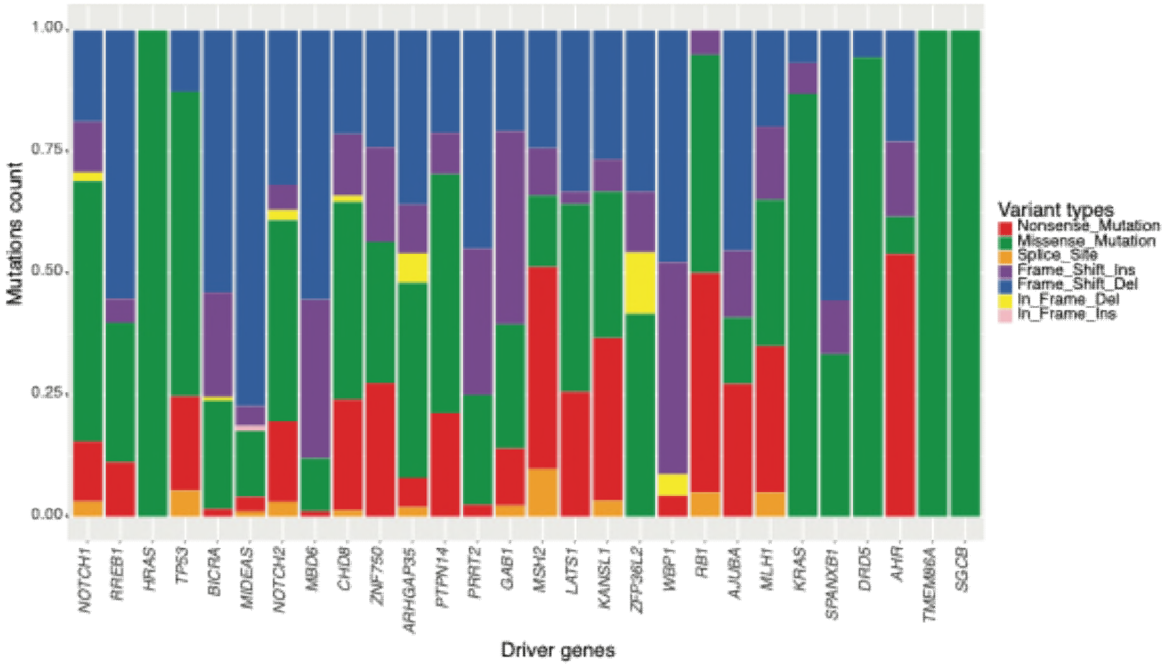

Figure R9/Supplementary Figure 4A - Driver genes and associated variant types in sebaceous tumours. (A) Barplot showing the proportion of the different variant types across the driver genes.

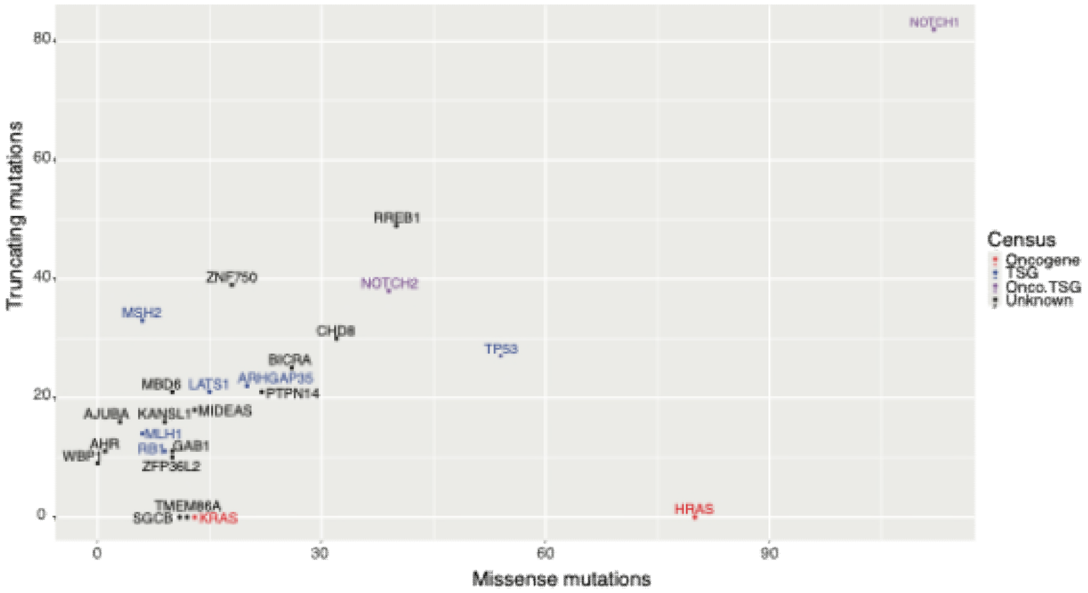

Figure R10/Supplementary Figure 4B - Driver genes and associated variant types in sebaceous tumours. (B) Scatterplot showing missense versus truncating mutation profile across the driver genes.

[1.4] It would be helpful if the authors cross-reference their gene list with the results of some pan-cancer analyses, such as Bailey et al. (2018, PMID: 29625053) or Martincorena et al. (2017, PMID: 29056346). This information could be easily summarized in a supplementary figure having genes as rows and cancer types as columns, with each tile colored according to their report status (e.g. oncogene, potential oncogene, tsg and potential tsg, which is available in Supplementary Table 1 of Bailey et al.). It would be useful for the readers for genes that haven't been reported as significantly mutated, what does the literature say about them that could support their role as an oncogene or tumor suppressor?

We made the plot requested cross-referencing our results with those of Bailey et al. (2018, PMID: 29625053) and we have added it to **Figure R11/Supplementary Figure 4D**.

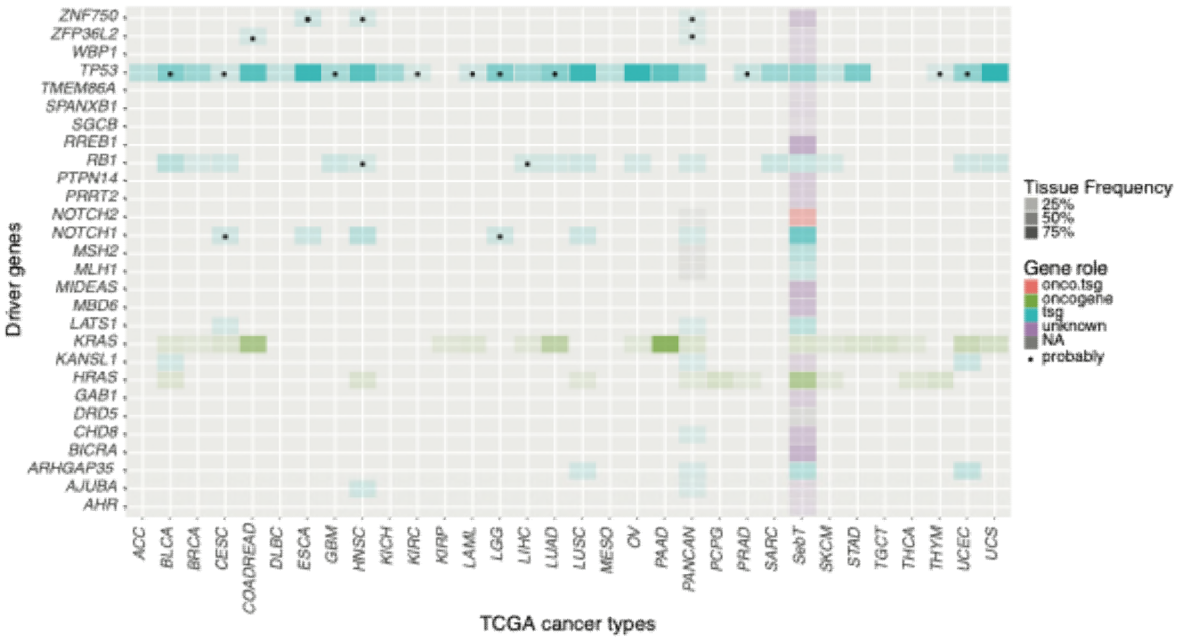

Figure R11/Supplementary Figure 4D – Comparison of mutation consequences in sebaceous tumours driver genes and across TCGA cancers. Tileplot showing the alteration of the driver genes across sebaceous tumours and pan-cancer[161]. The function of each gene has been defined previously[161]. Abbreviations: NA, not defined; onco, oncogene; onco.tsg, oncogene and tumour suppressor genes; tsg, tumour suppressor gene.

[1.5] I speculate the authors may have missed the identification of several drivers by testing all genes as they did impose a burden on significance levels after multiple testing corrections. In addition to their global analysis, I think it may be worth performing a restricted hypothesis testing (RHT), such as in Lawrence et al. (2014, PMID: 24390350) by considering only previously reported genes, for example those from Bailey et al. (2018), and those reported as drivers in sebaceous tumors. This additional analysis would be straightforward, requiring only the subsetting of the global results to these genes before applying multiple testing corrections. This may be valuable to the readers.

As requested, we have performed restricted hypothesis testing (RHT) with the dNdScv algorithm using the list of established driver genes reported by Bailey et al., (please see "Supplementary Table 5"). This analysis increased the detections of candidate driver genes as predicted (19 new genes were identified as driver while the previous reported driver genes were kept). We have drawn another oncoplot with this new list of driver genes and this can be found in **Figure R12/Supplementary Figure 5**.

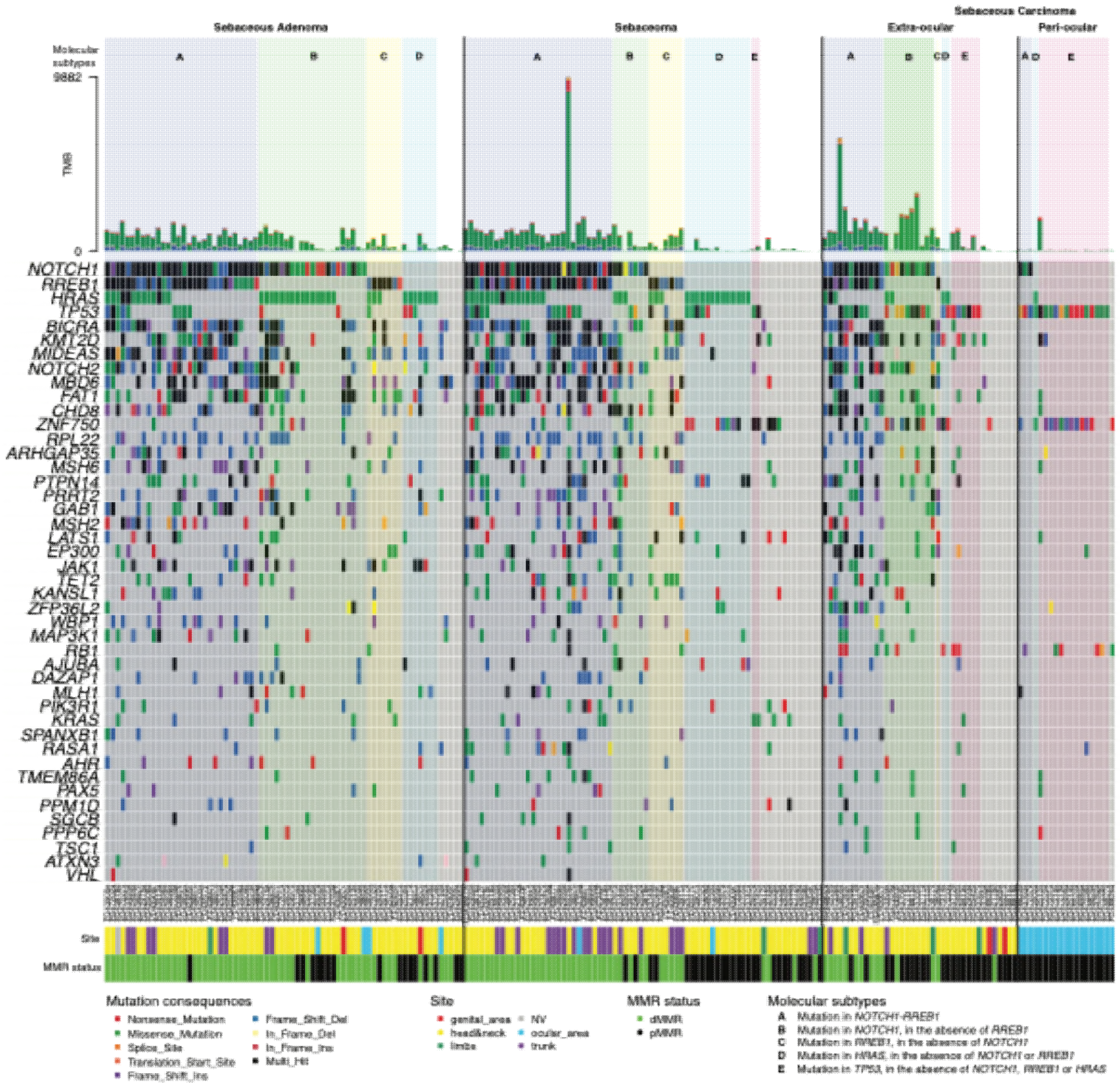

Figure R12/Supplementary Figure 5 - Somatic mutational landscape (driver genes post restricted hypothesis testing (RHT)) of sebaceous tumours across tumour subtypes. Oncoplot showing somatic mutations in the driver genes after RHT (ranking based on the percentage of samples altered (decreasing order)) of sebaceous tumours (STs). This includes the 44 driver genes identified by dNdScv when taking all four ST subtypes together, and performing RHT. Patterns of mutation of the top 4 driver genes (*NOTCH1, RREB1, TP53* and *HRAS*) allows division of the STs into five molecular subtypes (A-E) across the four ST subtypes. 'Site' is the body location of the tumour. The 'PD' number is the sample number, with those in red font being from patients that developed metastases. TMB is in mutations/MB, including SNVs, MNVs and indels). Abbreviations: NV, not available.

[1.6] The main oncoplot could be enhanced in several ways.

• Adding copy number track for the most frequently affected genes (deleted TSG or amplified oncogenes).

• Some drivers with less supporting evidence or of lower interest could be removed from the main figure for simplicity and clarity (but shown in a full supplementary oncoplot).

• Annotation tracks with tumor purity, RNA subtypes and mutational signature groups could

be added.
• Mode of action of each driver (TSG vs oncogene) could be highlighted.

As requested, we have added a track highlighting the proposed mode of action of each driver in **Figure R13/Figure 2** (red for oncogene and blue for TSG). However, after trying to add all the requested information, the figure became unreadable, so we elected to retain this information in other figures and in the supplementary Tables. Regarding the SCNAs that overlap with the driver genes, the only gene significantly recurrently deleted is *RB1*, which can be seen in Figure 5 as a significant peak for SC-E (cytoband 13q14.3) and as a broad peak/arm in SC-O in Supplementary Figure 10 (Arm 13q). Therefore, we did not include this track in the figure.

Figure 2

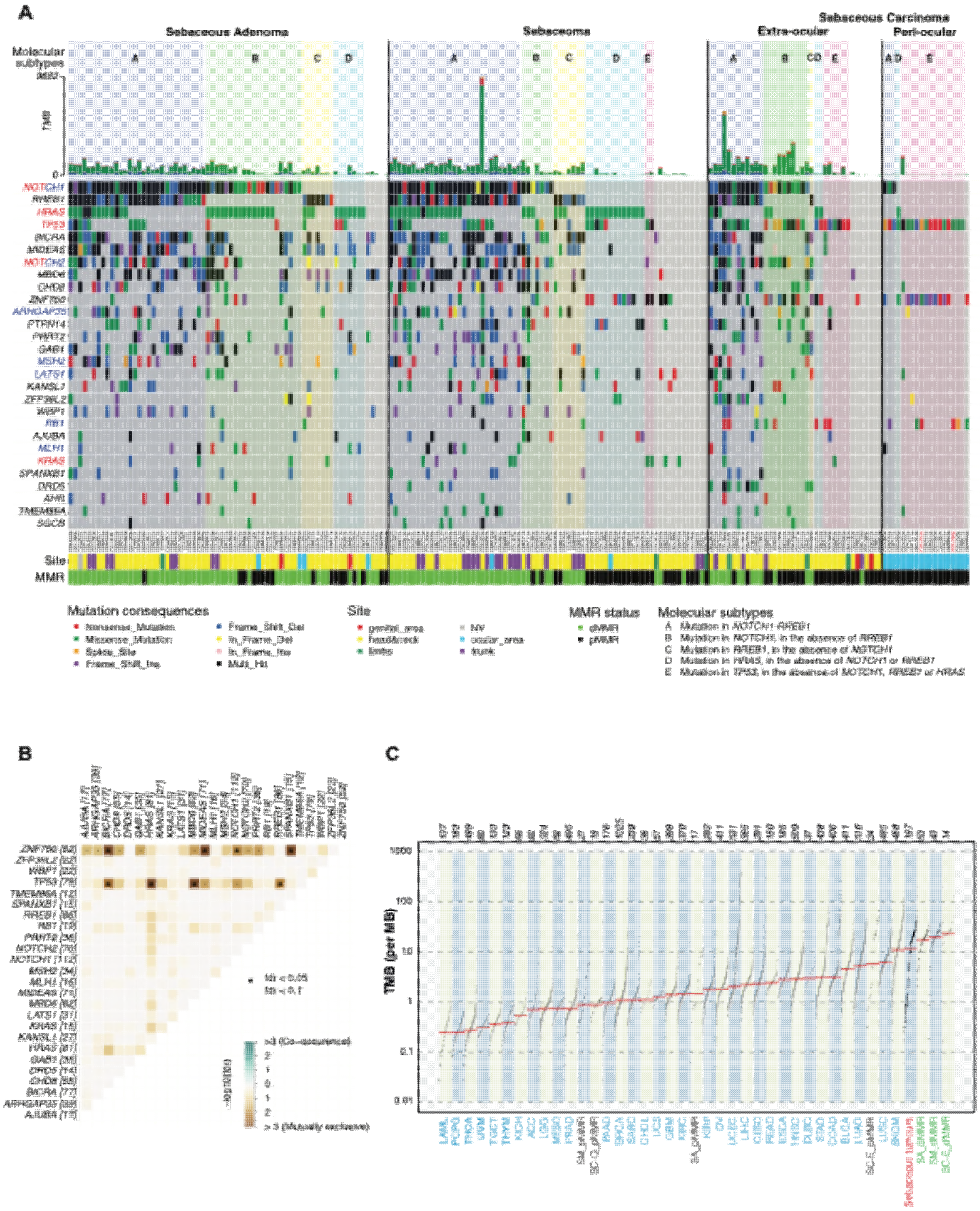

Figure R13/Figure 2 – The somatic mutational landscape of sebaceous tumours across subtypes.
(A) Oncoplot showing somatic mutations in the driver genes of sebaceous tumours (STs). This includes the 25 driver genes identified when taking all four ST subtypes together, and three additional driver genes when considering only specific ST subtypes. Gene names in red denote oncogenes, genes in blue denote tumour suppressors, while genes names in both colours are those reported to have both functions depending on the text. Patterns of mutations in the top 4 driver genes (*NOTCH1, RREB1, TP53* and *HRAS*) allows division of the STs into five molecular subtypes (A-E) across the four ST subtypes. 'Site' is the body location of the tumour. The 'PD' number is the sample number, with those in red font being from patients that developed metastases. (B) Mutual exclusive and co-occurring interactions based on DISCOVER. (C) Comparison of the tumour mutation burden

(TMB) of the sebaceous tumours (STs) against 33 TCGA cohorts (TCGA tumour types are shown using standard abbreviation). The ST cohort are shown all together (blue font), or separated by subtype and mismatch repair (MMR) status (green and red font). Abbreviations: MMR, mismatch repair; dMMR, deficient in MMR; pMMR, proficient in MMR; NV, not available; TMB, tumour mutational burden (in mutations/Mb, including SNVs, MNVs and indels).

[1.7] The authors only report the gene names with their corrected p-values in the supplementary material. It is important to include the complete dNdScv output in the supplementary material to provide a comprehensive view of the analysis and facilitate further validation and reproducibility of results.

As requested, we provided the data into “NatureComs_dNdScv_Output” in “SupplementaryMaterial” folder) and we will also make this available via the GitHub repository.

[1.8] On lines 199-200, the authors write, "Interestingly, when considering the ST subtypes individually, HRAS was only identified as a driver gene in the benign tumors." What is the uncorrected p-value for HRAS in the SC-E and SC-O subgroup analyses? Would it pass RHT? Even if not significant, the oncoplot shows several HRAS mutations in SC-E and SC-O, most of which are known activating mutations at G12/13 and Q61. Moreover, HRAS harbors no silent mutations in these tumors, strongly suggesting it is an actual driver, albeit less frequently mutated. Accordingly, it would be more appropriate to drop this statement or at least revise it to "HRAS was not reported as significant in (...) probably due to its lower mutation frequency and the smaller size of these groups (...)," and mention the presence of these hotspot mutations. In general, the absence of a significant result for a particular gene in a subgroup analysis cannot be used on its own to suggest an absence of “driver” activity.

We thank Reviewer2 for this comment and have amended the text as follow:
“Interestingly, when considering the ST subtypes individually, *HRAS* was only identified as a statistically significant driver gene in the benign tumours, although hotspot *HRAS* mutations were also observed in malignant subtypes, but these mutations occurred at a lower mutation frequency in these smaller cohorts (**Supplementary Table 5**).”

[1.9] In the discussion, lines 491-492, the authors state: “Use of somatic mutational profiling may help in the differential diagnosis of benign versus malignant STs, which can be challenging.” It would be helpful if more clinical context could be provided here, particularly around the standard histopathological diagnostic workflow. What are the gold-standard IHC stains, histopathological correlates, or clinical decision algorithms currently used to differentiate between the four types of sebaceous tumors? A deeper discussion on how mutational testing could provide clinical input would be ideal, given the clinical correlation angle in the discussion. Figure 2 does not clearly identify a mutational profile with high sensitivity or specificity for diagnosis. Could the authors consider using classifiers (e.g., Random Forests) and measure their performance with cross-validation?

We thank Reviewer2 for highlighting the importance of correlating clinicopathological and genetic aspect. Our team includes Professor Thomas Brenn who is a world-leading dermatopathologist and author of several textbook such the WHO blue book for skin and the McKee – Pathology of the skin, and a noted expert in adnexal tumours. At present, the gold standard to make the diagnosis of these sebaceous tumours is based on histology only, there is no core set of tests/IHC stains. Through our paper, we suggested markers/tools to better

detect Lynch syndrome thanks to clinical and histopathological variables (please see "**Figure 1**"). The use of somatic mutation profiling may help in the diagnosis of benign and malignant STs. In the first paragraph of the discussion we say the following:

"Several findings in this study may prove clinically relevant in the diagnosis of ST and LS. Firstly, somatic mutational profiling may help in the differential diagnosis of benign versus malignant STs, which can be challenging. For example, the malignant STs were driven by chromosomal instability and typically showed biallelic *TP53* inactivation, as well as *TP53* mutations co-occuring with *ZNF750* and/or *RB1* mutations. In addition, dMMR SC-E was characterised by mutations in *NOTCH1, RREB1, and DRD5*, whereas SC-O was characterised by significant focal amplifications encompassing *GNAS/SALL4* and *LYN/PLAG1*, significant broad 13q deletions (encompassing *BRCA2* and *RB1*), and 8q amplifications (encompassing *MYC*), with amplification of the *MYC* proto-oncogene previously reported by targeted sequencing and FISH [11]. Lastly, both SC-E and SC-O showed significant amplification of chromosomes 1q21.3 and 20. The former amplification encompasses *HNRN* (Hornerin), which is part of the epidermal differentiation complex on 1q21. Hornerin is expressed by the ocular surface (corneal and conjunctival aspects, including meibomian and Zeis glands), the lacrimal apparatus and the skin, and has antimicrobial and protective (keratinisation) functions [47,48], reinforcing its potential role in sebaceous carcinoma. STs, with the exception of SC-O, can be the first sentinel sign of LS and 3 clinical criteria and 4 histopathological criteria – including (i) the presence of multiple STs, (ii) STs being located outside the head and neck area, (iii) the presence of a LS-associated cancer, in the clinical model, and (i) the absence of epidermal connection, (ii) the delimitation of the ST by a collarette, (iii) a KA-like architecture, and (iv) the diagnosis of SM in the histopathological model, together with loss of MMR expression by IHC – were found to significantly associate with a LS diagnosis (the clinical model being a better predictor) and thus should raise the index of suspicion that could provoke a referral for germline genetic testing. Of note, while the idea of generating a classifier is one we feel is very validate, a comparable dataset of STs to the one we describe in our paper does not exist which makes validating such a tool problematic and thus we feel this analysis is premature. Further, this study highlights the importance of recording the precise location of SC-O tumours, and specifically restricting use of the term SC-O to those lesions arising on the eyelid. Indeed, the sample coming from the canthus showed a different phenotype from the other SC-O and was characterised by a UV mutational signature, rather than being pauci-mutated, the pattern observed in SC-Os."

Mutually exclusive and co-occurring mutations:

[2] The "mutual exclusions and co-occurring interactions" analysis is problematic as it combines the results of a Fisher's test with the output of DISCOVER, a tool specifically developed to replace the inappropriate usage of the Fisher's test in such analyses. The Fisher's test does not control for TMB, leading to a high number of false positive co-occurring interactions (as shown in Figure 2B). In contrast, DISCOVER accounts for TMB in each sample and gene, thus limiting these spurious results. Combining the results from both approaches does not make sense, as exemplified by the contradictions between the interactions reported in Figure 2B and the DISCOVER analysis output provided in Supplementary Table 6 (e.g., significant co-occurrence between ZNF750 and BICRA while Supplementary Table 6 reports significant mutual exclusion). I would suggest that the authors should rely solely on DISCOVER (or similar approaches controlling for TMB), and

completely exclude the Fisher’s test from this analysis.

We thank Reviewer2 for sharing their concerns about the use of Fisher test. As suggested, we have removed this analysis (please see **Figure R13/Figure 2**). We also ran DIALECT (Shuaibi A *et al*. 2024; https://www.ncbi.nlm.nih.gov/pmc/articles/PMC11071465/) on the cohort. The DIALECT software distinguishes between missense and nonsense mutation by design and claims to be superior to DISCOVER. However, even after talking to the authors and re-writing some of their code we were unable to deploy this algorithm.

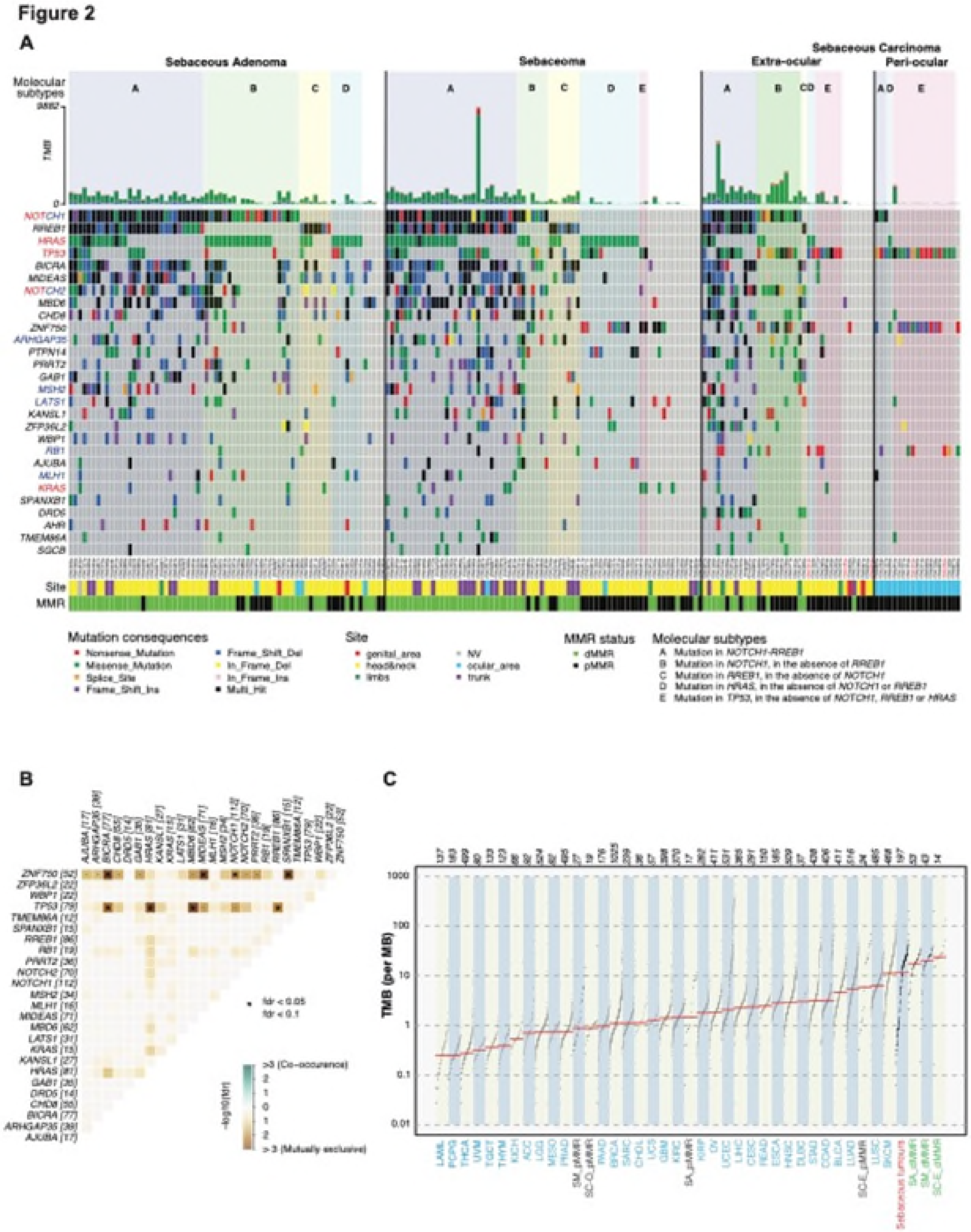

Figure R13/Figure 2 – The somatic mutational landscape of sebaceous tumours across subtypes.

(A) Oncoplot showing somatic mutations in the driver genes of sebaceous tumours (STs). This includes the 25 driver genes identified when taking all four ST subtypes together, and three additional driver genes when considering only specific ST subtypes. Gene names in red denote oncogenes, genes in blue denote tumour suppressors, while genes names in both colours are those reported to have both functions depending on the text.

Patterns of mutations in the top 4 driver genes (*NOTCH1, RREB1, TP53* and *HRAS*) allows division of the STs into five molecular subtypes (A-E) across the four ST subtypes. 'Site' is the body location of the tumour. The 'PD' number is the sample number, with those in red font being from patients that developed metastases. (B) Mutual exclusive and co-occurring interactions based on DISCOVER analysis. (C) Comparison of the tumour mutation burden (TMB) of the sebaceous tumours (STs) against 33 TCGA cohorts (TCGA tumour types are shown using standard abbreviation). The ST cohort are shown all together (blue font), or separated by subtype and mismatch repair (MMR) status (green and red font). Abbreviations: MMR, mismatch repair; dMMR, deficient in MMR; pMMR, proficient in MMR; NV, not available; TMB, tumour mutational burden (in mutations/Mb, including SNVs, MNVs and indels).

Mutational signatures of sebaceous tumours:

[3] The mutational signature analysis was not comprehensively explored and presented, and a global view of the cohort is lacking, specifically related to the relative contribution of the main signatures in each sample. The presentation of the results in Figure S5 show too many signatures simultaneously, and additional figures and analyses would be necessary to support the claims and observations made in the main text and discussion. In my opinion, an improved global view of the mutational signatures would warrant inclusion as a main figure.

We thank Reviewer 2 for their comment. We have log-transformed the data which makes the SBS signatures easier for the reader to see. This figure is available in **Figure R14/Figure 4A** of the revised manuscript as suggested by the reviewer.

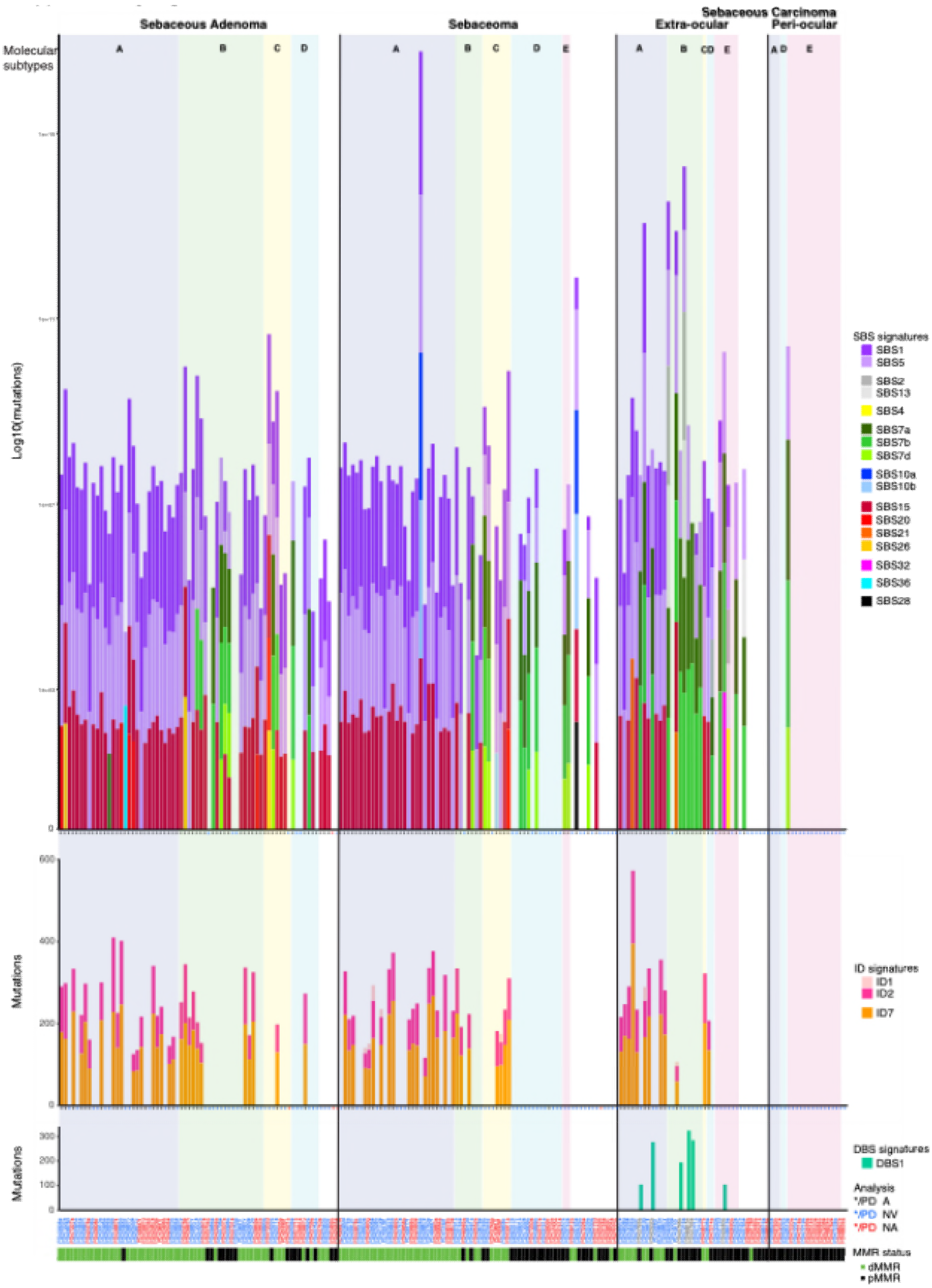

Figure R14/Figure 4A – Mutational signatures of sebaceous tumours. Bar plot showing the mutational signature profiles, including SBS (single-base substitutions), ID (insertions and deletions) and DBS (doublet-base substitutions) signatures, across each of the four sebaceous tumour subtypes. 'PD' numbers refer to the DNA sample identifiers while the asterisk denote samples with/without SBS or ID signatures. A black asterisk or a PD number in black (*/PD A) indicates that a signature was detected in that sample, while blue (*/PD NV) a or red asterisk or PD number (*/PD NA) indicates samples where no mutational signature was detected or samples were

not analysed, respectively. COSMIC signature groups: SBS: clock-like signatures (SBS1 and SBS5), APOBEC signatures (SBS2 and SBS13), tobacco signature (SBS4). UV signatures (SBS7a, SBS7b, SBS7d), *POLE* signatures (SBS10a and SBS10b), dMMR signatures (SBS15, SBS20, SBS21 and SBS26; SBS20 is linked to dMMR and *POLD1* mutation), azathioprine signature (SBS32), *MUTYH* signature (SBS36), unknown signature (SBS28). ID: slippage signatures (ID1 and ID2), dMMR signature (ID7). DBS: UV signature (DBS1).

[3.1] Figure S5 needs clarification. The graphical legend mentions */PD A, */PD NA, and */PD NV in three different colors, but none of these are explained in the textual legend. There is a reference to the blue and red labeling of the sample names, but this is confusing since "blue" samples (no significant signature) actually show some signatures. Additionally, the legend states that "one in red text was not included," but half of the samples are in red.

As suggested by Reviewer 2 we have clarified these symbols (*/PD A, */PD NA, and */PD NV) in the revised manuscript.

Figure 4A – Mutational signatures of sebaceous tumours. Bar plot showing the mutational signature profiles, including SBS (single-base substitutions), ID (insertions and deletions) and DBS (doublet-base substitutions) signatures, across each of the four sebaceous tumour subtypes. 'PD' numbers refer to the DNA sample identifiers while the asterisk denote samples with/without SBS or ID signatures. A black asterisk or a PD number in black (*/PD A) indicates that a signature was detected in that sample, while blue (*/PD NV) or red asterisk or PD number (*/PD NA) indicates samples where no mutational signature was detected or samples that were not analysed, respectively. COSMIC signature groups: SBS: clock-like signatures (SBS1 and SBS5), APOBEC signatures (SBS2 and SBS13), tobacco signature (SBS4). UV signatures (SBS7a, SBS7b, SBS7d), *POLE* signatures (SBS10a and SBS10b), dMMR signatures (SBS15, SBS20, SBS21 and SBS26; SBS20 is linked to dMMR and *POLD1* mutation), azathioprine signature (SBS32), *MUTYH* signature (SBS36), unknown signature (SBS28). ID: slippage signatures (ID1 and ID2), dMMR signature (ID7). DBS: UV signature (DBS1).

[3.2] The method section states that SigProfilerExtractor was used for de novo extraction, considering solutions with 1 to 10 signatures by NMF. However, Supplementary Figure 5 shows 21 signatures (17 SBS, 3 ID, and 1 DBS), and Supplementary Table 8 reports mutation counts for 27 signatures. This discrepancy needs clarification. It is essential to explain the criteria for determining the final set of signatures and how they relate to the initial NMF analysis to ensure consistency and transparency.

We thank Reviwer2 for their comment. For de novo extraction, we ran the analysis using 1 to 10 signatures. However, the next step is signature decomposition and signature assignment. At this step, mutations were assigned to 27 of the 257 signatures in the COSMIC catalogue. SigProfilerExtracter was run in exome mode using GRCh38 as the reference and opportunity genome and 500 replicates for non-negative matrix factorisation (NMF) with 1 to 10 signatures. The solution implemented for downstream analysis was the optimal solution provided by SigProfillerExtractor (referred to as the 'suggested solution'). Results from signature decomposition and assignment were taken forward if the cosine similarity between the de novo extracted signatures and signatures reconstructed from assigned COSMIC signatures was >= 0.9.

The de novo signatures are decomposed and assigned to the known COSMIC signatures, which is why there are more than 10 COSMIC signatures.

[3.3] I strongly suggest the authors include a main figure summarizing the mutational signature analysis. For example, a clustered heatmap of each signature log10 counts (as rows) in each sample (columns) clearly shows the 3 main mutational profiles within this cohort: MMR (SBS15 + ID2 + ID7 + high SBS1 + high SBS5), UV (DBS1 + SBS7a + SBS7b + low SBS1 + low SBS5) and others (low SBS1 + low SBS5). Annotation tracks for samples could include the MMR status, log10TMB, diagnosis and presence/absence of LoF mutations in MSH2 & MSH6. An annotation track for the signatures could inform the reader about their aetiology (UV, MMR etc). Such heatmap can be easily produced with R's pheatmap package and the ward.D2 clustering method. To make it more concise and suitable for a main figure, some highly correlated or same aetiology signatures could be merged (e.g. SBS7a & SBS7b) and low contributing signatures could be dropped and deferred to supplementary figures (if necessary). Alternatively, the authors could use the W matrix from NMF or apply PCA on the 96-SBS mutation proportion matrix to create a scatter plot of all samples colored by cancer types, MMR status and other variables of interest.

We thank Reviewer2 for their suggestion and have drawn the suggested heatmap and added it to **Figure R15/Figure 4B**.

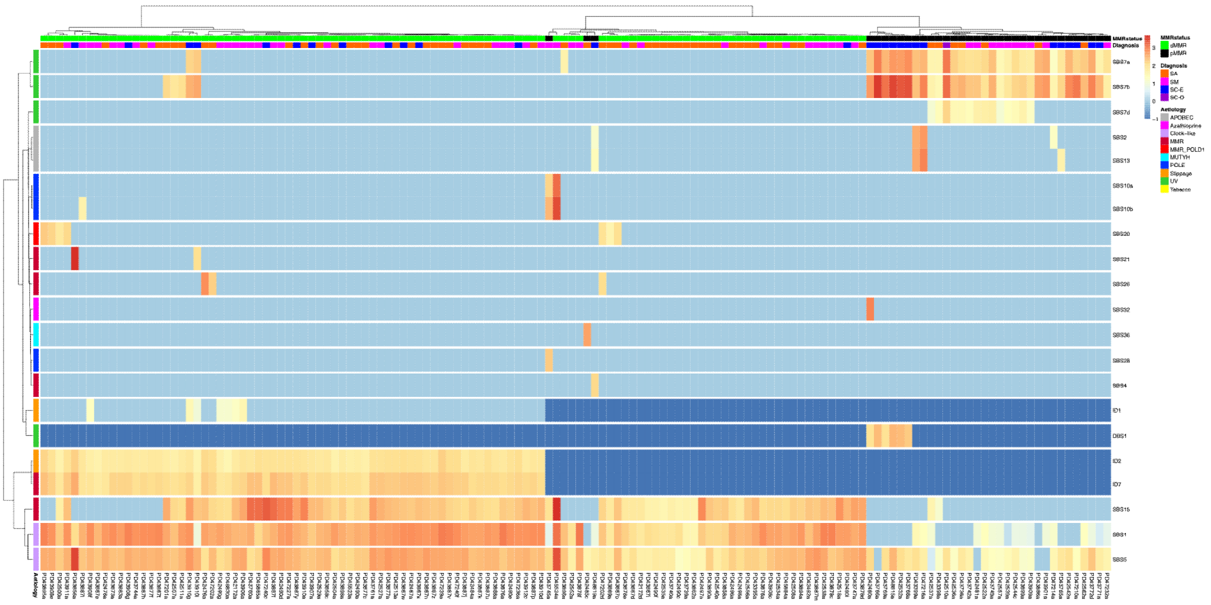

Figure R15/Figure 4B - Clustered heatmap of mutational signatures (log10 counts) in sebaceous tumours. Heatmap showing the mutational signature which tend to cluster by aetiology (left border of the plot). Colour gradient going from high mutations count (in red) to low mutational count (in yellow), to no mutational count (in light blue), to sample not included into the analysis (dark blue). COSMIC signature groups: SBS: clock-like signatures (SBS1 and SBS5), APOBEC signatures (SBS2 and SBS13), tobacco signature (SBS4). UV signatures (SBS7a, SBS7b, SBS7d), POLE signatures (SBS10a and SBS10b), dMMR signatures (SBS15, SBS20, SBS21 and SBS26; SBS20 is linked to dMMR and POLD1 mutation), azathioprine signature (SBS32), MUTYH signature (SBS36), unknown signature (SBS28). ID: slippage signatures (ID1 and ID2), dMMR signature (ID7). DBS: UV signature (DBS1).

[3.4] Lines 268-269, the authors write, “As expected, in the dMMR cases, the high TMB was attributed to dMMR-associated mutational signatures.” What proportion of TMB is attributed to dMMR signatures? Could the authors please include a figure supporting this? It seems that other signatures (SBS1 & SBS5) contribute more to TMB than dMMR-associated signatures, even in dMMR samples.

We thank Reviewer2 for these suggestions and we made a figure supporting what we said (please see **Figure R16/Supplementary Figure 8**).

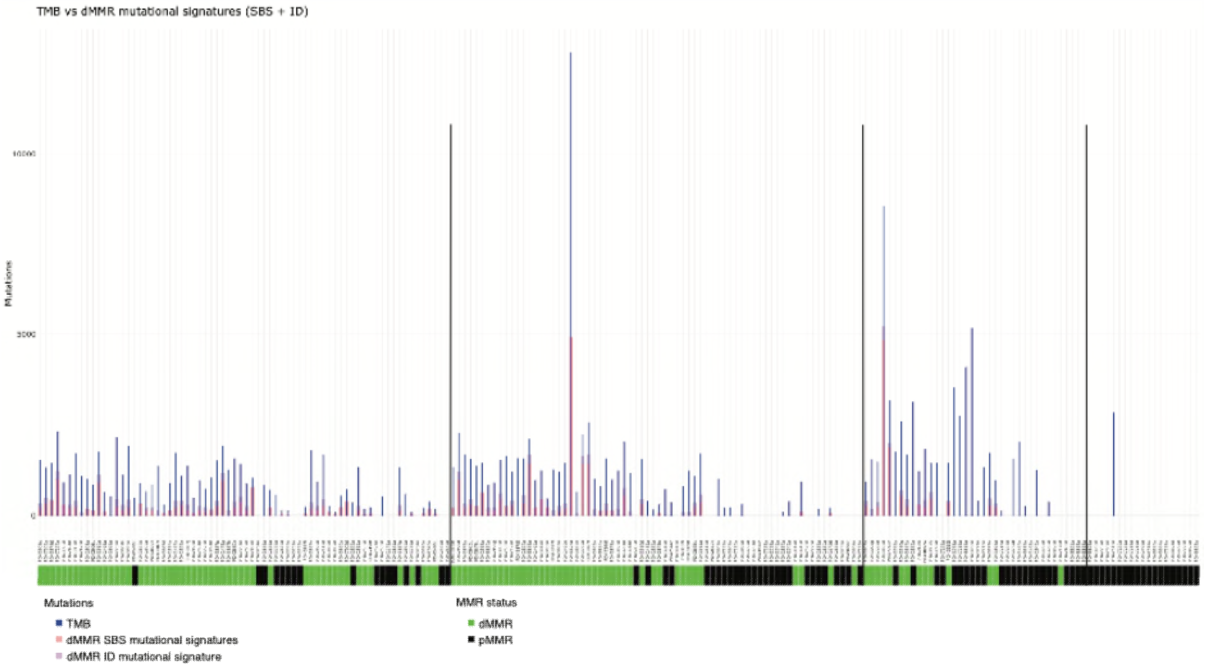

Figure R16/Supplementary Figure 8 – Comparison between TMB, SBS signatures and ID signatures load in sebaceous tumours. Barplot showing the proportion of SBS and ID signatures related to TMB across the cohort.

[3.5] Lines 163-166, the authors write, “Interestingly, indel rate rather than TMB, more accurately predicted MMR status (indel rate p-value < 2.2e-16 versus TMB rate p-value = 2.203e-08, Welch two samples t-test)”. I have a few concerns regarding this analysis. First, the considered data doesn’t meet this test's assumptions about normality. Second, p-values are not an appropriate measure of prediction accuracy. Third, according to the methods section, MMR status was in part determined by the indel rate (lines 772-774), which means using the indel rate to predict MMR status is circular reasoning. These issues should be addressed, for example by comparing ROC-AUCs and and an MMR classification that doesn’t rely on indel rate or TMB.

We thank Reviewer2 for their question. To test whether Indel rate or TMB are a better predictor of dMMR, we have used only samples that were defined as dMMR by having an IHC results suggesting loss of protein expression and also a biallelic alteration of a MMR gene (looking at somatic/germline mutations).

We then used a logistic regression for both Indel Rate and TMB and computed the ROC/AUC using leave-one-out cross validation (please see attached **Figure R17/Supplementary Figure 1A**).

As can be seen, IndelRate is a superior predictor over TMB:

	Indel Rate	TMB
MMR-p vs. MMR-d	0.9989177	0.8869048

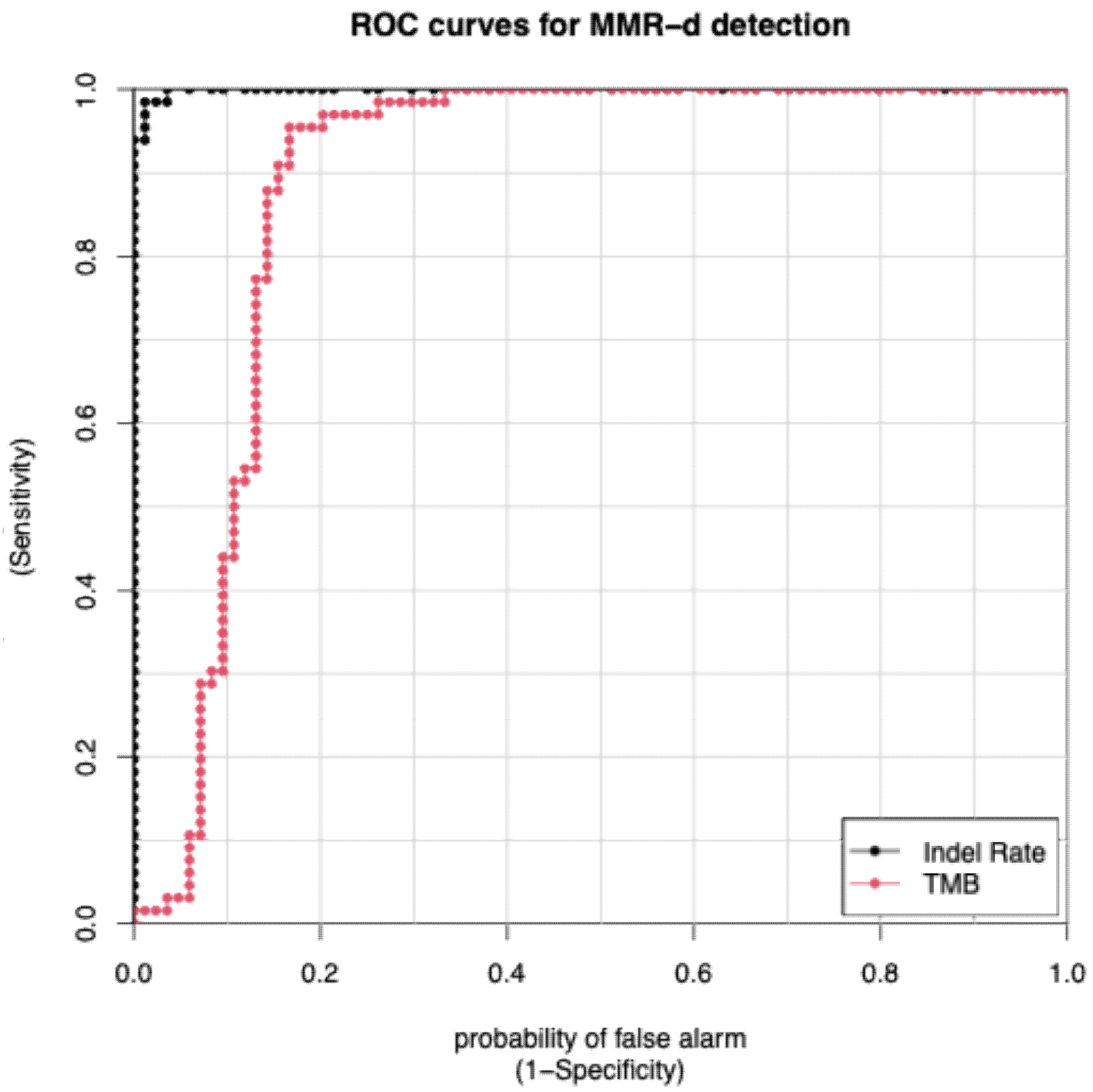

Figure R17/Supplementary Figure 2A - Receiver Operating Characteristic (ROC) curve for dMMR detection. Using logistic regression for both indel rate and TMB, and computing the ROC/area under the curve (AUC) using leave-one-out cross validation to define the best predictor for MMR status (pMMR versus dMMR), the AUC shows that indel rate (AUC = 0.999) is a better predictor than TMB (AUC = 0.887).

Thus, this figure shows indel are a better predictor than the TMB.

[3.6] On lines 501-513, the authors write “this study highlights the importance of recording the precise location of SC-O tumours, and specifically restricting use of the term SC-O for only those arising on the eyelid. Indeed, the sample coming from the canthus showed a different

phenotype from the other SC-O, being characterised by UV mutational signatures rather than being pauci-mutated". Could the authors please include a figure and additional analyses or references supporting this claim, for example by showing differences in mutated drivers or gene expression that suggest it is more similar to SC-E than SC-O?

We thank Reviewer2 for this suggestion. We have made a new figure (please see **Figure R18/Supplementary Figure 9**) including SC-Es from the head and neck only, and the SC-Os, and the case from the canthus. We can easily see that the sample from the canthus shares mutations only found in SC-E such as *CHD8, LATS1* and *TMEM86A*, as well as having a UV SBS mutational signatures.

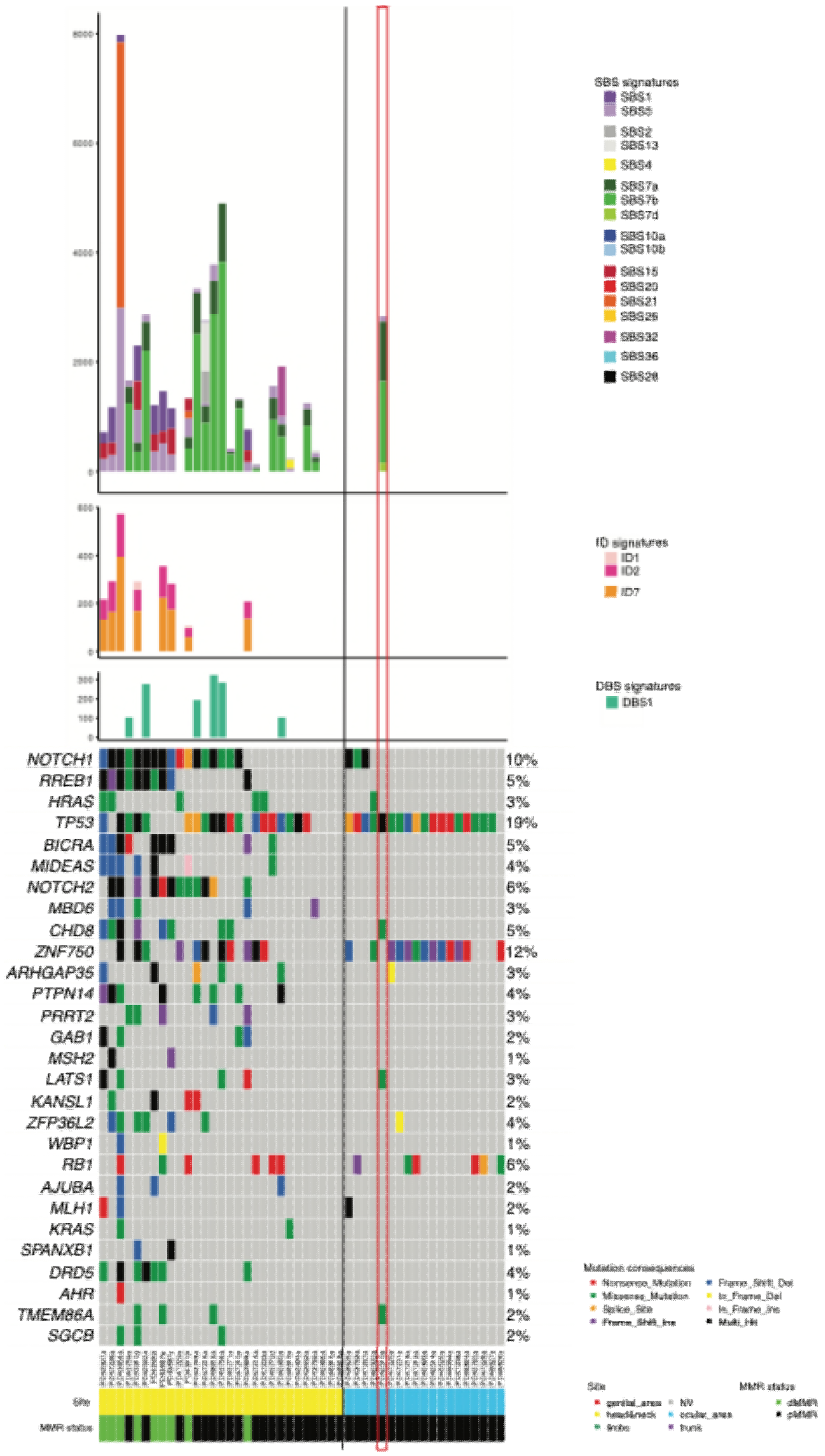

Figure R18/Supplementary Figure 9 - Mutational profile of SC-Es located on head and neck, and SC-Os. Comparison of SC-Es versus SC-Os across mutational signatures (A) and somatic mutations of drivers genes (B).

[3.7] Lines 1377-1379: “SBS” should be “DBS”. Also, a typo in “mutationa”.

We thank Reviewer2 for pointing out these typos, which we have fixed (please see revised Manuscript).

[3.8] In some other cancers, MMR status is linked to survival (e.g. Endometrial). Did the authors investigate whether in this cohort, MMR status is linked to survival in SC regardless of subtype? Authors note that sample size in SC was too small to measure but it may be helpful for readers to see this information anyways even if not statistically significant.

We thank Reviewer2 for this suggestion, however, only 4 patients in our cohort died from sebaceous tumours, of whom 2 metastatic SC-E, 1 metastatic SC-O and 1 SC-O with no reported metastasis where the patient died 4 months after diagnosis. Thus, we think a survival curve is therefore potentially misleading (please see **Figure R19**).

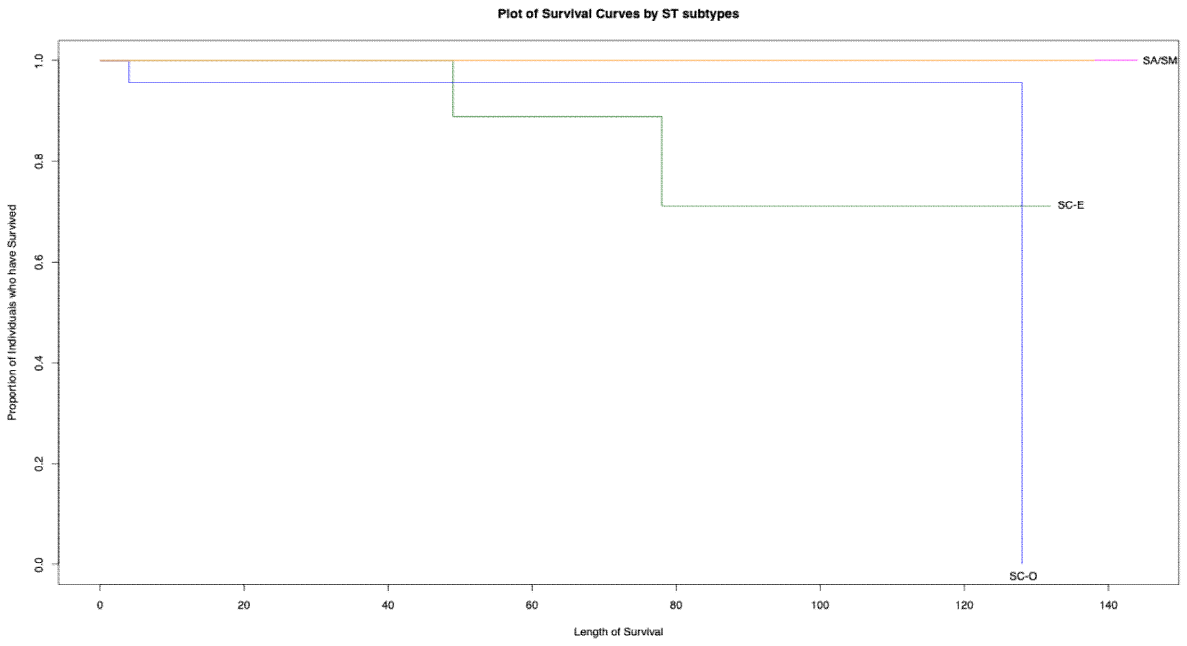

Figure R19 – Survival curve in sebaceous tumours.

Integrative cluster analysis:

[4] The results of this analysis are rather cryptic, and the methodological details raise concerns about their validity.

We agree that the integrative analysis was not properly described in the manuscript. In particular, we focused only on the clustering of tumours instead of the identification of molecular drivers, which is the main goal of this type of methods. We have now run two different methods: iCluster (https://doi.org/10.1093/bioinformatics/btp543) and MOFA (https://doi.org/10.15252/msb.20178124), both of them based on latent variable analysis and performed a comprehensive study on the robustness of the results. In the end, we decided to

use MOFA, as the fitting process is more robust, and the results were easier to interpret. We must note that the goal of this method is not clustering, but identification of molecular drivers. We have included these data in the revised manuscript along with text and figures to explain the results.

First, the expression data was combined with the mutation profiles of approximately 80 genes selected on the basis that they are frequently mutated in this cohort. Given that a typical tumor contains fewer than 10-15 driver mutations (referring here to Martincorena et al, 2017) and that these sebaceous tumors have huge TMBs (nearly half of them have a thousand mutations or more), we can be sure that most of the mutations in these 80 genes are passenger mutations. While a few relevant genes such as TP53 and MSH2 have been manually added and others like TTN have been manually removed, Supplementary Figure 4 suggests that the limited number of potentially meaningful mutations is still overwhelmed by the number of passengers.

The reviewer raises some interesting points. Indeed, the main goal of our integrative clustering is not so much to group tumours but to identify molecular driver events. To do that, both iCluster and MOFA methods identify sets of mutations and expression changes that co-occur in subsets of tumours. As both methods are computationally demanding, our first step in this analysis was feature selection. In the case of mutations, the reviewer is right that some of these tumours have a high mutational burden. We tried to identify a relatively small subset of genes that were potentially interesting, setting up filters based on the minimum number of samples that carry mutations. As the reviewer states, some of these could be passengers. We have added an additional filter testing for association of the mutation and the total mutational burden, removing those that were statistically significant. With this filter, we expected to remove those genes that were probably passengers. We also added manually some well-known driver genes. Of course, our final set of 84 genes might still contain some passenger genes, but both iCluster and MOFA will select only those that contribute to any driver event, as they contain regularisation to perform variable selection, removing uninteresting genes. In the case of expression, we used the 1,000 most expressed genes plus the genes present in the selected mutations.

Second, the method contains many steps with somehow arbitrary choices (e.g., a first selection step out of 16 fitted models followed by a second selection step for the number of k-means clusters), making it very difficult to assess the robustness of the findings. What happens if we use another fitted model (many had a similar balance of variance explained and a low number of clusters)? What happens if a different k is chosen or if we change the gene sets?

The optimal selection of the number of components is a well-known problem in Latent Variable Modelling and Cluster Analysis, and although several methods have been proposed, they often do not agree on the best solution. Starting with the selection of latent dimensions (that is, molecular drivers), MOFA is quite robust and in fact it recommends using the default number of 15. We checked the robustness of the models comparing the total variance explained and the minimum variance explained by any of the factors when we fitted a different number of latent factors in the model. As the plots below show, after 15 factors the increase in variance is minimal, therefore we used that value.

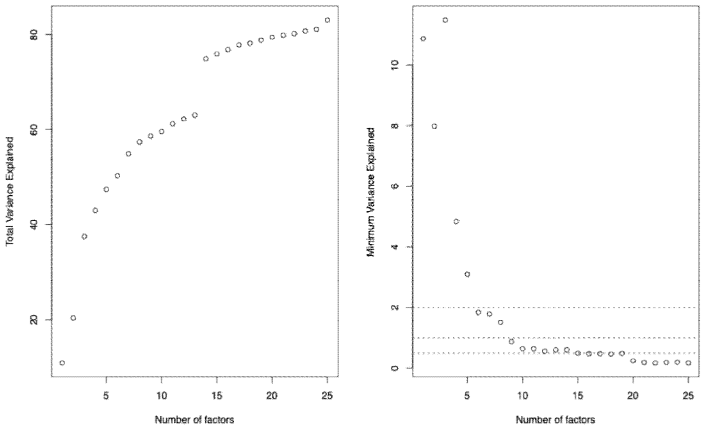

We also inspected the robustness in the model fitting using the recommended procedure in the vignette. We fitted our MOFA model using different random seeds and checked how similar the results were comparing the correlation of the factors obtained in each fit. We finally selected the best model.

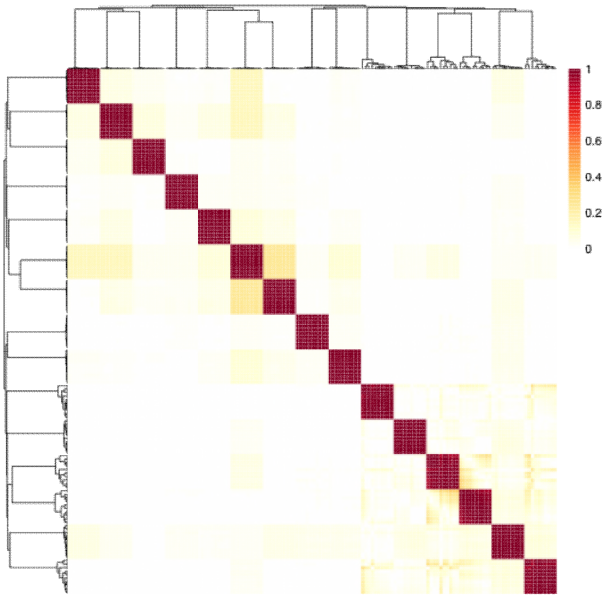

It is also easy to quantify how much variance from each data type the latent factors explain, in order to focus on the most interesting drivers:

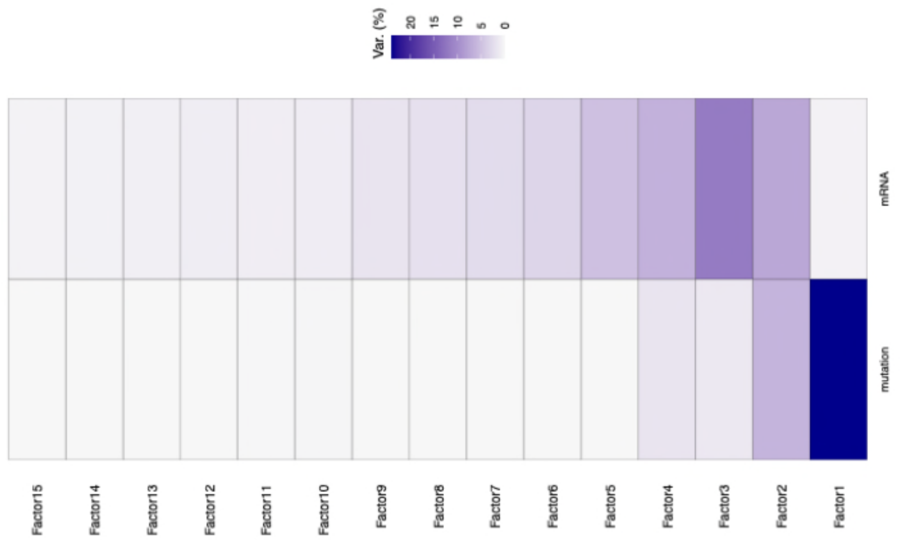

If we set up a threshold of minimum variance at 1%, we can retain for further study the first 10 latent factors. Indeed, they are the ones that contribute most to the tumours.
These drivers are characterised by both mutations:

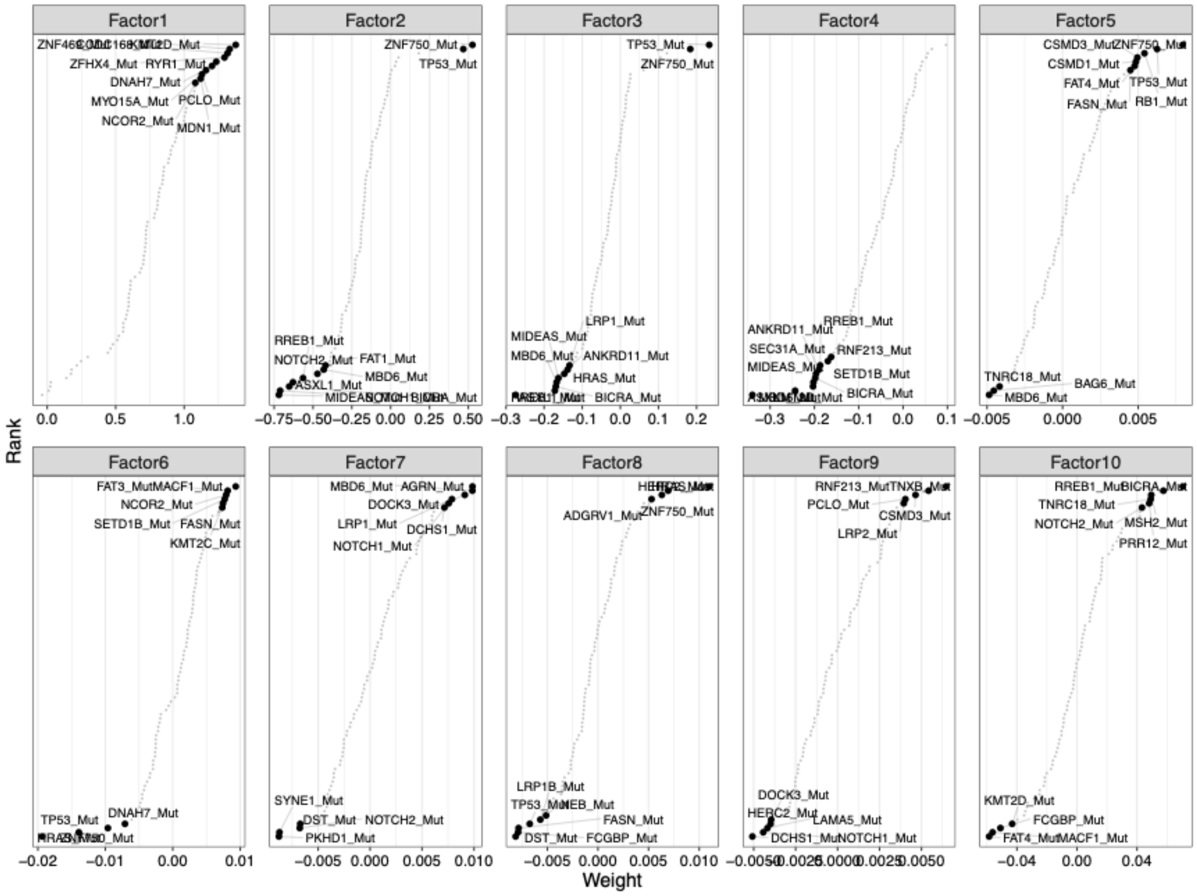

And gene expression:

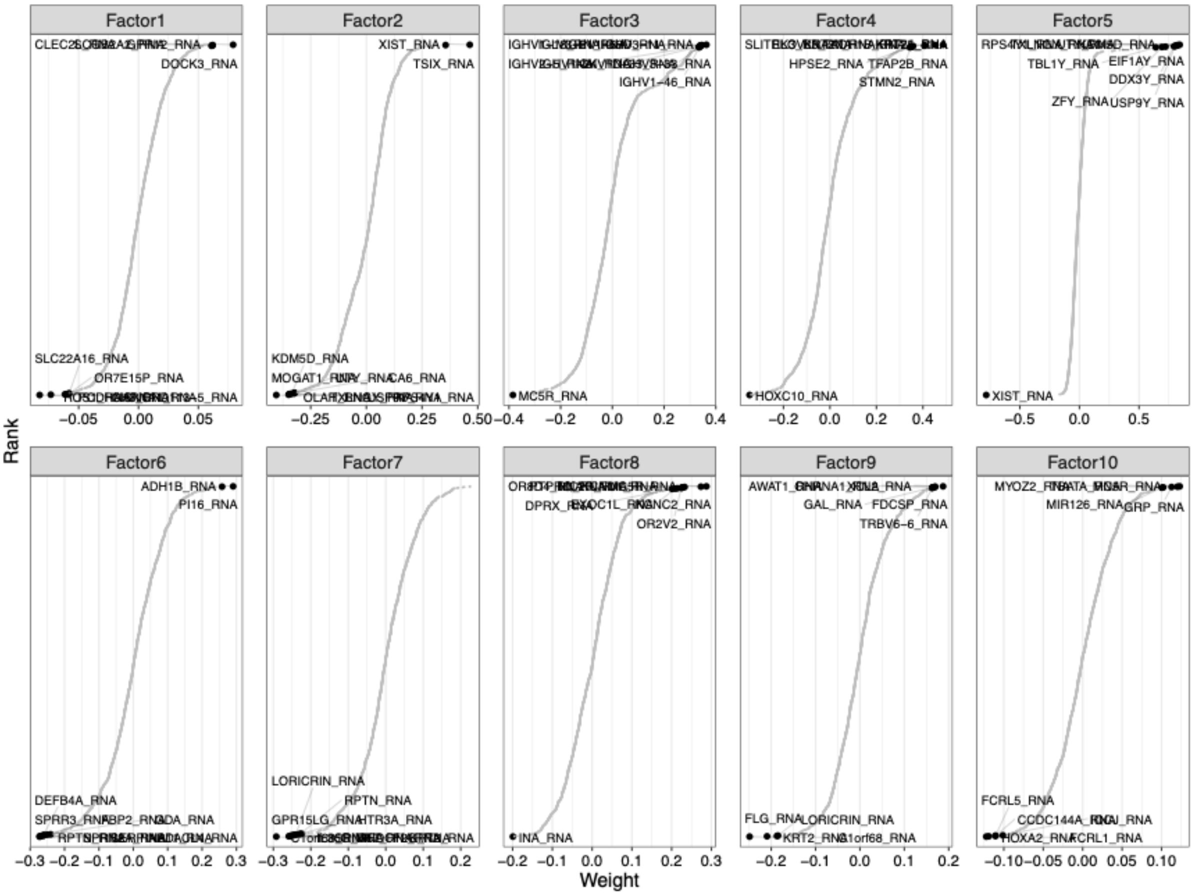

These plots show the weights of each feature in each latent factor. The larger (or smaller) the x-value (weight), the more important the feature is in defining that latent factor. For example, having mutations in *TP53* or *ZNF750* will produce a high score in Factor 3. Looking at those weights we can also see that Factor 1 is explained mostly by mutations, and not that much by expression. In fact, that factor explains 23.8% of the mutation data and only 0.54% of the expression. It reflects high mutational burden, as all mutations have a similar positive score.

The following heatmap shows the weights of each molecular driver (latent factor, columns) in each tumour (rows):

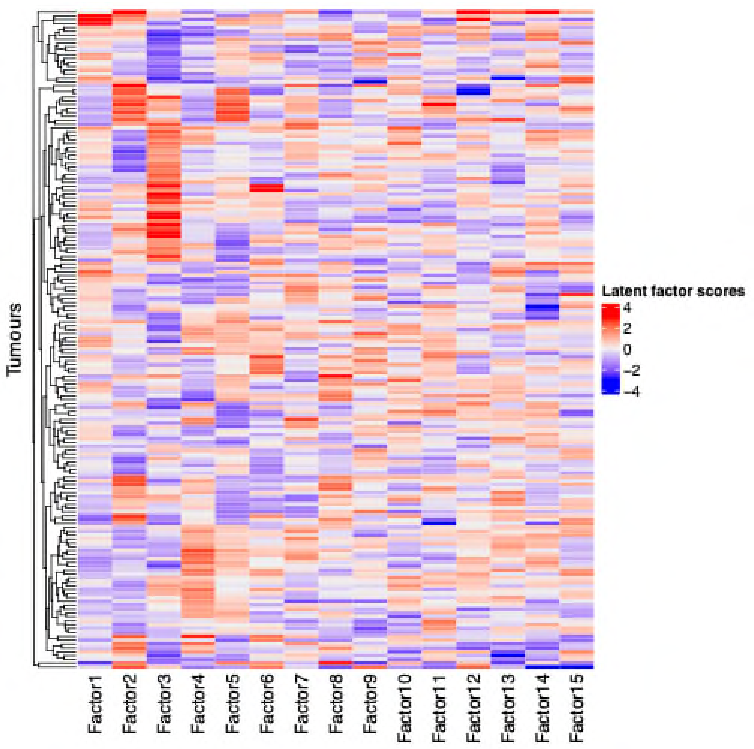

As we can see each tumour presents a combination of these drivers, therefore it makes sense to try to cluster tumours based on their score. Using the top 10 factors we identified two potential solutions with 7 or 9 subgroups based on the silhouette index. These solutions were almost identical; the two new clusters were partition of the first and the third cluster. The following heatmap allows us to compare and choose the best partition. In fact, 9 clusters seem to identify better the subgroup of tumours that have high scores in the first factor (high mutational burden) and splits the third cluster into a group with high scores of factor number 4. We kept the solution with 9 integrative subgroups.

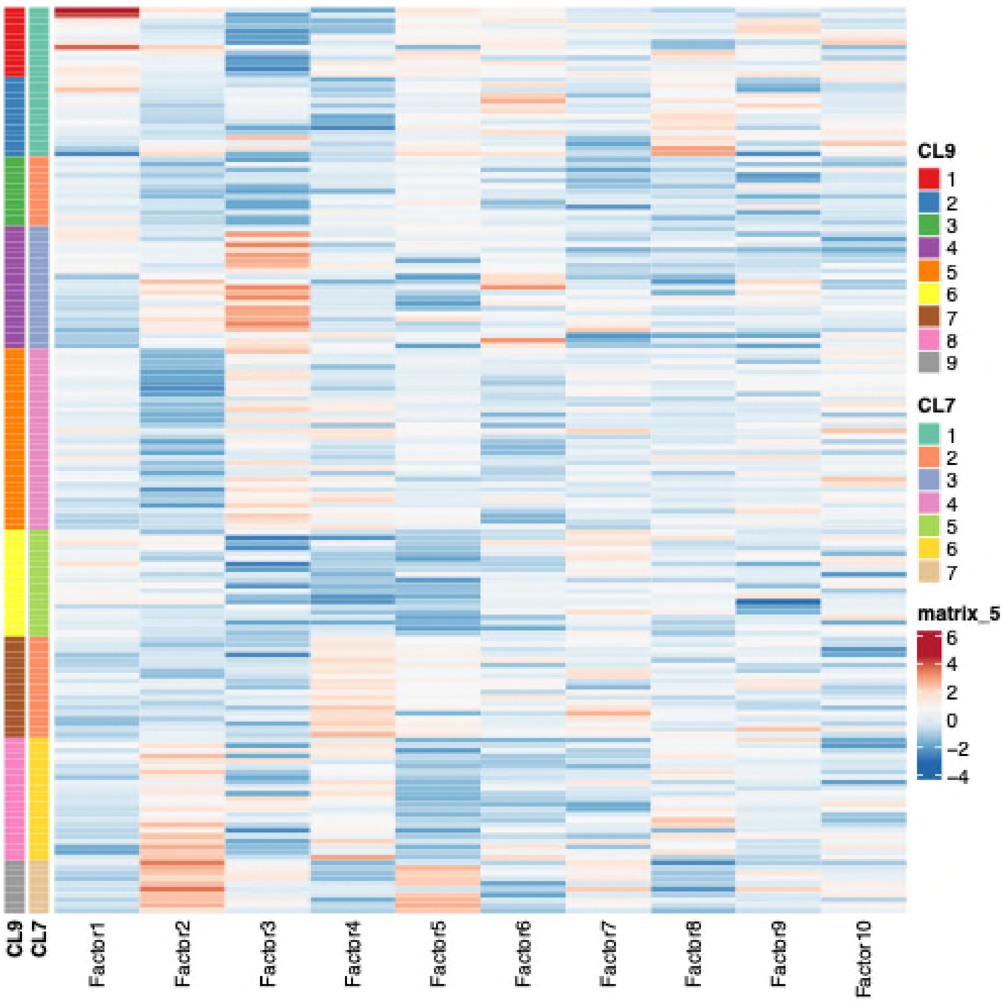
CL9
1
2
3
4
5
6
7
8
9
CL7
1
2
3
4
5
6
7
matrix_5
6
4
2
0
−2
−4
CL9
CL7
Factor1
Factor2
Factor3
Factor4
Factor5
Factor6
Factor7
Factor8
Factor9
Factor10

We describe now the 9 molecular subgroups:

For each integrative cluster (IntClust 1 to 9), the following data will be available:

1) (n=): the number of samples included into this IntClust;
2) Subtype: which tumour subtype is mostly included into the IntClust;
3) MMR: which MMR status represents the most the IntClust;
4) Indel and SNV rate: which compare the mentioned IntClust against all the others;
5) Proportion of cells: which compare the same cell type across the different Cluster and not the comparison between different cell type in a same Cluster;
6) Proportion of mutated tumours: which proportion of samples in this IntClust are mutated for a specific gene;
7) Pathway activation: activity of specific pathway in this IntClust;
8) Copy number aberration: CNA profile of the IntClust.
9) Mainly SC-O tumours.

IntClust 1(n=13 tumours):

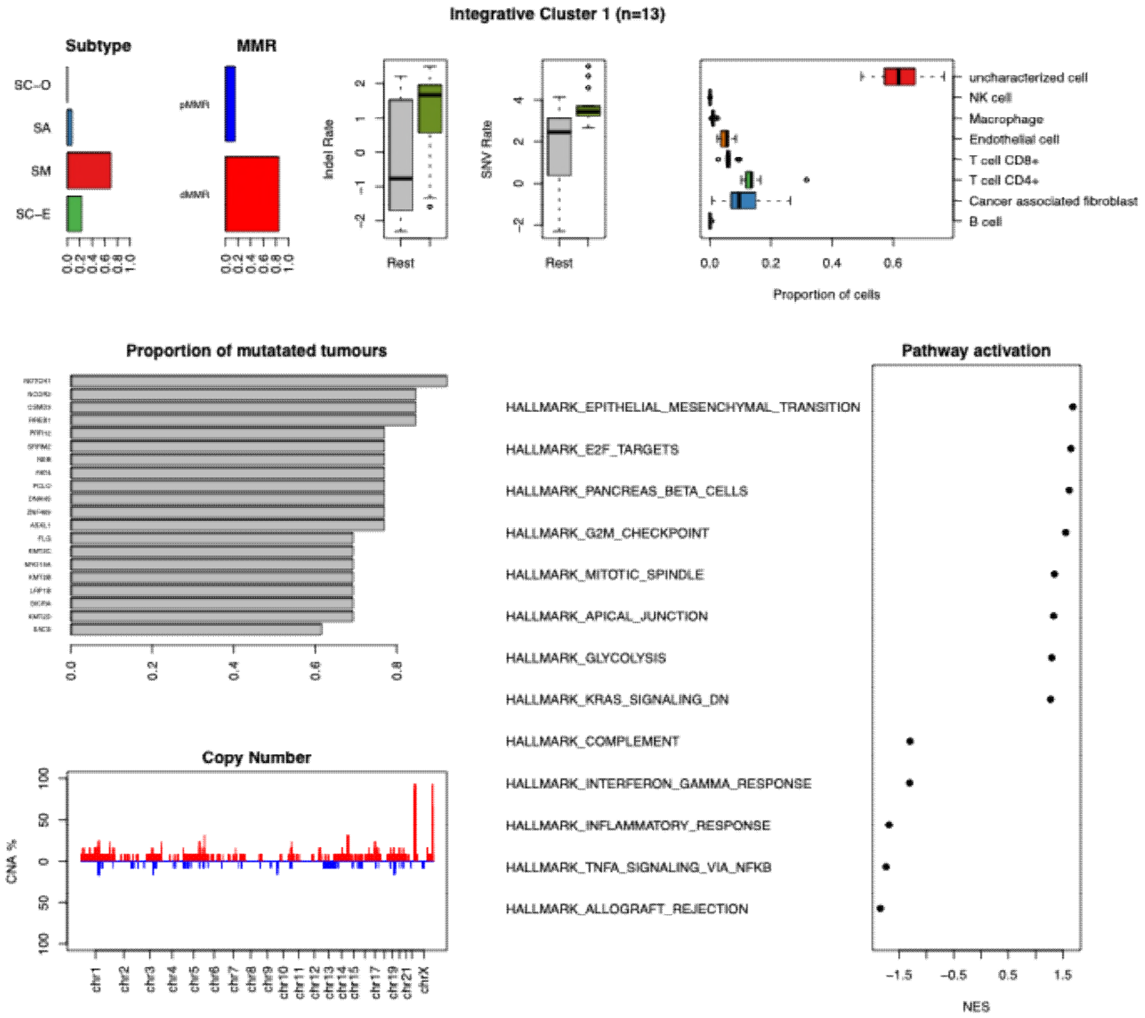

A mutational-burden driven subgroup, mostly SM tumours with MMR deficiency and up-regulation of G2M checkpoint and E2F targets. High proportion of CD8+ T cells and fibroblasts.

IntClust 2(n=15 tumours):

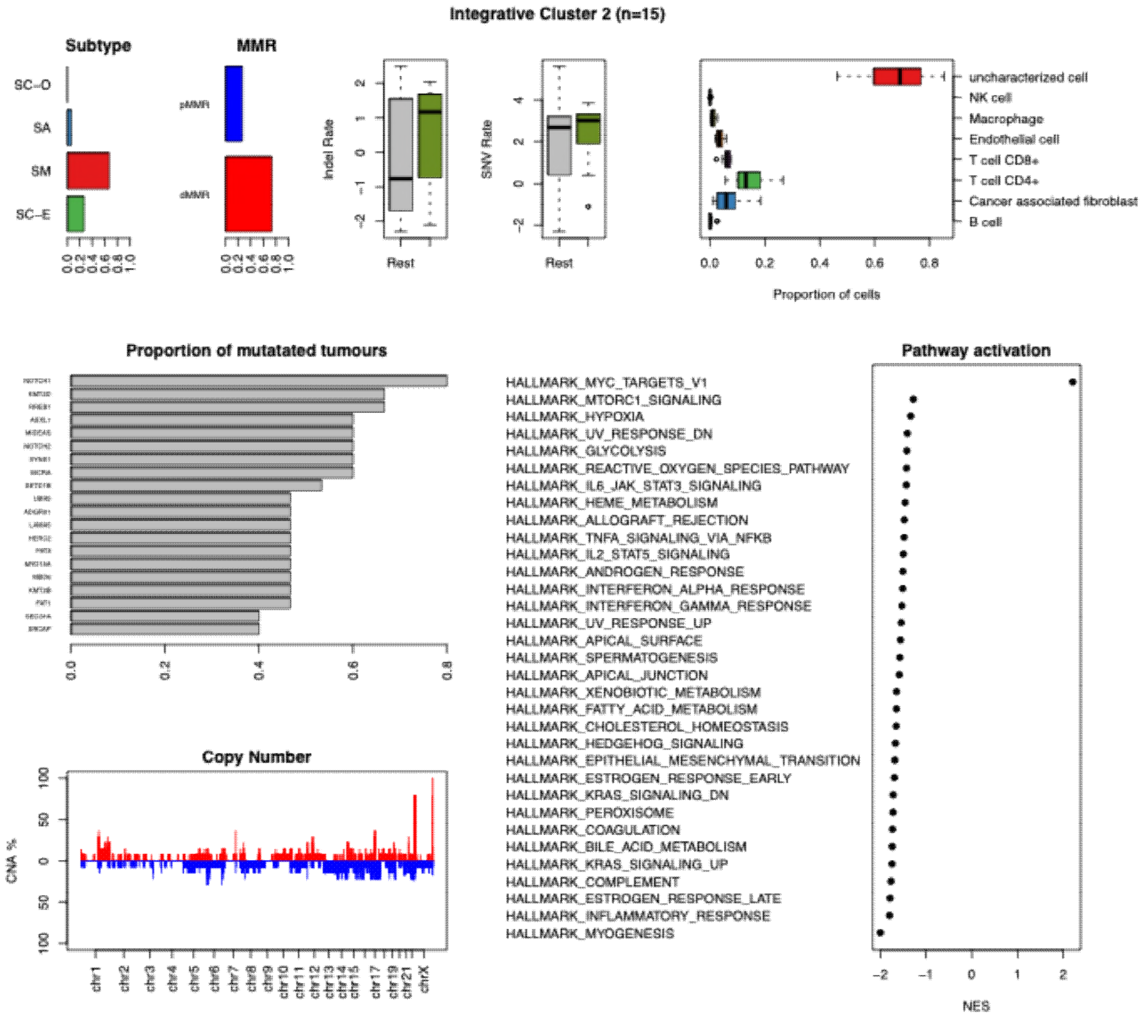

Indel-driven cluster, but with lower indel levels. Mostly SM tumours. dMMR, MYC up-regulated and down-regulation in interferon response. High proportion of CD8+ T cells.

IntClust 3(n=13 tumours):

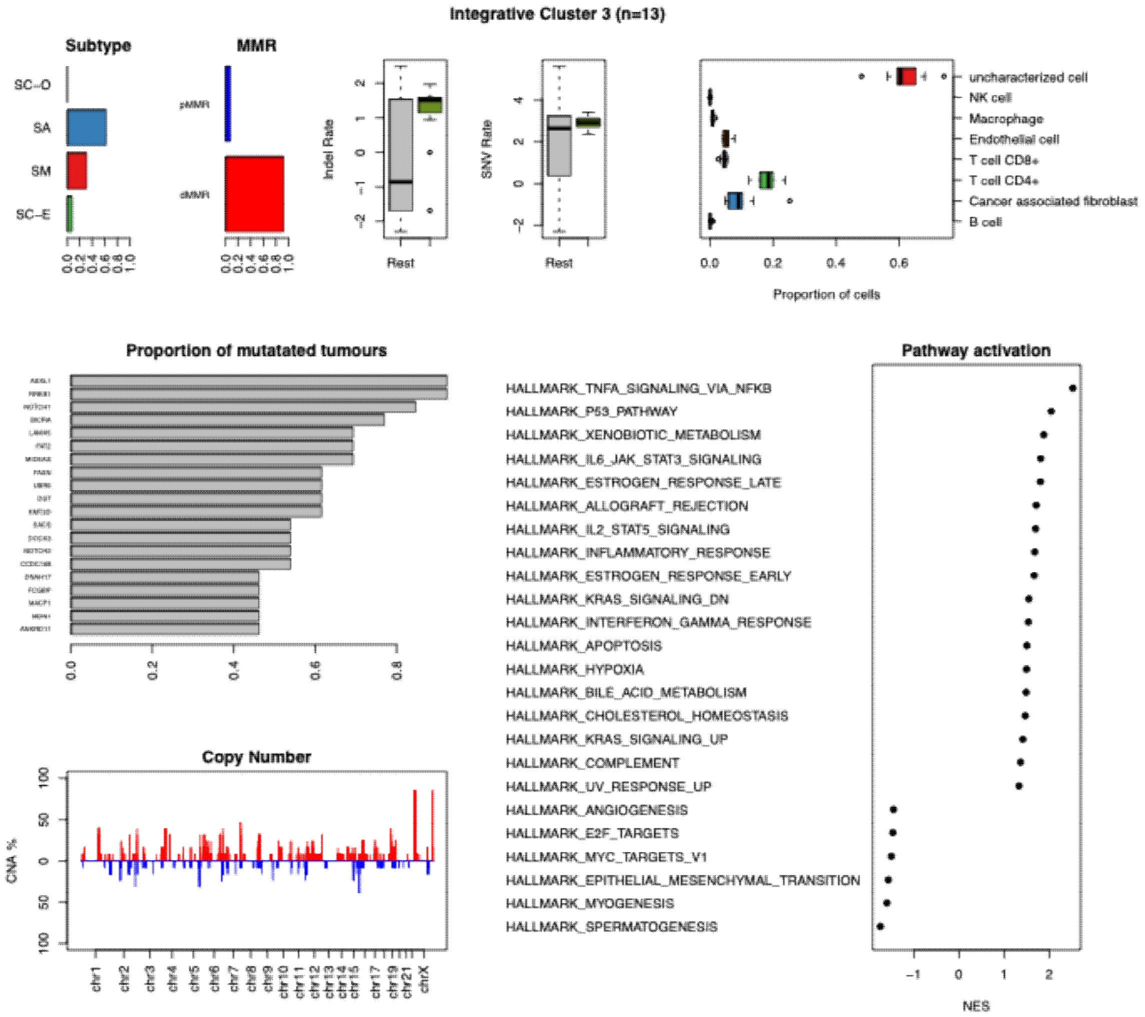

High indel burden, *NOTCH1* and *RREB1* mutations, dMMR but down-regulation of most hallmark pathways. High proportion of CD4+ T cells.

IntClust 4(n=23 tumours):

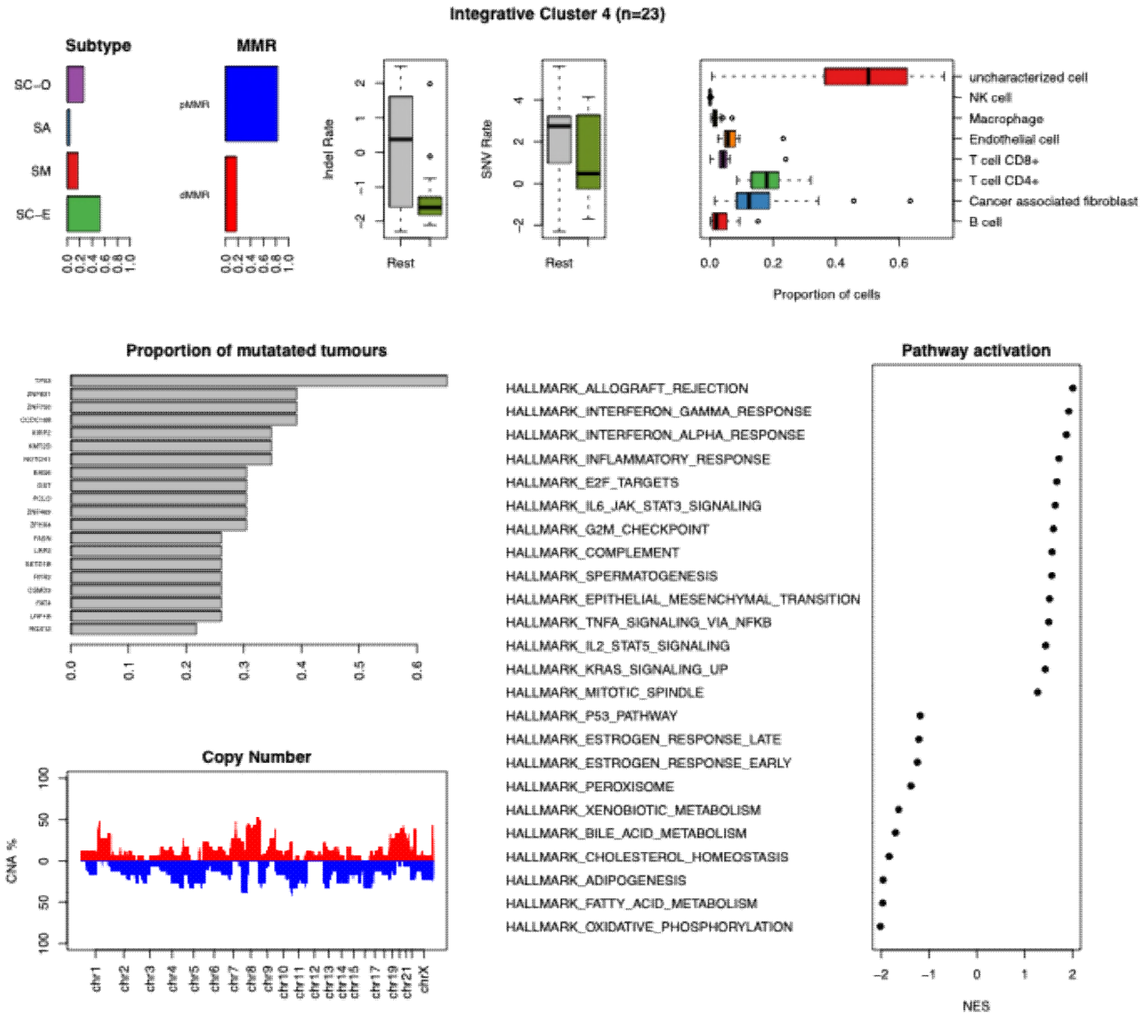

Lower mutational burden, but large proportion of *TP53* mutations. Many copy number aberrations and upregulation of interferon response. pMMR. High immune population (B cells and T cells in particular)

IntClust 5(n=34 tumours):

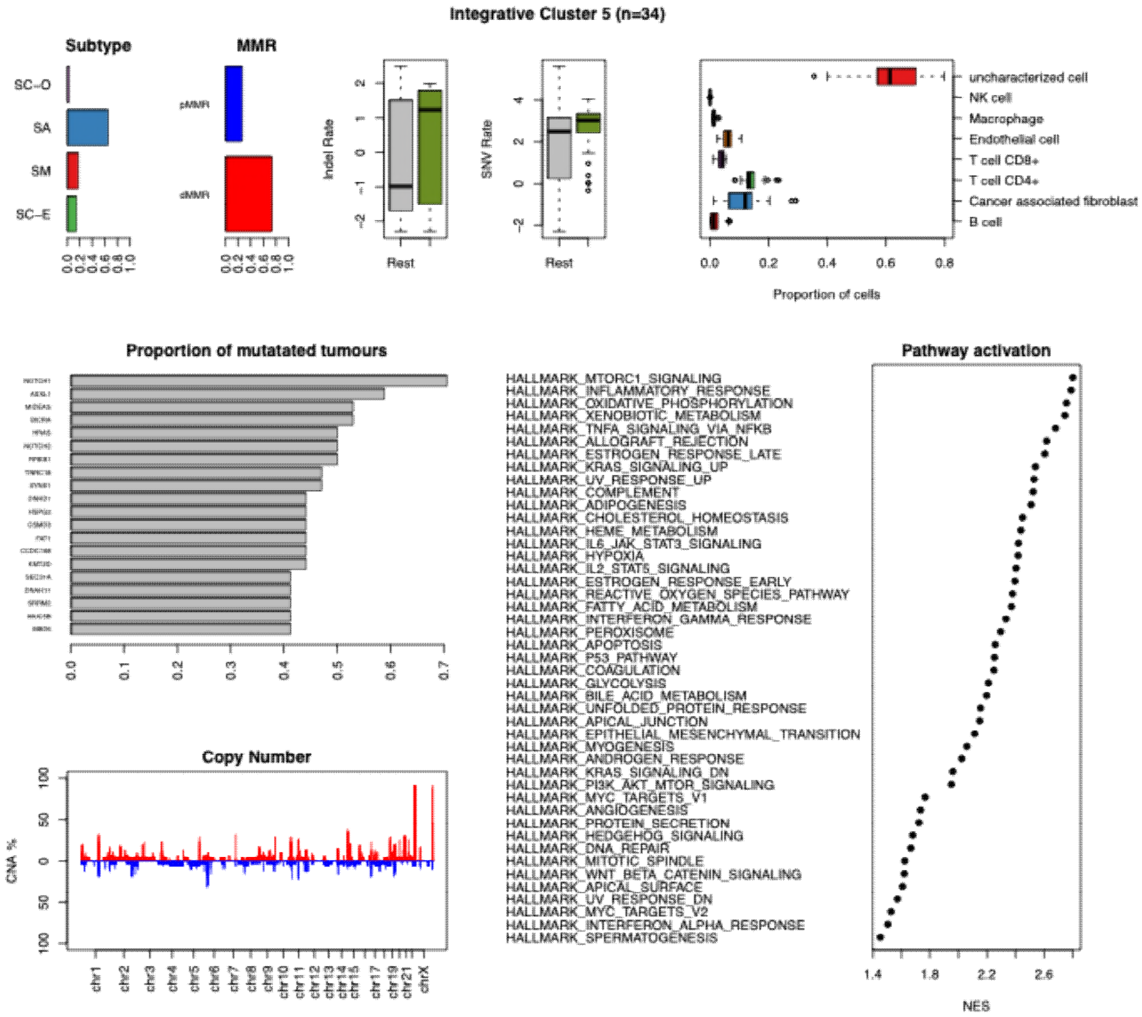

Mostly SA tumours with high mutational burden. Few copy number aberrations and many up-regulated pathways though. High B cells proportion.

IntClust 6 (n=20 tumours):

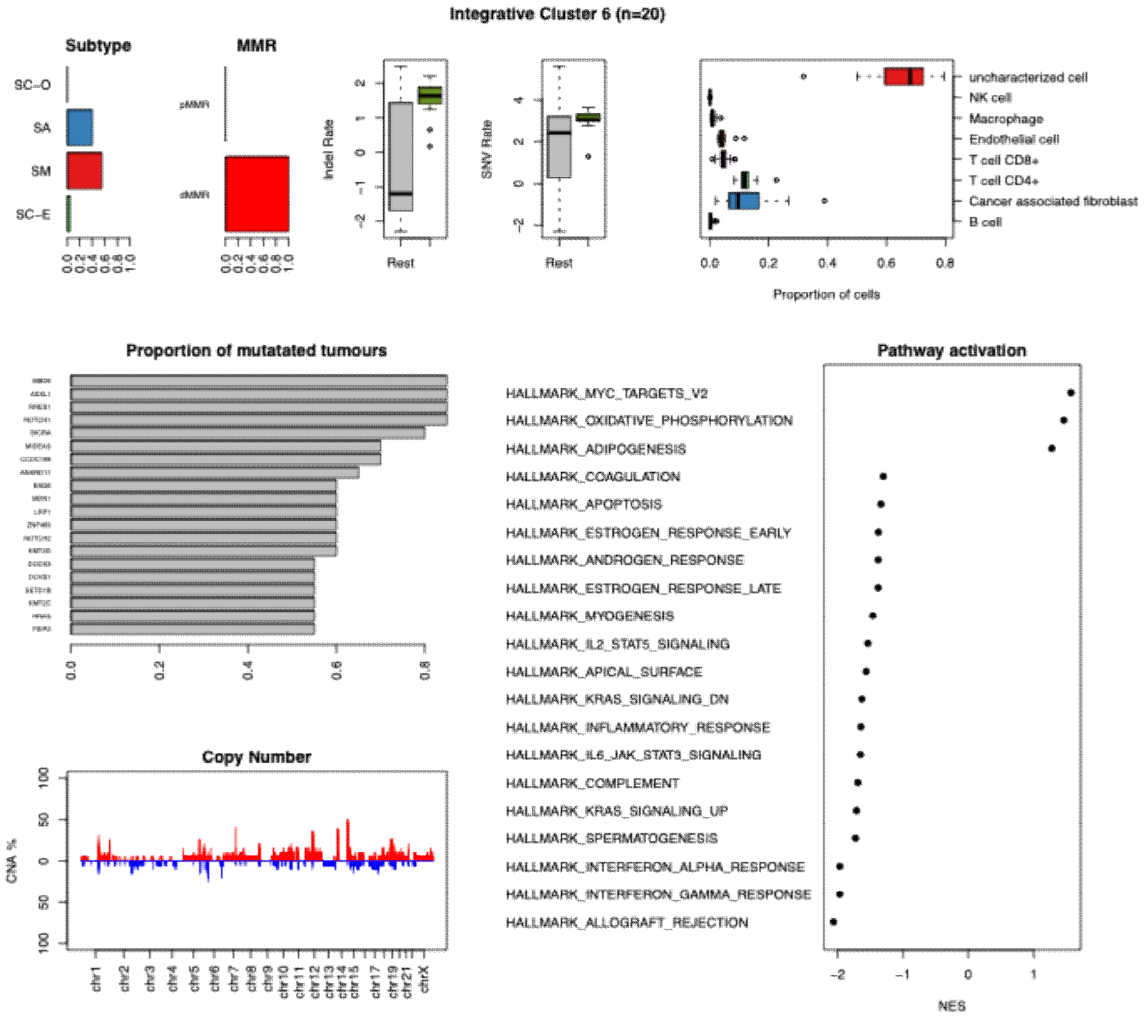

dMMR tumours with high mutational burden. Upregulation of MYC pathway.

IntClust 7 (n=19 tumours):

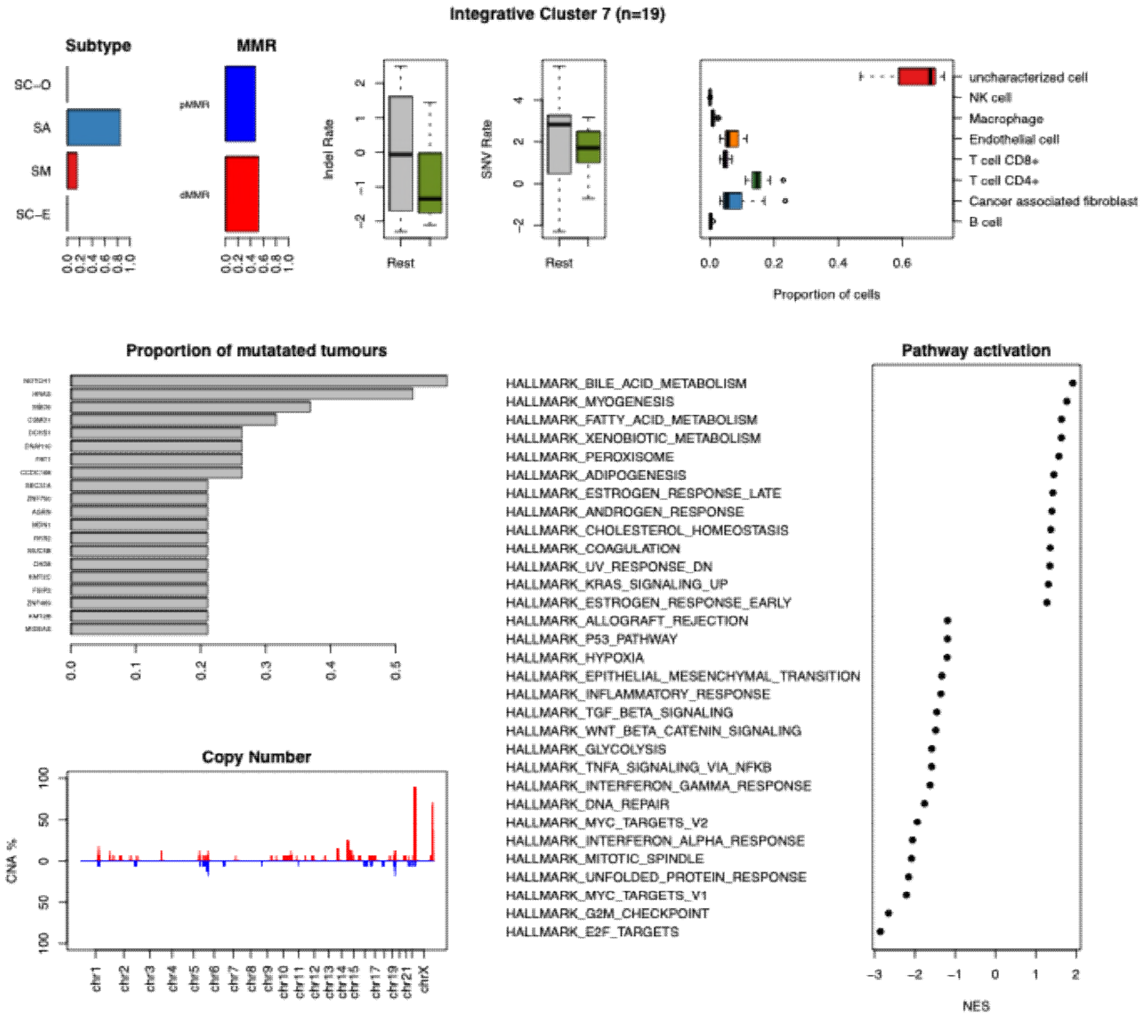

SA tumours mostly with lower mutational burden, however representation of mutations in *NOTCH1* and *HRAS*. Copy number aberrations are absent. Down-regulation of interferon pathways. High proportion of endothelial cells and fibroblasts.

IntClust 8 (n=23 tumours):

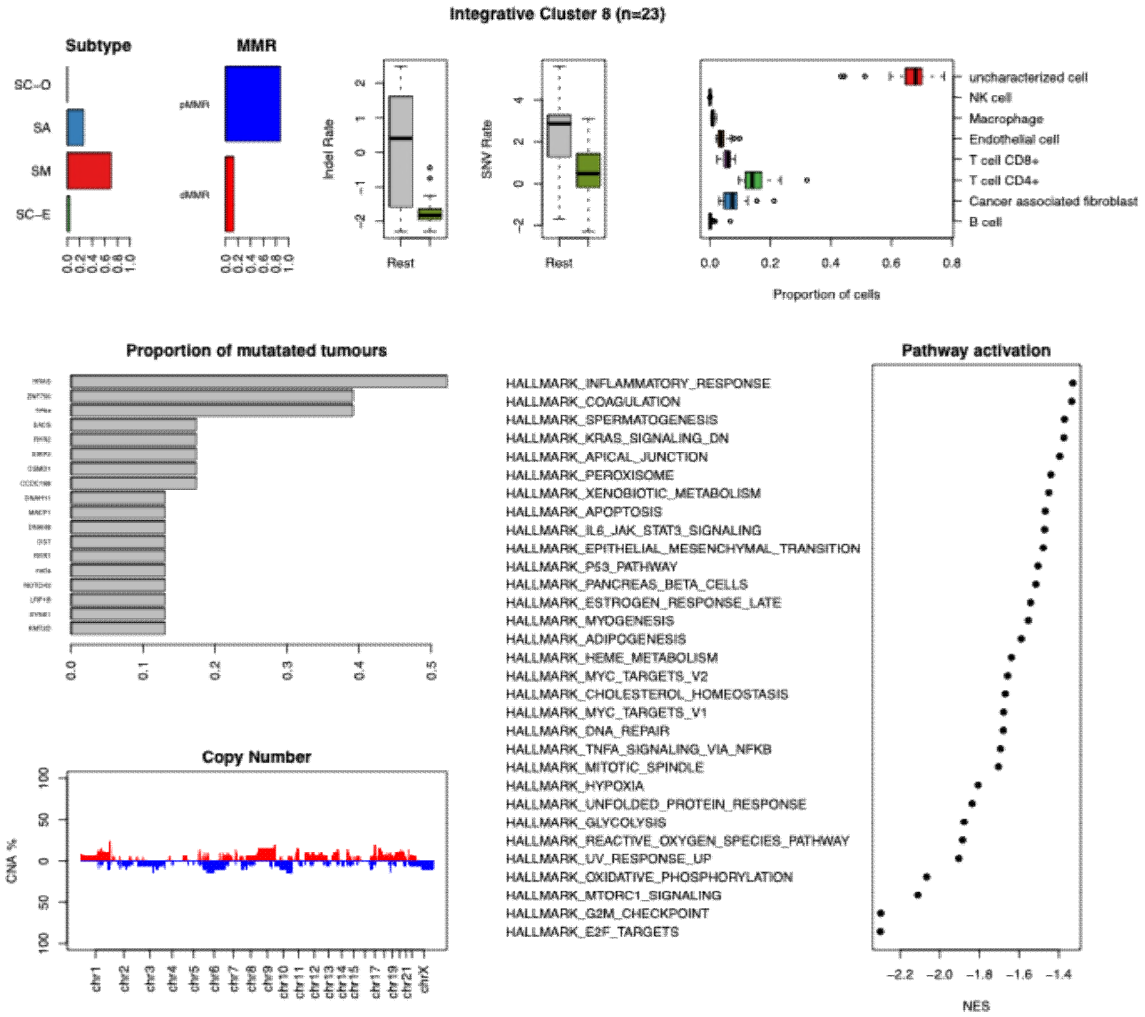

Mostly SM and SA tumours, low mutational burden but *HRAS* and *ZNF750* mutations. pMMR and many pathways down regulated.

IntClust 9 (n=10 tumours):

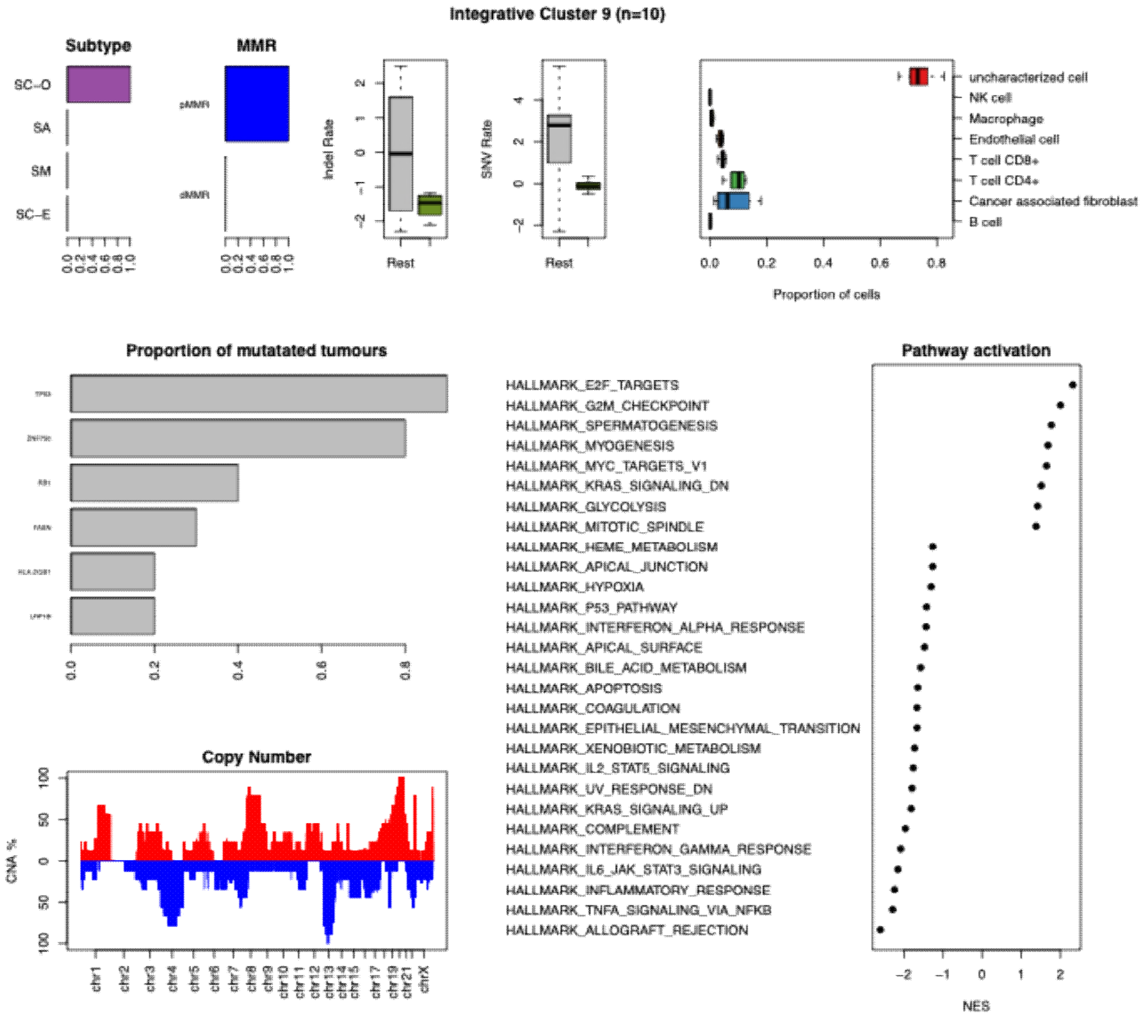

Group comprised exclusively of SC-O tumours, pMMR and low tumour burden. However, almost all of them have mutations in *TP53* and *ZNF750*. Upregulation of E2F and G2M checkpoint pathways. Lower immune component. Many copy number aberrations.

We also compared this clustering with the iCluster analysis that we presented in our previous version of the manuscript. We saw a moderate agreement between the two partitions (adjusted Rand index=0.43):

	1	2	3	4	5	6	7	8	9
1	0	11	4	1	10	4	0	0	0
2	0	0	1	1	2	0	17	0	0
3	1	0	0	1	0	7	1	7	0
4	0	0	0	8	0	0	0	3	0
5	0	0	0	0	0	1	0	13	0
6	1	0	0	1	0	0	0	0	10
7	0	0	0	2	22	0	0	0	0

8	11	2	8	0	0	0	1	0	0
9	0	0	0	0	0	8	0	0	0
10	0	2	0	9	0	0	0	0	0

I should note, even if the authors are able to address the concerns described above, and although I appreciate these exploratory types of integrative analysis, I would argue they cannot replace the more well-established analyses that many readers would expect, if only for clarity and easier interpretation of the results.

We agree with the reviewer that this is a complementary analysis that offers an alternate view of the biological features of the disease. In this study we use many different pieces of information that need integration, and we thank the reviewer for the suggestion of comparing this analysis with a purely transcriptomic clustering approach.

In the revised manuscript we provide the expression clustering analysis described below.

In other words:
[4.1] How many (purely) transcriptional groups seem to be there? If the exact number is hard to determine, the smallest number that make sense (visually) should be the starting point.

Using the most 5,000 variable genes, the Euclidean distance as the dissimilarity matrix and the Ward hierarchical clustering method on the z-scale gene expression matrix, we identified 6 transcriptomic clusters. The optimal number of clusters was selected based on the silhouette measure. We tried several options for the dissimilarity and clustering method and found quite consistent results. The following heatmap shows the expression patterns of the six groups.

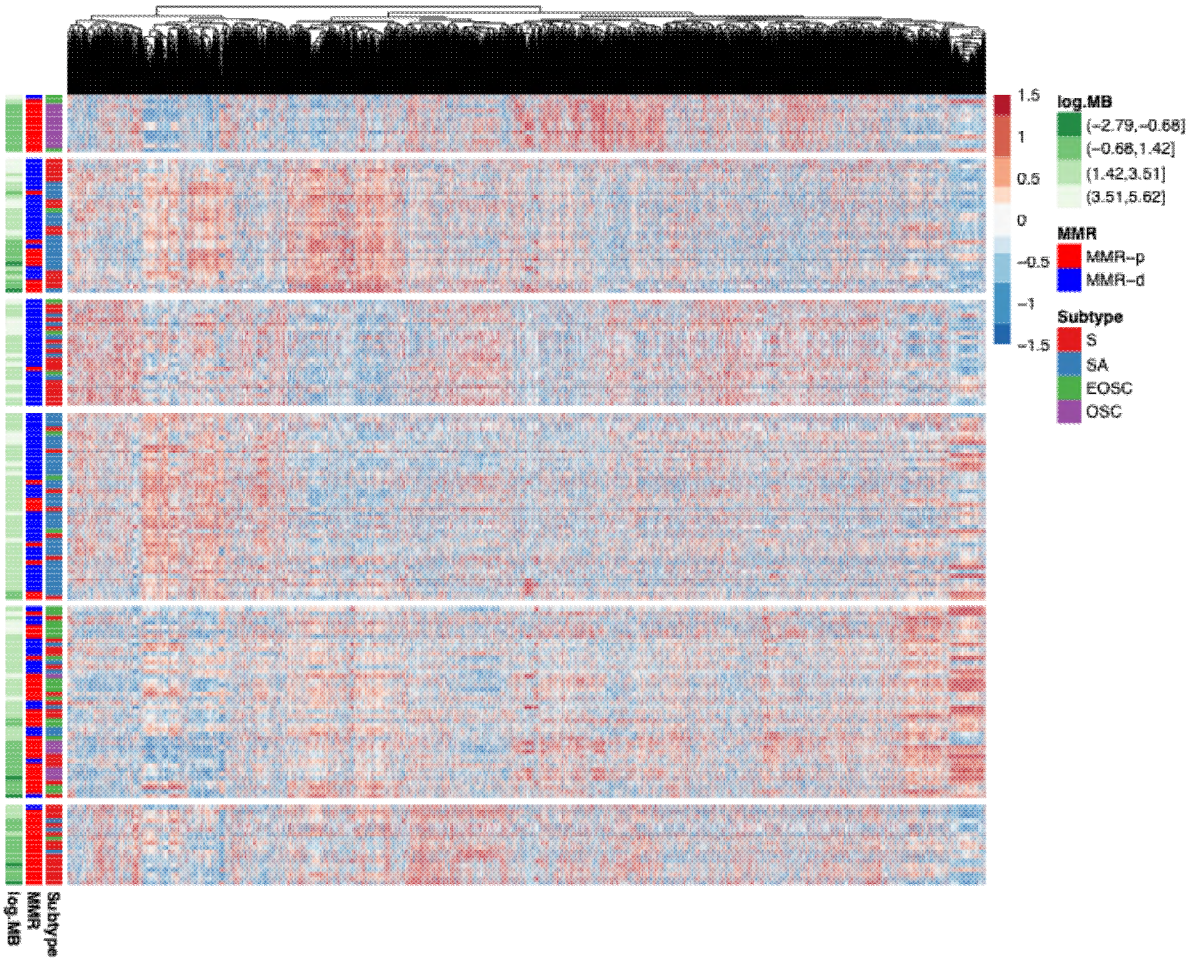

We provide these data and the associated matrix in the revised manuscript (in Github).

[4.2] Once these groups have been identified, do they correspond to the ST subtypes (SA vs. SC-E vs. SC-O)?

There is not a 1 to 1 correspondence between subtypes and transcriptomic clusters, but there is an association. The following table and the plot, representing the residuals of the Chi-square association test highlights the subtypes more enriched in each group. Exp-Cluster 1 is enriched in OSC (or SC-O) tumours, Exp-Cluster 3 in S tumours and Exp-Cluster 4 in SA tumours. Most of EOSC (or SC-E) tumours are in Exp-Cluster 5.

	1	2	3	4	5	6
EOSC	3	1	3	3	16	1
OSC	10	0	0	0	7	0
S	0	13	15	7	14	14
SA	0	16	6	32	6	3

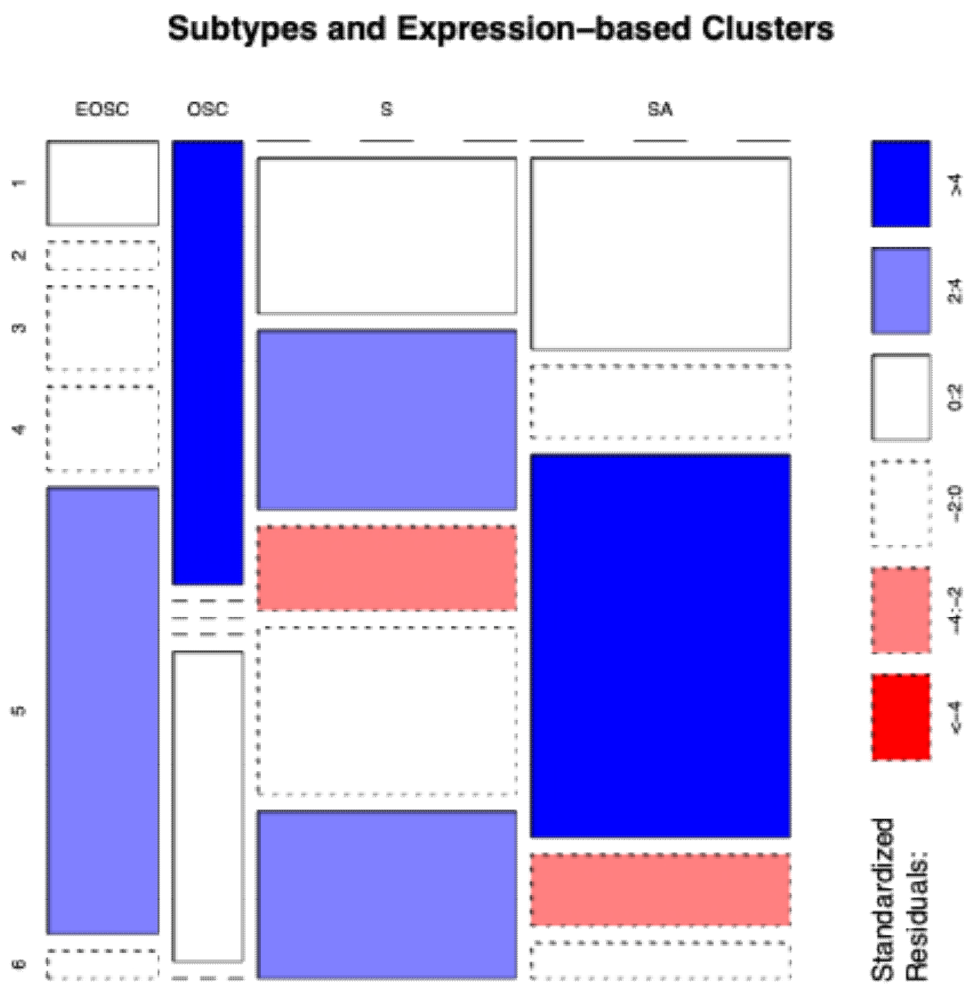

[4.3] Are they enriched for specific mutations or CNAs?

For each Exp-Cluster, we run a logistic regression comparing the probability of observing a mutation (from our subset of 84 genes) in the tumours of that Exp-Cluster with the probability of observing that mutation in the rest of the tumours. After multiple test FDR correction, we represented the significant associations in the following plot. Exp-Cluster 1 is characterised by mutations in *TP53, ZNF750* and *RB1* (although low mutational burden), Exp-Cluster 2 by *MSH6* mutations and lack of *TP53*, Exp-Cluster 3 and 4 by high mutational burden, and Exp-Cluster 6 by lower mutational rates (see figure attached).

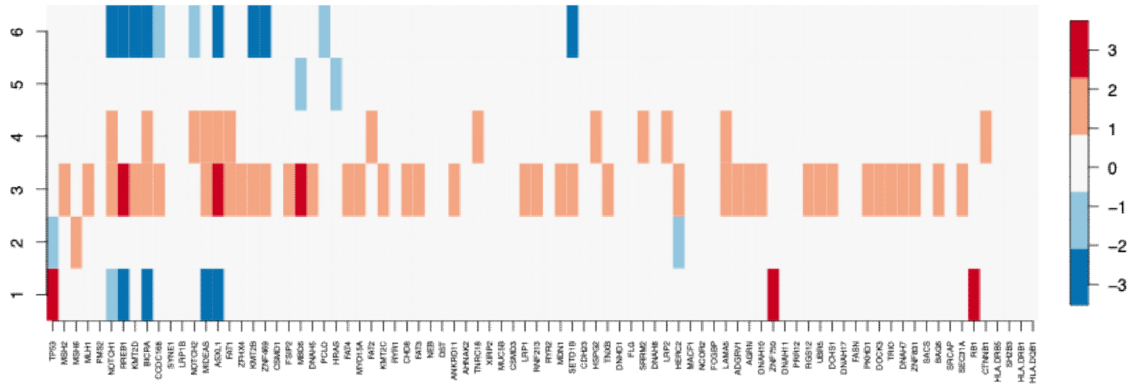

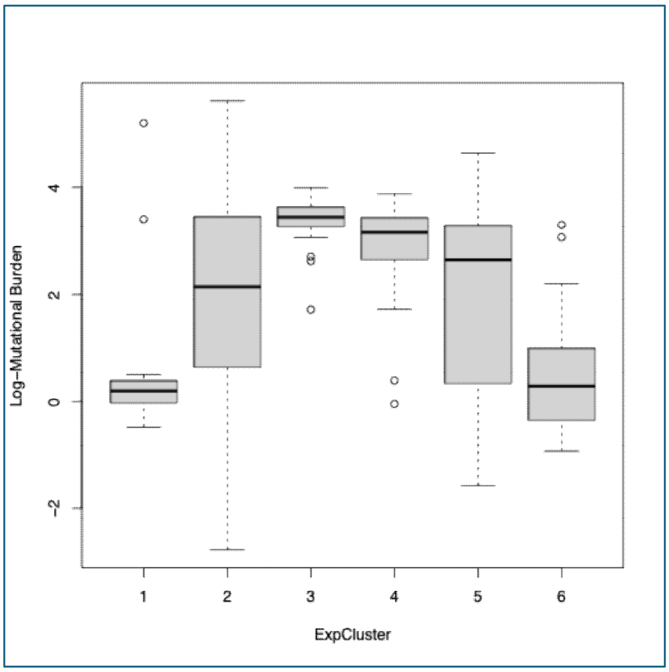

With respect to copy number aberrations, there are also important differences amongst groups, with group 1 having a very specific copy number pattern, and other groups with very few aberrations. The next plot shows the differences.

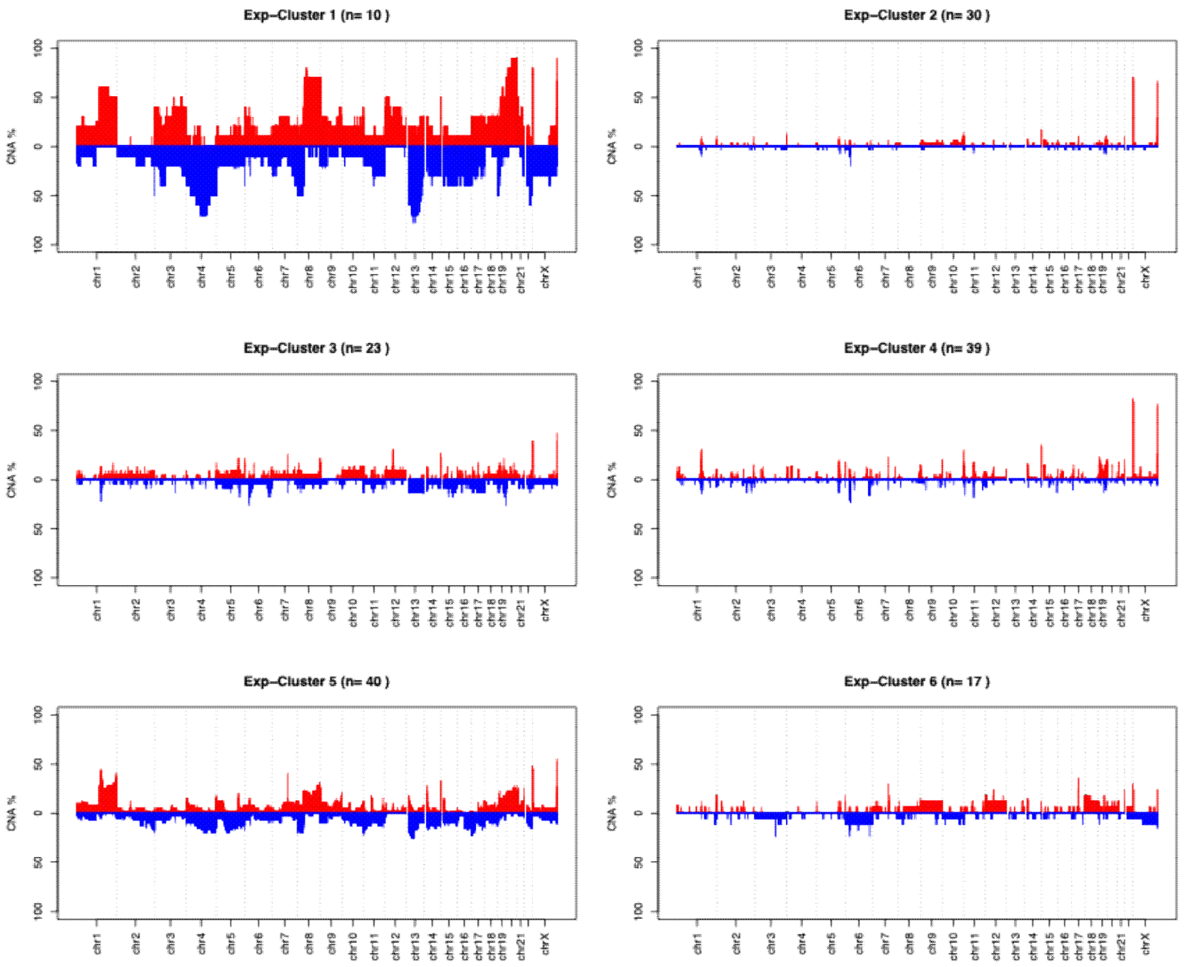

[4.4] Which immune and other non -tumor cell types are present in each of these bulk tumors? How does this correlate with tumor purity?

Using EPIC for cell deconvolution, we estimated proportion of different cell types. As the next plot shows, cluster 2 has a high proportion of endothelial cells, cluster 5 (with up-regulation in interferon pathways, see next section) has a high proportion of B cells, CD4+ T cells and macrophages. Cluster 6 has a slightly higher proportion of CD8+ T cells.

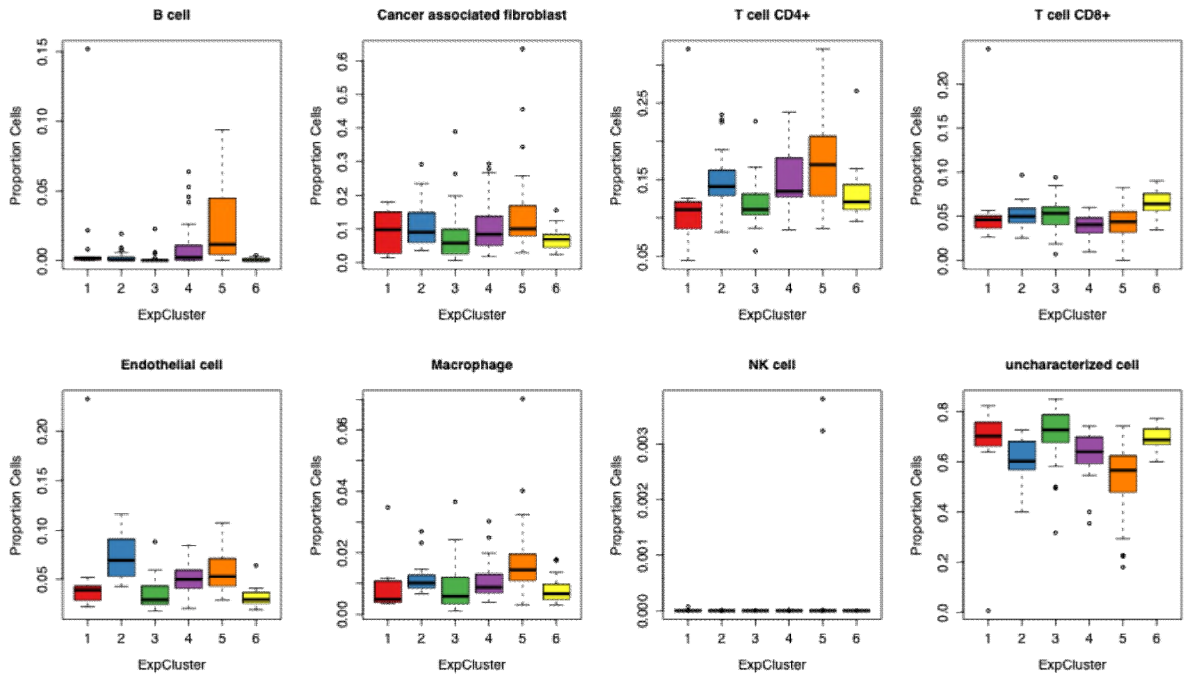

[4.5] Which pathways or genes seems differentially expressed between these groups? Could this be confounded by tumor purity or non-tumor cells or is there evidence that they could represent genuine differences between the cancerous or neoplastic cells?

We performed Gene set enrichment analysis on the full expression matrix on the 50 Hallmark Gene sets, and after FDR correction we identified the following enriched pathways in each Exp-Cluster (red means positive normalised enrichment score (NES), and blue negative NES)

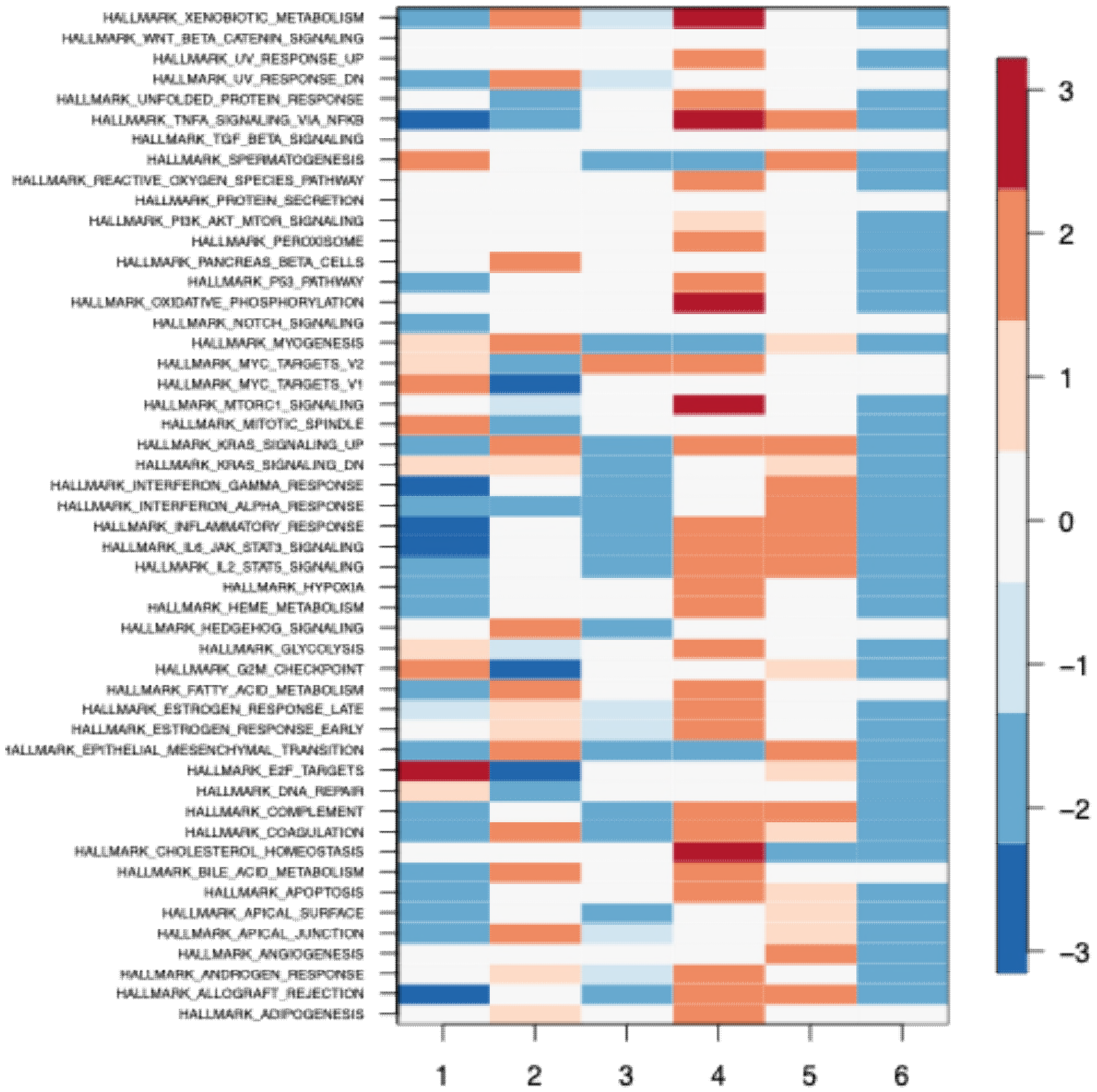

With respect to tumour cellularity, we had purity estimates obtained from the ASCAT algorithm on the DNA sample for 159 samples. We must note that this method is far from perfect and it produces several 100% purity values in 35 of the cases. However, it should give a rough idea of the association with our clusters. As the plot below shows, in the case of the purely transcriptomic clusters, only Exp-Cluster 5 is composed of lower purity tumours (although with a non-significant adjusted t-test from a linear model, p-value=0.055). In the case of the MOFA IntClusts, we also see a group with lower tumour purity, IntClust 4, but again a non statistically difference in the average purity. Overall, purity does not seem to impact any of the clusters.

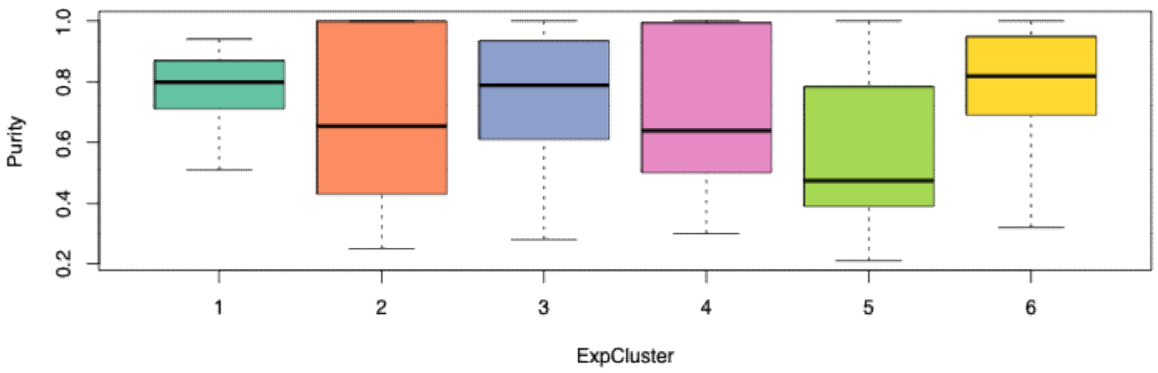

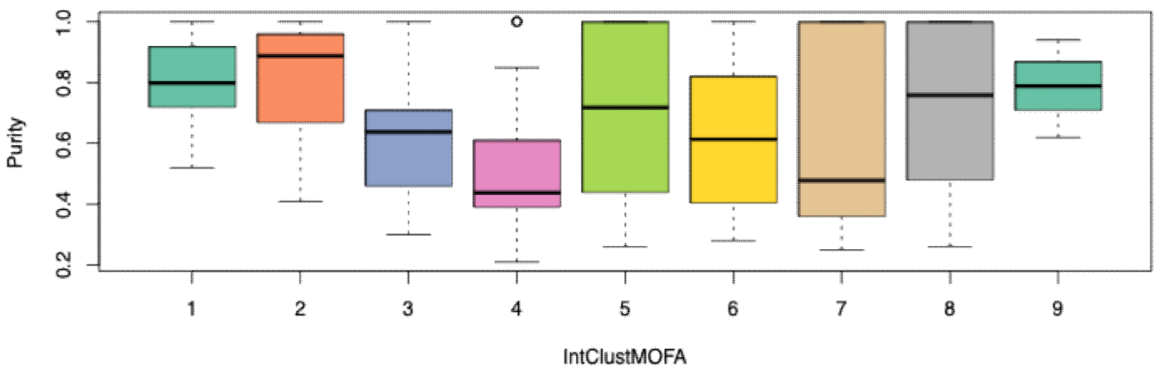

In summary:
Exp-Cluster 1 is characterised by mainly SC-O tumours and a few SC-E, low mutational burden, but selected mutations in *TP53, RB1* and *ZNF750*. Proficient MMR and pathway activation of E2F targets, G2M checkpoint and MYC targets.
Exp-Cluster 2 is characterised by SM and SA subtypes, 70% of the tumours are MMR deficient and 33% and have several overexpressed pathways, such as Bile Acid Metabolism, myogenesis and UV response down.
Exp-Cluster 3 is enriched in SM tumours, with high mutational burden and almost all of them (96%) MMR deficient (several with *MLH1* mutations). They have up-regulation of the MYC pathway and down-regulation of many other pathways.
Exp-Cluster 4 is mostly formed by SA tumours, carrying a large number of mutations (81% of them MMR deficient). Up-regulation of many pathways, such as Estrogen response, inflammatory response, MTORC1 signalling and P53.
Exp-Cluster 5 is mostly characterised by SC-E tumours and activation in the interferon gamma and alpha response pathways. It has a high proportion of B cells, CD4+ T cells and macrophages
Exp-Cluster 6 is mainly formed by SM tumours, with low mutational burden and MMR proficient (94%), with down-regulation of most pathways.

Comparing the purely transcriptomic clusters and the integrative clusters, we see some differences, but the integrative clustering is able to identify interesting tumours with unique combination of mutational and expression profiles, such as IntClust 4.

	1	2	3	4	5	6	7	8	9
1	1	0	0	3	0	0	0	0	9
2	6	0	1	1	1	1	15	5	0
3	4	6	0	0	1	13	0	0	0
4	0	0	11	0	22	4	4	1	0
5	1	6	1	19	10	2	0	3	1
6	1	3	0	0	0	0	0	14	0

Clinical data:

[5] Supplementary Table 1 is a somewhat ambiguous and includes a mix of patient-related and sample-related data with several issues. I recommend that the authors provide two separate tables: one for the patient-related data and one for the sample-related data, each with appropriate IDs (patient and sample).

We thank Reviewer2 for sharing his concern with us about Supplementary Table 1. We decided to keep the original and add two supplementary sheets to it following your suggestions, so the reader can select the one that is more appropriate (please see **Supplementary Table 1**).

[5.1] Some fields lack sufficient description in the legend, such as "Age," which does not specify whether it refers to the age at diagnosis or at sample procurement. Similarly, “Recurrence”, “TimeAfterInitialDiagnosis_Rec(months)”, “Metastasis” and “TimeAfterInitialDiagnosis_Met(months)” also needs some explanation.

We thank Reviewer2 for pointing this out. We corrected this (please see **Supplementary Table 1**).

[5.2] Column AE has no name. It contains “Father, Brother” for sample “PD42477a”, but “FamilyCancerLynch” says “no” for this sample.

We thank Reviewer2 for pointing out these mistakes, which we fixed (please see **Supplementary Table 1**).

[5.3] Some field names differ between the table and the legend. For example, "MMRstatus" is mentioned in the legend, but "MMR.d.MMR.p_simplified" appears in the table. Same for some IHC fields.

We thank Reviewer2 for highlighting these mistakes, which we fixed.

[5.4] The MMR status is not written correctly for PD42171a (MMR-p instead of pMMR).

We thank Reviewer2 for pointing out this mistake, which we have fixed.

[5.5] For "MLH1Hypermathylation," a mix of blank and "not tested" is used. Please use a unique value (either blank or "not tested") for all cases to maintain consistency.

We thank Reviewer2 and have amended the table as suggested.

[5.6] Typos: “Hypermathylation”, “Supplemntary”

We thank Reviewer2 for pointing out these mistakes, which we have fixed.

[5.7] There are a number of issues in this table. It would be ideal to ensure that all fields used in the analyses are clearly documented and are error-free. I would recommend fields that cannot be used in any reasonable analyses, because of incomplete or ambiguous data, should be dropped.

We thank Reviewer2 for sharing their concerns with us. We have replaced whitespace with NA. Since we have collected all the data variables included, we feel that we are future proofing the data set by including it.

Germline alleles in sebaceous tumour patients:

[6.1] This section could be further developed. Associated Figure 6 contains 27 genes, but only a handful of these genes are mentioned in the text. I would recommend improving figure (e.g., removing genes not discussed and adding other plots showing how mutations correlate with phenotypes) or moving it to a Supplementary Figure.

We thank Reviewer2 for this recommendation. We have made a new **Figure 6** (which is now Figure 7) including only samples mutated for MMR and/or *MUTYH* genes, as well as clinical details (please see **Figure R20/Figure 7**). We would like to highlight to the reviewers an important finding. Visual inspection of all germline variants suggested aberrant calls at the *POT1* locus which we have removed from the revised figure (below). Indeed, variants found in *POT1* were removed as after manual inspection they appeared to be artefactual (please see ‘Germline variant calling’ in the Methods).

Figure 7

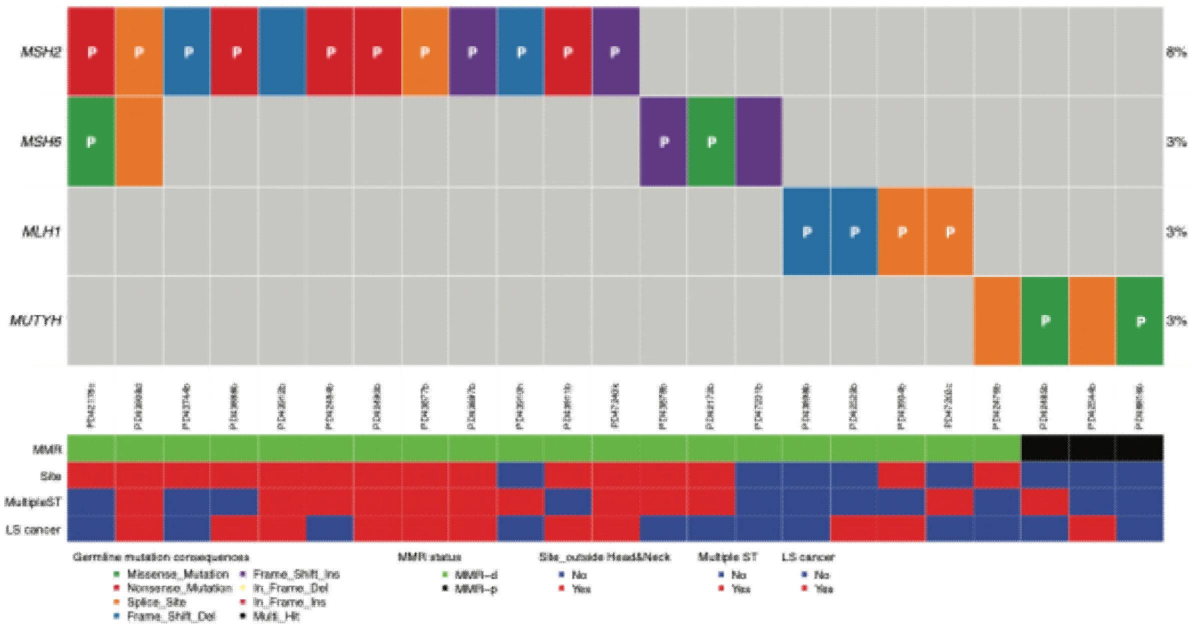

Figure R20/Figure 7 - Germline variant landscape of sebaceous tumour patients. Oncoplot showing the variants present in the normal samples. Constitutional (germline) variants or those with disruptive consequences (i.e. consequences classified as frameshift, nonsense or splice site by VEP, or variants defined as 'pathogenic'/'likely-pathogenic' by ClinVar) are shown. Only genes included in the NHS cancer national genomic list for testing and mutated in at least two 26 patients are shown. Abbreviations: P, variant defined as 'pathogenic'/'likely-pathogenic' by ClinVar; S, variant identified as significantly depleted by SGE.

[6.2] VAF and other important information is missing from Supp. Table 12. While the method section states that germline variants were identified with GATK HaplotypeCaller and GenotypeGVCFs, Supp. Table 12 includes fields such as "Tumor_Seq_Allele2" which are typically associated with somatic variant calling. Could the authors clarify this?

We thank Reviewer2 for reporting this issue. We have added to the Supp Table 12 the variant allele frequency for the germline analysis (VAF_norm), as well as modified the name of the columns (Tumor_Seq_Allele2 became ALT and Reference_Allele became REF).

Copy number landscape of sebaceous tumours:

[7.1] This section could be better developed. It would be ideal if both Figures 4 and 5 could be simplified to include only the key panels that are clearly highlighted to emphasize points made in the main text.

We thank Reviewer2 for this comment. We have added titles to make the figures more comprehensive for the reader (please see **Figure R21/Figure 5).**

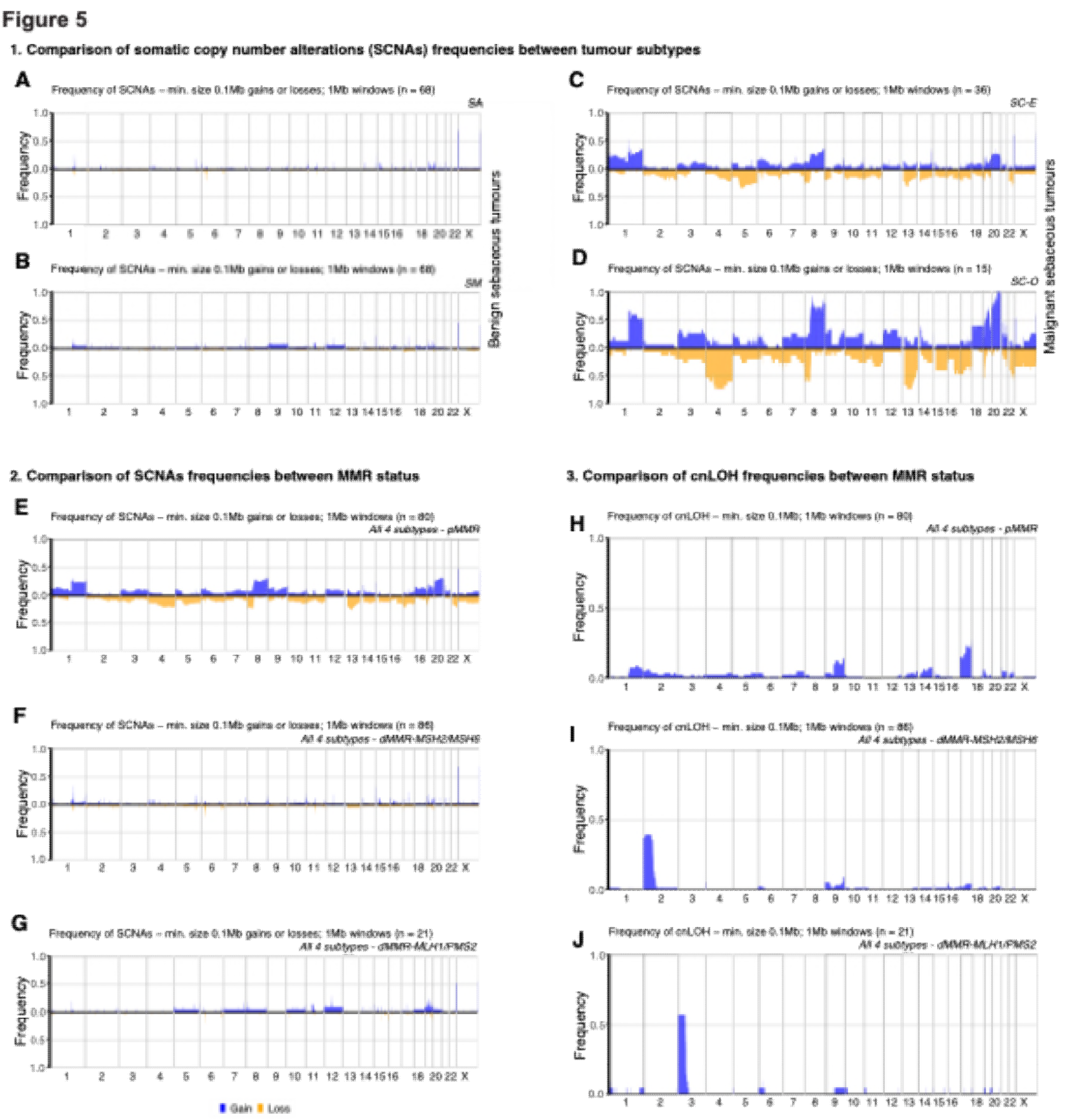

Figure R21/Figure 5 - Somatic copy number alterations in sebaceous tumours. Penetrance plots of somatic copy number alterations in (A) sebaceous adenomas (SA), (B) sebaceoma (SM), (C) extra-ocular sebaceous carcinoma (SC-E), (D) ocular sebaceous carcinoma (SC-O), (E) mismatch repair proficient (pMMR) sebaceous tumours (STs), (F), mismatch repair deficient (dMMR) STs showing loss of MSH2/MSH6, and (G) dMMR STs showing loss of MLH1/PMS2. Frequency plots of copy neutral loss of heterozygosity (cnLOH) in (H) pMMR STs, (I) dMMR STs showing loss of MSH2/MSH6, and (J) dMMR STs showing loss of MLH1/PMS2. Abbreviations: dMMR-MSH2/MSH6, deficient in MMR with MSH2/MSH6 loss of expression by immunohistochemistry; dMMR-MLH1/PMS2, deficient in MMR with MLH1/PMS2 loss of expression by immunohistochemistry; Mb, megabase; min, minimum.

Figure 6

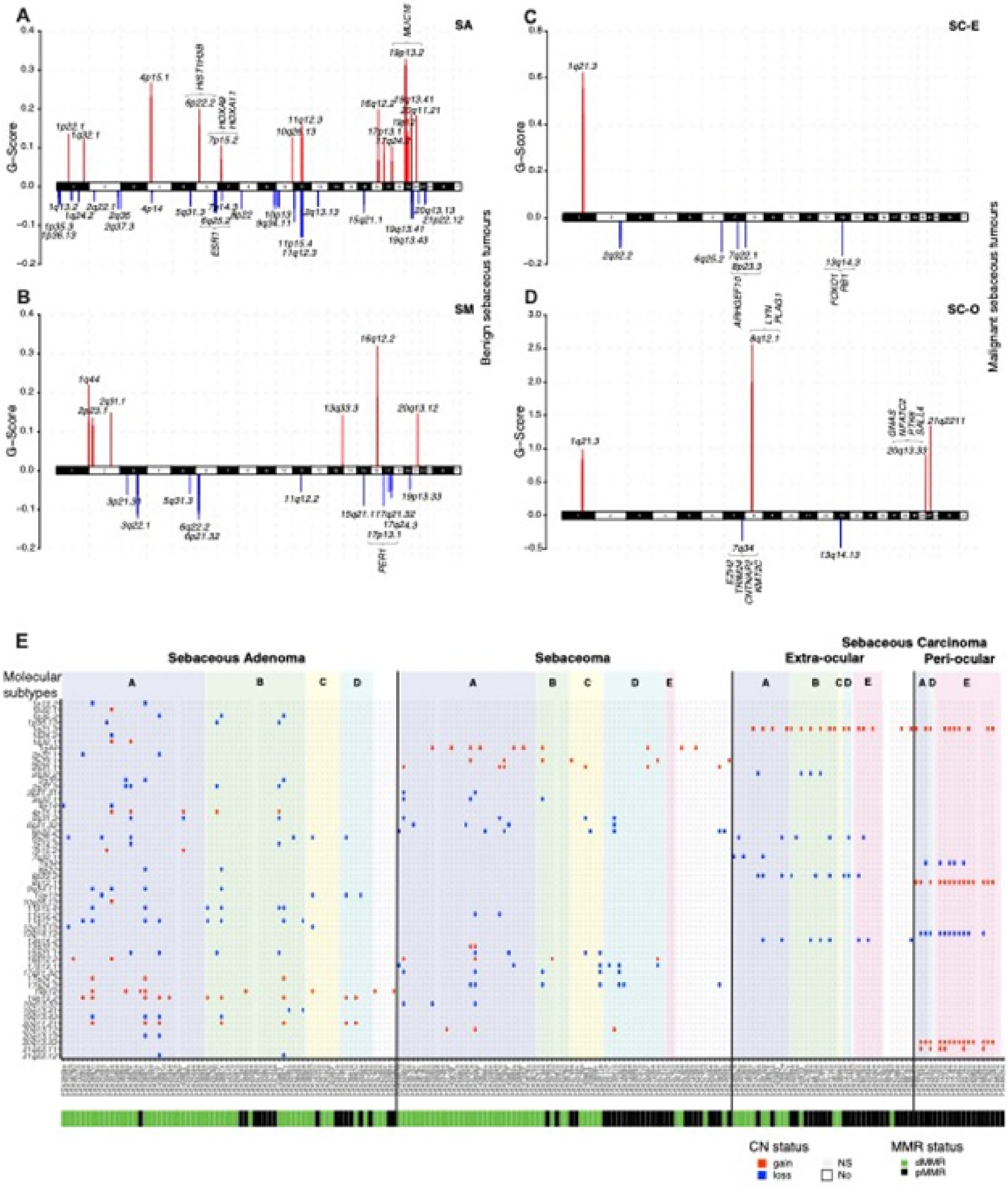

Figure R1/Figure 6 - Significant focal somatic copy number alterations in sebaceous tumours. Representation of the GISTIC2.0-identified by ST subtype significant focal peak regions (≤ half a chromosome arm) in (A) SA, (B) SM, (C) SC-E) and (D) SC-O. Chromosomal location of the peaks are indicated by the cytoband, with amplifications (gains) shown in red and deletions (losses) shown in blue; cytobands in grey are peaks defined as non-significant. The presence of known tumour suppressor genes or oncogenes within these peak regions are

labelled. For the G-score, positive values refer to gains, while negative values are related to deletions. (E) Oncoplot showing the individual tumour samples with the amplification and deletion regions shown in A-D. Samples with no significant gains or losses are indicated by ‘NS’. Five samples were not included in the GISTIC2.0 analysis (see **Methods**), so no data is shown (indicated by ‘No’). Note: *focal peaks were considered as significant when reaching the 4 criteria: (i) GISTIC2 residual q-value < 0.1, (ii) peak size >= 100,000bp, (iii) concordance with ASCAT CN calls ≥ 0.75, (iv) portion of peak not overlapping with Genome In A Bottle ‘difficult regions’ by >40% (**Supplementary Table 9**); **approximate cytobands are shown with related genomic coordinates available in **Supplementary Table 9**.

Neoantigen and ICI response prediction:

[8] The last section of the paper (lines 413-482) reports results based on a number of software tools (CODEFACS, TIMER2.0, EPIC, LIRICS, CIBERSORT, pVACseq, and others) and several external datasets that I evaluate as highly speculative. There are insufficient details provided to properly understand the methodology and the results, which are supported by a single, not particularly informative, main figure. At this stage, I would recommend removing this section to ensure clarity and rigor of the whole exome and transcriptome analysis of the paper, or significantly revise this section with more clear details on how the analysis was performed. However, I should note I anticipate the results will likely still be speculative and omission of the ligand-receptor analysis should be considered if validation by IHC/IF staining studies are not being included.

We thank Reviewer2 for sharing their concerns about the neoantigen and ligand-receptor analyses. We agree these analyses are purely speculative and require follow-up with further functional analysis. We have largely removed these analyses or moved the remaining elements to the supplementary information with a note that they require validation.

Other:

[9.1] There is some inconsistent naming and abbreviation usage for the tumor subtypes across the main text, figures, and their legends. For instance, "SM" is used for sebaceoma in Figure 1 and Table S1, while only "S" is used in Figure 3 and S5. Similarly, "PeriOcular," "Peri-ocular," and "ocular" are all used to refer to ocular sebaceous carcinoma. Please standardize the terminology and abbreviations for each disease subtype.

We thank Reviewer2 for pointing out these mistakes, which we have fixed.

[9.2] According to ST1, sample PD43886c and PD48829c have been used in DNA analysis (DNA_Analysis == Yes). However, I couldn’t find any mutations associated with these samples in ST4.

We thank Reviewer2 for pointing out this discordance. We have fixed the table to make it clearer.

[9.3] SF1A has no legend describing the meaning of each color and does not seem appropriate to clearly support the claims.

We thank Reviewer2 for pointing out and we have corrected the figure legend accordingly.

[9.4] The cohort size is relatively large, considering the rarity of these tumors. However the SC sub-group is quite small, particularly the SC-O subgroup which may limit the power of the analyses. More discussion of this (and other limitations) would be appreciated in a short, dedicated section.

We agree that the SC-O collection is small but it remains the biggest collection of this rare tumour type ever studied. In future, additional SC-O may be sequenced and combined with our dataset. We inform the reader about the challenges posed by the SC-O sample number in the results section ("**RESULTS - Sample ascertainment and clinico-histopathological features**" please see revised Manuscript).

[9.5] Suggestion: Is it necessary to note that no recurrence or metastasis arose in SA or SM (Line.149) as these are benign tumors that do not usually (or at all?) metastasize. If they do, this should be noted in the text.

We thank Reviewer2 and removed the sentence.

[9.6] Suggestion: It would be ideal to list the 6 countries that these tumors come from (line 81) and mention in discussion any potential biases from tumor selection based on ethnicity or geographical location – for example, authors mention that SC-O is more common in people of Asian descent (40-60% of eyelid tumors). Have the authors investigated the ethnic makeup of their cohort? Is it representative of the global or regional prevalence/distribution of these tumor subtypes? If not, how might this affect the results of their study?

We thank Reviewer2 for making this point and have specified the different countries where the tumours came from. An ancestry analysis was performed to explore the genetic origin of the patients. We projected their genotypes against the 1000 genomes dataset (**Supplementary Figure 1**) revealing largely European and Asian descent as expected.

Reviewer #3 (Remarks to the Author): expertise in neoantigen prediction

Authors investigated the genetics and mutational signatures of sebaceous tumors and reached into the conclusion that there are multiple subtypes present. The study also has an interesting observation that some of these tumors also have lynch signatures. The lynch signatures should be investigated further using current non tumor and historical patient samples to reach a definitive conclusion that these mutations were not acquired later in development.

We thank Reviewer3 for their comment however, it remains unclear for us what they want us to do. We have performed a germline analysis where we identified known disruptive variants across established genes from the Cancer national germline genomic testing panel performed by the NHS/UK. We observed that several patients carried an inherited mutations in MMR genes and *MUTYH* as previously described making the aetiology of the Lynch signatures in these patients most likely related to these germline alleles.

Figure 7

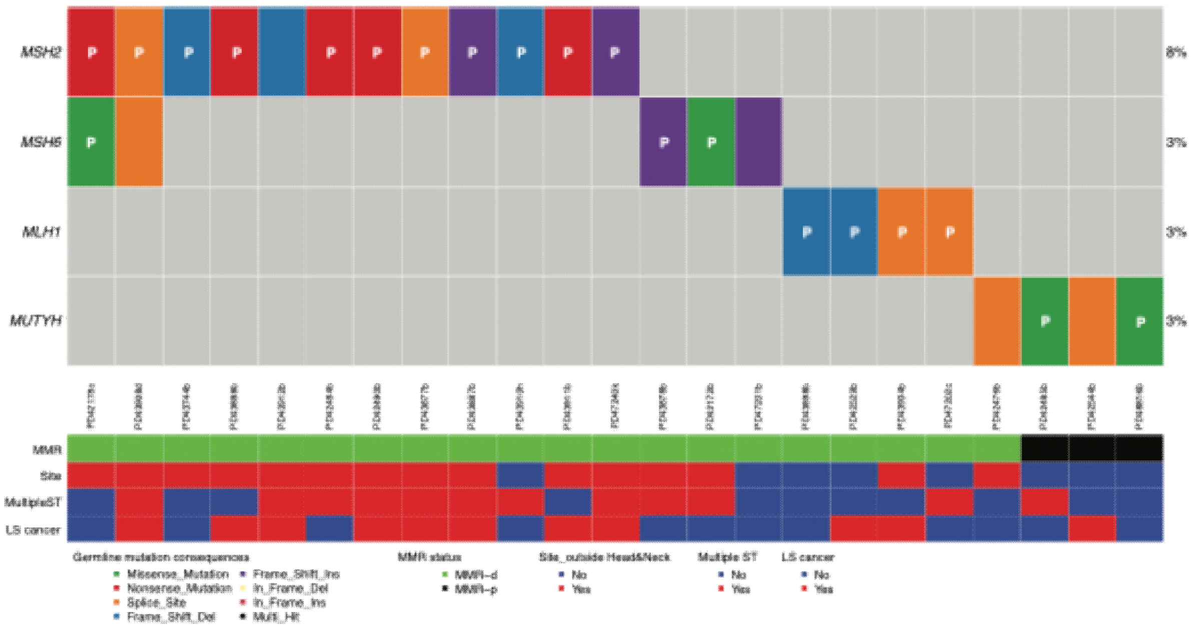

Figure R20/Figure 7 - Germline variant landscape of sebaceous tumour patients. Oncoplot showing the variants present in the normal samples. Constitutional (germline) variants or those with disruptive consequences (i.e. consequences classified as frameshift, nonsense or splice site by VEP, or variants defined as 'pathogenic'/'likely-pathogenic' by ClinVar) are shown. Only genes included in the NHS cancer national genomic list for testing and mutated in at least two patients are shown. Abbreviations: P, variant defined as 'pathogenic'/'likely-pathogenic' by ClinVar.

Utilizing some published material to support Sebaceous tumor prevalence in Lynch syndrome would also be informative.

Sebaceous tumours develop in less than 5% of patients with Lynch syndrome (LS) or hereditary non-polyposis colorectal cancer (HNPCC) syndrome, which has also been referred

to as Muir-Torre syndrome (MTS) (Marcoval J *et al.* 2019, Schwartz RA *et al*. 1995). The incidence of LS/MTS in patients with sebaceous neoplasms ranges between 14 and 50% (Marcoval J *et al.* 2019, Ponti G *et al*. 2025, Roberts ME *et al*. 2013, Roberts ME *et al*. 2014, Lee BA et al. 2012, Everett JN *et al.* 2014) suggesting patient ascertainment is an important factor in reporting the frequency of the disease.

Authors utilize available pipelines to perform a lot of their computational analysis, it would be helpful if same pipelines are used on some examples of published historical skin tumors such as Melanoma, squamous carcinoma, UV mediated Merkel cell carcinoma for comparison in these figures such that the reader could make comparisons of mutational burden etc. these results may be skewed by the pipelines used.

We thank Reviewer3 for they comment. We have extracted data from MSK-impact via the cBioPortal and compare the mutation frequency to our ST data as shown in the plot below.

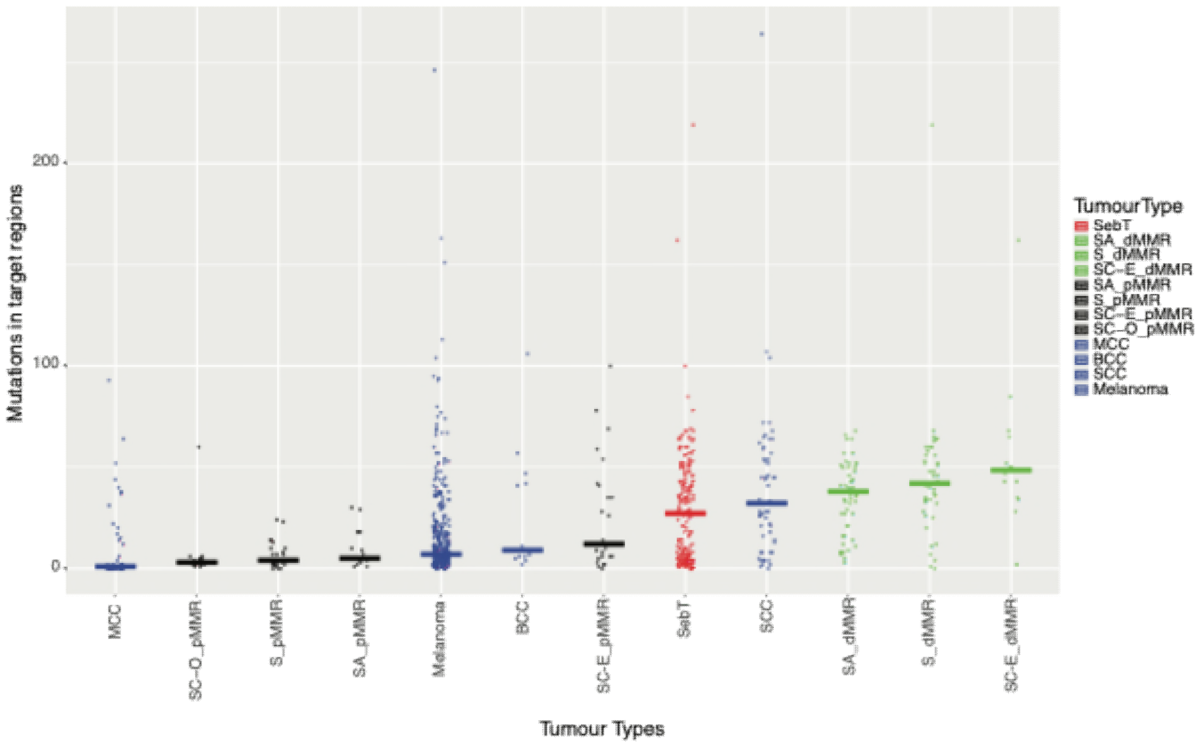

Figure R22 - Comparison of mutation count in target regions from four MSK-Impact skin tumour types (Merkel cell [MCC], melanoma, BCC and SCC) to the sebaceous tumours (STs) in this study. The ST cohort are shown all together (red dots), or separated by subtype and mismatch repair (MMR) status (dMMR as green dotsfont and pMMR as black dots). Abbreviations: BCC, basal cell carcinoma; MCC, Merkel cell carcinoma; SCC, squamous cell carcinoma.

Comparing the same panel of mutated genes, we can observe dMMR STs tumours (benign and malignant STs; highlighted in green) have a higher mutation burden compared to other malignant skin tumour subtypes.

Authors make some suggestions about the immunogenicity of RCC tumors, this is still currently a puzzle since we don't know what makes RCC tumors highly immunogenic. First the Mutational burden for sure does not explain RCC immunogenicity and secondly the CD8 T cell infiltration in RCC tumors is actually deleterious. So the discussion and comparison to these tumors would need to be carefully worded and considered in the text. Authors make conclusions about the immunogenicity of the tumors. This part requires additional work. First the authors again utilize some pipeline to do their analysis, this would be valid if there are some controls presented for comparison. So the Neoantigen score for Melanomas, squamous carcinomas and UV induced merkel cell carcinoma utilizing published data should be presented as a control comparison.

We thank Reviewer3 for their comment about the neoantigen and deconvolution analyses. We have deleted most of these analyses and/or moved them to the supplementary material. We have removed the direct comparisons across tumour types. We feel we present a meaningful analysis of these data.

Additionally the authors present histology data in figure 1. As a supplement an immune score calculation utilizing CD4/CD8 infiltration should be presented for each tumor since the authors have access to FFPE blocks. This would make the interpretation of the all neoantigen loads the authors are presenting and would have to validate some of the observations

We would not know how to interpret the data based on this as, for example, Brisk and non-Brisk infiltration is more important in melanoma. Again, we moved all the Figures related to these analyses to supplementary material.

We thank Reviewer 3 for their very thoughtful comments.

RESPONSE TO REVIEWERS' COMMENTS

Reviewer #1 (Remarks to the Author):

The authors did a thorough job in addressing the reviewers' concerns and the manuscript has been significantly improved. My comments have been adequately addressed.

> We thank Reviewer #1 for reviewing this paper and their insights into our study.

Reviewer #2/3 (Remarks to the Author):

The authors' have made considerable efforts to revise their manuscript and respond to suggested revisions and comments. However, two key issues have not been appropriately addressed and require further attention.

> We thank Reviewer #2 for reviewing our revised manuscript and apologise if we misunderstood some of their points.

1. Driver Gene Analysis & False Positives

In the initial review, concerns were raised about the likely high proportion of false positives due to the authors' reliance on a single tool (dNdScv) without proper filtering or orthogonal validation. Specifically, SPANXB1 was highlighted as an example of a likely false positive due to the low VAF of its mutations. In response, the authors argue that, based on the interquartile range method, SPANXB1's VAF is not an outlier. Unfortunately, the authors response misses the point of the major concern raised—that further examination of the significantly mutated gene list is needed to identify and remove/flag likely false positives before publication. For example, in re-analyzing the data provided by the authors, 85% of SPANXB1 mutations have a VAF < 0.25, which is directly at odds with their own Figure R8, where they claim LOH is observed in nearly 70% of cases. Additionally, it is important to note that SPANXB1 is located on the X chromosome, and most of its reported mutations occur in males—where there is no heterozygosity to lose. This raises concerns regarding the LOH claim in Figure R8 and the robustness of the significant mutated gene list.

> We thank Reviewer #2 for noting that *SPANXB1* is the only driver genes located on Chr X which could cause misinterpretation in the copy number analysis and more specifically in cnLOH. In agreement with Reviewer #2, we then decided to remove *SPANXB1* from Figure R8 which is included in Supplementary Figure 4.

Supplementary Figure 4

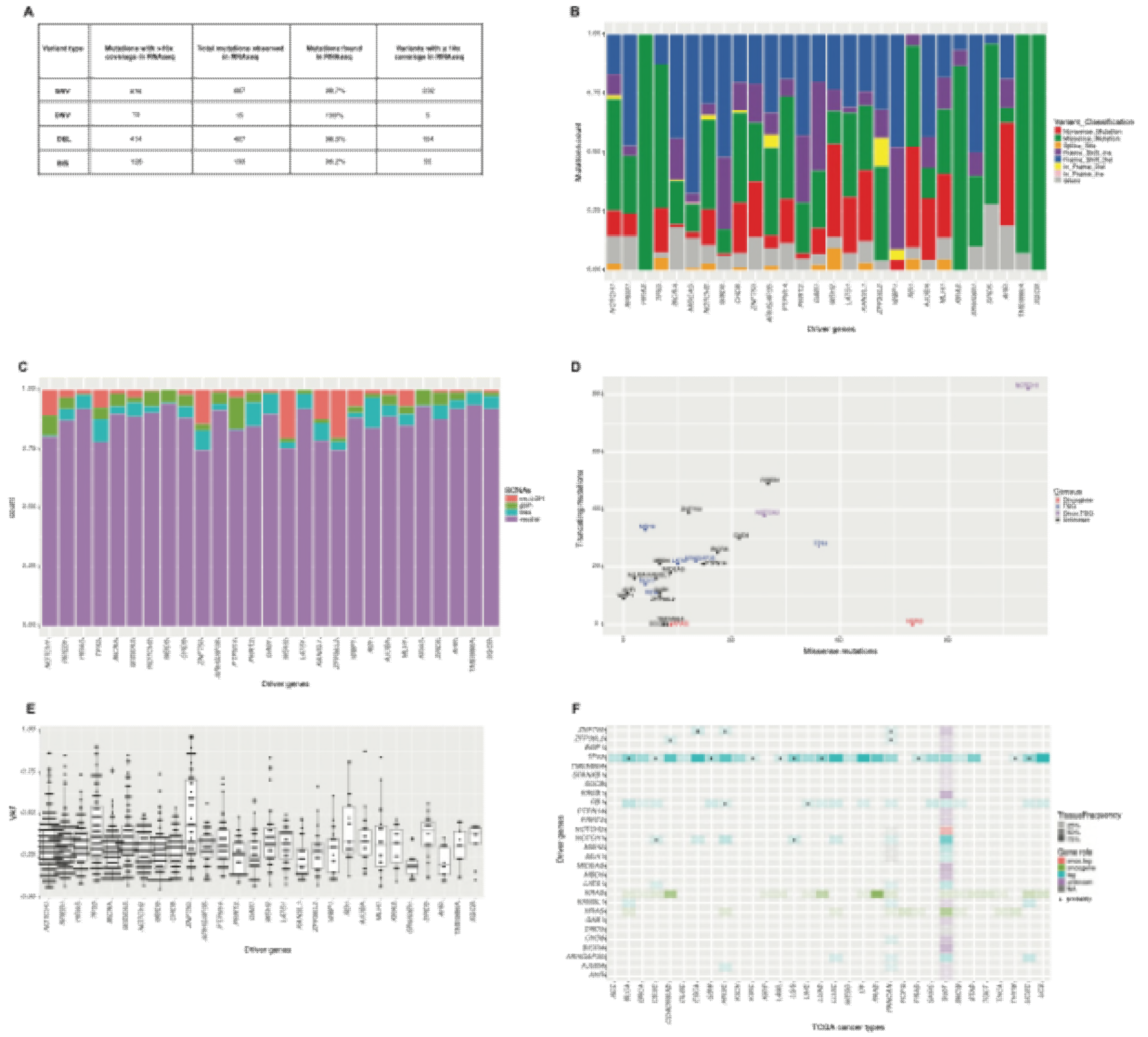

Supplementary Figure 4 – Driver genes and associated changes. (A) Table showing the proportion of somatic mutations also present in RNAseq. (B) Barplot showing the proportion of the different variant types across driver genes. (C) Barplot showing the proportion of the somatic copy number alterations (SCNAs) across autosomal driver genes. (D) Scatter plot showing missense versus truncating mutation profiles across the driver genes. (E) Barplot showing the distribution of the variant allele frequency (VAF) across the driver genes. Each dot is a tumour. Of note, *SPANXB1* is located on the X chromosome which is likely reflective of the low VAF we observe. (E) Tileplot showing the alteration of the driver genes across sebaceous tumours and pan-cancer analyses. Genes are from Bailey MH et al., 17. The gene role is taken as defined by Bailey MH et al., in 17 and COSMIC Cancer Gene Census database for TCGA cancers and sebaceous tumours, respectively. Abbreviations: NA, not defined; onco, oncogene; onco.tsg, oncogene and tumour suppressor genes; tsg, tumour suppressor gene.

Importantly, the suggested additional analyses —VAF distribution, mutation signatures, gene-level RNA-Seq validation (e.g. RNA-VAF vs DNA-VAF), and co-occurrence of LOH with loss-of-function mutations —were essential recommendations for validation/filtering steps to refine the driver mutation gene list before publication (i.e. identify and remove/flag the likely false positives). These analyses should be included in the paper to demonstrate that the final significantly mutated gene list is robust and

biologically meaningful.

> We thank Reviewer #2/3 for this suggestion and we have added Figure R5 and the table comparing the number of mutations found in the DNA and RNA data, which reinforce the fact that the data are of high quality, to Supplementary Figure 4.

Supplementary Figure 4

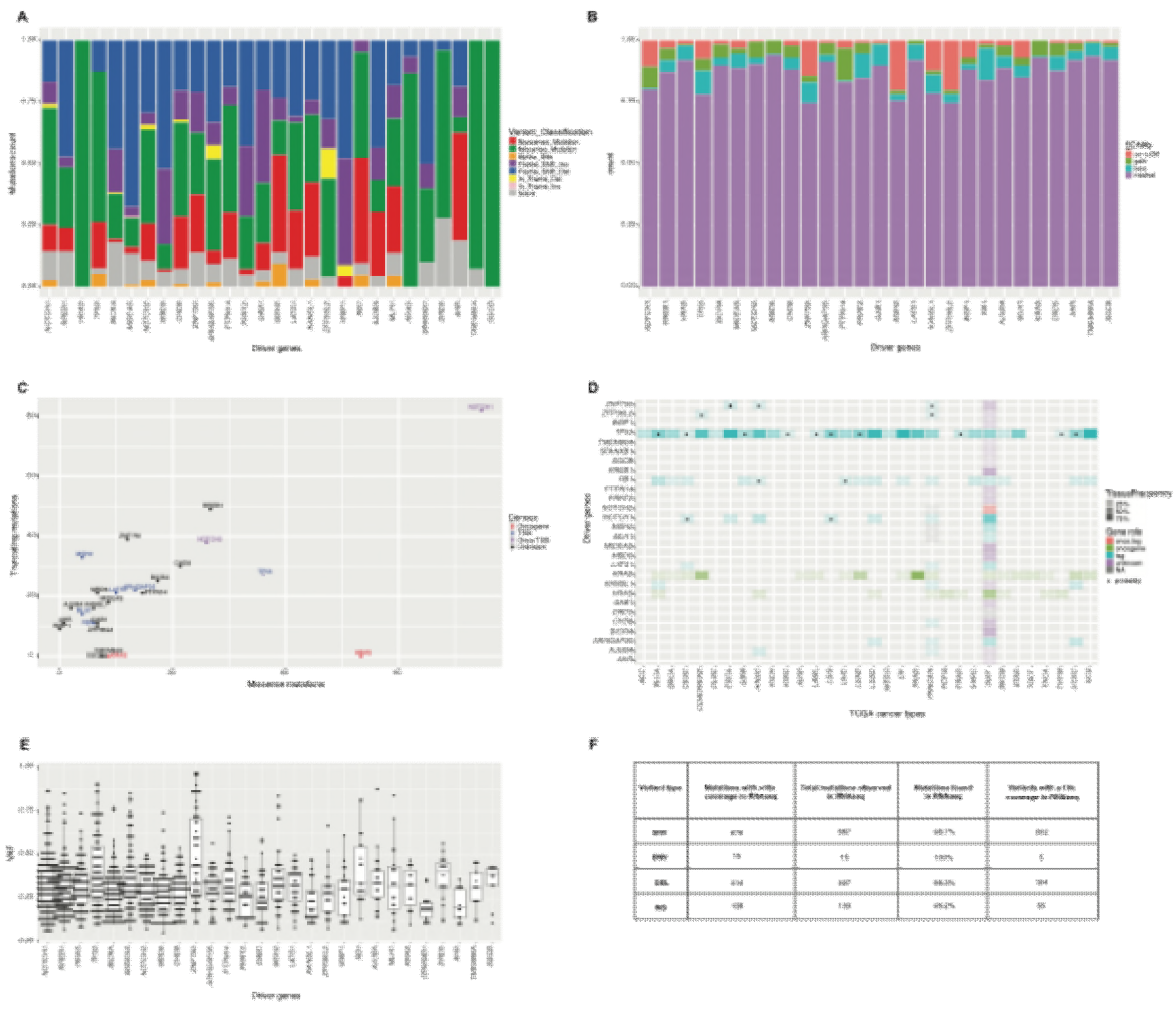

Supplementary Figure 4 – Driver genes and associated changes. (A) Table showing the proportion of somatic mutations also present in RNAseq. (B) Barplot showing the proportion of the different variant types across driver genes. (C) Barplot showing the proportion of the somatic copy number alterations (SCNAs) across autosomal driver genes. (D) Scatter plot showing missense versus truncating mutation profiles across the driver genes. (E) Barplot showing the distribution of the variant allele frequency (VAF) across the driver genes. Each dot is a tumour. Of note, *SPANXB1* is located on the X chromosome which is likely reflective of the low VAF we observe. (E) Tileplot showing the alteration of the driver genes across sebaceous tumours and pan-cancer analyses. Genes are from Bailey MH et al., 17. The gene role is taken as defined by Bailey MH et al., in 17 and COSMIC Cancer Gene Census database for TCGA cancers and sebaceous tumours, respectively. Abbreviations: NA, not defined; onco, oncogene; onco.tsg, oncogene and tumour suppressor genes; tsg, tumour suppressor gene.

One last additional point, when showing the distribution of mutation consequences by gene (Figure R9), synonymous mutations must be included, as they are essential for interpreting selection patterns (e.g. a high proportion of synonymous mutations

suggests weak or no selection).

> We thank Reviewer #2/3 for this suggestion and we have modified Figure R9 by including synonymous (silent) variants which we include into Supplementary Figure 4. As shown, synonymous mutations are not major events in these driver genes.

Supplementary Figure 4

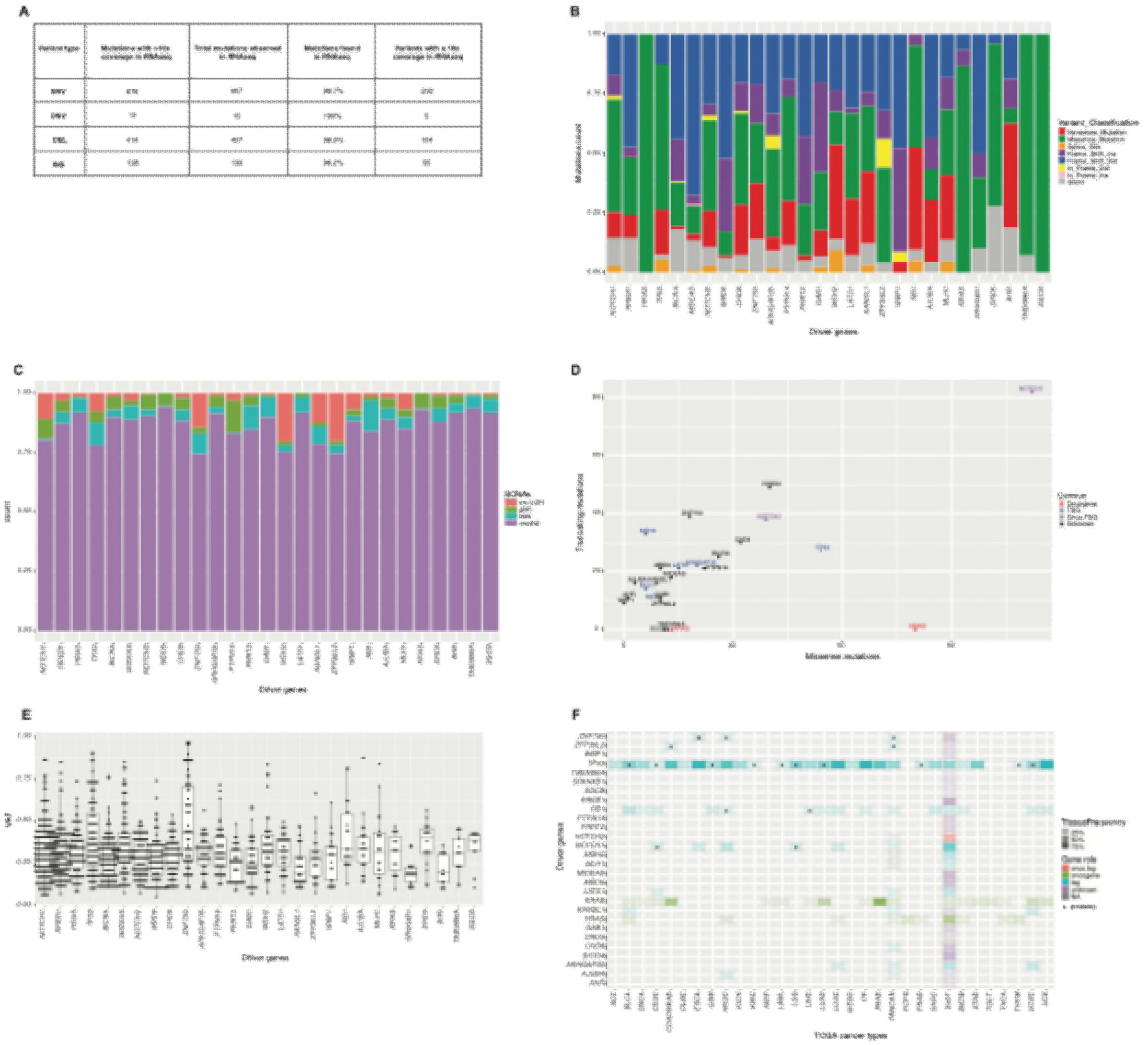

Supplementary Figure 4 – Driver genes and associated changes. (A) Table showing the proportion of somatic mutations also present in RNAseq. (B) Barplot showing the proportion of the different variant types across driver genes. (C) Barplot showing the proportion of the somatic copy number alterations (SCNAs) across autosomal driver genes. (D) Scatter plot showing missense versus truncating mutation profiles across the driver genes. (E) Barplot showing the distribution of the variant allele frequency (VAF) across the driver genes. Each dot is a tumour. Of note, *SPANXB1* is located on the X chromosome which is likely reflective of the low VAF we observe. (E) Tileplot showing the alteration of the driver genes across sebaceous tumours and pan-cancer analyses. Genes are from Bailey MH et al., 17. The gene role is taken as defined by Bailey MH et al., in 17 and COSMIC Cancer Gene Census database for TCGA cancers and sebaceous tumours, respectively. Abbreviations: NA, not defined; onco, oncogene; onco.tsg, oncogene and tumour suppressor genes; tsg, tumour suppressor gene.

2. Integrative Clustering Analysis Based on Unreliable Data

Another major concern was that the integrative clustering analysis relies on mutation data dominated by passenger mutations (not just a few as claimed by the authors). The authors did perform a more rigorous expression-based analysis for in their revised manuscript. However, instead of highlighting this new and improved analysis, they relegated it to the supplementary material, while keeping the original problematic figure in the main text. I suggest they do the opposite: put their new analysis in the main text and relegate the one based on passenger mutations in the supp mat (or consider discarding it).

> We disagree with this comment as we think there is a misunderstanding in the goals and the details of our Integrative Clustering. Methods such as MOFA or IntClust+, the ones we performed on this manuscript, do not simply perform clustering of tumours using the features as inputs, as the hierarchical clustering based on expression data mentioned by the reviewer would do. Instead, they fit a version of a factor analysis model that includes regularisation on the parameters. This means that the model “shuts off” mutations or expression values that occur randomly in tumours and not systematically in subsets of tumours.
In fact, both MOFA and IntClust were designed as methods to identify driver genes, and have been used for that task in many studies, such as identification of driver genes in triple negative breast cancer tumours (https://doi.org/10.1016/j.celrep.2023.113564), identification of key biological processes in kidney cancer (doi: 10.1038/s44320-024-00072-3), discovery of driver-based breast cancer subtypes (doi:10.1038/nature10983), driver genes discovery in lung cancer (doi: 10.1038/s41467-019-11276-9), or Crohn disease (doi: 10.1093/ibd/izaa281).
Our integrative analysis identified several driver processes, one of them characterised by overall mutation burden, and others by only a handful of mutations, typically ZNF750, TP53, RREB1 and NOTCH2. These are the genes with high values of the parameters that will contribute to the final Integrative Clustering of tumours. It is true that there can be passenger genes within the set of input features, but MOFA reduces (or sets to zero) the coefficient of these genes, having no impact in the integrative clustering. We think that our analysis shows the interplay between mutations and gene expression, providing a richer view of the driver event landscape than a simple clustering based on gene expression. Comparing both approaches, as we showed in our previous response, our integrative clustering provides additional insights.
In summary, the integrative clustering is not dominated by passenger mutations. MOFA identifies a handful of driver genes and uses only those to perform the clustering of tumours.

Conclusion

We encourage the authors to directly address our original concerns and incorporate the necessary analyses to ensure their findings are well-supported.

Reviewer #4 (Remarks to the Author):

I believe the authors have appropriately addressed all of the valid concerns raised by Reviewer #3. Regarding the previous question about 'these results may be skewed by the pipelines used' and whether 'it would be helpful if the same pipelines were applied to some examples of published historical skin tumors,' while this is a valid concern regarding potential bias arising from differences in bioinformatics pipelines, however, re-analyzing the data using the same pipeline would not eliminate systematic biases between studies. These biases also arise from variations in sequencing methods and sample preparation protocols used by different research institutes. Additionally, publicly available datasets, such as MSK-IMPACT, utilize custom panels with different targeted regions, re-analyzing these datasets using the same bioinformatics pipeline would not resolve the inherent systematic differences.

We thank Reviewer #4 for raising this important point, and we agree. Quantitative comparisons between sample groups must be treated objectively, particularly when comparing cohorts with both high and low mutational load tumors, and when data are generated on different platforms. We have therefore included a caveat in the discussion of these data and discuss them qualitatively.

NCOMMS-24-26940B: The molecular cartography of malignant and benign sebaceous tumours

Reviewer #2 (Remarks to the Author)

Despite previous requests for more robust validation of the nominated drivers, the authors persist with their original list of 28 drivers, relying solely on the outputs from dNdScv. While dNdScv is effective, it is prone to reporting false positives especially in high TMB contexts.

The original dNdScv publication (Martincorena et al., PMID: 29056346) has been cited over 1,500 times and is widely used in the field, including by the Hartwig Medical Foundation (PMIDs: 29056346, 31645765), the international PCAWG project (PMID: 33831375), and in studies of positive selection in normal tissues, such as during clonal hematopoiesis (PMID: 38744975).

Independent benchmarking across multiple cohorts—including exome sequencing datasets—has shown that dNdScv achieves a low false discovery rate and high sensitivity (e.g., PMIDs: 38808071, 31363082). We acknowledge Reviewer 2's previous comment regarding the potential impact of mutational load on false positives. To address this, we filtered our dataset by mutation count and repeated the driver gene analysis and supplied this in the previous revision of the paper. The results were consistent, confirming that our predictions are robust. While no driver gene method is perfect for samples with high mutational burden, dNdScv performs better than most alternatives due to its use of a background mutation rate–adjusted statistical model.

This is concerning as many of the nominated drivers are not established cancer-associated genes or have not been identified in large pan-cancer driver mutation analysis (for example nearly half (13/28) not recognized in a characterization of cancer driver genes in the TCGA PanCancer analysis by Bailey et al).

Reviewer 2 suggests that our list of driver genes includes false positives. We would like to clarify that the Bailey et al. dataset is not a definitive reference or ground truth. The tumours included in that study did not include sebaceous tumours—or, more broadly, adnexal skin tumors. As such, the absence of certain genes from their list may not diminish or invalidate our findings. Notably, the Bailey list also omits several well-established cancer genes, including *ETV6, POT1,* and *ALPK1,* the latter of which is a known driver in an adnexal tumour type closely related to sebaceous tumours. Our study was explicitly designed to identify novel driver genes, including those that may not have been previously reported. Filtering or disregarding genes because they do not

appear in the Bailey et al. list may risk missing biologically meaningful discoveries. In response to the specific comment about the 28 genes we report, we have annotated the list below. Importantly, 15 of these 28 genes *are* included in the Bailey et al. list. The other genes are tightly linked to tumorigenesis. We believe this supports the relevance of our findings and underscores the importance of considering tumor-type–specific contexts when evaluating driver gene predictions.

All tumours	
1 TP53 0.000000e+00	On the Bailey et al., list
2 HRAS 0.000000e+00	On the Bailey et al., list
3 NOTCH1 0.000000e+00	On the Bailey et al., list
4 MSH2 0.000000e+00	On the Bailey et al., list
5 ZNF750 0.000000e+00	On the Bailey et al., list
6 RREB1 0.000000e+00	On the Bailey et al., list
7 NOTCH2 1.219088e-12	On the Bailey et al., list
8 AJUBA 4.527517e-06	On the Bailey et al., list
9 CHD8 2.043482e-05	On the Bailey et al., list
10 LATS1 5.479646e-05	In a biological pathway/process annotated by Bailey etal (see Table S2)
11 KANSL1 8.490699e-05	On the Bailey et al., list
12 MLH1 4.473292e-04	On the Bailey et al., list
13 KRAS 1.035442e-03	On the Bailey et al., list
14 PTPN14 1.058224e-03	A negative regulator of YAP (PMID: 23613971) whose disruption can transform mammary cells.
15 ZFP36L2 1.758428e-03	On the Bailey et al., list
16 WBP1 3.908975e-03	WBP1 regulates mitochondrial function and ferroptosis (PMID: 40075333)
17 ARHGAP35 3.908975e-03	On the Bailey et al., list
18 AHR 4.234483e-03	Numerous papers showing this is a driver. For example PMID: 40222634 and PMID: 35710704. These papers published after Bailey et al.,
19 RB1 1.136855e-02	On the Bailey et al., list
20 BICRA 1.379002e-02	Many papers on BICRA s role in cancer. For example PMID: 33232675, PMID: 35218185 , PMID: 31832310, PMID: 30405818 .
21 MBD6 1.506188e-02	In the same complex as BAP1/AXSL1, known tumour suppressors (PMID: 36180891). New driver this study.
22 GAB1 3.386166e-02	Fusion driver in soft tissue tumours (PMID: 34036664). Alterations activate the AKT/MAPK/JAK pathway.
23 TMEM86A 5.809764e-02	Hydrolyzes lysoplasmalogens—lipids involved in membrane structure and signaling. Link in other cancers to be examined.
24 MIDEAS 5.809764e-02	Linked it melanoma (PMID: 32591534). Involved in chromatin regulation.
25 SGCB 6.491304e-02	No reported link to cancer. New driver from this study.
3 SPANXB1 0.07962803	Experimental evidence that this gene can function as a driver. PMID: 31406142
2 DRD5 0.051372991	Previously linked to treatment resistance in brain cancer (PMID: 30559168).
11 PRRT2 6.754382e-02	Linked to glioma proliferation (PMID: 28192116). Reported as a mismatch repair hotspot where it has been shown as a functional driver(PMID: 27907910).

The authors do mention using oncodriveFML as a complementary tool in their methods section, yet these results are never discussed in the manuscript, nor are they integrated with those from dNdScv. The raw dNdScv output is still not included in this revision as originally requested.

We thank Reviewer 2 for this comment. Firstly, we provide the oncodriveFML analysis in the paper and point readers at this list in the manuscript. We do not discuss it extensively because driver gene analysis is just one part of the paper but, as requested, we provide this analysis as a complement to dNdScv. We do not feel that it is valuable to integrate driver predictions from both methods because there is no clear rubric as to how to do this and there is no way of knowing if we have over filtered the data or introduced errors. The raw dNdScv output was provided with the revision we returned (see NatureComs_dNdScv_Output.xls).

Despite clear evidence that indicates SPANXB1 is a false positive, the authors continue to nominate it as a driver gene. They incorrectly attribute the unusually low variant allele frequency (VAF) of its mutations to its location on the X chromosome in their revised manuscript. Indeed, VAF of mutations on the X chromosome should be twice that of heterozygous mutations on autosomes in males.

There are many genes that have subclonal drivers, for example low VAF *EGFR* (PMID: 33869038) and *NOTCH1* (PMID: 35430388) mutations are common. We cannot exclude a gene from an analysis because we can't explain how it is contributing to tumorigenesis. We suspect that the reason why this gene has a significant P value is that it mainly has frameshift alterations. It is only called a driver in extra-ocular SC with a borderline P value, but as above we do not feel that it is appropriate to exclude data from an analysis when we have no technical evidence suggesting that the result is not correct. The statement about VAF is only correct in males. Reviewer 2 will note that although *SPANXB1* is called as a driver we have now focused our analysis on drivers on the autosomes, so it does not feature in any of the downstream analyses. We hope that this strikes a balance between transparency and pragmatism given Reviewer 2s comment.

In Supplementary Table 5B, where the results of a driver analysis were supposedly restricted to drivers identified by Bailey et al., genes such as TTN, OBSCN, and NEB with extremely low p-values were included. These genes are known for accumulating passenger mutations due to their length and are not listed among the drivers in Bailey et al. study. This discrepancy indicates that either the analysis was not conducted properly or the incorrect results were presented.

We acknowledge that we made a mistake with this analysis, and we thank Reviewer 2 for noticing this. We have corrected the table and supply it with the revised manuscript.

Fig S4A contains obvious errors. How could 676 SNV mutations be observed with >10X coverage if the total number of SNV mutations is 667? The authors also missed the point since the idea here was to show that most mutations in *each* of the nominated drivers are observed in the RNA-seq, in contrast to passenger mutations in other genes that are often not expressed. As is, the table does not inform about the individual plausibility of the nominated drivers. Figure S4C and S4E also miss the point and do not inform the plausibility of the nominated drivers. The idea was to show if mutations often co-occur with cnLOH or cnLoss in nominated TSGs (and hence have higher VAF), in contrast to passenger mutations in *other* genes, as would be expected. Additionally, in Figures S4B and S4C, the y-axis is inaccurately labeled as 'count'.

There are 676 variants that are in driver genes with RNA-Seq >10x. Of these 676 variants, 667 variants (called from the exome data) were re-called (98.7%) in the RNA-Seq data.

Given that >98.7% of all SNV mutations validated (as above) we feel that we answered the reviewer's question as written. i.e. the analysis reveals that we can see the driver events in the deep RNA-Seq data we generated.

Similarly, with Figure S4C and E we delivered the analysis requested by the reviewer, so we are unsure of what further to provide.

Please note, we are absolutely committed to responding to reviewer 2s comments, but we cannot interpret what they are requesting unless they provide a full description.

The purpose of these additional analyses we requested was to identify which candidate drivers are substantiated by orthogonal evidence and to pinpoint likely false positives. Unfortunately, the authors chose to present inconsequential data and assert that their initial list of drivers remains valid without requiring any modifications.

We thank you for your attention to detail. We certainly didn't intend to present data that Reviewer 2 didn't feel answered the question. We genuinely don't know what we haven't done here.

I do still think this is an important resource paper to the cancer genomics community.

We thank Reviewer 2 for their help in improving our manuscript.

Reviewer #5 (Remarks to the Author):

I have gone through the questions from Reviewer #2 and the authors' responses. Although some of the points are addressed in detail, there are still some points that require clarification.

a) In the author's response, it was mentioned that 15 out of 28 predicted drivers are present in the TCGA PanCancer analysis by Bailey et al., and the remaining ones are linked to tumorigenesis based on literature evidence. I think this is informative, and the authors may consider providing this information in the supplementary table and also mentioning this overlap number in the results section.

> We thank Reviewer #5 for its comment and have amended the manuscript accordingly. The data is in Supplementary Table 5B.

b) The results of restricted hypothesis testing using Bailey et al., (299 cancer driver genes) reported in Supplementary Table 5B still show genes such as TTN, OBSCN, and NEB, which are likely false positives. The authors stated that they were removed, but they're not reflected in the table yet.

> These genes are not in the table – after the comments from Reviewer 2 they were removed.

c) The results of oncodriveFML were not directly included in the supplementary table, so it is difficult to compare the results. The GitHub link to the oncodriveFML output is also not working. It would be helpful for the readers to know at least what fraction of the 28 potential drivers predicted by the dNdScv overlap with the oncodriveFML significant hits. In particular, whether the four drivers (NOTCH1, RREB1, HRAS, TP53) used for molecular subtyping are supported by oncodriveFML or not.

> We thank Reviewer #5 for this comment. The results of oncodriveFML have been put on the GitHub repository. Out of the 25 driver genes called in the entire cohort by dNdScv, 22 were also significantly found by oncodriveFML (88%) (please see "oncodrivefml_variants-oncodrivefml_dNdScvComparison"). The last 3 genes found in specific groups could not be confirmed as we did not run oncodriveFML that way.
